# Cobalt-Catalyzed Aerobic Aminocyclization of Unsaturated Amides for the Synthesis of Functionalized γ- and δ-Lactams

**DOI:** 10.1021/acs.orglett.3c02390

**Published:** 2023-08-23

**Authors:** Manuel Freis, Moritz Balkenhohl, David M. Fischer, Tony Georgiev, Roman C. Sarott, Erick M. Carreira

**Affiliations:** ETH Zürich, Department of Chemistry and Applied Biosciences, Laboratory of Organic Chemistry, 8093 Zürich, Switzerland

## Abstract

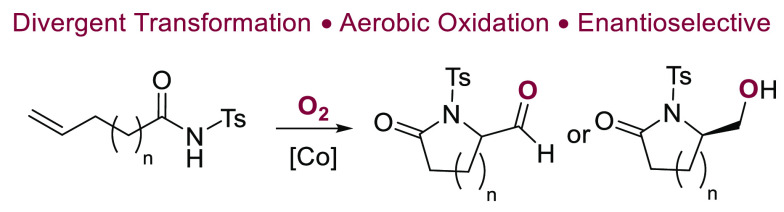

We report the cobalt-catalyzed aminocyclization of unsaturated *N*-acyl sulfonamides in the presence of oxygen to provide γ- and δ-lactam aldehydes. Use of an optically active cobalt catalyst resulted in the formation of enantiomerically enriched γ-and δ-lactam alcohols. The γ-lactam aldehydes and alcohols obtained were elaborated into useful building blocks.

Lactams, in particular γ- and δ-lactams, constitute integral parts of numerous natural products and pharmaceuticals.^1^ For example, they are a key structural feature of antibiotics,^2^ HIV-1 integrase inhibitors,^3^ antitumor agents,^4^ and antidepressants,^5^ as well as drugs for the treatment of type 2 diabetes (Scheme 1).^6^ Herein, we report novel cobalt-catalyzed aerobic cyclization reactions of unsaturated *N*-acyl sulfonamides **1**, which provide formyl (**2**) and hydroxymethyl (**3**) γ- and δ-lactams. The transformation proceeds with cobalt complexes under an oxygen atmosphere in toluene over 2 h in the presence of molecular sieves. Depending on the workup conditions, aldehydes or primary alcohols may be isolated.

**Scheme 1:**
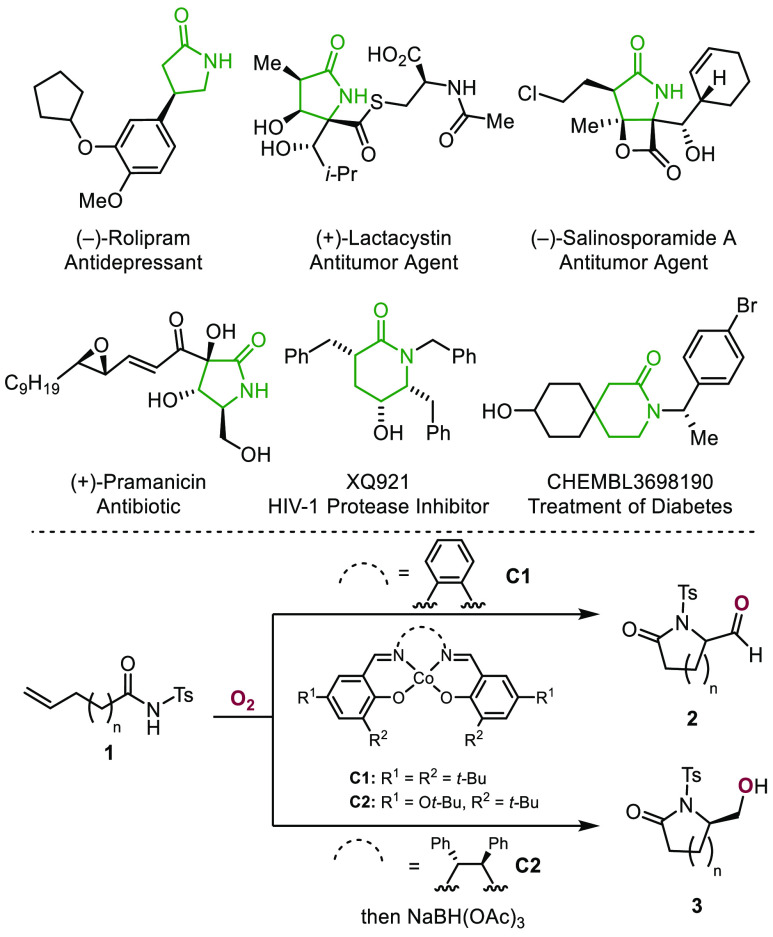
Biologically Active γ- and δ-Lactams and Aminocyclization of Unsaturated Amides

Cycloisomerization reactions of alkenyl *N*-acyl sulfonamides mediated by gold,^7^ niobium,^8^ iridium,^9^ and zinc catalysts^10^ provide access to *N*-sulfonyl lactams (Scheme 2). Oxidative cyclizations of γ-unsaturated *N*-aryl carboxamides have also been described.^11^ For example, aminofluorination of alkenyl *N*-aryl amides leads to 5-fluoromethyl-substituted γ-lactams.^12^ Additionally, photoredox catalysis has been employed to effect aminofluorosulfonylation and amidoarylation of unactivated olefins.^13^ Recently, aminocyanation of *N*-aryl-pent-4-enamides with trimethylsilyl cyanide to provide cyano-containing γ-lactams has been reported.^14^

**Scheme 2:**
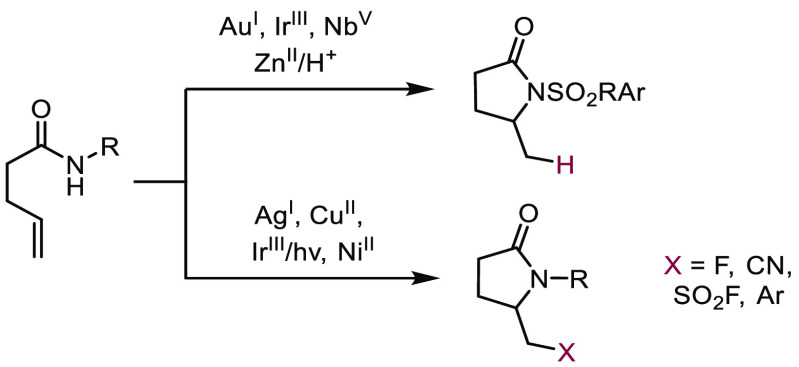
Prior Work: Cyclization of Unsaturated *N*-Acyl Sulfonamides and Unsaturated *N*-Aryl Amides

In previous studies, we disclosed manganese- and cobalt-catalyzed cyclization reactions of unsaturated hydrazones to access a range of complex and highly functionalized pyrazolines.^15^ We subsequently became interested in expanding the transformation beyond hydrazones. On the basis of reactivity observed for *N*-acyl sulfonamides in related transformations,^16^**1a** was subjected to a collection of cobalt catalysts in the presence of oxygen and 4 Å molecular sieves (Table 1). Initially, *N*-acyl sulfonamide **1a** was heated to 55 °C for 12 h in the presence of salcomine under an oxygen atmosphere (1 atm) in toluene (0.1 M). Aldehyde **2a** was obtained in 8% yield in addition to unreacted starting material (Entry 1). When the reaction temperature was increased to 105 °C after 2 h, 45% of **2a** could be isolated (Entry 2). The use of related cobalt catalysts **C3** and **C4** did not lead to an increased yield of product (Entries 3 and 4). Elevating the temperature to 110 °C decreased the yield to 41% (Entry 5). Finally, when cobalt salen catalyst **C1** was used, aldehyde **2a** was obtained in 52% yield (Entry 6). Reducing the catalyst loading of **C1** from 20 to 5 mol % increased the yield of product **2a** to 63% (Entry 7). In the absence of a catalyst, no product formation was observed (Entry 8).

**Table 1:**
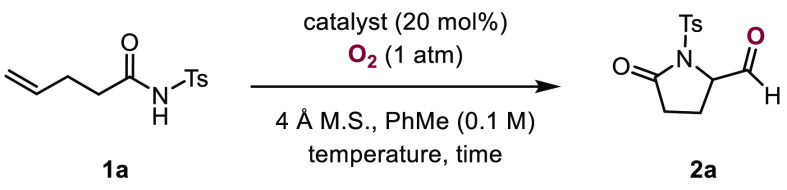
Selected Optimization Results for the Cobalt-Catalyzed Cyclization of *N*-Acyl Sulfonamide **1a**

entry	catalyst	temperature, time	yield^a^
1	salcomine	55 °C, 12 h	8%
2	salcomine	105 °C, 2 h	45%
3	**C3**	105 °C, 2 h	45%
4	**C4**	105 °C, 2 h	38%
5	**C3**	110 °C, 2 h	41%
6	**C1**	105 °C, 2 h	52%
**7**	**C1** (5 mol %)	**105 °C, 2 h**	**63%**
8	none	105 °C, 2 h	0%

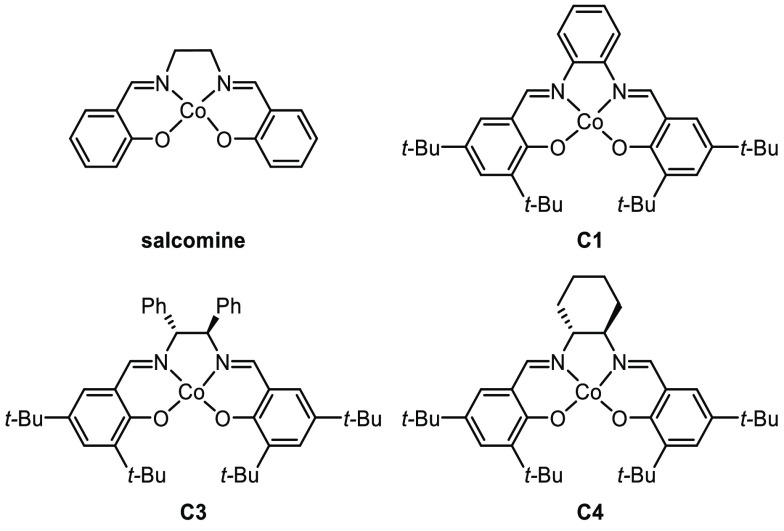

a Isolated yields.

With the optimized reaction parameters in hand, the scope of the cyclization reaction was investigated (Scheme 3). Various unsaturated *N*-acyl sulfonamides **1a**–**n** were subjected to the optimized reaction conditions, and γ- and δ-lactam aldehydes **2a**–**n** were obtained. Substrates incorporating methyl and phenyl substituents in the α- and β-position led to products **2b** and **2f** in 36–61% yield. We next evaluated the formation of spiro-γ-lactams. Gratifyingly, **2g**–**2i** were obtained in 46–64% yield. Fused cyclohexane-γ-lactam **2j** was produced in 28% yield. We then extended the transformation to δ-lactams, furnishing **2k** and dihydroisoquinolinone **2l** in 34% and 50% yield, respectively. Pyrrole containing δ-lactam **2m** was accessed from the corresponding *N*-acyl sulfonamide in 40% yield. To expand the scope beyond *N*-tosyl sulfonamides, *N*-methoxyphenyl sulfonyl protected amide **1n** was subjected to the reaction conditions, giving rise to γ-lactam **2n** in 66% yield.

**Scheme 3:**
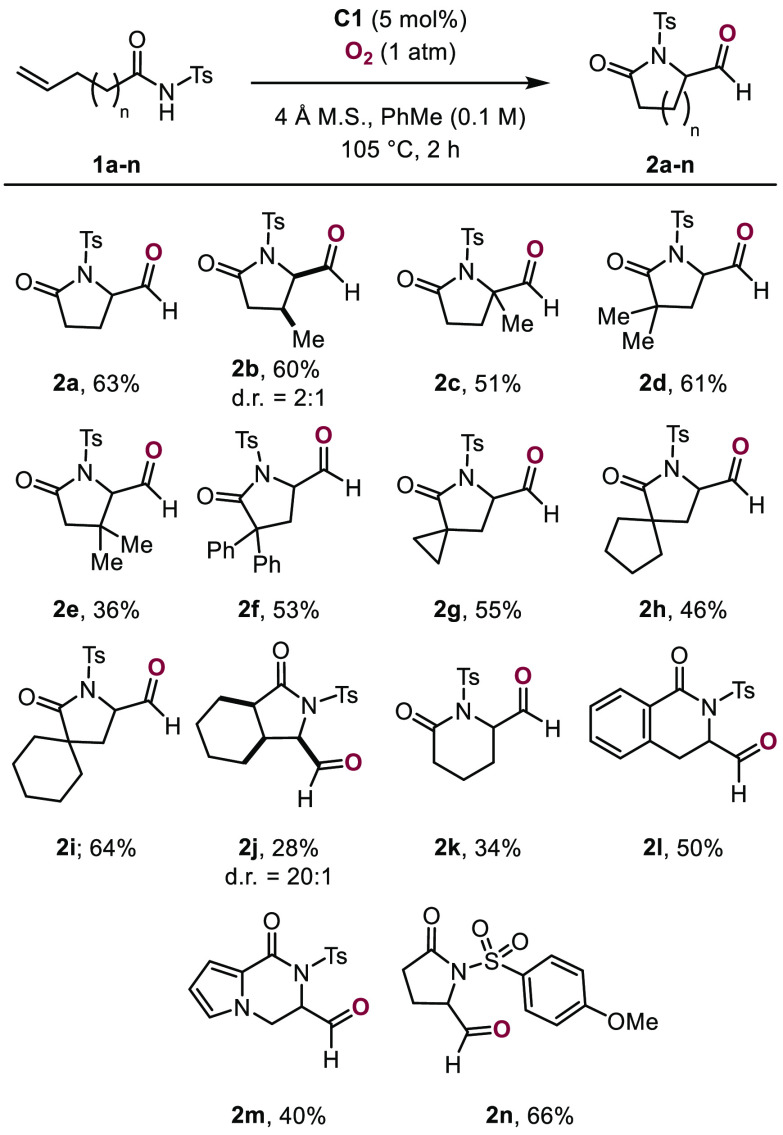
Substrate Scope for the Cobalt-Catalyzed Aminocyclization of Unsaturated *N*-Acyl Sulfonamides

Cobalt-salen catalysts have previously been employed in enantioselective transformations.^17^ Consequently, we sought to examine whether the use of a chiral salen ligand would render the transformation we developed enantioselective. To this end, **1a** was subjected to cobalt catalyst **C2** under conditions otherwise identical to those described above (O_2_, 4 Å molecular sieves, PhMe, 105 °C). Under these conditions, formation of product **2a** was observed in 40% yield and 70:30 e.r.^18^

We reasoned that at high temperatures, epimerization of the product aldehyde may readily occur, prompting us to conduct the reaction at lower temperatures in an attempt to abate the erosion of e.r. When the reaction with **1a** was carried out at 80 °C, a mixture of aldehyde **2a** and alcohol **3a** was obtained. This mixture was subsequently reduced *in situ* by the addition of acetic acid (1.0 equiv) and sodium triacetoxyborohydride (1.1 equiv). Gratifyingly, the alcohol product was thus obtained in 63% yield and 83:17 e.r. (Scheme 4). We demonstrated that when the reaction temperature was lowered further to 55 °C and the reaction was conducted for 2 h the enantiomeric ratio could be improved to 90:10, albeit at a diminished yield of 25%. Conducting the reaction at 55 °C for 48 h led to formation of **3a** in 72% yield and 86:14 e.r., which does not constitute a marked benefit over the original conditions (80 °C, 2 h).

**Scheme 4:**
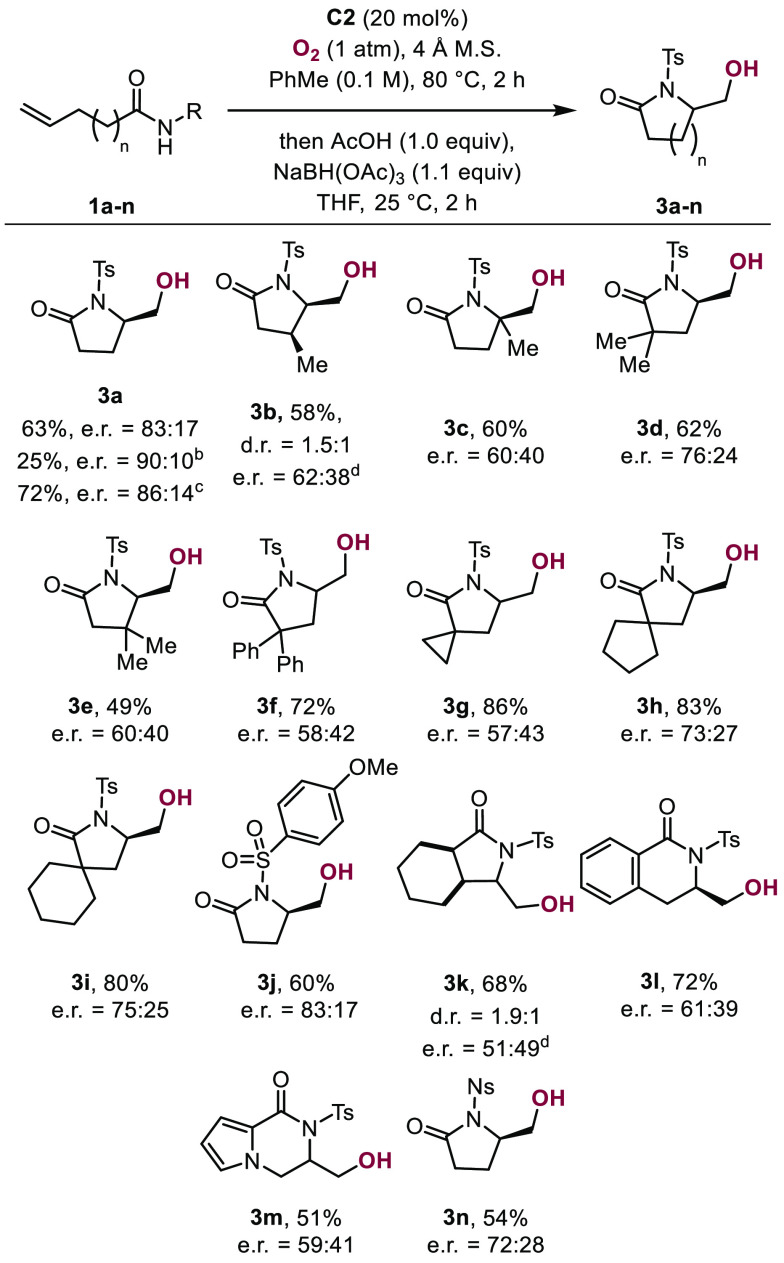
Substrate Scope of the Cobalt-Catalyzed Aminocyclization with Subsequent Reduction Enantiomeric ratios were determined via chiral HPLC or SFC by comparison with racemic samples (see SI). For the enantioenriched reaction it was found, that catalyst C2 required higher catalyst loadings of 20 mol %. Reaction conditions: (b) 55 °C, 2 h; (c) 55 °C, 48 h. (d) e.r. of the major diastereomer.

We next examined the substrate scope of the aminocyclization reaction. Subjecting **1b**–**1f** to reaction conditions at 80 °C followed by reductive workup resulted in the formation of γ-lactams **3b** to **3f** bearing methyl and phenyl groups in 49–72% yield and up to 76:24 e.r. *N*-Methoxyphenyl sulfonyl protected aminoalcohol **3j** was accessed from the corresponding sulfonamide in 60% yield (e.r. = 83:17). Spiro-fused γ-lactam alcohols **3g** to **3i** were obtained in 80–86% yield (e.r.= 57:43 to 75:25). Bicyclic γ-lactam **3k** and fused δ-lactams **3l** and **3m** were isolated in 68% (d.r. = 1.9:1), 72% (e.r. = 61:39), and 51% yields (e.r. = 59:41), respectively. Finally, product **3n** bearing an *N-*nosyl group was obtained in 54% yield with 72:28 e.r.

To showcase the versatility of the lactams, they were subjected to a variety of product derivatization reactions (Scheme 5). Treatment of **3a** with sodium borohydride resulted in a reductive ring opening, providing diol **4** in 85% yield. When aldehyde **2a** was subjected to the Ohira–Bestmann reagent in the presence of K_2_CO_3_ in methanol, ester **5** was obtained in 76% yield. Removal of *N*-protecting groups can be challenging, which led us to showcase said reaction for one of the products obtained in this transformation. To this end, when γ-lactam **3n** was subjected to a combination of thiophenol (3.0 equiv) and K_2_CO_3_ (4.0 equiv), removal of the *N*-nosyl group was effected and free amide **6** obtained in 75% yield

**Scheme 5:**
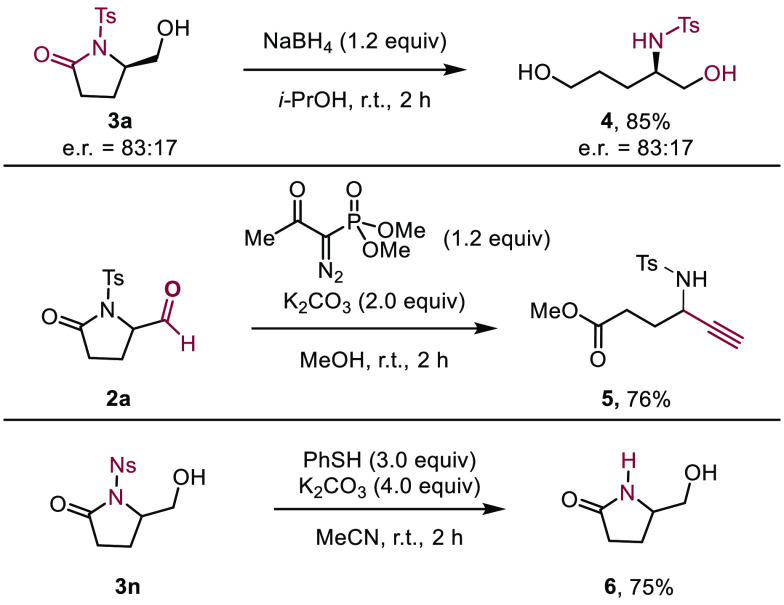
Selected Derivatization of γ-Lactam Aldehydes and Alcohols

In conclusion, we have reported the cobalt-catalyzed aminocyclization of unsaturated *N*-acyl sulfonamides to give access to a range of formyl and hydroxymethylated γ- and δ-lactams. Aerobic cobalt catalysis led to the formation of γ- and δ-lactam aldehydes, whereas the use of chiral cobalt catalyst **C2** with reductive workup gave rise to enantiomerically enriched γ- and δ-lactam alcohols. Finally, derivatizations of the prepared products, including ring opening and *N*-nosyl deprotection, were carried out, demonstrating the versatility of the products derived from the cyclization protocol.

## Data Availability

The data underlying this study are available in the published article and its Supporting Information.

## Supporting Information Available

Experimental procedures and characterization data for all new compounds (PDF)
