# Supplementary material for: Cobalt-Catalyzed Aerobic Aminocyclization of Unsaturated Amides for the Synthesis of Functionalized γ- and δ-Lactams
Source: Org Lett. 2023 Aug 23;25(34):6380–4. doi: 10.1021/acs.orglett.3c02390 (PMC10476186; doi:10.1021/acs.orglett.3c02390)
Supplement: Supplementary file 1 — ol3c02390_si_001.pdf [file ol3c02390_si_001.pdf]

Supporting Information

Cobalt-Catalyzed Aerobic Aminocyclization of Unsaturated Amides for the Synthesis of Functionalized γ- and δ-Lactams

Manuel Freis, Moritz Balkenhohl, David M. Fischer, Tony Georgiev, Roman C. Sarott and Erick M. Carreira*

Table of Contents

1. General Remarks 2

2. General Procedures 4

3. Optimization Studies 6

4. Preparation of Cobalt Catalysts 8

5. Starting material synthesis (unsaturated *N*-acyl sulfonamides) 14

6. Racemic aldehyde synthesis 15

7. Enantioenriched alcohol synthesis 29

8. Product derivatization 45

9. Mosher ester studies 48

10. Chiral Chromatography Data 53

11. ^{1}H and ^{13}C NMR spectra 68

1. General Remarks

Procedure

Unless otherwise noted, all reactions were carried out under nitrogen atmosphere in glassware dried with a heat gun (650 °C) under high vacuum (<1 mbar). Syringes which were used to transfer anhydrous solvents or reagents were purged thrice with nitrogen prior to use. All reagents were purchased from commercial suppliers (ABCR, ACROS, Sigma Aldrich, Fluka, TCI, Strem, Alfa, Combi-Blocks or Fluorochem) and used without further purification.

Chemicals

All reagents were purchased from commercial suppliers (ABCR, ACROS, Sigma Aldrich, Fluka, TCI, Strem, Alfa, Combi-Blocks or Fluorochem) and purified where appropriate. Anhydrous solvents over molecular sieves (4Å) were purchased from Acros and used as received. 4Å molecular sieves were activated in a microwave (10 x 60 s, 600 W) and cooled under vacuum in a Schlenk flask.

Thin-Layer Chromatography

For reaction controls TLC glass plates from Supelco® (TLC silica gel 60 F254: 25 glass plates, 20 x 20 cm) were used. Spotted substances were made visible by exposure to ultraviolet light (254 nm or 365 nm) or TLC stain (aqueous potassium permanganate solution or aqueous ceric ammonium molybdate solution followed by heating).

Column Chromatography

For column chromatography Sigma-Aldrich silica gel sorbent (high purity grade (9385), 230-400 mesh particle size, pore size 60) was used as a stationary phase. When stated, neutral silica from Nacalai Tesque (Silica Gel 60, spherical, neutral, catalog No. 30511-51) was used.

Nuclear Magnetic Resonance Spectroscopy

All NMR spectra were measured in deuterated solvents at room temperature with a Bruker Avance 400 (400 MHz, equipped with 9.4 T magnet and BBFO probe), Bruker Ascend 400 (400 MHz, equipped with 9.4 T magnet and BBFO probe), Bruker

Ultrashield 400 (400 MHz, equipped with 9.4 T magnet and BBFO probe), Oxford 400 (400 MHz, equipped with 9.4 T magnet and BBFO probe) or Bruker Avance 500 (500 MHz, equipped with 11.7 T magnet and a BBFO probe). The chemical shifts are referenced to the solvent residual signal ($CDCl_3$, 1H: δ = 7.26 ppm, ^{13}C: δ = 77.16 ppm; DMSO-d_6, ^{1}H: δ = 2.50 ppm, ^{13}C: δ = 39.52 ppm) and reported in parts per million (ppm). The following abbreviations are used in reporting NMR data: s = singlet, d = doublet, t = triplet, q = quartet, b = broad, dd = doublet of doublets, m = multiplet, etc.

High-Resolution Mass Spectrometry

All mass spectra were measured by the mass spectrometry service of the Laboratory of Organic Chemistry at ETH Zurich on a Bruker Daltonics maXis ESI-QTOF or a Bruker Daltonics maXis II ESI-QTOF.

IR Spectroscopy

Infrared spectra were recorded on a Perkin Elmer Two FT-IR spectrometer as thin films. Absorptions are given in wavenumbers (cm^{-1}).

Enantiomeric Ratio

Enantiomeric ratios were determined on a Jasco2080Plus supercritical fluid chromatography (SFC), Waters Acquity UPC2 (SFC), Waters e2695 (HPLC).

Optical rotations

Optical rotations were measured with Jasco P-2000 Polarimeter, 10 cm, 1.5 mL cell.

Structural assignments

Structural assignments were made with additional information from gCOSY, gHSQC, and gHMBC experiments.

2. General Procedures

General procedure 1: Cobalt-Salen Complexation

To a flame-dried round-bottom flask equipped with a magnetic stirring bar was added $Co(OAc)_2 \cdot 4H_2O$ (1.0 equiv). The flask was heated to 80 ˚C under vacuum until the color changed from pink to purple (approx. 10 min). The flask was left to cool to room temperature, opened to air, and salen ligand (1.0 equiv) was added. The flask was set under N_2 atmosphere and degassed ethanol (0.04 M) was added. The solution was heated to 80 ˚C and was stirred for 16 h. The mixture was cooled to 0 ˚C and left to stir for 20 min. The solid precipitate was isolated by vacuum filtration and washed with cold ethanol. The solid was further dried under vacuum overnight to yield the cobalt-salen catalyst.

Note: It is sufficient to degas ethanol (anhydrous) by sparging with N_2 for 30 min prior to use.

General procedure 2: Racemic aldehyde synthesis

An oven dried (110°C) 5 mL crimp vial was equipped with a magnetic stirring bar and was cooled to r.t. under vacuum. The vial was charged with catalyst **C1** (5 mol%), 4 Å molecular sieves, and *N*-acyl-sulfonamide (1.00 equiv). The vial was set under vacuum followed by addition of O_2 atmosphere via O_2-balloon. Toluene (0.1 M) was added, the solution was purged with O_2 for 1 min, and the vial was set in a preheated (105 °C) heating block and stirred for 2 h. The solvent was removed under reduced pressure to yield the crude product which was purified by flash silica gel column chromatography using the appropriate eluent.

General procedure 3: Enantioenriched alcohol synthesis

An oven dried (110°C) 5 mL crimp vial was equipped with a magnetic stirring bar and was cooled to r.t. under vacuum. The vial was charged with catalyst **C2** (20 mol%), 4 Å molecular sieves, and unsaturated *N*-acyl sulfonamide (1.00 equiv). The vial was set under vacuum followed by addition of O_2 atmosphere via O_2-balloon. Toluene (0.1 M) was added, the solution was purged with O_2 for 1 min, and the vial was put in a preheated (80 °C) heating block and stirred until full consumption of starting material was observed by TLC. The vial was then opened to air and THF (0.1 M) was added,

followed by acetic acid (1.00 equiv) and sodium triacetoxyborohydride (1.10 equiv). The vial was capped with a septum and the atmosphere was briefly purged with N_2. The mixture was left to stir until complete aldehyde reduction was observed, upon which it was transferred to a separatory funnel and diluted with ethyl acetate (30 mL). The organic layer was washed with water and the aqueous layer was extracted with ethyl acetate (2 x 30 mL). The combined organic phases were dried over anhydrous $MgSO_4$, filtered, and concentrated under reduced pressure to yield the crude product, which was purified by flash silica gel column chromatography using the appropriate eluent.

3. Optimization Studies

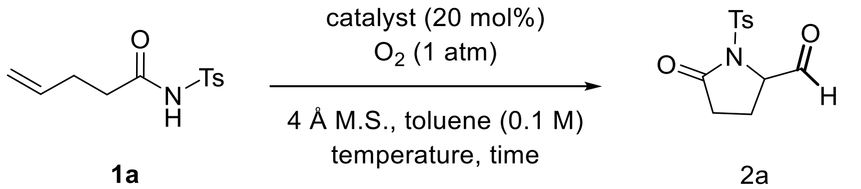

entry	catalyst	temperature, time	**yield 2a (%)**[1]
1	Mn(dpm)$_3$	55 °C, 12 h	2%
2	**C5**	55 °C, 12 h	-
3	salcomine	55 °C, 12 h	8%
4	salcomine	105 °C, 2 h	45%
5	**C5**	105 °C, 2 h	-
6	**C3**	105 °C, 2 h	45%
7	**C3**	110 °C, 2 h	41%
8	**C4**	105 °C, 2 h	38%
9	**C1** (5 mol%)	105 °C, 2 h	63%
10	**C1**	105 °C, 2 h	52%

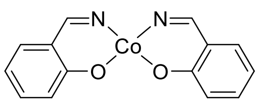

Salcomine

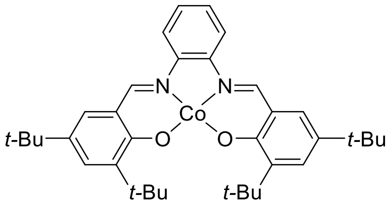

C1

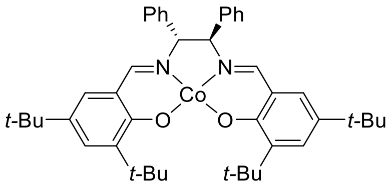

C3

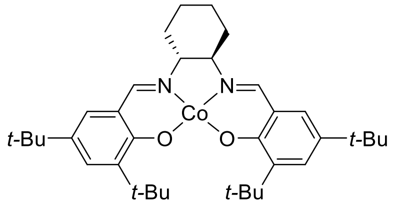

C4

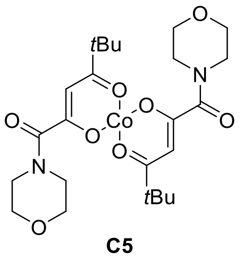

C5

[1] Isolated yields

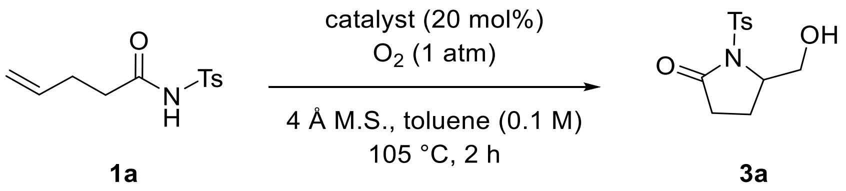

entry	catalyst	yield **3a** (%)	e.r.
1	**C3**	45	70:30
2	**C4**	38	70:30
3	**C6**	34	67:33
4	**C1**	27	51:49
5	**C7**	8	-
6	**C8**	-	-
7	**C9**	-	-
8	**C2**	63	83:17

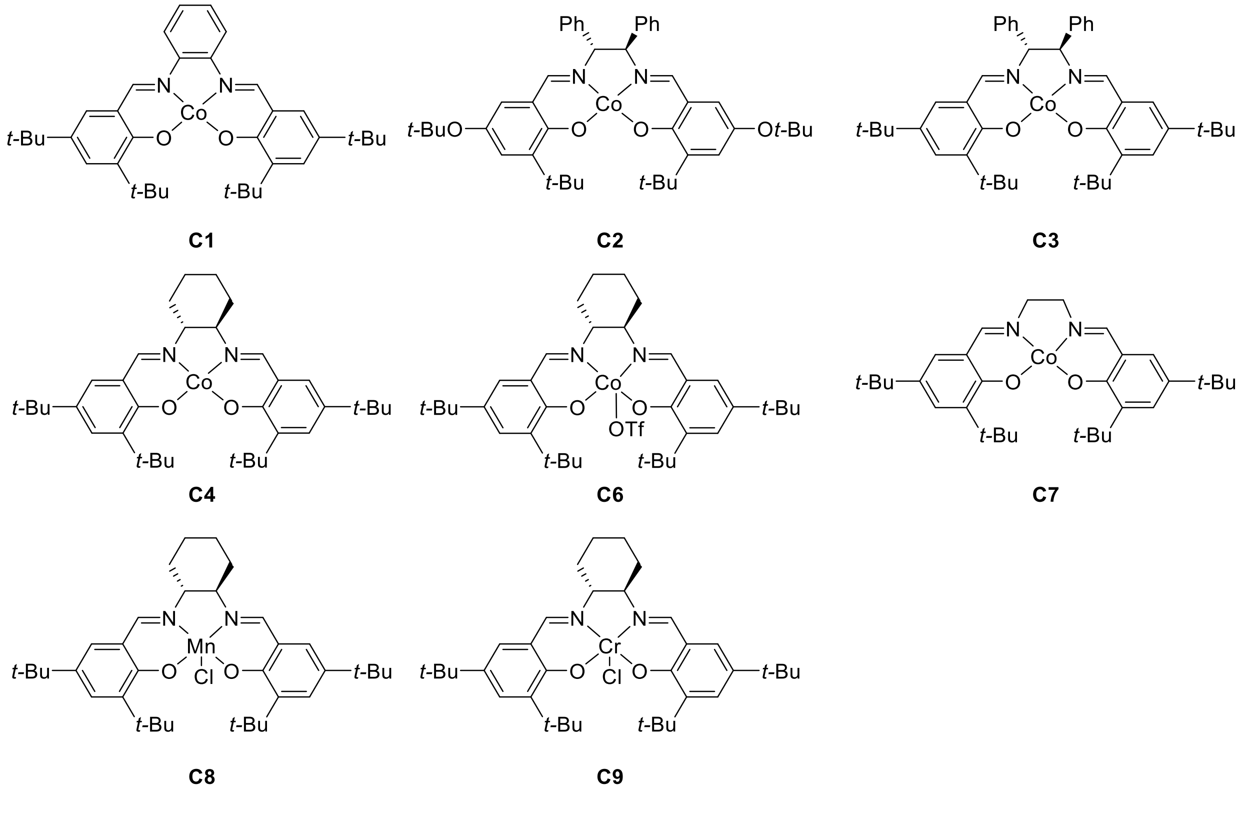

4. Preparation of Cobalt Catalysts

Cobalt Catalyst (C1)

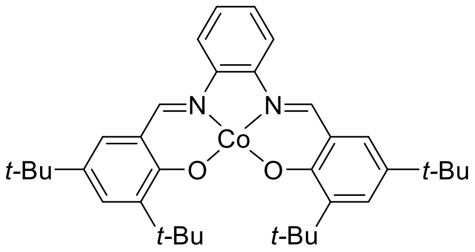

C1

Cobalt Catalyst **C1** was prepared according to literature.[2]

Chiral Cobalt Catalyst (C2)

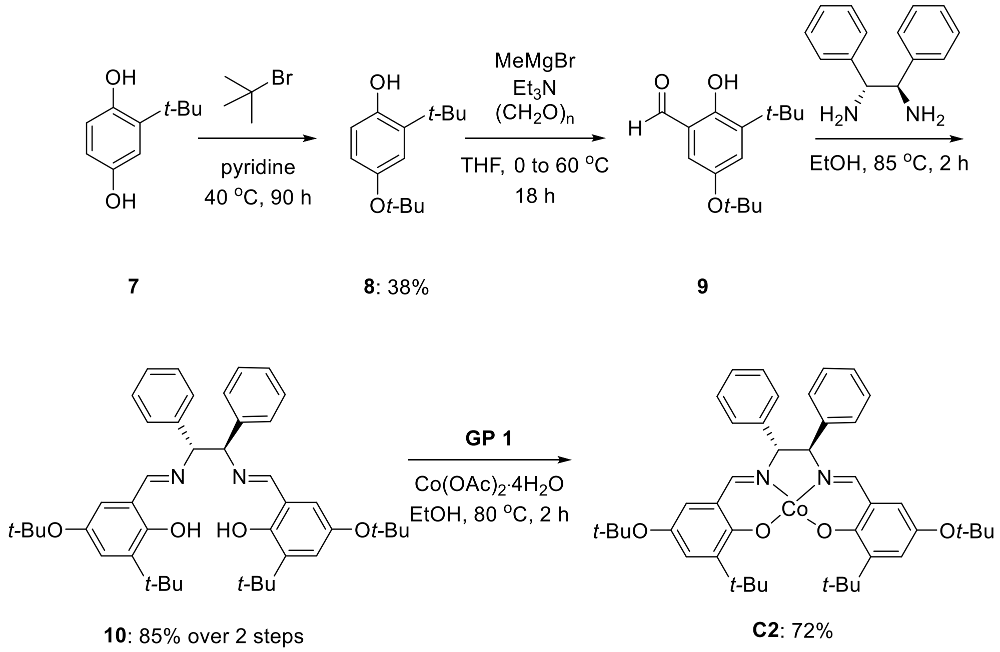

Scheme 1. Synthesis of Chiral Cobalt Catalyst (**C2**)

---

[2] Shimakoshi, H.; Hirose, S.; Ohba, M.; Shiga, T.; Okawa, H.; Hisaeda, Y. *Bulletin of the Chemical Society of Japan* **2005**, *78*, 1040–1046.

4-(*Tert*-butoxy)-2-(*tert*-butyl)phenol (8)

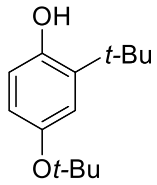

TBHQ (24.9 g, 150 mmol, 10.0 equiv) was submitted to a 100 mL round-bottom flask equipped with a magnetic stirring bar. Pyridine (5.00 mL, 61.8 mmol, 4.12 equiv) was added, leading to the formation of a slurry, to which 2-bromo-2-methylpropane (1.70 mL, 15.0 mmol, 1.00 equiv) was added and the mixture was heated to 40 °C and stirred for 90 h. The slurry was diluted with ethyl acetate (200 mL) and the organic phase was washed with 1M HCl (50 mL), brine (50 mL), dried over anhydrous $MgSO_4$, filtered and concentrated under reduced pressure to yield the crude material which was purified *via* silica gel column chromatography (hexane:ethyl acetate = 9:1) to give **8** as a colorless solid.

Yield: 1.27 g, 5.72 mmol, 38%

^{1}H NMR (400 MHz, $CDCl_3$): δ (ppm) = 6.89 (d, J = 2.8 Hz, 1H), 6.69 (dd, J = 8.4, 2.8 Hz, 1H), 6.53 (d, J = 8.4 Hz, 1H), 4.79 (s, 1H), 1.38 (s, 9H), 1.30 (s, 9H).

^{13}C NMR (101 MHz, $CDCl_3$): δ (ppm) = 150.5, 148.2, 136.5, 123.7, 122.3, 116.4, 78.0, 34.6, 29.6, 28.8.

IR (thin film, cm^{-1}): 3324, 2976, 2950, 1507, 1415, 1391, 1367, 1195, 1158, 948, 905, 854.

HRMS (ESI, *m/z*): calcd for $C_{14}H_{22}NaO_2$ $[M+Na]^+$: 245.1512, found: 245.1505.

m.p. (°C): 158−160.

6,6'-((1*E*,1'*E*)-(((1*R*,2*R*)-1,2-diphenylethane-1,2-diyl)bis(azaneylylidene))bis(methaneylylidene))bis(4-(tert-butoxy)-2-(*tert*-butyl)phenol) (10)

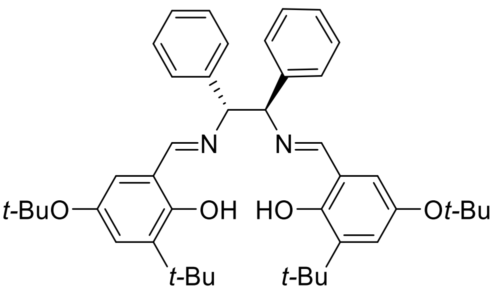

To an oven dried Schlenk flask equipped with a magnetic stirring bar was added phenol **8** (1.22 g, 5.50 mmol, 1.00 equiv). The flask was placed under nitrogen, dry THF (13.8 mL, 0.4 M) was added and the resulting solution was cooled to 0 °C. MeMgBr (3.0 M in Et_2O, 2.83 mL, 8.50 mmol, 1.50 equiv) was added dropwise *via* syringe over 5 min. The reaction mixture was warmed to room temperature and stirred for 30 min. Dry triethylamine (1.43 mL, 11.0 mmol, 2.00 equiv) was added *via* syringe, followed by paraformaldehyde (611 mg, 20.3 mmol, 3.70 equiv). The reaction mixture was then heated to 65 °C for 18 h. After cooling to 0 °C, 1 M HCl (12 mL) was added and the mixture was extracted twith diethyl ether (3 x 20 mL). The combined organic extracts were washed with brine (40 mL), dried over anhydrous $MgSO_4$, filtered, and dried under reduced pressure to yield the crude formylated phenol which was used in the next step without further purification.

The crude aldehyde was dissolved in ethanol (27.5 mL, 0.2 M) and was transferred to a 100 mL round-bottom flask equipped with a magnetic stirring bar. (1*R*,2*R*)-1,2-diphenylethane-1,2-diamine (584 mg, 2.75 mmol, 0.50 equiv) was added and the solution was heated to reflux and stirred for 2 h. The solution was cooled to room temperature and the solvent was removed under reduced pressure. The crude residue was purified *via* flash column chromatography (hexane:ethyl acetate = 95:5) to yield salen ligand **10** (1.58 g, 2.33 mmol, 85% calculate based on diamine) as a yellow solid.

^{1}H NMR (400 MHz, $CDCl_3$): δ (ppm) = 13.48 (s, 2H), 8.27 (s, 2H), 7.25 – 7.15 (m, 10H), 6.92 (d, J = 2.8 Hz, 2H), 6.62 (d, J = 2.8 Hz, 2H), 4.71 (s, 2H), 1.40 (s, 18H), 1.22 (s, 18H).

^{13}C NMR (101 MHz, $CDCl_3$): δ (ppm) = 166.8, 156.7, 145.9, 139.6, 137.8, 128.5, 128.2, 127.7, 127.3, 124.6, 118.0, 80.4, 78.0, 34.9, 29.4, 28.8.

IR (thin film, cm^{-1}): 2974, 2872, 1627, 1592, 1436, 1365, 1315, 1258, 1171, 1148, 1036, 905, 855, 733, 697.

HRMS (ESI, *m/z*): calcd for $C_{44}H_{57}N_2O_4$ $[M+H]^+$: 677.4313, found: 677.4297.

m.p. (°C) = 88−91.

$[\alpha]_D^{24°}$ = -68.6 (c = 1.0, $CDCl_3$).

(*R*,*R*) Chiral catalyst (C2)

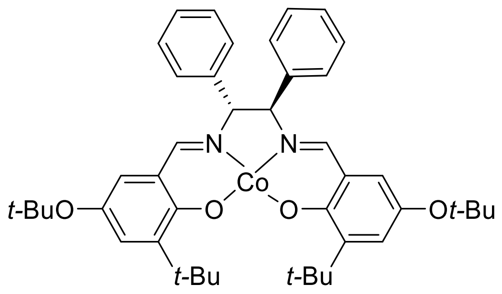

Cobalt-Salen complex **C2** was prepared according to **GP 1** using salen ligand **10** (677 mg, 1.00 mmol, 1.00 equiv), $Co(OAc)_2*4H_2O$ (249 mg, 1.00 mmol, 1.00 equiv) and ethanol (24 mL). The product was isolated after vacuum filtration and further drying as a dark brown/red solid (531 mg, 0.72 mmol, 72%).

IR (thin film, cm^{-1}): 2971, 2866, 1587, 1523, 1403, 1387, 1352, 1311, 1226, 1164, 1152, 959, 881, 859, 699.

HRMS (ESI, *m/z*): calcd for $C_{44}H_{54}CoN_2O_4$ [M]: 733.3410, found: 733.3405.

Chiral Cobalt Catalyst (C3)

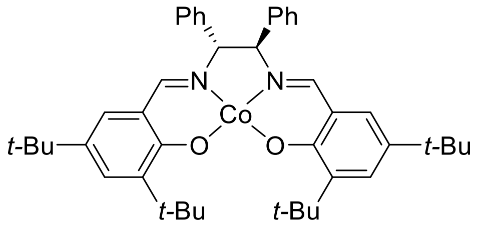

Chiral cobalt catalyst **C3** was prepared according to literature.[3]

Chiral Cobalt Catalyst (C4)

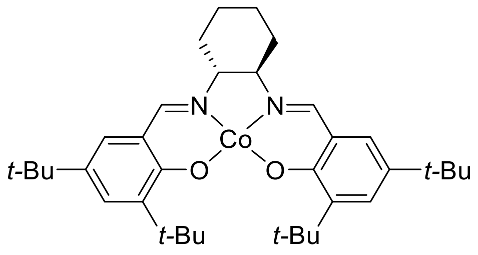

Chiral cobalt catalyst **C4** was prepared according to literature.[4]

Cobalt Catalyst (C5)

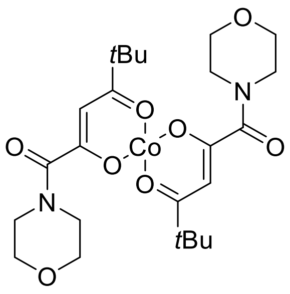

Cobalt Catalyst **C5** was prepared according to literature.[5]

Chiral Cobalt Catalyst (C6)

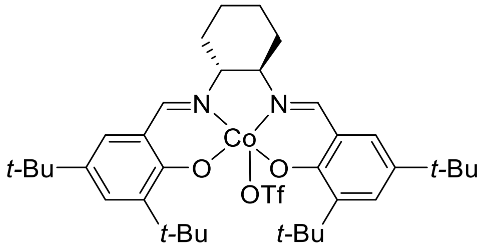

---

[3] Rajkumar T.; Manish K.; Tusharkumar M.; Naveen G.; Rukhsana K. I. K.; Noor-ul H. K..; Suresh E. *Advanced Synthesis and Catalysis*, **2017**, *359*, 3990–4001.
[4] Chapman J. J.; Day C. S.; Welker M. E. *Organometallics*, **2000**, *19*, 1615–1618.
[5] Kato K.; Yamada T.; Takai T.; Inoki S.; Isayama S. *Bulletin of the Chemical Society of Japan*, **1990**, *63*, 179–186.

Cobalt Catalyst **C6** was prepared according to literature.[6]

Cobalt Catalyst (C7)

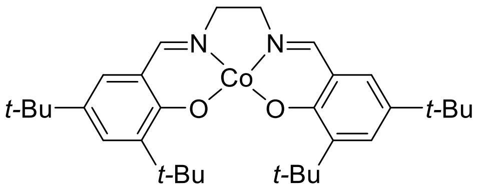

Cobalt Catalyst **C7** was prepared according to literature.[7]

Chiral Mangan Catalyst (C8)

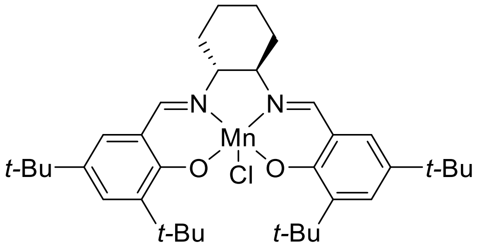

Cobalt Catalyst **C8** was prepared according to literature.[8]

Chiral Chromium Catalyst (C9)

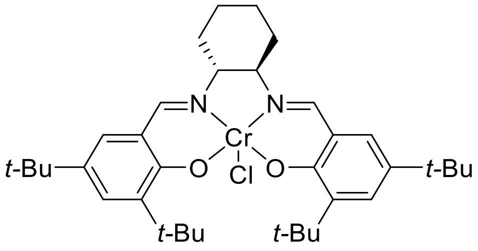

Chromium Catalyst **C9** was prepared according to literature.[9]

---

[6] White, D. E.; Tadross, P. M.; Lu, Z.; Jacobsen, E. N. *Tetrahedron*, **2014**, *70*, 4165–4180.
[7] Rhodes, B.; Rowling, S.; Tidswell, P.; Woodward, S.; Brown, S. M. *Journal of Molecular Catalysis A: Chemical*, **1997**, *116*, 375–384.
[8] Robert, A.; Tsapara, A.; Meunier, B. *Journal of Molecular Catalysis*, **1993**, *85*, 13–20.
[9] Li B.; Zhang R., Lu X.-B. *Macromolecules*, **2007**, *40*, 2303–2307.

5. Starting material synthesis (unsaturated *N*-acyl sulfonamides)

Starting materials **1a, 1c, 1d**, **1f**, and **1h-l** were prepared according to the work of Waser et al.[10]

Starting material **1b, 1e** and **1g** was prepared according to the work of Carreira et al.[11]

Starting material **1m, 1n** was prepared according to literature.[12]

[10] Nicolai, S.; Piemontesi, C.; Waser, J. *Angew. Chem. Int. Ed.* **2011**, *50*, 4680–4683.
[11] Fischer, D. M.; Balkenhohl, M.; Carreira, E. M. *J. Am. Chem.* **2022**, *2*, 1071–1077.
[12] Maurya, S. K.; Dow, M.; Warriner, S.; Nelson, A. *Beilstein J. Org. Chem.*, **2013**, *9*, 775–785.

6. Racemic aldehyde synthesis

5-oxo-1-tosylpyrrolidine-2-carbaldehyde (2a)

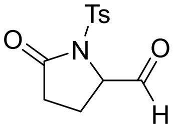

Aldehyde **2a** was prepared according to **GP 2** using unsaturated *N*-acyl sulfonamide **1a** (50.7 mg, 0.20 mmol, 1.00 equiv), catalyst **C1** (6.0 mg, 0.01 mmol, 0.05 equiv), and toluene (2.0 mL). The crude material was purified via flash column chromatography (hexane:ethyl acetate = 1:1) to give **2a** as brown-red oil.

Note: Aldehyde **2a** was prepared on larger scale by an adapted procedure based on **GP 2**, using *N*-acyl sulfonamide **1a** (253 mg, 1.00 mmol, 1.00 equiv), catalyst **C1** (30 mg, 0.05 mmol, 0.05 equiv), and toluene (10 mL) and the reaction was stirred for 3 h. The crude material was purified via flash column chromatography (hexane:ethyl acetate = 1:1) to give **2a** as brown-red oil in 52% (138 mg, 0.52 mmol).

Yield: 33.7 mg, 0.126 mmol, 63%

^{1}H NMR (400 MHz, $CDCl_3$): δ (ppm) = 9.74 (d, J = 1.5 Hz, 1H), 8.01 – 7.92 (m, 2H), 7.40 – 7.32 (m, 2H), 4.80 (ddd, J = 9.3, 4.2, 1.5 Hz, 1H), 2.45 (s, 3H), 2.44 – 2.41 (m, 2H), 2.31 (m, 1H), 2.21 – 2.09 (m, 1H)

^{13}C NMR (101 MHz, $CDCl_3$): δ (ppm) = 196.2, 172.6, 145.9, 134.8, 129.8, 129.1, 65.3, 30.5, 21.9, 19.7.

IR (thin film, cm^{-1}): 2925, 1728, 1597, 1402, 1350, 1222, 1186, 1087, 959, 882, 730, 649, 551.

HRMS (ESI, *m/z*): calcd for $C_{13}H_{17}NNaO_3S$ $[M+Na]^+$: 290.0457, found: 290.0458.

3-methyl-5-oxo-1-tosylpyrrolidine-2-carbaldehyde (2b)

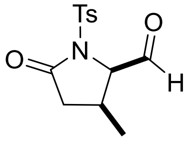

Aldehyde **2b** was prepared according to **GP 2** using unsaturated *N*-acyl sulfonamide **1b** (53.5 mg, 0.20 mmol, 1.00 equiv), catalyst **C1** (6.00 mg, 0.01 mmol, 0.05 equiv), and toluene (2.0 mL). The crude material was purified *via* flash column chromatography (hexane:ethyl acetate = 3:2) to give **2b** (both diastereomers collected separately) as an orange oil.

Note: Relative configuration was determined via 2D NOESY NMR analysis.

Yield: 33.8 mg, 0.120 mmol, 60%, d.r = 2:1.

^{1}H NMR (Major) (400 MHz, $CDCl_3$): δ (ppm) = 9.79 (d, J = 1.7 Hz, 1H), 7.94 – 7.89 (m, 2H), 7.38 – 7.31 (m, 2H), 4.85 (dd, J = 8.8, 1.7 Hz, 1H), 2.97 – 2.78 (m, 1H), 2.53 (dd, J = 17.1, 8.0 Hz, 1H), 2.45 (s, 3H), 2.18 (dd, J = 17.1, 11.0 Hz, 1H), 1.20 (d, J = 7.2 Hz, 3H).

^{13}C NMR (Major) (101 MHz, $CDCl_3$): δ (ppm) = 198.4, 172.0, 145.8, 134.8, 129.6, 129.1, 68.2, 39.0, 31.5, 21.9, 15.4.

^{1}H NMR (Minor) (400 MHz, $CDCl_3$): δ (ppm) = 9.70 (d, J = 2.2 Hz, 1H), 7.98 – 7.91 (m, 2H), 7.39 – 7.34 (m, 2H), 4.25 (dd, J = 5.2, 2.2 Hz, 1H), 2.63 (dd, J = 17.4, 8.3 Hz, 1H), 2.53 – 2.47 (m, 1H), 2.46 (s, 3H), 2.08 (dd, J = 17.4, 5.9 Hz, 1H), 1.19 (d, J = 6.8 Hz, 3H).

^{13}C NMR (Minor) (101 MHz, $CDCl_3$): δ (ppm) = 195.5, 172.2, 145.9, 134.7, 129.8, 128.9, 72.2, 38.9, 28.2, 21.9, 18.9.

IR (thin film, cm^{-1}): 2925, 2853, 1736, 1357, 1167, 667, 563.

HRMS (ESI, *m/z*): calcd for $C_{13}H_{16}NO_4S$ $[M+H]^+$: 282.0795, found: 282.0788.

2-Methyl-5-oxo-1-tosylpyrrolidine-2-carbaldehyde (2c)

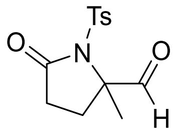

Aldehyde **2c** was prepared according to **GP 2** using unsaturated *N*-acyl sulfonamide **1c** (53.4 mg, 0.20 mmol, 1.00 equiv), catalyst **C1** (5.98 mg, 0.01 mmol, 0.05 equiv), and toluene (2.0 mL). The crude material was purified via flash column chromatography (hexane:ethyl acetate = 1:1) to give **2c** as orange oil.

Yield: 28.7 mg, 0.102 mmol, 51%.

^{1}H NMR (400 MHz, $CDCl_3$): δ (ppm) = 9.71 (s, 1H), 7.98 – 7.93 (m, 2H), 7.39 – 7.30 (m, 2H), 2.62 – 2.46 (m, 2H), 2.45 (s, 3H), 2.26 – 2.10 (m, 1H), 1.84 – 1.76 (m, 1H), 1.73 (s, 3H).

^{13}C NMR (101 MHz, $CDCl_3$): δ (ppm) = 196.1, 172.9, 145.8, 135.2, 129.7, 129.2, 72.1, 30.0, 28.0, 21.9, 20.2.

IR (thin film, cm^{-1}): 2934, 1740, 1355, 1238, 1167, 1086, 668, 560.

HRMS (ESI, *m/z*): calcd for $C_{13}H_{16}NO_4S$ $[M+H]^+$: 282.0795, found: 282.0795.

4,4-Dimethyl-5-oxo-1-tosylpyrrolidine-2-carbaldehyde (2d)

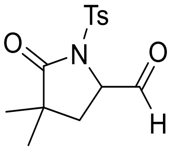

Aldehyde **2d** was prepared according to **GP 2** using unsaturated *N*-acyl sulfonamide **1d** (56.3 mg, 0.20 mmol, 1.00 equiv), catalyst **C1** (5.98 mg, 0.01 mmol, 0.05 equiv), and toluene (2.0 mL). The crude material was purified via flash column chromatography (hexane:ethyl acetate = 7:3) to give **2d** as light yellow oil.

Yield: 36.0 mg, 0.122 mmol, 61%

^{1}H NMR (400 MHz, $CDCl_3$): δ (ppm) = 9.73 (d, J = 2.3 Hz, 1H), 8.00 – 7.91 (m, 2H), 7.40 – 7.30 (m, 2H), 4.55 (ddd, J = 8.9, 6.7, 2.3 Hz, 1H), 2.45 (s, 3H), 2.13 (dd, J = 13.2, 8.9 Hz, 1H), 1.93 (dd, J = 13.2, 6.7 Hz, 1H), 1.11 (s, 3H), 1.01 (s, 3H).

^{13}C NMR (101 MHz, $CDCl_3$): δ (ppm) = 196.7, 177.9, 145.9, 134.5, 129.8, 128.8, 62.6, 41.8, 34.8, 24.7, 24.3, 21.9.

IR (thin film, cm^{-1}): 2972, 2932, 1738, 1597, 1458, 1232, 1189, 1171, 1089, 986, 704, 667, 546.

HRMS (ESI, *m/z*): calcd for $C_{14}H_{18}NO_4S$ $[M+H]^+$: 296.0951, found: 296.0945.

3,3-Dimethyl-5-oxo-1-tosylpyrrolidine-2-carbaldehyde (2e)

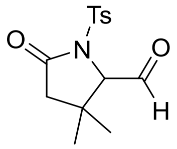

Aldehyde **2e** was prepared according to **GP 2** using unsaturated *N*-acyl sulfonamide **1e** (56.3 mg, 0.20 mmol, 1.00 equiv), catalyst **C1** (5.98 mg, 0.01 mmol, 0.05 equiv), and toluene (2.0 mL). The crude material was purified via flash column chromatography (hexane:ethyl acetate = 7:3) to give **2e** as light yellow oil.

Yield: 21.3 mg, 0.072 mmol, 36%

^{1}H NMR (400 MHz, $CDCl_3$): δ (ppm) = 9.76 (d, J = 2.3 Hz, 1H), 7.98 – 7.87 (m, 2H), 7.39 – 7.33 (m, 2H), 4.32 (d, J = 2.3 Hz, 1H), 2.45 (s, 3H), 2.36 (d, J = 16.9 Hz, 1H), 2.19 (d, J = 17.0 Hz, 1H), 1.23 (s, 3H), 1.20 (s, 3H)

^{13}C NMR (101 MHz, $CDCl_3$): δ (ppm) = 197.6, 171.8, 145.8, 134.7, 129.8, 128.9, 73.8, 46.1, 37.9, 28.7, 23.4, 21.9.

IR (thin film, cm^{-1}): 2964, 1735, 1597, 1494, 1452, 1396, 1356, 1237, 1167, 1149, 971, 705, 684, 550.

HRMS (ESI, *m/z*): calcd for $C_{14}H_{18}NO_4S$ $[M+H]^+$: 296.0951, found: 296.0945.

5-Oxo-4,4-diphenyl-1-tosylpyrrolidine-2-carbaldehyde (2f)

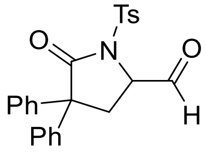

Aldehyde **2f** was prepared according to **GP 2** using unsaturated *N*-acyl sulfonamide **1f** (81.1 mg, 0.20 mmol, 1.00 equiv), catalyst **C1** (5.98 mg, 0.01 mmol, 0.05 equiv), and toluene (2.0 mL). The crude material was purified via flash column chromatography (hexane:ethyl acetate = 4:1) to give **2f** as light yellow oil.

Yield: 44.5 mg, 0.106 mmol, 53%.

^{1}H NMR (400 MHz, $CDCl_3$): δ (ppm) = 9.60 (d, J = 2.7 Hz, 1H), 7.94 – 7.87 (m, 2H), 7.33 – 7.26 (m, 4H), 7.25 – 7.13 (m, 6H), 7.02 – 6.97 (m, 2H), 4.44 (td, J = 7.3, 2.8 Hz, 1H), 3.04 (dd, J = 13.1, 7.4 Hz, 1H), 2.81 (dd, J = 13.2, 7.3 Hz, 1H), 2.47 (s, 3H).

^{13}C NMR (101 MHz, $CDCl_3$): δ (ppm) = 196.5, 173.7, 146.0, 140.3, 138.7, 134.0, 129.8, 128.9, 128.8, 128.8, 128.0, 127.8, 127.7, 127.4, 62.8, 58.2, 35.8, 21.9

IR (thin film, cm^{-1}): 3061, 2925, 1737, 1596, 1400, 1362, 1237, 1187, 1087, 1004, 784, 662, 544.

HRMS (ESI, *m/z*): calcd for $C_{24}H_{22}NO_4S$ $[M+H]^+$: 420.1270, found: 420.1272.

4-Oxo-5-tosyl-5-azaspiro[2.4]heptane-6-carbaldehyde (2g)

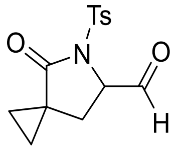

Aldehyde **2g** was prepared according to **GP 2** using unsaturated *N*-acyl sulfonamide **1g** (55.9 mg, 0.20 mmol, 1.00 equiv), catalyst **C1** (5.98 mg, 0.01 mmol, 0.05 equiv), and toluene (2.0 mL). The crude material was purified via flash column chromatography (hexane:ethyl acetate = 7:3) to give **2g** as light yellow oil.

Yield: 32.3 mg, 0.110 mmol, 55%

^{1}H NMR (400 MHz, $CDCl_3$): δ (ppm) = 9.77 (d, J = 1.8 Hz, 1H), 8.01 – 7.90 (m, 2H), 7.38 – 7.32 (m, 2H), 4.81 (ddd, J = 10.0, 4.4, 1.8 Hz, 1H), 2.50 – 2.40 (m, 4H), 2.10 (dd, J = 13.3, 4.4 Hz, 1H), 1.26 – 1.10 (m, 2H), 0.89 – 0.81 (m, 2H)

^{13}C NMR (101 MHz, $CDCl_3$): δ (ppm) = 196.6, 174.9, 145.7, 135.0, 129.7, 128.9, 63.2, 28.0, 22.4, 21.9, 16.8, 15.0.

IR (thin film, cm^{-1}): 3459, 2954, 2721, 1737, 1596, 1419, 1356, 1295, 1169, 1119, 957, 814, 704, 666, 577.

HRMS (ESI, *m/z*): calcd for $C_{14}H_{16}NO_4S$ $[M+H]^+$: 294.0795, found: 294.0796.

1-Oxo-2-tosyl-2-azaspiro[4.4]nonane-3-carbaldehyde (2h)

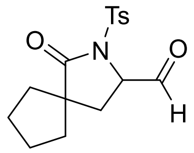

Aldehyde **2h** was prepared according to **GP 2** using unsaturated *N*-acyl sulfonamide **1h** (61.5 mg, 0.20 mmol, 1.00 equiv), catalyst **C1** (5.98 mg, 0.01 mmol, 0.05 equiv), and toluene (2.0 mL). The crude material was purified via flash column chromatography (hexane:ethyl acetate = 4:1) to give **2h** as light brown oil.

Yield: 29.6 mg, 0.092 mmol, 46%

^{1}H NMR (400 MHz, $CDCl_3$): δ (ppm) = 9.73 (d, J = 2.2 Hz, 1H), 7.99 – 7.94 (m, 2H), 7.38 – 7.33 (m, 2H), 4.57 (ddd, J = 8.8, 5.9, 2.2 Hz, 1H), 2.45 (s, 3H), 2.19 (dd, J = 13.1, 8.8 Hz, 1H), 2.02 (dd, J = 13.1, 5.9 Hz, 1H), 1.95 – 1.84 (m, 1H), 1.81 – 1.68 (m, 3H), 1.66 – 1.54 (m, 2H), 1.49 – 1.35 (m, 2H)

^{13}C NMR (101 MHz, $CDCl_3$): δ (ppm) = 196.9, 178.3, 145.8, 134.7, 129.8, 128.8, 63.4, 51.6, 37.3, 37.0, 35.0, 25.8, 25.6, 21.9.

IR (thin film, cm^{-1}): 2955, 1736, 1597, 1494, 1356, 1293, 1214, 1135, 1018, 932, 883, 664, 577.

HRMS (ESI, *m/z*): calcd for $C_{16}H_{20}NO_4S$ $[M+H]^+$: 322.1108, found: 322.1106.

1-Oxo-2-tosyl-2-azaspiro[4.5]decane-3-carbaldehyde (2i)

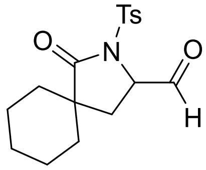

Aldehyde **2i** was prepared according to **GP 2** using unsaturated *N*-acyl sulfonamide **1i** (64.3 mg, 0.20 mmol, 1.00 equiv), catalyst **C1** (5.98 mg, 0.01 mmol, 0.05 equiv), and toluene (2.0 mL). The crude material was purified via flash column chromatography (hexane:ethyl acetate = 7:3) to give **2i** as brown oil.

Yield: 42.9 mg, 0.128 mmol, 64%

^{1}H NMR (400 MHz, $CDCl_3$): δ (ppm) = 9.72 (d, J = 2.4 Hz, 1H), 7.97 – 7.92 (m, 2H), 7.38 – 7.32 (m, 2H), 4.52 (ddd, J = 9.0, 6.5, 2.4 Hz, 1H), 2.45 (s, 3H), 2.21 (dd, J = 13.4, 9.1 Hz, 1H), 1.95 (dd, J = 13.4, 6.5 Hz, 1H), 1.71 – 1.53 (m, 5H), 1.43 (td, J = 11.1, 9.4, 3.1 Hz, 1H), 1.36 – 1.28 (m, 1H), 1.28 – 1.19 (m, 5H).

^{13}C NMR (101 MHz, $CDCl_3$): δ (ppm) = 197.0, 177.7, 145.9, 134.6, 129.8, 128.8, 62.8, 46.0, 33.0, 32.1, 31.1, 25.1, 21.9, 21.7, 21.6.

IR (thin film, cm^{-1}): 2933, 2859, 1737, 1597, 1525, 1450, 1234, 1063, 972, 918, 728, 635, 547.

HRMS (ESI, *m/z*): calcd for $C_{17}H_{22}NO_4S$ $[M+H]^+$: 336.1264, found: 336.1263.

3-Oxo-2-tosyloctahydro-1H-isoindole-1-carbaldehyde (2j)

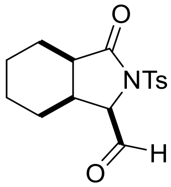

Aldehyde **2j** was prepared according to **GP 2** using unsaturated *N*-acyl sulfonamide **1j** (61.5 mg, 0.20 mmol, 1.00 equiv), catalyst **C1** (5.98 mg, 0.01 mmol, 0.05 equiv), and toluene (2.0 mL). The crude material was purified via flash column chromatography (hexane:ethyl acetate = 3:2) to give **2j** as light yellow oil.

Note: Relative configuration was determined via 2D NOESY NMR analysis.

Yield: 18.0 mg, 0.056 mmol, 28%, d.r = 20:1.

^{1}H NMR (400 MHz, $CDCl_3$): δ (ppm) = 9.78 (d, J = 3.5 Hz, 1H), 7.96 – 7.88 (m, 2H), 7.38 – 7.33 (m, 2H), 4.34 (dd, J = 6.2, 3.5 Hz, 1H), 2.72 (dtd, J = 11.7, 6.8, 5.5 Hz, 1H), 2.44 (s, 4H), 2.08 – 1.96 (m, 1H), 1.72 – 1.57 (m, 2H), 1.56 – 1.42 (m, 2H), 1.41 – 1.32 (m, 1H), 1.27 – 1.02 (m, 2H).

^{13}C NMR (101 MHz, $CDCl_3$): δ (ppm) = 198.1, 174.6, 145.8, 134.7, 130.0, 128.6, 67.7, 43.7, 36.4, 23.6, 23.4, 22.8, 22.2, 21.9.

IR (thin film, cm^{-1}): 2935, 1734, 1597, 1495, 1307, 1224, 1188, 1007, 989, 873, 840, 704, 668, 574.

HRMS (ESI, *m/z*): calcd for $C_{16}H_{20}NO_4S$ $[M+H]^+$: 322.1108, found: 322.1105.

6-Oxo-1-tosylpiperidine-2-carbaldehyde (2k)

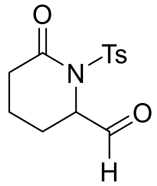

Aldehyde **2k** was prepared according to **GP 2** using unsaturated *N*-acyl sulfonamide **1k** (53.5 mg, 0.20 mmol, 1.00 equiv), catalyst **C1** (5.98 mg, 0.01 mmol, 0.05 equiv), and toluene (2.0 mL). The crude material was purified via flash column chromatography (hexane:ethyl acetate = 1:1) to give **2k** as light yellow oil.

Yield: 19.1 mg, 0.068 mmol, 34%

^{1}H NMR (400 MHz, $CDCl_3$): δ (ppm) = 9.67 (s, 1H), 7.96 – 7.90 (m, 2H), 7.36 – 7.29 (m, 2H), 5.14 (ddd, J = 6.7, 3.1, 0.9 Hz, 1H), 2.45 – 2.34 (m, 6H), 2.03 (dddd, J = 14.4, 12.4, 6.8, 3.9 Hz, 1H), 1.82 – 1.72 (m, 1H), 1.63 – 1.49 (m, 1H).

^{13}C NMR (101 MHz, $CDCl_3$): δ (ppm) = 197.2, 169.4, 145.3, 135.6, 129.9, 129.3, 64.1, 33.5, 23.1, 21.8, 17.2.

IR (thin film, cm^{-1}): 2955, 1735, 1695, 1596, 1495, 1346, 1261, 1188, 1158, 984, 845, 814, 674, 550.

HRMS (ESI, *m/z*): calcd for $C_{13}H_{16}NO_4S$ $[M+H]^+$: 282.0795, found: 282.0796.

1-Oxo-2-tosyl-1,2,3,4-tetrahydroisoquinoline-3-carbaldehyde (2l)

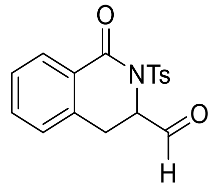

Aldehyde **2l** was prepared according to **GP 2** using unsaturated *N*-acyl sulfonamide **1l** (62.1 mg, 0.20 mmol, 1.00 equiv), catalyst **C1** (5.98 mg, 0.01 mmol, 0.05 equiv), and toluene (2.0 mL). The crude material was purified via flash column chromatography (hexane:ethyl acetate = 3:2) to give **2l** as light brown oil.

Yield: 32.9 mg, 0.100 mmol, 50%

^{1}H NMR (400 MHz, $CDCl_3$): δ (ppm) = 9.60 (s, 1H), 8.04 – 7.99 (m, 2H), 7.95 (dd, J = 7.8, 1.4 Hz, 1H), 7.45 (td, J = 7.5, 1.4 Hz, 1H), 7.38 – 7.27 (m, 3H), 7.20 (d, J = 7.6 Hz, 1H), 5.48 (dd, J = 5.2, 3.6 Hz, 1H), 3.56 – 3.50 (m, 2H), 2.44 (s, 3H).

^{13}C NMR (101 MHz, $CDCl_3$): δ (ppm) = 197.3, 162.4, 145.4, 135.8, 135.4, 134.0, 129.6, 129.5, 129.4, 128.2, 128.0, 127.7, 63.0, 28.7, 21.9.

IR (thin film, cm^{-1}): 2924, 1739, 1689, 1493, 1381, 1187, 1165, 1005, 957, 856, 793, 699, 567.

HRMS (ESI, *m/z*): calcd for $C_{17}H_{16}NO_4S$ $[M+H]^+$: 330.0795, found: 330.0787.

1-Oxo-2-tosyl-1,2,3,4-tetrahydropyrrolo[1,2-a]pyrazine-3-carbaldehyde (2m)

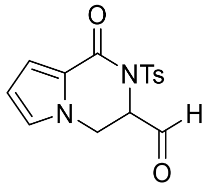

Aldehyde **2m** was prepared according to **GP 2** using unsaturated *N*-acyl sulfonamide **1m** (60.9 mg, 0.20 mmol, 1.00 equiv), catalyst **C1** (5.98 mg, 0.01 mmol, 0.05 equiv), and toluene (2.0 mL). The crude material was purified via flash column chromatography (hexane:ethyl acetate = 3:2) to give **2m** as light yellow oil.

Yield: 26.1 mg, 0.082 mmol, 40%

^{1}H NMR (400 MHz, $CDCl_3$): δ (ppm) = 9.59 (d, J = 1.3 Hz, 1H), 8.05 – 7.98 (m, 2H), 7.39 – 7.33 (m, 2H), 6.99 (dd, J = 4.1, 1.5 Hz, 1H), 6.78 (dd, J = 2.5, 1.5 Hz, 1H), 6.21 (dd, J = 4.1, 2.5 Hz, 1H), 5.42 (dd, J = 4.4, 1.9 Hz, 1H), 4.76 (dd, J = 13.1, 1.9 Hz, 1H), 4.39 (ddd, J = 13.1, 4.5, 1.3 Hz, 1H), 2.45 (s, 3H).

^{13}C NMR (101 MHz, $CDCl_3$): δ (ppm) = 196.5, 155.3, 145.7, 136.0, 129.7, 129.4, 125.9, 122.7, 118.1, 112.0, 63.2, 44.2, 21.9.

IR (thin film, cm^{-1}): 2924, 1752, 1730, 1596, 1482, 1340, 1293, 1246, 1069, 1011, 973, 704, 671, 568.

HRMS (ESI, *m/z*): calcd for $C_{15}H_{14}N_2NaO_4S$ $[M+Na]^+$: 341.0566, found: 341.0563.

1-((4-Methoxyphenyl)sulfonyl)-5-oxopyrrolidine-2-carbaldehyde (2n)

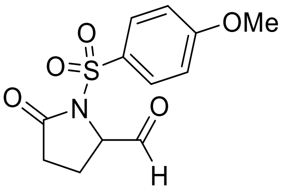

Aldehyde **2n** was prepared according to **GP 2** using unsaturated *N*-acyl sulfonamide **1n** (53.8 mg, 0.20 mmol, 1.00 equiv), catalyst **C1** (5.98 mg, 0.01 mmol, 0.05 equiv), and toluene (2.0 mL). The crude material was purified via flash column chromatography (hexane:ethyl acetate = 3:2) to give **2n** as light yellow oil.

Yield: 37.4 mg, 0.132 mmol, 66%.

^{1}H NMR (400 MHz, $CDCl_3$): δ (ppm) = 9.73 (d, J = 1.5 Hz, 1H), 8.05 – 7.98 (m, 2H), 7.04 – 6.99 (m, 2H), 4.80 (ddd, J = 9.3, 4.2, 1.5 Hz, 1H), 3.89 (s, 4H), 2.44 (m, 2H), 2.38 – 2.26 (m, 1H), 2.20 – 2.10 (m, 1H).

^{13}C NMR (101 MHz, $CDCl_3$): δ (ppm) = 196.3, 172.7, 164.6, 131.5, 129.0, 114.3, 65.4, 55.9, 30.5, 19.7.

IR (thin film, cm^{-1}): 2948, 1735, 1594, 1578, 1498, 1312, 1158, 954, 834, 730, 673, 563.

HRMS (ESI, *m/z*): calcd for $C_{12}H_{14}NO_5S$ $[M+H]^+$: 284.0587, found: 284.0583.

7. Enantioenriched alcohol synthesis

(*R*)-5-(hydroxymethyl)-1-tosylpyrrolidin-2-one (3a)

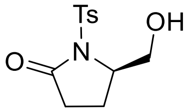

Alcohol **3a** was prepared according to **GP 3** using unsaturated *N*-acyl sulfonamide **1a** (50.7 mg, 0.20 mmol, 1.00 equiv), catalyst **C2** (29.3 mg, 0.04 mmol, 0.2 equiv), and toluene (2.0 mL). The reaction was stirred for 2 h. For the reduction, THF (2.0 mL), acetic acid (0.1 mL, 0.2 mmol, 1.0 equiv), and sodium triacetoxyborohydride (46.6 mg, 0.22 mmol, 1.10 equiv) were employed, and the mixture was stirred for 2 h. The crude material was purified by flash column chromatography (hexane:ethyl acetate = 2:3) to give **3a** (34 mg, 0.13 mmol, 63%) as an orange oil.

Yield: 33.9 mg, 0.126 mmol, 63%

^{1}H NMR (400 MHz, $CDCl_3$): δ (ppm) = 8.07 – 7.85 (m, 2H), 7.46 – 7.29 (m, 2H), 4.47 – 4.35 (m, 1H), 4.16 – 4.00 (m, 1H), 3.89 – 3.75 (m, 1H), 2.78 – 2.61 (m, 1H), 2.42 (s, 3H), 2.37 – 2.01 (m, 4H).

^{13}C NMR (101 MHz, $CDCl_3$): δ (ppm) = 174.5, 145.3, 135.7, 129.7, 128.4, 65.0, 61.2, 31.5, 22.6, 21.8.

IR (thin film, cm^{-1}): 3522, 2925, 1728, 1597, 1308, 1293, 1186, 1163, 1087, 959, 882, 730, 671, 533.

HRMS (ESI, *m/z*): calcd for $C_{12}H_{16}NO_4S$ $[M+H]^+$: 270.0795, found: 270.0791.

$[\alpha]_D^{24^\circ}$ = -14.8 (c = 0.3, $CDCl_3$).

Chiral SFC: Daicel Chiralpak AS-H, CO_2:^{i}PrOH = 96:4, 2 mL/min, λ = 232, t_R (major) = 12.8 min; t_R (minor) = 14.2 min, e.r. = 83:17.

(5*R*)-5-(hydroxymethyl)-4-methyl-1-tosylpyrrolidin-2-one (3b)

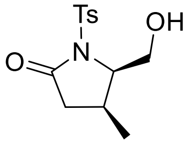

1.5:1 d.r.

Alcohol **3b** was prepared according to **GP 3** using unsaturated *N*-acyl sulfonamide **1b** (53.5 mg, 0.20 mmol, 1.00 equiv), catalyst **C2** (29.3 mg, 0.04 mmol, 0.2 equiv), and toluene (2.0 mL). The reaction mixture was stirred for 2 h. For the reduction, THF (2.0 mL), acetic acid (0.1 mL, 0.2 mmol, 1.0 equiv), and sodium triacetoxyborohydride (46.6 mg, 0.22 mmol, 1.10 equiv) were employed, and the mixture was stirred for 2 h. The crude material was purified by flash column chromatography (hexane:ethyl acetate = 3:2 to 1:1) to give **3b** (both diastereomers collected separately) as an orange oil.

Note: Major and minor diastereomer extrapolated from aldehyde 2D NOESY study

Yield: 32. mg, 0.116 mmol, 58%, d.r. = 1.5:1

^{1}H NMR (Major) (400 MHz, $CDCl_3$): δ (ppm) = 8.00 – 7.92 (m, 2H), 7.38 – 7.28 (m, 2H), 4.29 – 4.21 (m, 1H), 4.19 (dd, J = 12.3, 2.9 Hz, 1H), 3.88 (dd, J = 12.3, 1.8 Hz, 1H), 2.66 – 2.54 (m, 1H), 2.54 – 2.45 (m, 1H), 2.43 (s, 3H), 2.32 (dd, J = 16.3, 8.1 Hz, 1H), 1.87 (br s, 1H), 1.19 (d, J = 6.6 Hz, 3H).

^{13}C NMR (Major) (101 MHz, $CDCl_3$): δ (ppm) = 174.2, 145.2, 135.9, 129.7, 128.4, 64.3, 61.6, 39.8, 31.6, 21.8, 14.5.

^{1}H NMR (Minor) (400 MHz, $CDCl_3$): δ (ppm) = 7.99 – 7.85 (m, 2H), 7.41 – 7.31 (m, 2H), 4.04 (dd, J = 11.6, 4.1 Hz, 1H), 3.93 – 3.89 (m, 1H), 3.84 (dd, J = 11.7, 3.1 Hz, 1H), 2.87 (dd, J = 17.5, 8.3 Hz, 1H), 2.43 (s, 3H), 2.41 – 2.34 (m, 1H), 2.27 (br s, 1H), 1.90 (dd, J = 17.5, 2.1 Hz, 1H), 1.00 (d, J = 7.1 Hz, 3H).

^{13}C NMR (Minor) (101 MHz, $CDCl_3$): δ (ppm) = 174.0, 145.4, 135.5, 129.8, 128.4, 68.7, 64.6, 39.5, 30.0, 21.8, 20.8.

IR (thin film, cm^{-1}): 3536, 2928, 1728, 1597, 1350, 1331, 1167, 1157, 1087, 679, 558, 550.

HRMS (ESI, *m/z*): calcd for $C_{13}H_{17}NNaO_4S$ $[M+Na]^+$: 306.0770, found: 306.0771.

$[\alpha]_D^{24°}$ (Major) = -2.8 (c = 0.5, $CDCl_3$).

Chiral SFC (Major): Daicel Chiralpak AS-H, CO_2:^{i}PrOH = 95:5, 2 mL/min, λ = 228, t_R (major) = 8.7 min; t_R (minor) = 10.8 min, e.r. = 62:38.

(*R*)-5-(hydroxymethyl)-5-methyl-1-tosylpyrrolidin-2-one (3c)

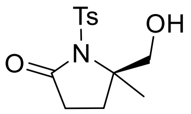

Alcohol **3c** was prepared according to **GP 3** using unsaturated *N*-acyl sulfonamide **1c** (26.7 mg, 0.10 mmol, 1.00 equiv), catalyst **C2** (14.7 mg, 0.02 mmol, 0.2 equiv), and toluene (1.0 mL). The reaction mixture was stirred for 2 h. For the reduction, THF (1.0 mL), acetic acid (0.05 mL, 0.10 mmol, 1.00 equiv), and sodium triacetoxyborohydride (23.3 mg, 0.11 mmol, 1.10 equiv) were employed, and the mixture was stirred for 2 h. The crude material was purified by flash column chromatography (hexane:ethyl acetate = 2:3) to give **3c** as an orange oil.

Yield: 17.0 mg, 0.060 mmol, 60%

^{1}H NMR (400 MHz, $CDCl_3$): δ (ppm) = 8.02 – 7.93 (m, 2H), 7.37 – 7.29 (m, 2H), 4.23 (d, J = 11.8 Hz, 1H), 3.65 (d, J = 11.9 Hz, 1H), 2.63 – 2.49 (m, 1H), 2.43 (s, 3H), 2.38 – 2.22 (m, 2H), 1.91 – 1.75 (m, 1H), 1.59 (s, 3H).

^{13}C NMR (101 MHz, $CDCl_3$): δ (ppm) = 175.2, 145.1, 136.2, 129.5, 128.8, 70.6, 68.8, 31.5, 30.3, 23.7, 21.8.

IR (thin film, cm^{-1}): 3538, 2938, 1733, 1597, 1350, 1170, 1088, 948, 815, 675, 563.

HRMS (ESI, *m/z*): calcd for $C_{13}H_{17}NNaO_4S$ $[M+Na]^+$: 306.0770, found: 306.0770.

$[\alpha]_D^{24°}$ = +3.7 (c = 0.1, $CDCl_3$).

Chiral HPLC: Daicel Chiralpak AM-R, H_2O:ACN +0.1% FA = 7:3, 1 mL/min, λ = 232, t_R (major) = 9.9 min; t_R (minor) = 19.5 min, e.r. = 60:40.

(*R*)-5-(hydroxymethyl)-3,3-dimethyl-1-tosylpyrrolidin-2-one (3d)

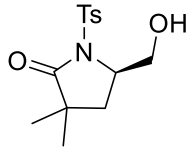

Alcohol **3d** was prepared according to **GP 3** using unsaturated *N*-acyl sulfonamide **1d** (56.3 mg, 0.20 mmol, 1.00 equiv), catalyst **C2** (29.3 mg, 0.04 mmol, 0.2 equiv), and toluene (2.0 mL). The reaction mixture was stirred for 2 h. For the reduction, THF (2.0 mL), acetic acid (0.1 mL, 0.2 mmol, 1.0 equiv), and sodium triacetoxyborohydride (46.6 mg, 0.22 mmol, 1.10 equiv) were employed, and the mixture was stirred for 2 h. The crude material was purified by flash column chromatography (hexane:ethyl acetate = 3:2) to give **3d** as an orange oil.

Yield: 36.9 mg, 0.124 mmol, 62%

^{1}H NMR (400 MHz, $CDCl_3$): δ (ppm) = 7.97 – 7.88 (m, 2H), 7.36 – 7.30 (m, 2H), 4.23 (ddt, J = 8.2, 7.2, 3.6 Hz, 1H), 4.06 (dd, J = 12.1, 3.6 Hz, 1H), 3.83 (dd, J = 12.1, 3.7 Hz, 1H), 2.44 (s, 3H), 2.02 (dd, J = 13.1, 8.2 Hz, 1H), 1.88 (dd, J = 13.1, 7.1 Hz, 1H), 1.17 (s, 3H), 0.96 (s, 3H).

^{13}C NMR (101 MHz, $CDCl_3$): δ (ppm) = 179.5, 145.5, 135.1, 129.8, 128.3, 65.0, 58.9, 41.0, 36.8, 25.5, 24.7, 21.8.

IR (thin film, cm^{-1}): 3534, 2968, 1733, 1597, 1352, 1167, 1108, 1088, 664, 580, 546.

HRMS (ESI, *m/z*): calcd for $C_{14}H_{20}NO_4S$ $[M+H]^+$: 298.1108, found: 298.1104.

$[\alpha]_D^{23^\circ}$ = -4.4 (c = 1.0, $CDCl_3$).

Chiral SFC: Daicel Chiralpak OB-H, CO_2:^{i}PrOH = 98.6:1.4, 2 mL/min, λ = 229, t_R (major) = 27.0 min; t_R (minor) = 23.7 min, e.r. = 76:24.

(*R*)-5-(hydroxymethyl)-4,4-dimethyl-1-tosylpyrrolidin-2-one (3e)

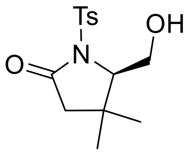

Alcohol **3e** was prepared according to a modification of **GP 3** using unsaturated *N*-acyl sulfonamide **1e** (56.3 mg, 0.20 mmol, 1.00 equiv), catalyst **C2** (29.3 mg, 0.04 mmol, 0.2 equiv), and toluene (2.0 mL). The cyclization step was run for 5 h, the solvent was removed under reduced pressure, and the aldehyde/alcohol mixture was isolated *via* flash column chromatography (hexane:ethyl acetate = 3:2). The mixture was then redissolved in dry THF (2.0 mL), and acetic acid (0.2 mL, 0.2 mmol, 2.0 equiv) and sodium triacetoxyborohydride (84.8 mg, 0.22 mmol, 2.00 equiv) were employed. The solution was stirred for 2 h and after the noted workup, the crude material was purified by flash column chromatography (hexane:ethyl acetate = 65:35) to give **3e** as an orange oil.

Yield: 29.1 mg, 0.098 mmol, 49%

^{1}H NMR (400 MHz, $CDCl_3$): δ (ppm) = 7.99 – 7.92 (m, 2H), 7.40 – 7.31 (m, 2H), 4.19 (ddd, J = 12.4, 5.2, 3.1 Hz, 1H), 3.91 (ddd, J = 12.3, 6.3, 2.2 Hz, 1H), 3.81 (t, J = 2.6 Hz, 1H), 2.70 (dd, J = 16.8, 0.8 Hz, 1H), 2.44 (s, 3H), 1.96 (d, J = 16.8 Hz, 1H), 1.21 (s, 3H), 0.99 (s, 3H).

^{13}C NMR (101 MHz, $CDCl_3$): δ (ppm) = 173.9, 145.3, 135.4, 129.7, 128.4, 70.5, 62.4, 46.3, 36.6, 29.6, 22.6, 21.8.

IR (thin film, cm^{-1}): 3535, 2958, 2932, 2879, 1741, 1597, 1353, 1165, 1089, 671, 564.

HRMS (ESI, *m/z*): calcd for $C_{14}H_{20}NO_4S$ $[M+H]^+$: 298.1108, found: 298.1099.

$[\alpha]_D^{24^\circ}$ = +7.1 (c = 0.5, $CDCl_3$).

Chiral SFC: Daicel Chiralpak AS-H, CO_2:^{i}PrOH = 95:5, 2 mL/min, λ = 228, t_R (major) = 10.4 min; t_R (minor) = 6.5 min, e.r. = 60:40.

(*R*)-5-(hydroxymethyl)-3,3-diphenyl-1-tosylpyrrolidin-2-one (3f)

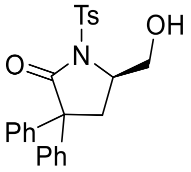

Alcohol **3f** was prepared according to **GP 3** using unsaturated *N*-acyl sulfonamide **1f** (81.1 mg, 0.20 mmol, 1.00 equiv), catalyst **C2** (29.3 mg, 0.04 mmol, 0.2 equiv), and toluene (2.0 mL). The reaction mixture was stirred for 2 h. For the reduction, THF (2.0 mL), acetic acid (0.1 mL, 0.2 mmol, 1.0 equiv), and sodium triacetoxyborohydride (46.6 mg, 0.22 mmol, 1.10 equiv) were employed, and the mixture was stirred for 2 h. The crude material was purified by flash column chromatography (hexane:ethyl acetate = 7:3) to give **3f** as a light-brown solid.

Yield: 60.7 mg, 0.144 mmol, 72%

^{1}H NMR (400 MHz, $CDCl_3$): δ (ppm) = 7.82 – 7.74 (m, 2H), 7.27 – 7.08 (m, 10H), 7.03 – 6.94 (m, 2H), 4.22 – 4.12 (m, 1H), 4.01 (dd, J = 12.2, 3.9 Hz, 1H), 3.91 (dd, J = 12.2, 3.9 Hz, 1H), 2.93 (dd, J = 13.4, 6.6 Hz, 1H), 2.74 (dd, J = 13.4, 8.6 Hz, 1H), 2.43 (s, 3H).

^{13}C NMR (101 MHz, $CDCl_3$): δ (ppm) = 174.9, 145.4, 141.9, 139.3, 134.3, 129.6, 128.8, 128.5, 128.5, 127.9, 127.5, 127.5, 127.4, 64.5, 59.6, 57.8, 36.9, 21.8.

IR (thin film, cm^{-1}): 3539, 2924, 1732, 1597, 1358, 1171, 1088, 700, 663, 573.

HRMS (ESI, *m/z*): calcd for $C_{24}H_{24}NO_4S$ $[M+H]^+$: 422.1421, found: 422.1416.

m.p. (°C) = 62−65.

$[\alpha]_D^{25°}$ = +11.0 (c = 1.0, $CDCl_3$).

Chiral SFC: Daicel Chiralpak OB-H, CO_2:^{i}PrOH = 91:9, 2 mL/min, λ = 200, t_R (major) = 16.6 min; t_R (minor) = 19.3 min, e.r. = 58:42.

(*R*)-6-(hydroxymethyl)-5-tosyl-5-azaspiro[2.4]heptan-4-one (3g)

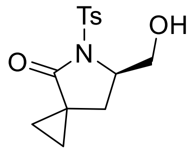

Alcohol **3g** was prepared according to **GP 3** using unsaturated *N*-acyl sulfonamide **1g** (55.9 mg, 0.20 mmol, 1.00 equiv), catalyst **C2** (29.3 mg, 0.04 mmol, 0.2 equiv), and toluene (2.0 mL). The reaction mixture was stirred for 5 h. For the reduction, THF (2.0 mL), acetic acid (0.1 mL, 0.2 mmol, 1.0 equiv), and sodium triacetoxyborohydride (46.6 mg, 0.22 mmol, 1.10 equiv) were employed, and the mixture was stirred for 5h. The crude material was purified by flash column chromatography (hexane:ethyl acetate = 1:1) to give **3g** as an orange oil.

Yield: 50.2 mg, 0.170 mmol, 86%

^{1}H NMR (400 MHz, $CDCl_3$): δ (ppm) = 8.01 – 7.91 (m, 2H), 7.40 – 7.30 (m, 2H), 4.54 – 4.45 (m, 1H), 4.02 (dd, J = 11.6, 4.7 Hz, 1H), 3.86 (dd, J = 11.6, 3.6 Hz, 1H), 2.52 – 2.39 (m, 4H), 1.94 (dd, J = 12.9, 2.4 Hz, 1H), 1.21 (ddd, J = 10.6, 6.9, 3.9 Hz, 1H), 1.13 (ddd, J = 10.2, 6.8, 3.7 Hz, 1H), 0.90 (ddd, J = 9.4, 6.9, 3.8 Hz, 1H), 0.79 (ddd, J = 9.1, 6.9, 3.9 Hz, 1H).

^{13}C NMR (101 MHz, $CDCl_3$): δ (ppm) = 176.2, 145.2, 135.8, 129.7, 128.5, 65.0, 59.0, 30.6, 22.8, 21.8, 18.1, 14.7.

IR (thin film, cm^{-1}): 3531, 2954, 1733, 1357, 1169, 1109, 1090, 666, 580, 547.

HRMS (ESI, *m/z*): calcd for $C_{14}H_{17}NNaO_4S$ $[M+Na]^+$: 318.0770, found: 318.0762.

$[\alpha]_D^{25^{\circ}}$ = +2.9 (c = 1.0, $CDCl_3$).

Chiral HPLC: Daicel Chiralpak AM-R, H_2O:ACN +0.1% FA = 75:25, 1 mL/min, λ = 233, t_R (major) = 15.1 min; t_R (minor) = 24.3 min, e.r. = 57:43.

(*R*)-3-(hydroxymethyl)-2-tosyl-2-azaspiro[4.4]nonan-1-one (3h)

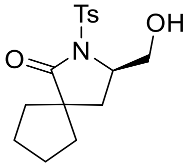

3h

Alcohol **3h** was prepared according to **GP 3** using unsaturated *N*-acyl sulfonamide **1h** (61.5 mg, 0.20 mmol, 1.00 equiv), catalyst **C2** (29.3 mg, 0.04 mmol, 0.2 equiv), and toluene (2.0 mL). The reaction was run for 2 h. For the reduction, THF (2.0 mL), acetic acid (0.1 mL, 0.2 mmol, 1.0 equiv), and sodium triacetoxyborohydride (46.6 mg, 0.22 mmol, 1.10 equiv) were employed, and the mixture was stirred for 2 h. The crude material was purified by flash column chromatography (hexane:ethyl acetate = 3:2) to give **3h** as an orange oil.

Yield: 53.7 mg, 0.166 mmol, 83%

^{1}H NMR (400 MHz, $CDCl_3$): δ (ppm) = 7.97 – 7.88 (m, 2H), 7.39 – 7.29 (m, 2H), 4.30 – 4.20 (m, 1H), 3.99 (dd, J = 11.9, 4.2 Hz, 1H), 3.88 (dd, J = 11.9, 4.1 Hz, 1H), 2.44 (s, 3H), 2.09 (dd, J = 13.1, 8.2 Hz, 1H), 2.01 – 1.90 (m, 2H), 1.79 – 1.52 (m, 6H), 1.47 – 1.37 (m, 1H).

^{13}C NMR (101 MHz, $CDCl_3$) δ (ppm) = 179.8, 145.3, 135.4, 129.8, 128.3, 65.0, 59.5, 51.2, 38.5, 37.5, 37.1, 25.7, 25.5, 21.8.

IR (thin film, cm^{-1}): 3531, 2955, 2871, 1731, 1355, 1169, 666, 579, 548.

HRMS (ESI, *m/z*): calcd for $C_{16}H_{22}NO_4S$ $[M+H]^+$: 324.1264, found: 324.1260.

$[\alpha]_D^{24^\circ}$ = -3.1 (c = 1.0, $CDCl_3$).

Chiral SFC: Daicel Chiralpak OB-H, CO_2:^{i}PrOH = 95:5, 2 mL/min, λ = 229, t_R (major) = 20.9 min; t_R (minor) = 15.7 min, e.r. = 73:27.

(*R*)-3-(hydroxymethyl)-2-tosyl-2-azaspiro[4.5]decan-1-one (3i)

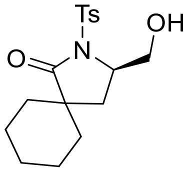

Alcohol **3i** was prepared according to **GP 3** using unsaturated *N*-acyl sulfonamide **1i** (64.3 mg, 0.20 mmol, 1.00 equiv), catalyst **C2** (29.3 mg, 0.04 mmol, 0.2 equiv), and toluene (2.0 mL). The reaction mixture was stirred for 5 h. For the reduction, THF (2.0 mL), acetic acid (0.1 mL, 0.2 mmol, 1.0 equiv), and sodium triacetoxyborohydride (46.6 mg, 0.22 mmol, 1.10 equiv) were employed, and the mixture was stirred for 2 h. The crude material was purified by flash column chromatography (hexane:ethyl acetate = 7:3) to give **3i** as an orange oil.

Yield: 54.0 mg, 0.160 mmol, 80%

^{1}H NMR (400 MHz, $CDCl_3$): δ (ppm) = 7.97 – 7.87 (m, 2H), 7.39 – 7.28 (m, 2H), 4.27 – 4.19 (m, 1H), 4.05 (dd, J = 12.0, 3.7 Hz, 1H), 3.82 (dd, J = 12.0, 3.6 Hz, 1H), 2.43 (s, 3H), 2.14 (dd, J = 13.2, 8.6 Hz, 1H), 1.84 (ddd, J = 13.3, 6.5, 0.9 Hz, 1H), 1.75 – 1.44 (m, 5H), 1.39 – 1.13 (m, 5H).

^{13}C NMR (101 MHz, $CDCl_3$): δ (ppm) = 179.2, 145.4, 135.3, 129.7, 128.3, 65.2, 59.0, 45.4, 33.2, 33.1, 32.9, 25.2, 21.9, 21.8, 21.7.

IR (thin film, cm^{-1}): 3527, 2931, 2858, 1729, 1354, 1167, 664, 578, 547.

HRMS (ESI, *m/z*): calcd for $C_{17}H_{24}NO_4S$ $[M+H]^+$: 338.1421, found: 338.1422.

$[\alpha]_D^{23^\circ}$ = -7.4 (c = 1.0, $CDCl_3$).

Chiral SFC: Daicel Chiralpak AS-H, CO_2:^{i}PrOH = 97:3, 2 mL/min, λ = 229, t_R (major) = 24.8 min; t_R (minor) = 27.4 min, e.r. = 75:25.

(*R*)-5-(hydroxymethyl)-1-((4-methoxyphenyl)sulfonyl)pyrrolidin-2-one (3j)

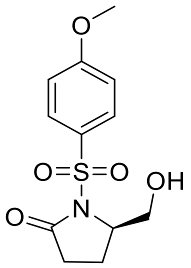

Alcohol **3j** was prepared according to **GP 3** using unsaturated *N*-acyl sulfonamide **1n** (53.9 mg, 0.20 mmol, 1.00 equiv), catalyst **C2** (29.3 mg, 0.04 mmol, 0.2 equiv), and toluene (2.0 mL). The reaction mixture was stirred for 2 h. For the reduction, THF (2.0 mL), acetic acid (0.1 mL, 0.2 mmol, 1.0 equiv), and sodium triacetoxyborohydride (46.6 mg, 0.22 mmol, 1.10 equiv) were employed, and the mixture was stirred for 2 h. The crude material was purified by flash column chromatography (hexane:ethyl acetate = 2:3) to give **3j** as an orange oil.

Yield: 34.2 mg, 0.120 mmol, 60%

^{1}H NMR (400 MHz, $CDCl_3$): δ (ppm) = 8.08 – 7.97 (m, 2H), 7.06 – 6.91 (m, 2H), 4.47 – 4.35 (m, 1H), 4.08 (dq, J = 13.5, 4.5 Hz, 1H), 3.87 (s, 3H), 3.81 (dq, J = 11.7, 2.9 Hz, 1H), 2.68 (dt, J = 16.9, 9.9 Hz, 1H), 2.37 – 1.97 (m, 4H).

^{13}C NMR (101 MHz, $CDCl_3$): δ (ppm) = 174.5, 164.1, 130.8, 130.0, 114.3, 65.1, 61.1, 55.8, 31.5, 22.6.

IR (thin film, cm^{-1}): 3530, 2949, 1728, 1595, 1498, 1352, 1254, 1160, 1091, 678, 558.

HRMS (ESI, *m/z*): calcd for $C_{12}H_{16}NO_5S$ $[M+H]^+$: 286.0744, found: 286.0744.

$[\alpha]_D^{23^\circ}$ = -18.7 (c = 1.0, $CDCl_3$).

Chiral HPLC: Daicel Chiralpak AM-R, H_2O:ACN +0.1% FA = 77:23, 1 mL/min, λ = 247, t_R (major) = 9.8 min; t_R (minor) = 12.3 min, e.r. = 83:17.

(3*R*)-3-(hydroxymethyl)-2-tosyloctahydro-1H-isoindol-1-one (3k)

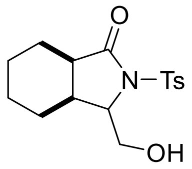

1.9:1 d.r.

Alcohol **3k** was prepared according to a modification of **GP 3** using unsaturated *N*-acyl sulfonamide **1k** (56.3 mg, 0.20 mmol, 1.00 equiv), catalyst **C2** (29.3 mg, 0.04 mmol, 0.2 equiv), and toluene (2.0 mL). The cyclization step was run for 3 h, the solvent was removed under reduced pressure, and the aldehyde/alcohol mixture was isolated *via* flash column chromatography (hexane:ethyl acetate = 3:2). The mixture was then redissolved in dry THF (2.0 mL), and acetic acid (0.3 mL, 0.3 mmol, 3.0 equiv) and sodium triacetoxyborohydride (127 mg, 0.30 mmol, 3.00 equiv) were employed. The solution was stirred for 4 h and after the noted workup, the crude material was purified by flash column chromatography (hexane:ethyl acetate = 3:2) to give **3k** as an orange oil.

Yield: 44.0 mg, 0.136 mmol, 68%

Note: the diastereomers were co-polar and could not be separated

^{1}H NMR (400 MHz, $CDCl_3$): δ (ppm) = 8.00 – 7.85 (m, 2H), 7.46 – 7.29 (m, 2H), 4.22 – 3.75 (m, 3H), 3.45 – 2.87 (m, 1H), 2.51 – 2.29 (m, 5H), 2.10 – 1.95 (m, 1H), 1.86 – 1.60 (m, 2H), 1.57 – 1.30 (m, 2H), 1.17 – 0.65 (m, 3H).

Note: Peaks of the diastereomeric mixture are given

^{13}C NMR (Major) (101 MHz, $CDCl_3$): δ (ppm) = 175.9, 145.4, 135.3, 129.8, 128.2, 67.0, 62.7, 43.5, 36.7, 23.5, 22.5, 22.5, 22.3, 21.8.

^{13}C NMR (Minor) (101 MHz, $CDCl_3$): δ (ppm) = 175.2, 145.3, 135.4, 129.7, 128.4, 66.1, 63.9, 41.2, 36.3, 29.1, 23.7, 22.6, 22.5, 21.9.

IR (thin film, cm^{-1}): 3535, 2931, 2857, 1738, 1597, 1354, 1164, 1089, 667, 579.

HRMS (ESI, *m/z*): calcd for $C_{16}H_{21}NNaO_4S$ $[M+Na]^+$: 346.1084, found: 346.1086.

$[\alpha]_D^{25^\circ}$ = 0.3 (c = 1.0, $CDCl_3$).

Chiral HPLC: Daicel Chiralpak AD-H, hexane:^{i}PrOH = 92:8, 1 mL/min, λ = 229, t_R (major) = 39.0 min; t_R (minor) = 44.2 min, e.r. = 51:49.

(*R*)-3-(hydroxymethyl)-2-tosyl-3,4-dihydroisoquinolin-1(2H)-one (3l)

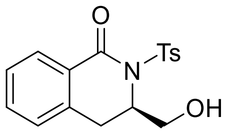

Alcohol **3l** was prepared according to **GP 3** using unsaturated *N*-acyl sulfonamide **1l** (63.1 mg, 0.20 mmol, 1.00 equiv), catalyst **C2** (29.3 mg, 0.04 mmol, 0.2 equiv), and toluene (2.0 mL). The reaction mixture was stirred for 2 h. For the reduction, THF (2.0 mL), acetic acid (0.1 mL, 0.2 mmol, 1.0 equiv), and sodium triacetoxyborohydride (46.6 mg, 0.22 mmol, 1.10 equiv) were employed, and the mixture was stirred for 2 h. The crude material was purified by flash column chromatography (hexane:ethyl acetate = 3:2) to give **3l** as a light-brown solid.

Yield: 47.7 mg, 0.144 mmol, 72%

^{1}H NMR (400 MHz, $CDCl_3$): δ (ppm) = 8.06 – 7.99 (m, 2H), 7.94 (ddd, J = 7.8, 1.4, 0.5 Hz, 1H), 7.48 (td, J = 7.5, 1.4 Hz, 1H), 7.37 – 7.28 (m, 3H), 7.24 – 7.17 (m, 1H), 5.06 – 4.96 (m, 1H), 3.81 (dd, J = 11.0, 6.2 Hz, 1H), 3.57 (t, J = 9.6 Hz, 1H), 3.37 (dd, J = 16.5, 5.9 Hz, 1H), 3.20 (dd, J = 16.6, 1.8 Hz, 1H), 2.42 (s, 3H), 1.94 (br s, 1H).

^{13}C NMR (101 MHz, $CDCl_3$): δ (ppm) = 162.8, 145.0, 136.7, 136.5, 134.0, 129.5, 129.2, 129.0, 128.3, 128.0, 127.6, 63.2, 56.2, 29.8, 21.8.

IR (thin film, cm^{-1}): 3524, 2925, 1686, 1603, 1349, 1166, 1062, 738, 667, 559.

HRMS (ESI, *m/z*): calcd for $C_{17}H_{18}NO_4S$ $[M+H]^+$: 332.0951, found: 332.0946.

m.p. (°C) = 54−57.

$[\alpha]_D^{25^\circ}$ = +10.4 (c = 1.0, $CDCl_3$).

Chiral SFC: Daicel Chiralpak OB-H, CO_2:^{i}PrOH = 91:9, 2 mL/min, λ = 200, t_R (major) = 18.2 min; t_R (minor) = 11.2 min, e.r. = 61:39.

(*R*)-3-(Hydroxymethyl)-2-tosyl-3,4-dihydropyrrolo[1,2-a]pyrazin-1(2H)-one (3m)

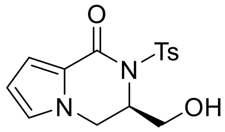

Alcohol **3m** was prepared according to **GP 3** using unsaturated *N*-acyl sulfonamide **1m** (60.9 mg, 0.20 mmol, 1.00 equiv), catalyst **C2** (29.3 mg, 0.04 mmol, 0.2 equiv), and toluene (2.0 mL). The reaction mixture was stirred for 7.5 h. For the reduction, THF (2.0 mL), acetic acid (0.1 mL, 0.2 mmol, 1.0 equiv), and sodium triacetoxyborohydride (46.6 mg, 0.22 mmol, 1.10 equiv) were employed, and the mixture was stirred for 2 h. The crude material was purified by flash column chromatography (hexane:ethyl acetate = 1:1) to give **3m** as an orange oil.

Note: The starting material was not completely consumed, but there was no notable difference between 5.5 and 7.5 h reaction time.

Yield: 32.7 mg, 0.102 mmol, 51%

^{1}H NMR (400 MHz, $CDCl_3$): δ (ppm) = 8.06 – 7.91 (m, 2H), 7.31 (d, J = 8.1 Hz, 2H), 7.01 – 6.89 (m, 1H), 6.84 – 6.71 (m, 1H), 6.28 – 6.19 (m, 1H), 4.97 (dddd, J = 9.6, 5.7, 4.1, 1.5 Hz, 1H), 4.49 (dd, J = 13.2, 1.5 Hz, 1H), 4.26 (dd, J = 13.2, 4.2 Hz, 1H), 3.84 (dd, J = 10.8, 5.6 Hz, 1H), 3.51 (t, J = 10.1 Hz, 1H), 2.41 (s, 3H), 2.37 (s, 1H).

^{13}C NMR (101 MHz, $CDCl_3$): δ (ppm) = 156.1, 145.1, 136.4, 129.5, 129.1, 125.7, 122.3, 117.1, 111.3, 61.3, 56.4, 44.3, 21.8.

IR (thin film, cm^{-1}): 3436, 2926, 1677, 1533, 1486, 1401, 1348, 1166, 1059, 814, 560.

HRMS (ESI, *m/z*): calcd for $C_{15}H_{17}N_2O_4S$ $[M+H]^+$: 321.0904, found: 321.0906.

$[\alpha]_D^{25°}$ = +1.8 (c = 1.0, $CDCl_3$).

Chiral SFC: Daicel Chiralpak OB-H, CO_2:^{i}PrOH = 91:9, 2 mL/min, λ = 278, t_R (major) = 13.4 min; t_R (minor) = 11.5 min, e.r. = 59:41.

5-(Hydroxymethyl)-1-((4-nitrophenyl)sulfonyl)pyrrolidin-2-one (3n)

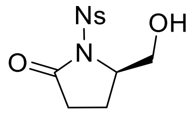

Alcohol **3n** was prepared according to **GP 3** using unsaturated *N*-acyl sulfonamide **1n** (57.0 mg, 0.20 mmol, 1.00 equiv), catalyst **C2** (29.3 mg, 0.04 mmol, 0.2 equiv), and toluene (2.0 mL). The reaction mixture was stirred for 2 h. For the reduction, THF (2.0 mL), acetic acid (0.1 mL, 0.2 mmol, 1.0 equiv), and sodium triacetoxyborohydride (46.6 mg, 0.22 mmol, 1.10 equiv) were employed, and the mixture was stirred for 2 h. The crude material was purified by flash column chromatography (hexane:ethyl acetate = 2:3) to give **3n** as an light brown oil.

Yield: 32.4 mg, 0.108 mmol, 54%

^{1}H NMR (400 MHz, CD_3OD): δ (ppm) = 8.45 – 8.40 (m, 2H), 8.33 – 8.27 (m, 2H), 4.49 (dddd, J = 8.1, 4.0, 2.4, 1.5 Hz, 1H), 3.97 (dd, J = 11.5, 4.1 Hz, 1H), 3.75 (dd, J = 11.5, 2.5 Hz, 1H), 2.75 – 2.61 (m, 1H), 2.37 – 2.20 (m, 2H), 2.17 – 2.05 (m, 2H).

^{13}C NMR (101 MHz, CD_3OD): δ (ppm) = 176.7, 152.3, 145.6, 131.0, 125.1, 64.7, 62.9, 32.2, 23.5.

IR (thin film, cm^{-1}): 3419, 2927, 1741, 1607, 1476, 1350, 1169, 1108, 911, 855, 740, 653, 550.

HRMS (ESI, *m/z*): calcd for $C_{11}H_{12}N_2NaO_6S$ $[M+Na]^+$: 323.0308, found: 323.0301.

Chiral HPLC: Daicel Chiralpak AM-R, H_2O:ACN +0.1% FA = 75:25, 1 mL/min, λ = 262, t_R (major) = 17.6 min; t_R (minor) = 26.5 min, e.r. = 72:28.

8. Product derivatization

(*R*)-N-(1,5-Dihydroxypentan-2-yl)-4-methylbenzenesulfonamide (4)

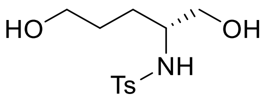

Alcohol **4** (30.0 mg, 0.11 mmol, 1.00 equiv) was added to a heat-dried 5 mL round-bottom flask equipped with a magnetic stirring bar and was dissolved in dry THF (1.1 mL, 0.1 M). Sodium borohydride (4.52 mg, 0.13 mmol, 1.20 equiv) was added and the solution was stirred at room temperature for 30 min. The mixture was quenched with water (1 mL), diluted with ethyl acetate (10 mL).The organic phase was washed with brine (10 mL) and the aqueous layer was extracted with ethyl acetate (10 mL). The combined organic phases were dried over anhydrous $MgSO_4$, filtered and concentrated under reduced pressure to yield the crude product, which was purified by silica gel flash column chromatography (dichloromethane:ethyl acetate = 1:4) to yield **4** as a pale-yellow oil.

Yield: 25.7 mg, 0.094 mmol, 85%

^{1}H NMR (400 MHz, $CDCl_3$): δ (ppm) = 7.81 – 7.71 (m, 2H), 7.28 (d, J = 8.0 Hz, 2H), 5.93 (d, J = 7.8 Hz, 1H), 3.62 – 3.46 (m, 4H), 3.39 (br s, 1H), 3.26 – 3.16 (m, 1H), 3.00 (br s, 1H), 2.40 (s, 3H), 1.67 – 1.31 (m, 4H).

^{13}C NMR (101 MHz, $CDCl_3$): δ (ppm) = 143.6, 137.8, 129.8, 127.2, 64.6, 62.3, 55.4, 28.3, 21.6.

IR (thin film, cm^{-1}): 3500, 2382, 2945, 2878, 1598, 1436, 1319, 1154, 1092, 815, 665, 550.

HRMS (ESI, *m/z*): calcd for $C_{12}H_{19}NNaO_4S$ $[M+Na]^+$: 296.0927, found: 296.0928.

$[\alpha]_D^{25^\circ}$ = -2.5 (c = 1.0, $CDCl_3$).

Chiral SFC: Daicel Chiralpak IB, CO_2:^{i}PrOH = 93:7, 2 mL/min, λ = 227, t_R (major) = 31.6 min; t_R (minor) = 29.9 min, e.r. = 83:17.

Note: The e.r. was retained between the cyclized alcohol and ring-opened diol derivative.

Methyl 4-((4-methylphenyl)sulfonamido)hex-5-ynoate (5)

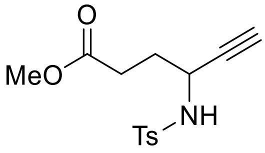

Aldehyde **5** (14.4 mg, 54.0 μmol, 1.00 equiv) was added to a heat-dried 5 mL round-bottom flask equipped with a magnetic stirring bar and and was dissolved in dry MeOH (0.6 ml, 0.1 M). Potassium carbonate (20.0 mg, 140 μmol, 2.60 equiv) and dimethyl (1-diazo-2-oxopropyl)phosphonate (21.0 mg, 108 μmol, 2.00 equiv) were added at 0 °C and the resulting mixture was stirred at the same temperature for 1 h. The mixture was quenched with saturated aqueous ammonium chloride solution (10 mL) and was extracted with ethyl acetate (3 x 20 mL). The combined organic phases were dried over anhydrous Na_2SO_4.The solvent was removed under vacuum and the residue was purified by column chromatography (hexane:ethyl acetate = 7:3) to give **5** as a pale-yellow oil.

Yield: 12.1 mg, 0.041 mmol, 76%

^{1}H NMR (400 MHz, $CDCl_3$): δ (ppm) = 7.79 – 7.74 (m, 2H), 7.33 – 7.28 (m, 2H), 4.82 (d, J = 9.0 Hz, 1H), 4.16 (dtd, J = 9.2, 6.9, 2.3 Hz, 1H), 3.68 (s, 3H), 2.50 (dt, J = 8.0, 7.2 Hz, 2H), 2.43 (s, 3H), 2.11 (d, J = 2.3 Hz, 1H), 2.00 (q, J = 7.1 Hz, 2H).

^{13}C NMR (101 MHz, $CDCl_3$): δ (ppm) = 173.4, 143.8, 137.3, 129.7, 127.5, 81.2, 73.3, 52.0, 45.0, 31.5, 29.9, 21.7.

IR (thin film, cm^{-1}): 3271, 2954, 1733, 1599, 1496, 1333, 1261, 1206, 1160, 981, 894, 706, 668, 546.

HRMS (ESI, *m/z*): calcd for $C_{14}H_{17}NNaO_4S$ $[M+Na]^+$: 318.0770, found: 318.0766.

Chiral SFC: Daicel Chiralpak OB-H, CO_2:^{i}PrOH = 91:9, 2 mL/min, λ = 278, t_R (major) = 13.4 min; t_R (minor) = 11.5 min, e.r. = 60:40.

5-(Hydroxymethyl)-1-((4-nitrophenyl)sulfonyl)pyrrolidin-2-one (6)

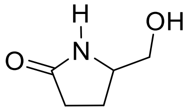

Alcohol **6** (31.0 mg, 0.10 mmol, 1.00 equiv) was added to a oven-dried 10 mL round-bottom flask equipped with a magnetic stirring bar and was dissolved in dry MeCN (2.7 ml) and DMSO (0.1 ml, 0.04 M). Potassium carbonate (55.3 mg, 0.40 mmol, 4.00 equiv) and thiophenol (33.1 mg, 0.03 ml, 3.00 equiv) were added at room temperature and the resulting mixture was stirred for 2 h. The mixture was quenched with sat. aq. NH_4Cl solution (10 mL) and was extracted with ethyl acetate (3 x 20 mL). The organic phases were combined and dried over anhydrous Na_2SO_4.The solvent was removed under vacuum and the residue was purified by column chromatography (ethyl acetate:acetic acid = 98:2) to give **6** as a colourless oil.

Yield: 8.6 mg, 0.075 mmol, 75%

^{1}H NMR (400 MHz, $CDCl_3$): δ (ppm) = 7.09 (d, J = 16.9 Hz, 1H), 3.93 – 3.75 (m, 2H), 3.73 – 3.60 (m, 1H), 3.46 (dd, J = 11.3, 7.1 Hz, 1H), 2.44 – 2.25 (m, 2H), 2.24 – 2.10 (m, 1H), 1.79 (dddd, J = 13.0, 9.5, 7.4, 5.5 Hz, 1H).

^{13}C NMR (101 MHz, $CDCl_3$): δ (ppm) = 179.4, 179.4, 66.1, 56.5, 30.4, 22.8, 22.8.

IR (thin film, cm^{-1}): 3279, 2931, 1670, 1463, 1423, 1284, 1091, 1057, 976, 648, 496

HRMS (ESI, *m/z*): calcd for $C_5H_9NNaO_2$ $[M+Na]^+$: 138.0525, found: 138.0527.

9. Mosher ester studies

((*R*)-5-oxo-1-tosylpyrrolidin-2-yl)methyl (R)-3,3,3-trifluoro-2-methoxy-2-phenylpropanoate (11a)

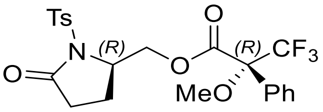

(*S*)-(+)-Mosher's acid chloride (13.0 µL, 75.5 µmol, 1.10 equiv) was added to a solution of **3a** (86:14 e.r., 18.5 mg, 68.5 µmol, 1.00 equiv), triethyl amine (0.02 mL, 0.14 mmol, 2.00 equiv) and DMAP (2.10 mg, 17.0 µmol, 0.25 equiv) in dry dichloromethane (0.8 mL, 0.08 M). After stirring the reaction mixture at room temperature for 2 h, TLC analysis indicated completion of the reaction. Water (1 mL) was added and the mixture was stirred for 5 min. The mixture was diluted with ethyl acetate (10 mL), washed with brine (10 mL), and concentrated under reduced pressure. The residue was purified by preparative TLC (hexane:ethyl acetate = 7:3) to obtain ester **11a** (single diastereomer) as a pale-yellow oil.

Yield: 16.7 mg, 34.3 µmol, 50%

^{1}H NMR (400 MHz, $CDCl_3$): δ (ppm) = 8.01 – 7.91 (m, 2H), 7.45 – 7.29 (m, 7H), 4.90 (dd, J = 12.0, 2.9 Hz, 1H), 4.69 – 4.61 (m, 1H), 4.29 (dd, J = 12.0, 2.0 Hz, 1H), 3.23 (d, J = 1.3 Hz, 3H), 2.41 (s, 3H), 2.29 – 2.07 (m, 3H), 1.80 – 1.64 (m, 1H).

^{13}C NMR (101 MHz, $CDCl_3$): δ (ppm) = 173.5, 166.4, 145.6, 135.5, 132.1, 129.9, 129.8, 128.7, 128.7, 127.0, 124.3, 122.0, 68.1, 57.3, 55.5, 30.7, 22.4, 21.8.

IR (thin film, cm^{-1}): 2955, 1748, 1360, 1274, 1168, 1123, 1024, 676, 553.

HRMS (ESI, *m/z*): calcd for $C_{22}H_{23}F_3NO_6S$ $[M+H]^+$: 486.1193, found: 486.1185.

$[\alpha]_D^{24^\circ}$ = -14.0 (c = 0.5, $CDCl_3$).

((*R*)-5-oxo-1-tosylpyrrolidin-2-yl)methyl (S)-3,3,3-trifluoro-2-methoxy-2-phenylpropanoate (11b)

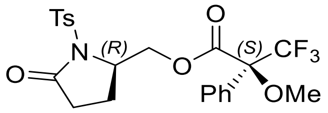

(*R*)-(+)-Mosher's acid chloride (13.0 µL, 75.5 µmol, 1.10 equiv) was added to a solution of **3a** (86:14 e.r., 18.5 mg, 68.5 µmol, 1.00 equiv), triethyl amine (0.02 mL, 0.14 mmol, 2.00 equiv) and DMAP (2.10 mg, 17.0 µmol, 0.25 equiv) in dry dichloromethane (0.8 mL, 0.08 M). After stirring the reaction mixture at room temperature for 2 h, TLC analysis indicated completion of the reaction. Water (1 mL) was added and the mixture was stirred for 5 min. The mixture was diluted with ethyl acetate (10 mL), washed with brine (10 mL), and concentrated under reduced pressure. The residue was purified by preparative TLC (hexane:ethyl acetate = 7:3) to obtain ester **11b** (single diastereomer) as a pale-yellow oil.

Yield: 16.7 mg, 34.3 µmol, 50%

^{1}H NMR (400 MHz, $CDCl_3$): δ (ppm) = 7.99 – 7.90 (m, 2H), 7.43 – 7.38 (m, 1H), 7.38 – 7.32 (m, 4H), 7.32 – 7.28 (m, 2H), 4.87 (dd, J = 11.8, 3.7 Hz, 1H), 4.71 – 4.62 (m, 1H), 4.39 (dd, J = 11.9, 2.3 Hz, 1H), 3.29 (d, J = 1.1 Hz, 3H), 2.38 (s, 3H), 2.30 – 2.16 (m, 3H), 1.93 – 1.78 (m, 1H).

^{13}C NMR (101 MHz, $CDCl_3$): δ (ppm) = 173.3, 166.3, 145.6, 135.4, 131.3, 130.0, 129.7, 128.8, 128.6, 127.5, 124.3, 122.0, 67.5, 57.2, 55.2, 30.6, 22.4, 21.7.

IR (thin film, cm^{-1}): 2953, 2850, 1748, 1358, 1275, 1240, 1168, 1122, 1019, 676, 553.

HRMS (ESI, *m/z*): calcd for $C_{22}H_{23}F_3NO_6S$ $[M+H]^+$: 486.1193, found: 486.1190

$[\alpha]_D^{24^\circ}$ = -35.1 (c = 0.5, $CDCl_3$).

With the Mosher esters in hand, we proceeded to perform a series of 2D and 1D NMR experiments and 3D modelling based on the review by Riguera et al.[13]

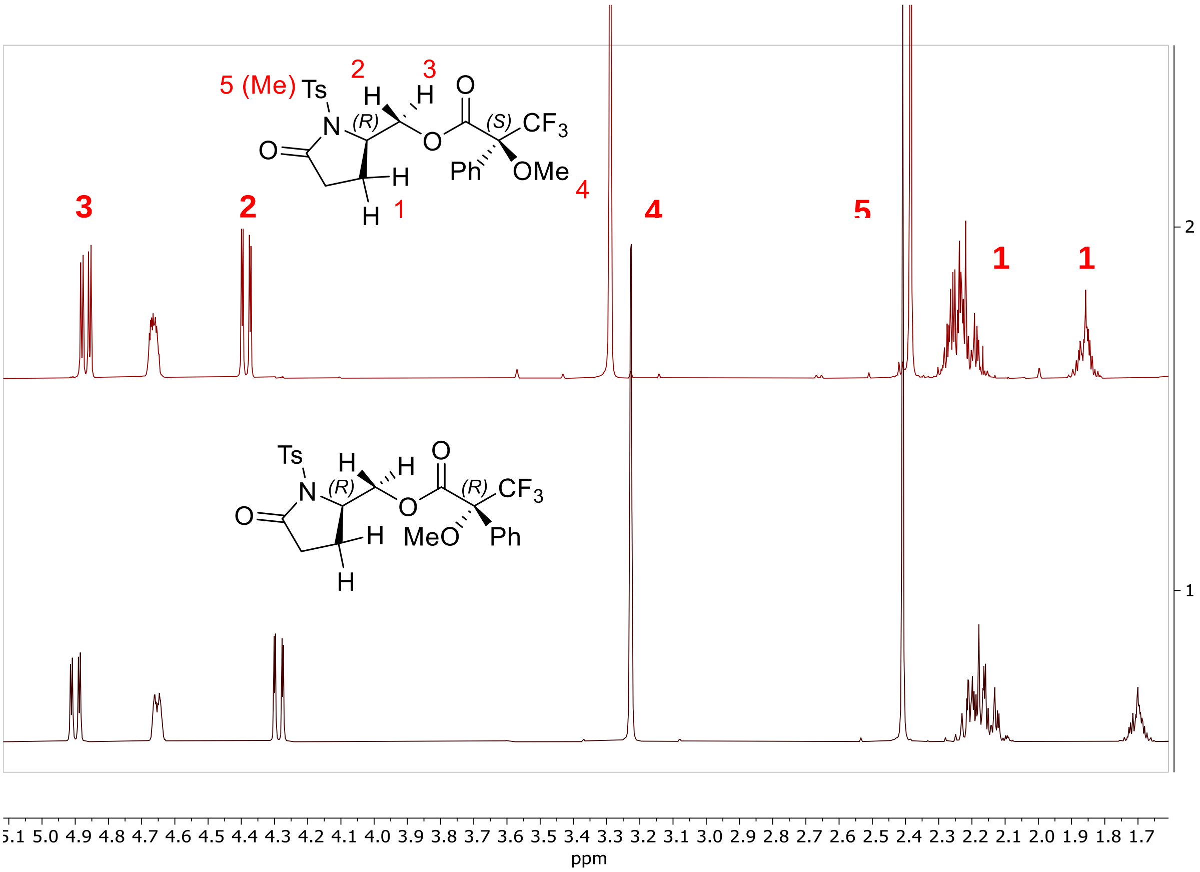

Figure 1. ^{1}H NMR stack between the two Mosher esters (5.1 – 1.6 ppm).

As can be observed in Figure 1, the phenyl group on the Mosher ester moiety is in closest proximity to the former-primary alcohol protons 2 and 3. The respective shielding effect on proton 2 is noted in the case of the *R*-ester while shielding of proton 3 is observed in the *S*-Ester. Another clear shielding effect is observed on proton 1 in the case of the *R*-ester, which was the first hint that the unknown stereocenter on alcohol **3a** is indeed R (as drawn). Since results on primary alcohols are not as straight-forward to corroborate, further investigation was carried on. Upon building a 3D model of the molecule, it could be noted that the tosyl group would be in close proximity with the Methoxy group in the *R*-ester and phenyl group in the S-ester. Appropriate shielding effects can be observed on Figure 1, as the methoxy protons (4)

[13] Seco, M. J.; Quiñoá, E.; Riguera, R.; *Chem. Rev.* 2004, *104*, *1*, 17–118.

are shielded in the case of the *R*-ester, whereas the methyl group of the tosyl moiety (5) shows slight shielding in the *S*-ester.

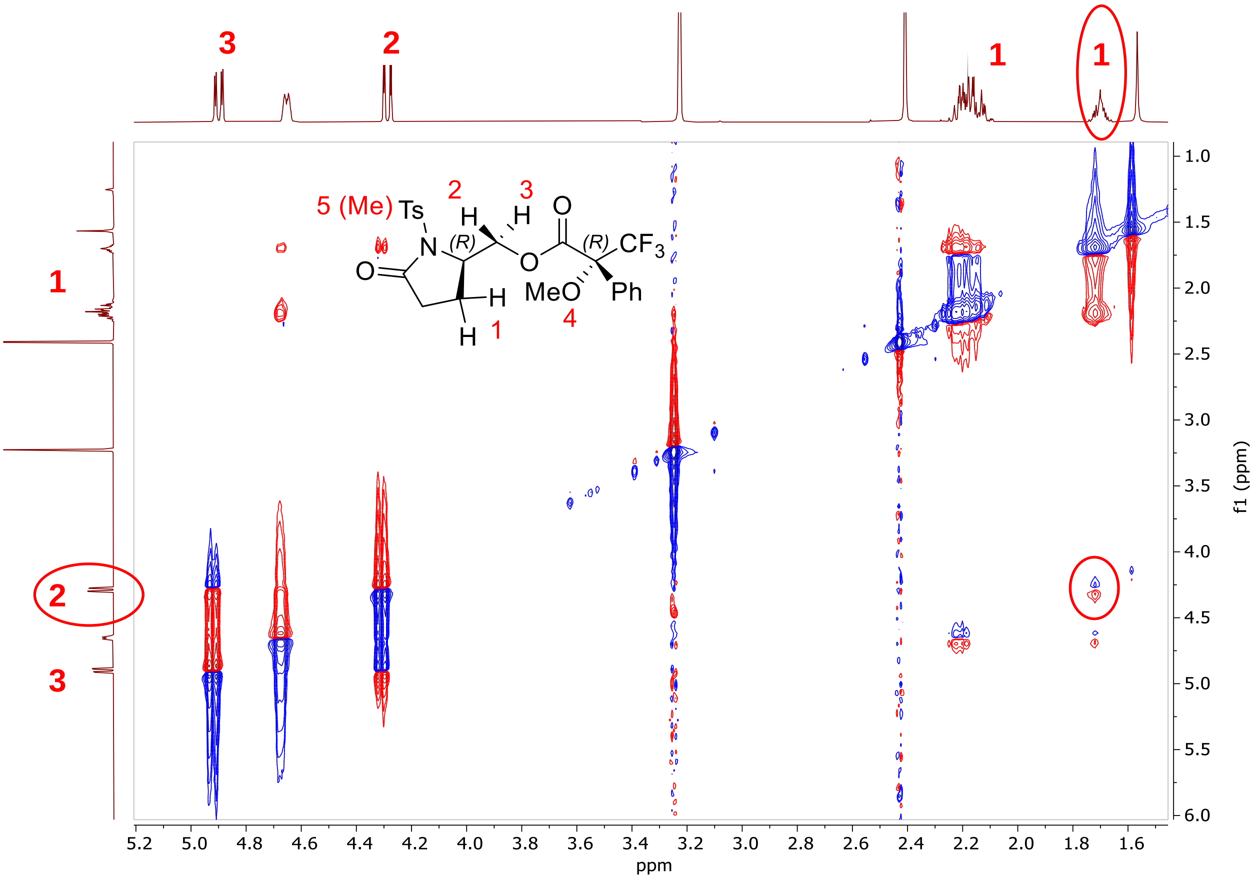

Figure 2. NOESY spectrum of the *R*,*R*-Mosher ester, focused on the relevant ppm range for analysis

In order to corroborate the shielding effects observed in Figure 1, a NOESY spectrum was measured. As can be noted on Figure 2, there is an NOE coupling between protons 1 and 2, which were both shielded in the *R*-ester. This further indicated that the shielding effect is consistent and that the absolute configuration of alcohol **3a** would be *R*.

Lastly, ^{19}F NMR spectra were measured of both esters, which allowed the comparison of the CF_3 group shifts (Figure 3). According to Riguera,[13] the coplanarity of the ester carbonyl group and the CF_3 group can be perturbed if the phenyl group is situated on the side of the bulkier substituent of the partnering alcohol moiety. In the case of our esters, that would be the side of the N-Ts moiety. When the coplanarity is lost, the CF_3 group moves from the deshielding zone to the shielding zone of the carbonyl group. This is also observed in Figure 3 as the *R*,*S*-ester CF_3 peak is more shielded than that

of the *R*,*R*-ester. With this data in hand, we have assigned the absolute configuration of the alcohol **3a** to be *R* (coming from the *R*,*R* Salen ligands). We can only speculate that this is translated to the rest of the substrates.

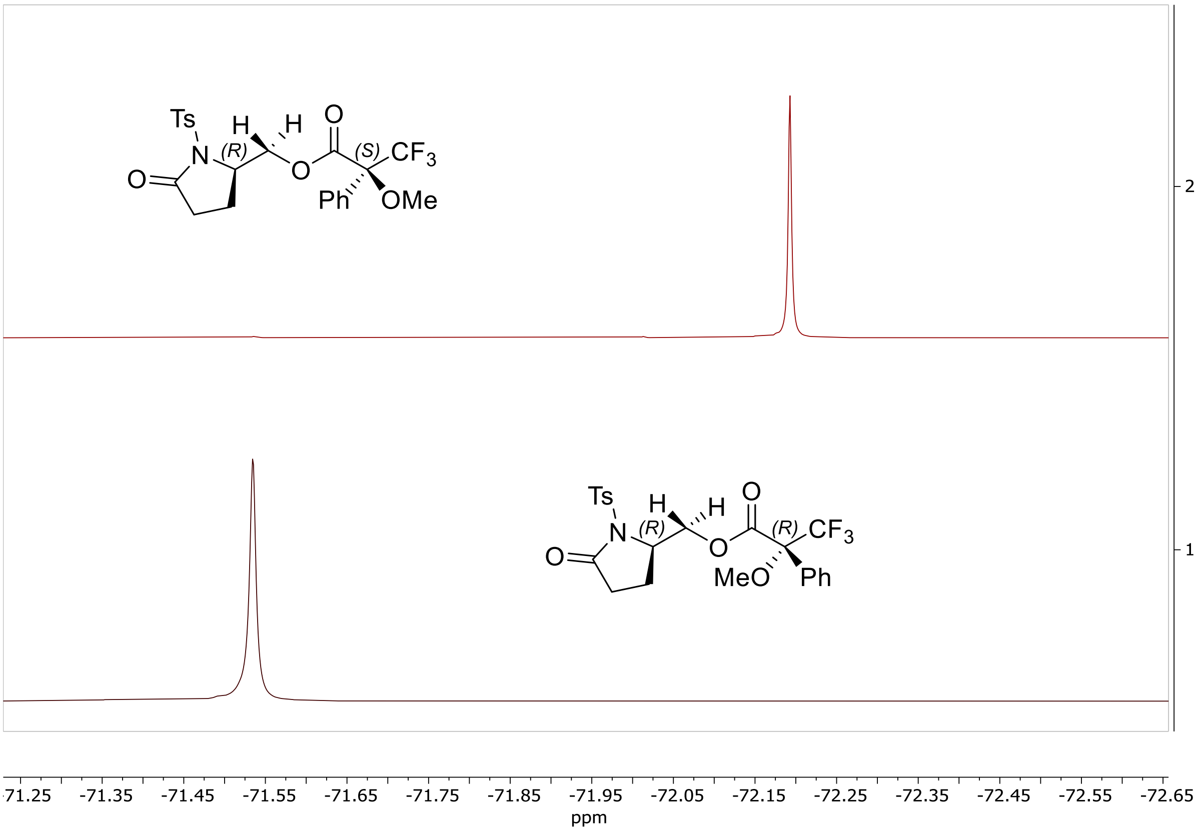

Figure 3. Stacked ^{19}F NMR spectra of the two Mosher esters.

10. Chiral Chromatography Data

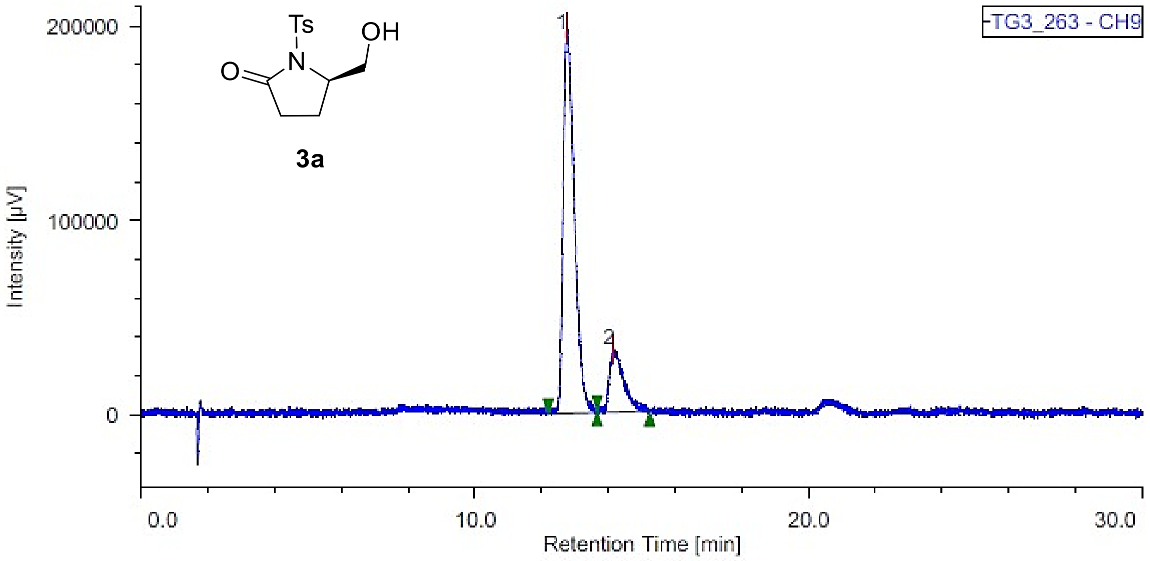

#	Peak Name	CH	tR [min]	Area [µV·sec]	Height [µV]	Area%	Height%	Quantity	NTP	Resolution	Symmetry Factor	Warning
1	Unknown	9	12.760	4613004	198457	82.690	86.031	N/A	6984	1.975	1.627	
2	Unknown	9	14.167	965636	32223	17.310	13.969	N/A	4807	N/A	1.484	

Racemic

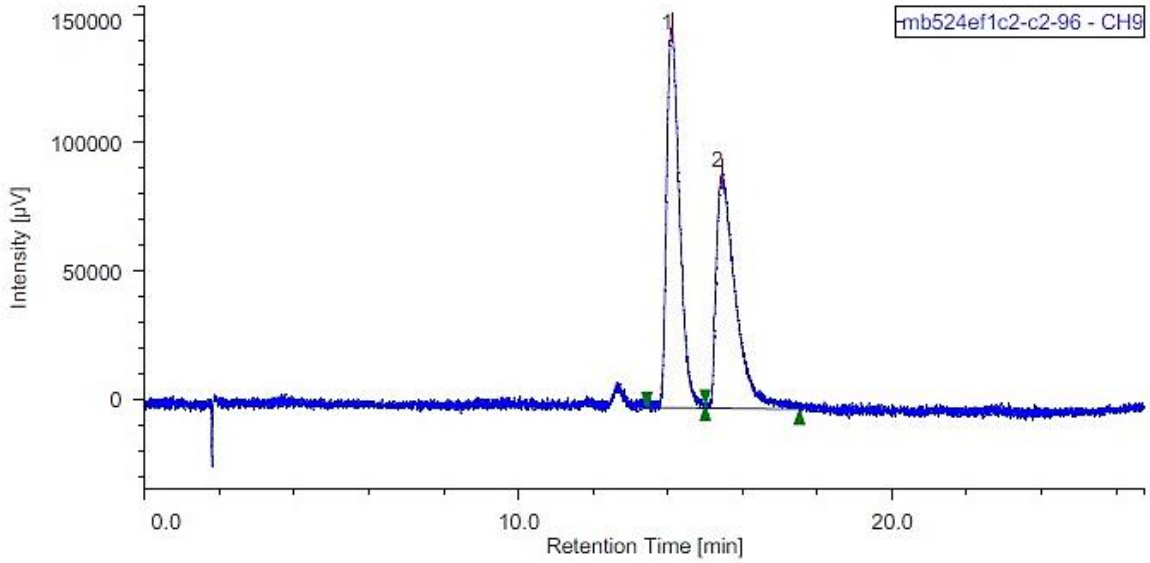

#	Peak Name	CH	tR [min]	Area [µV·sec]	Height [µV]	Area%	Height%	Quantity	NTP	Resolution	Symmetry Factor	Warning
1	Unknown	9	14.113	3541953	147754	51.948	61.816	N/A	8175	1.811	1.415	
2	Unknown	9	15.460	3276316	91267	48.052	38.184	N/A	5085	N/A	2.457	

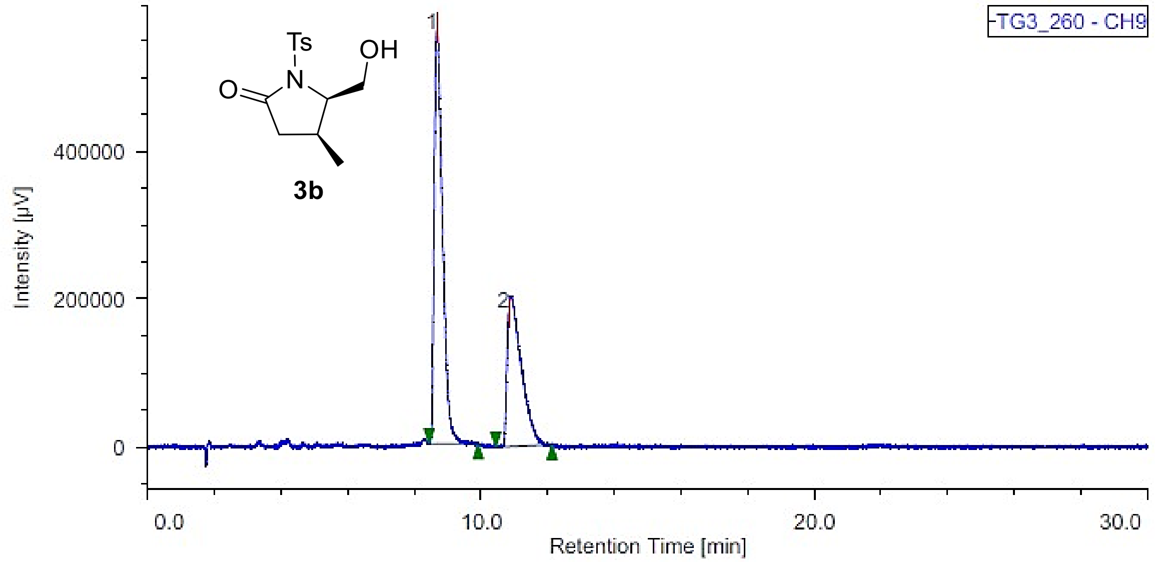

#	Peak Name	CH	tR [min]	Area [µV·sec]	Height [µV]	Area%	Height%	Quantity	NTP	Resolution	Symmetry Factor	Warning
1	Unknown	9	8.673	9709580	562701	61.982	75.685	N/A	5886	3.345	1.845	
2	Unknown	9	10.827	5955704	180777	38.018	24.315	N/A	2667	N/A	4.089	

Racemic

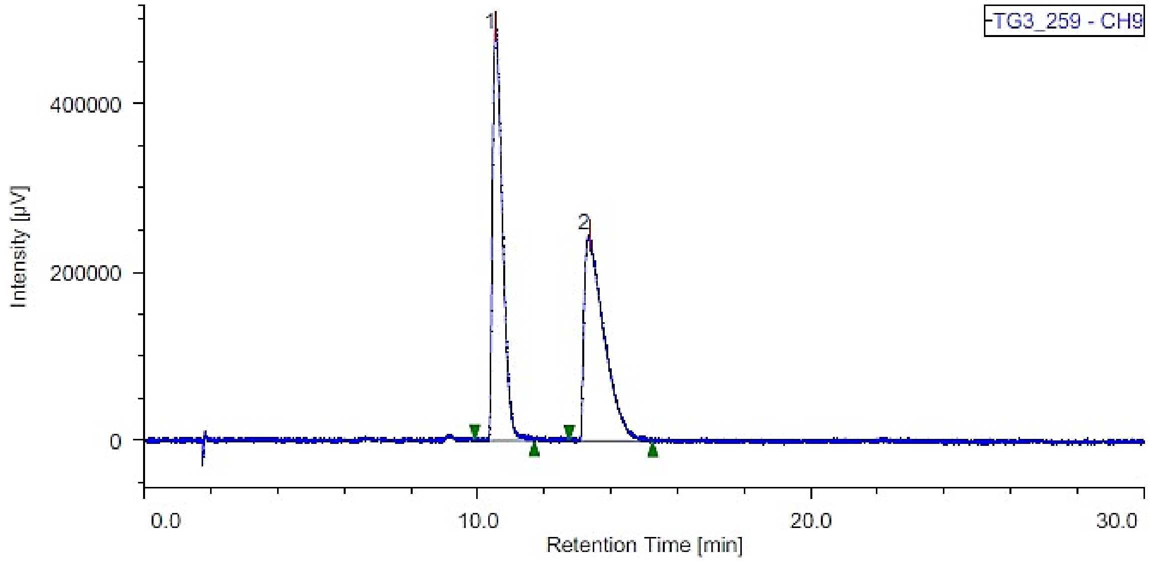

#	Peak Name	CH	tR [min]	Area [µV·sec]	Height [µV]	Area%	Height%	Quantity	NTP	Resolution	Symmetry Factor	Warning
1	Unknown	9	10.540	10158526	491006	49.944	66.742	N/A	5928	3.452	1.902	
2	Unknown	9	13.340	10181187	244675	50.056	33.258	N/A	2445	N/A	2.966	

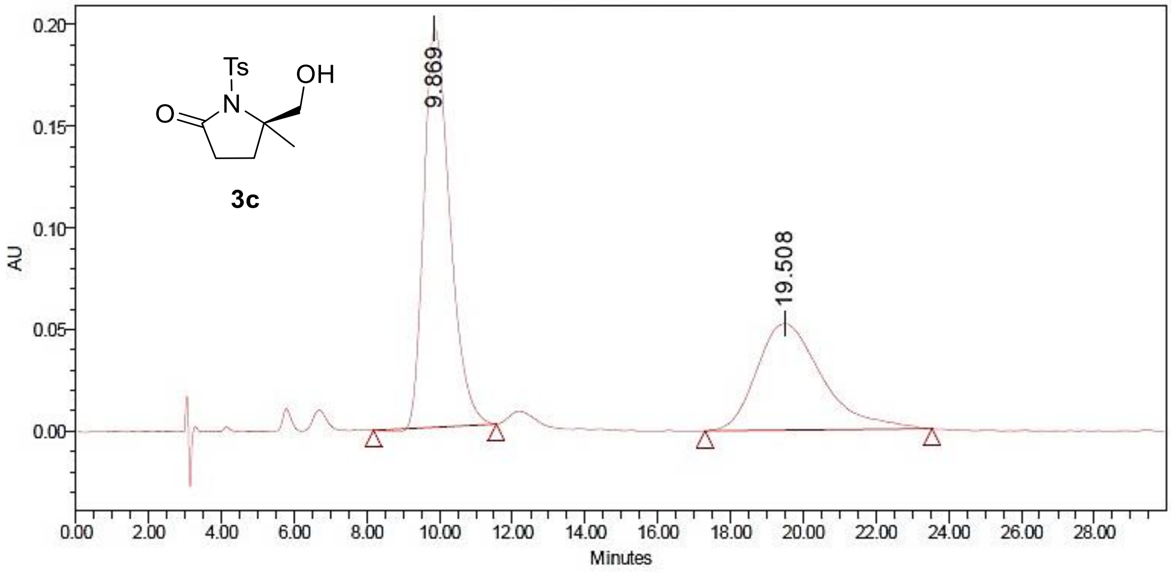

Peak Results

	Name	RT	Area	Height	Amount	Units	% Area
1		9.869	9752818	196089			59.76
2		19.508	6566020	52408			40.24

Racemic

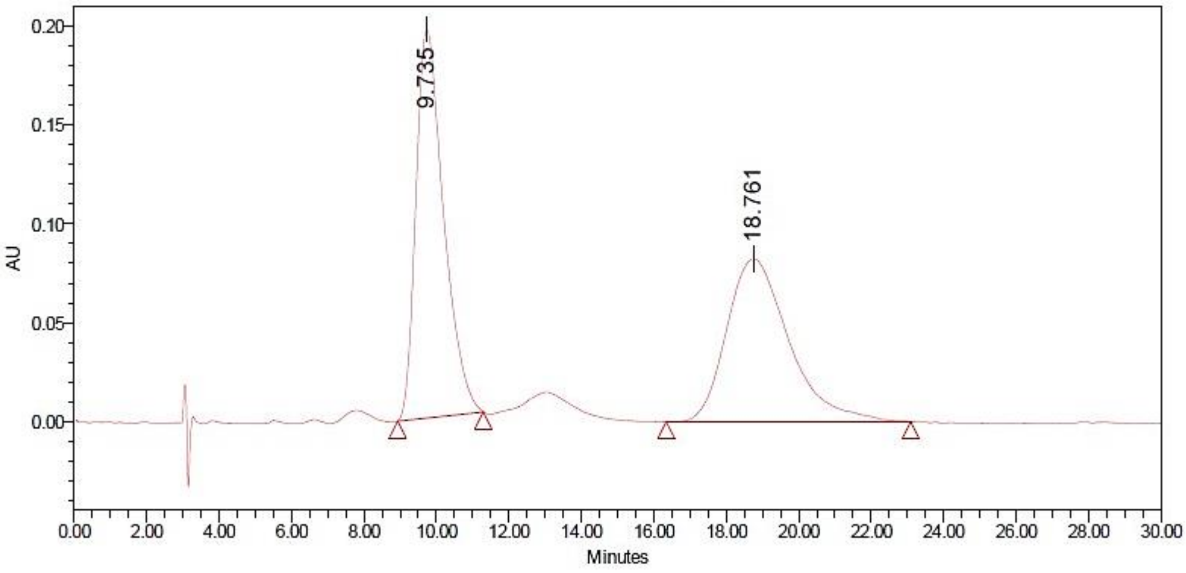

Peak Results

	Name	RT	Area	Height	Amount	Units	% Area
1		9.735	10422272	196271			51.50
2		18.761	9816905	82272			48.50

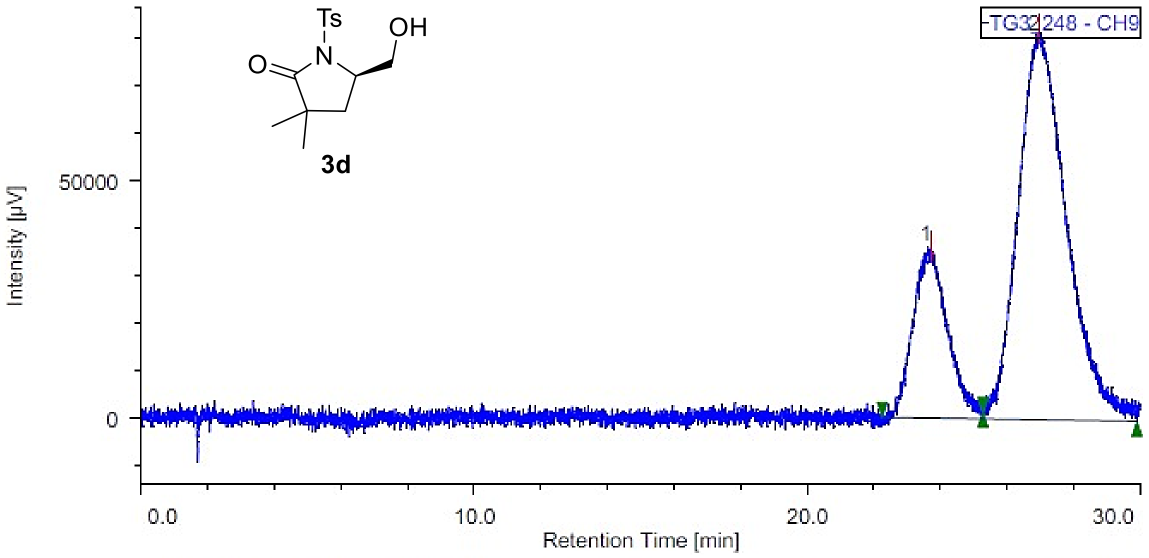

#	Peak Name	CH	tR [min]	Area [µV·sec]	Height [µV]	Area%	Height%	Quantity	NTP	Resolution	Symmetry Factor	Warning
2	Unknown	9	23.720	2558443	36131	23.699	30.533	N/A	2423	1.437	1.102	
1	Unknown	9	26.960	8237298	82205	76.301	69.467	N/A	1727	N/A	1.428	

Racemic

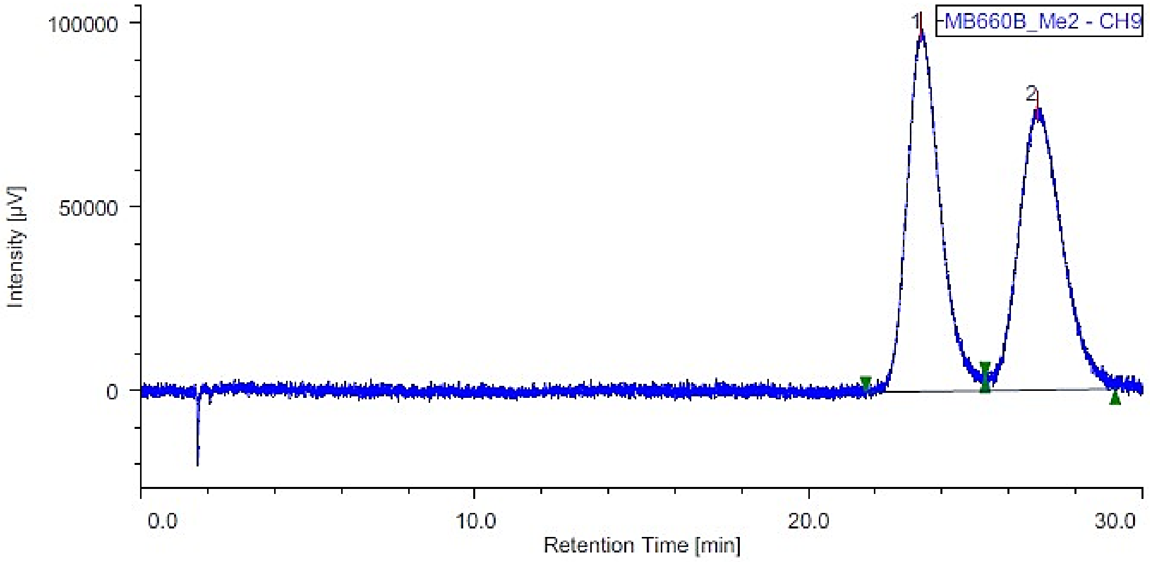

#	Peak Name	CH	tR [min]	Area [µV·sec]	Height [µV]	Area%	Height%	Quantity	NTP	Resolution	Symmetry Factor	Warning
1	Unknown	9	23.373	6822891	99217	49.385	56.231	N/A	2796	1.684	1.446	
2	Unknown	9	26.840	6992790	77227	50.615	43.769	N/A	2071	N/A	1.233	

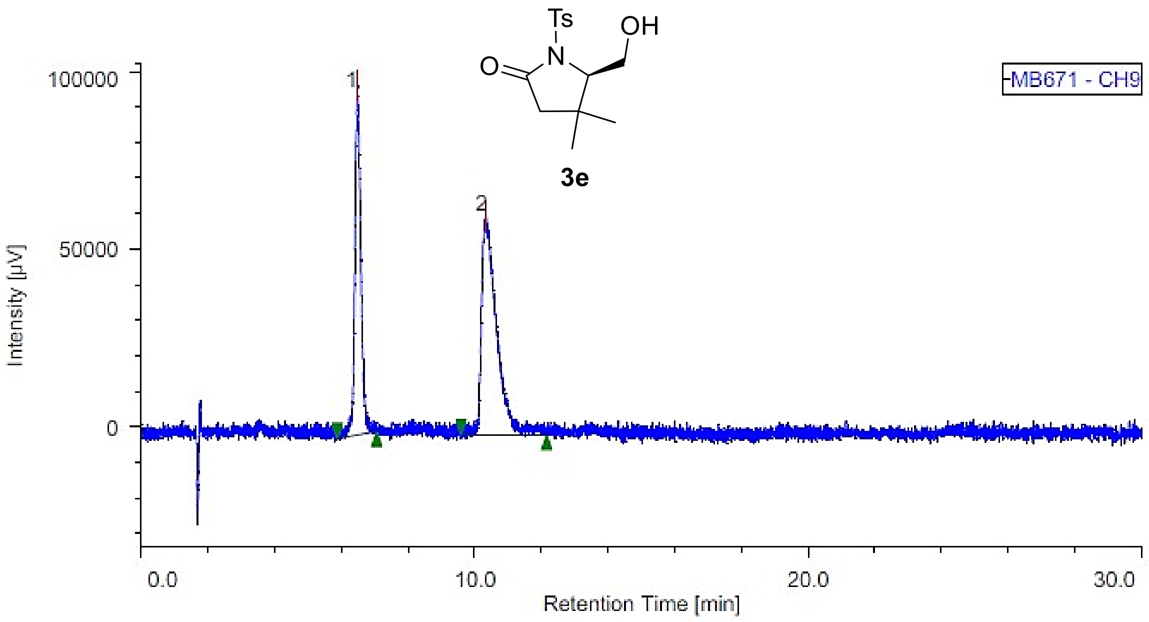

3e

#	Peak Name	CH	tR [min]	Area [µV·sec]	Height [µV]	Area%	Height%	Quantity	NTP	Resolution	Symmetry Factor	Warning
1	Unknown	9	6.473	1264365	98334	39.518	61.646	N/A	6929	7.221	1.090	
2	Unknown	9	10.360	1935119	61179	60.482	38.354	N/A	2909	N/A	1.726	

Racemic

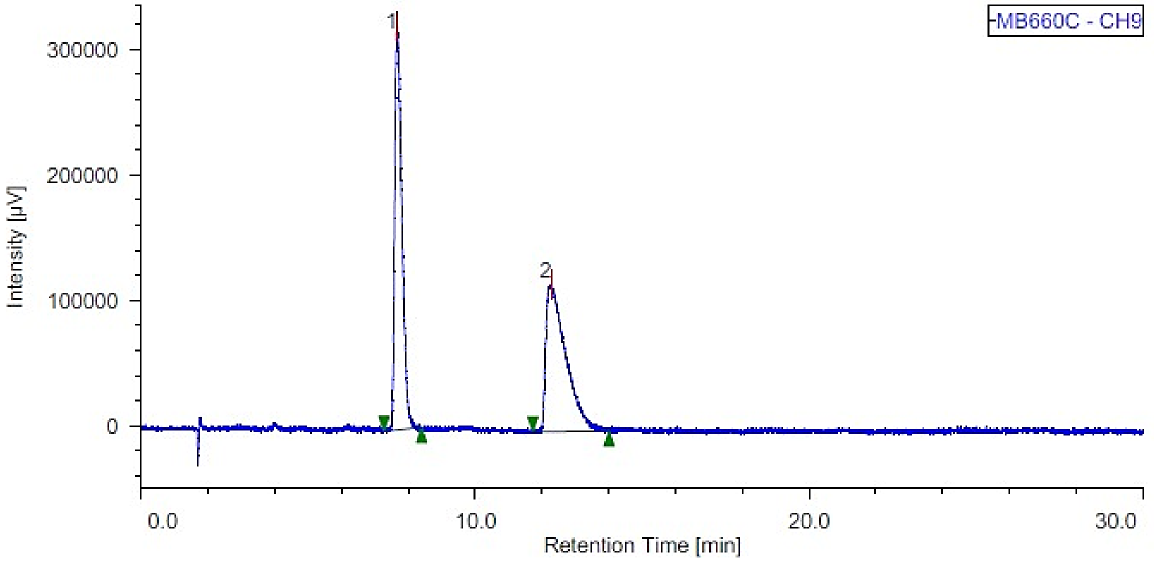

#	Peak Name	CH	tR [min]	Area [µV·sec]	Height [µV]	Area%	Height%	Quantity	NTP	Resolution	Symmetry Factor	Warning
1	Unknown	9	7.660	4772132	321240	49.457	73.202	N/A	6140	6.250	1.689	
2	Unknown	9	12.273	4876906	117602	50.543	26.798	N/A	2032	N/A	2.816	

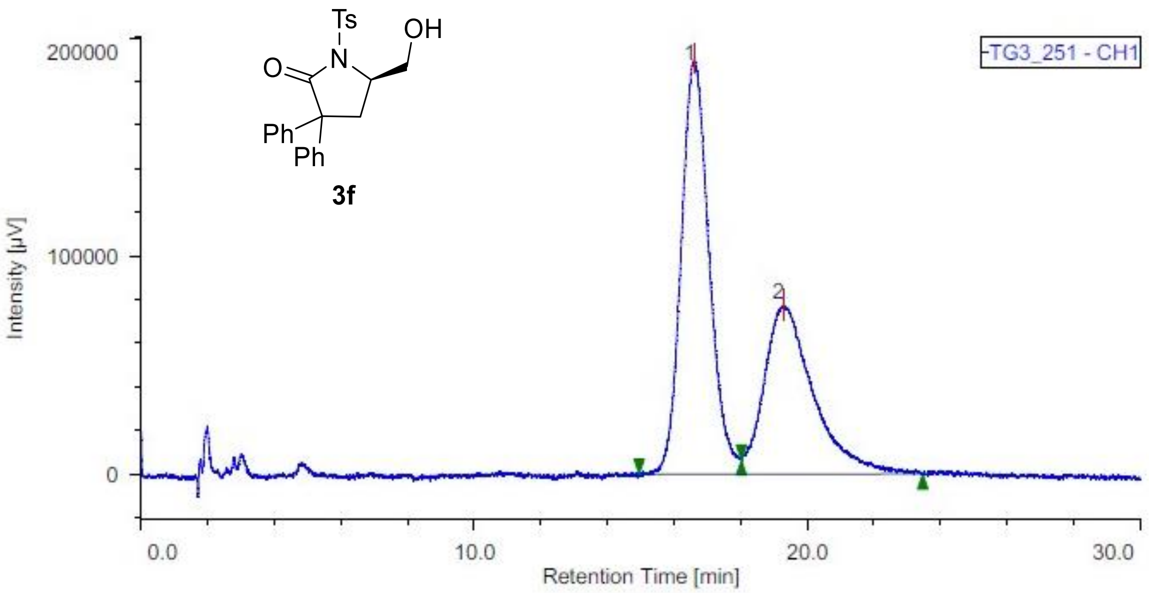

#	Peak Name	CH	tR [min]	Area [µV·sec]	Height [µV]	Area%	Height%	Quantity	NTP	Resolution	Symmetry Factor	Warning
1	Unknown	1	16.617	11100508	190547	58.345	71.022	N/A	1942	1.338	1.176	
2	Unknown	1	19.283	7925187	77745	41.655	28.978	N/A	961	N/A	N/A	

Racemic

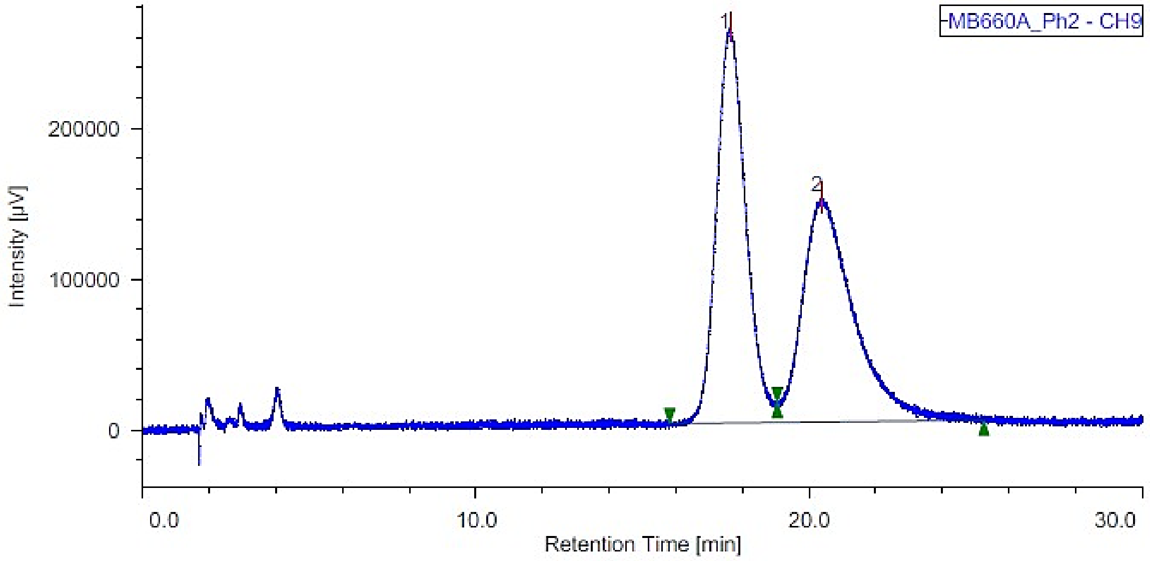

#	Peak Name	CH	tR [min]	Area [µV·sec]	Height [µV]	Area%	Height%	Quantity	NTP	Resolution	Symmetry Factor	Warning
1	Unknown	9	17.627	15979092	262339	50.156	63.746	N/A	1999	1.300	1.280	
2	Unknown	9	20.373	15879838	149201	49.844	36.254	N/A	939	N/A	N/A	

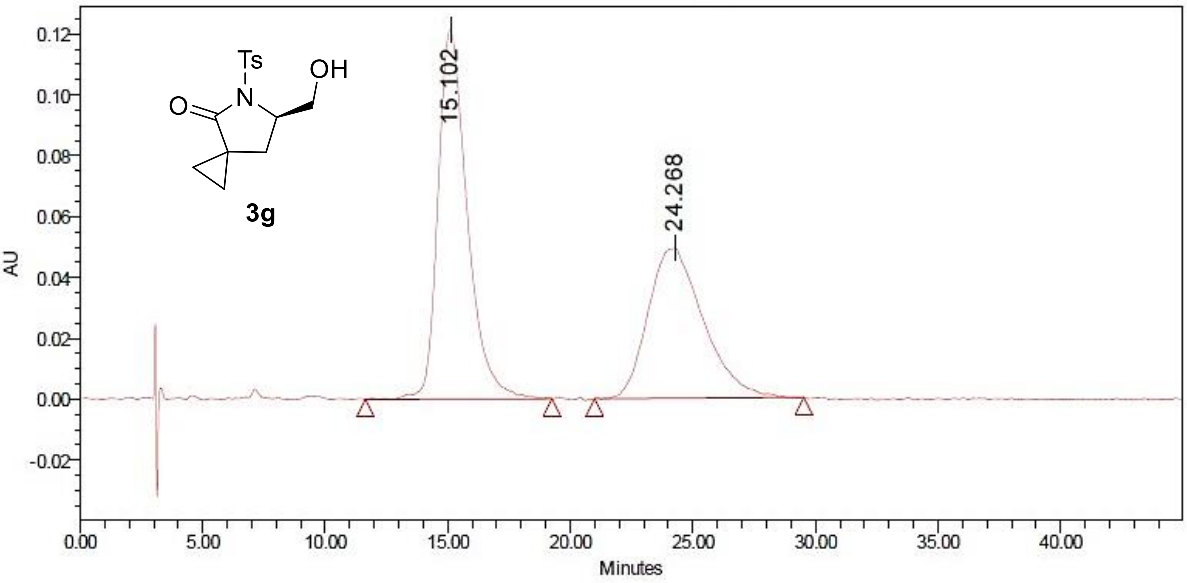

Peak Results

	Name	RT	Area	Height	Amount	Units	% Area
1		15.102	10074258	121385			56.60
2		24.268	7726318	49398			43.40

Racemic

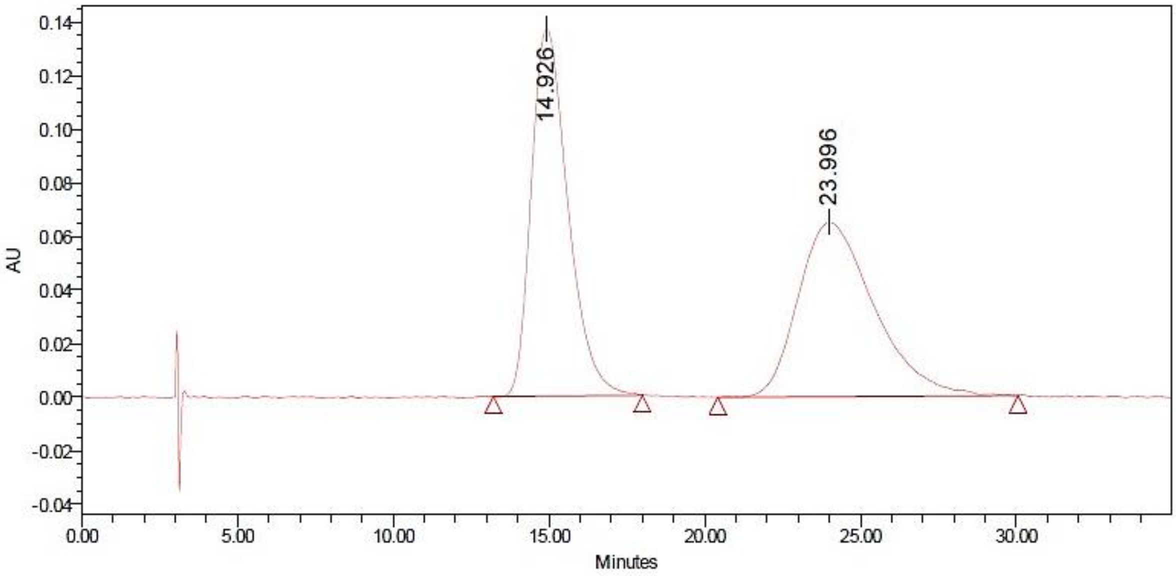

Peak Results

	Name	RT	Area	Height	Amount	Units	% Area
1		14.926	11207806	137066			50.39
2		23.996	11032892	65155			49.61

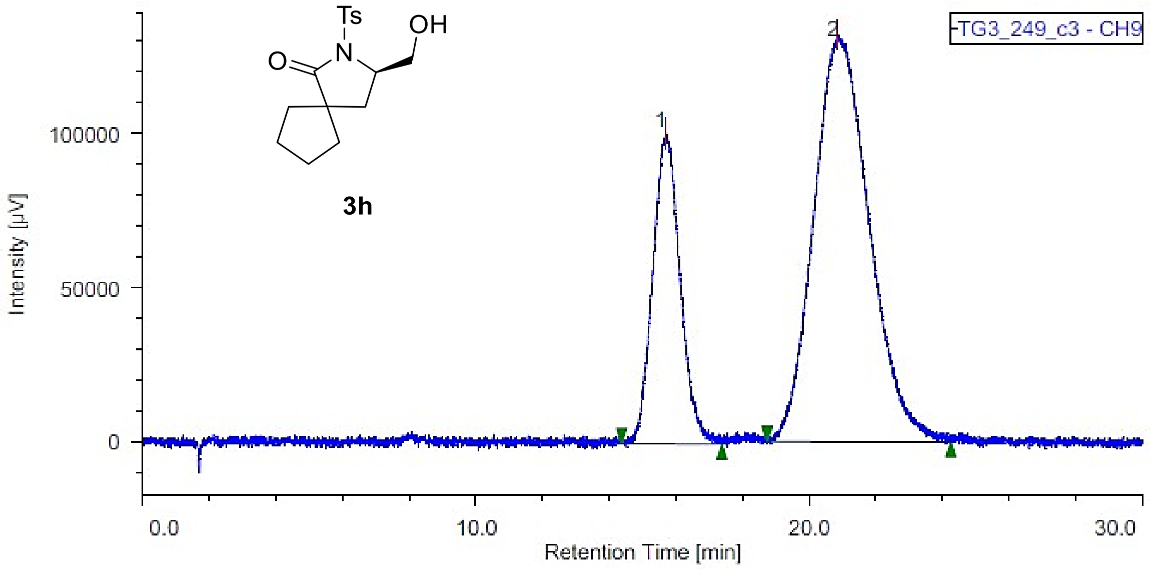

#	Peak Name	CH	tR [min]	Area [µV·sec]	Height [µV]	Area%	Height%	Quantity	NTP	Resolution	Symmetry Factor	Warning
1	Unknown	9	15.700	5751413	100728	27.383	43.319	N/A	1746	2.269	1.133	
2	Unknown	9	20.867	15252300	131798	72.617	56.681	N/A	743	N/A	1.213	

Racemic

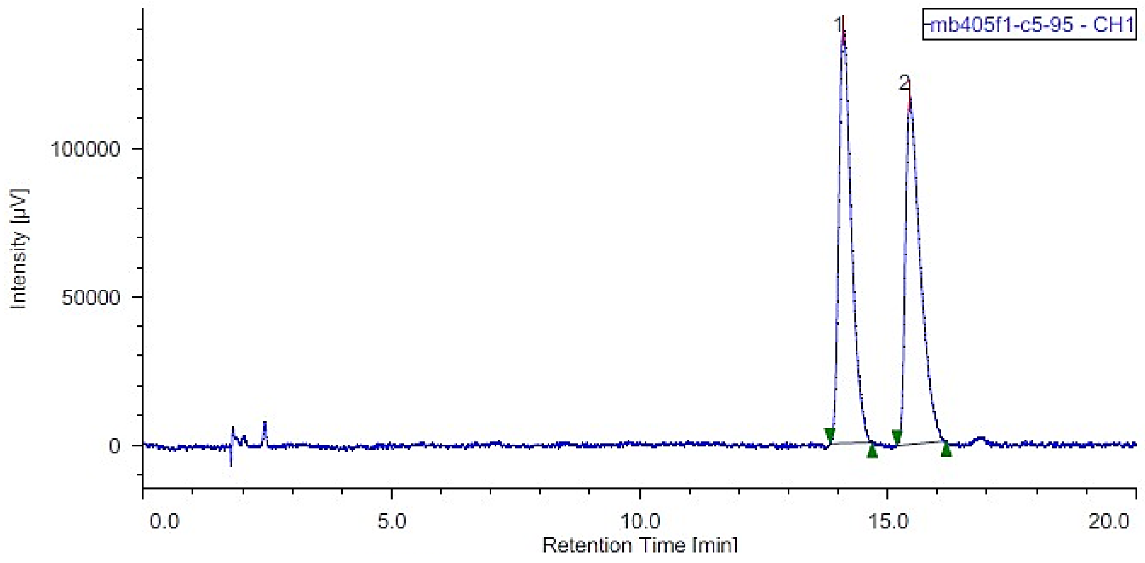

#	Peak Name	CH	tR [min]	Area [µV·sec]	Height [µV]	Area%	Height%	Quantity	NTP	Resolution	Symmetry Factor	Warning
1	Unknown	1	14.108	2460433	139051	50.184	54.263	N/A	15390	2.712	1.548	
2	Unknown	1	15.450	2442387	117201	49.816	45.737	N/A	13233	N/A	1.909	

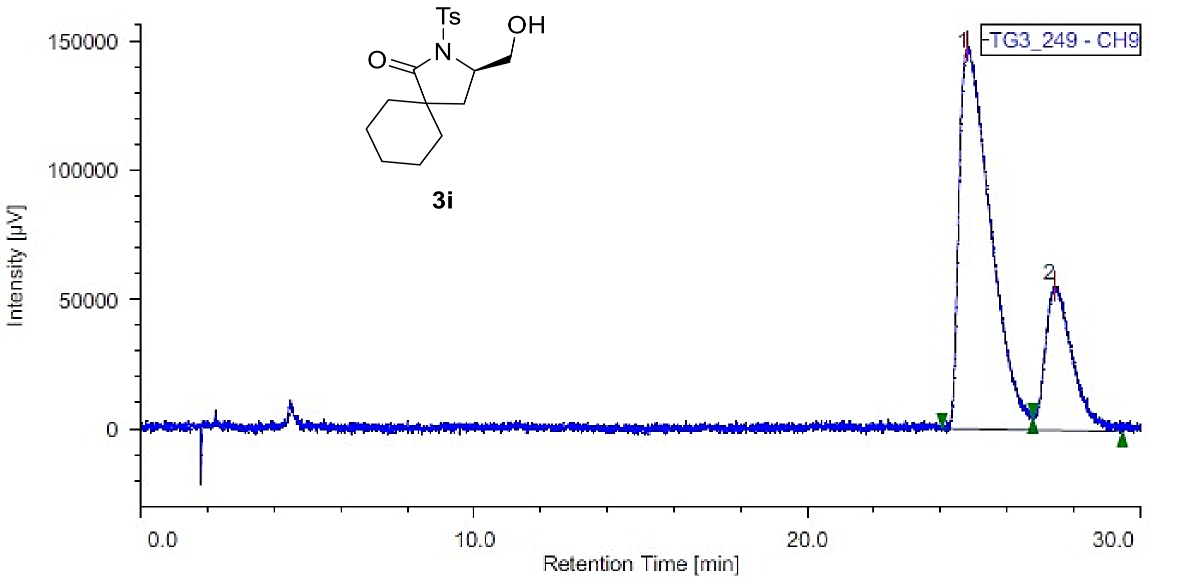

#	Peak Name	CH	tR [min]	Area [µV·sec]	Height [µV]	Area%	Height%	Quantity	NTP	Resolution	Symmetry Factor	Warning
1	Unknown	9	24.827	9654275	148056	75.067	72.666	N/A	3243	1.628	2.489	
2	Unknown	9	27.433	3206594	55693	24.933	27.334	N/A	5602	N/A	N/A	

Racemic

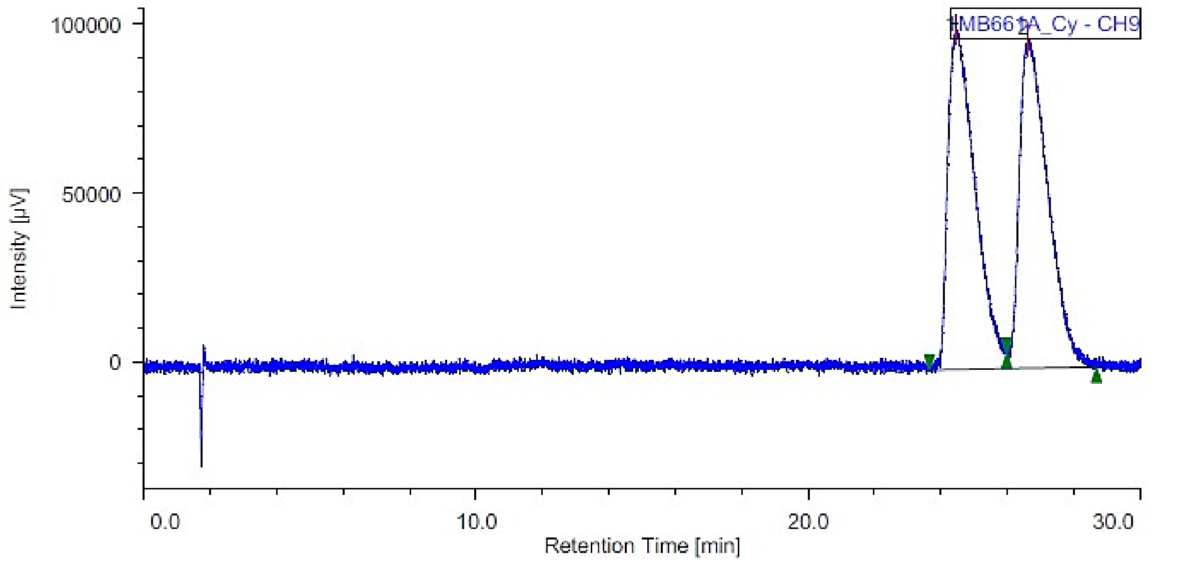

#	Peak Name	CH	tR [min]	Area [µV·sec]	Height [µV]	Area%	Height%	Quantity	NTP	Resolution	Symmetry Factor	Warning
1	Unknown	9	24.453	5679255	100588	49.277	50.883	N/A	4137	1.388	2.036	
2	Unknown	9	26.627	5845980	97097	50.723	49.117	N/A	4332	N/A	1.739	

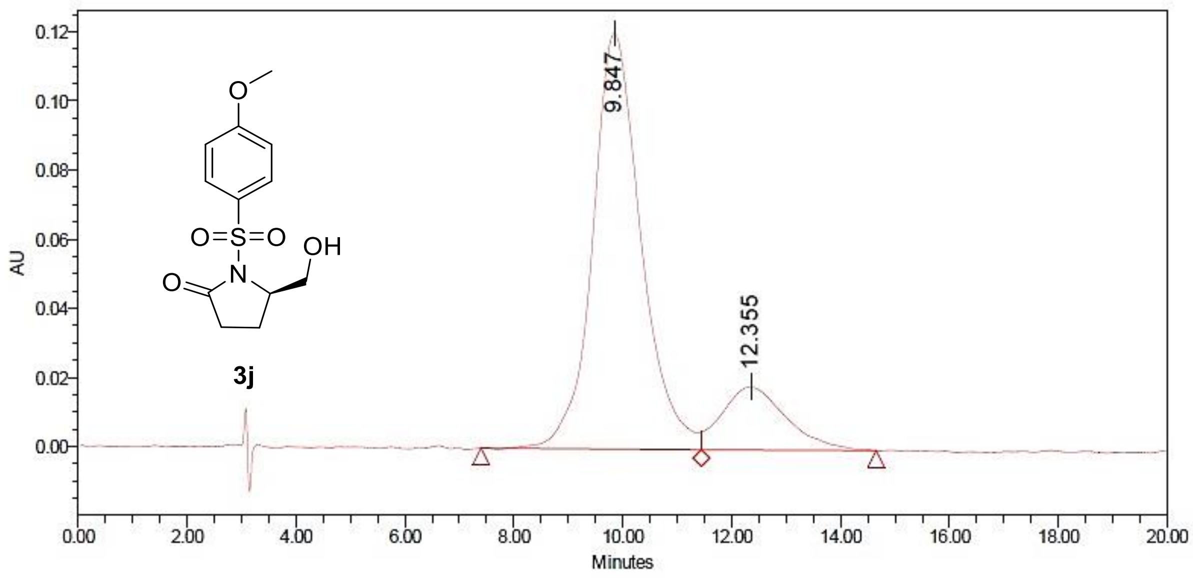

Peak Results

	Name	RT	Area	Height	Amount	Units	% Area
1		9.847	7441900	120288			83.28
2		12.355	1494184	18273			16.72

Racemic

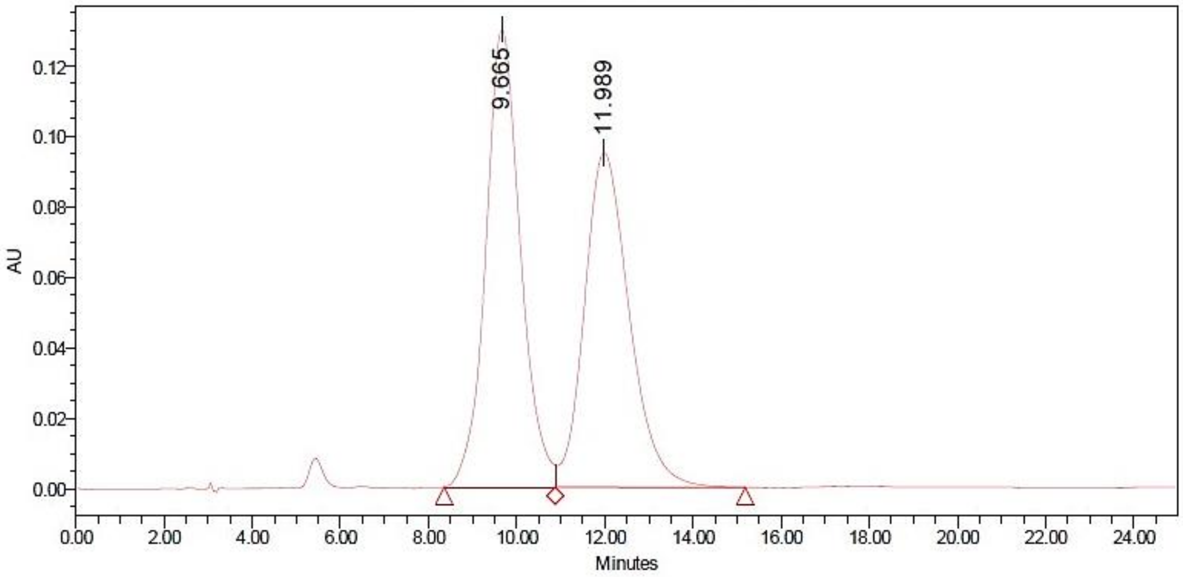

Peak Results

	Name	RT	Area	Height	Amount	Units	% Area
1		9.665	7113469	129972			51.41
2		11.989	6722716	95000			48.59

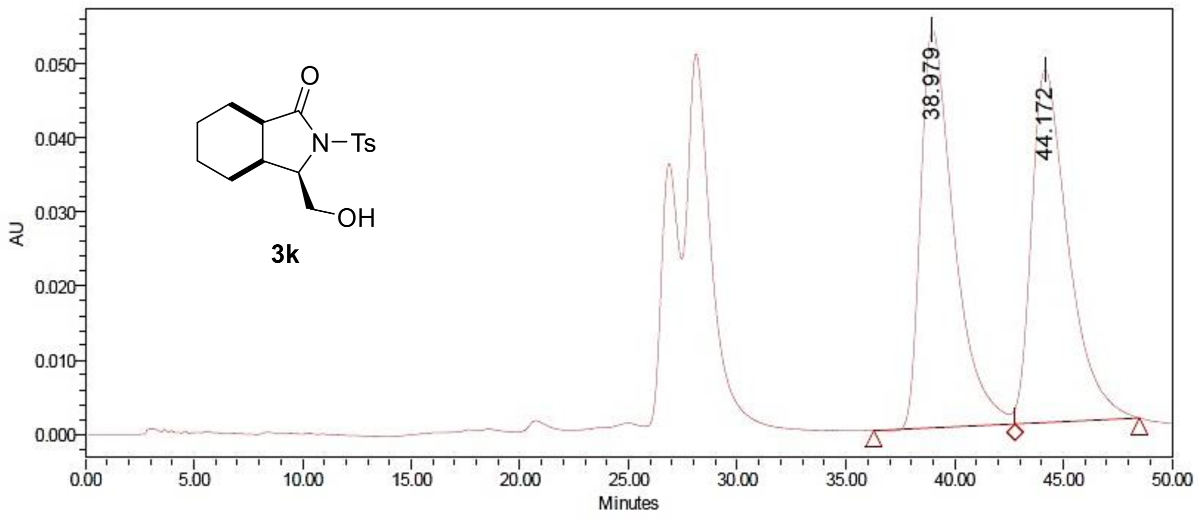

Peak Results

	Name	RT	Area	% Area	Height	Units
1		38.979	5759658	50.68	53751	
2		44.172	5605306	49.32	47644	

Racemic

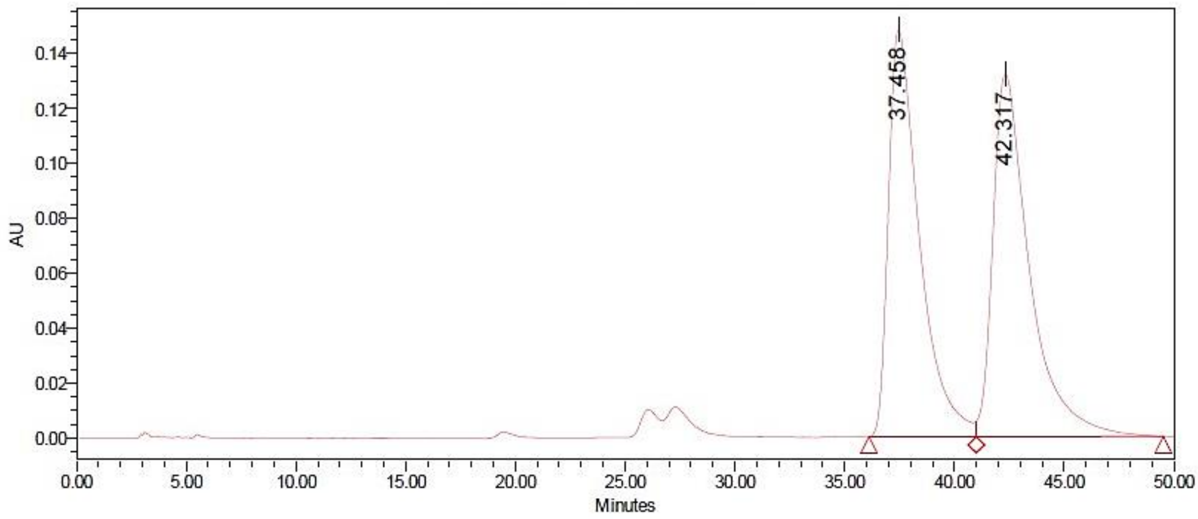

Peak Results

	Name	RT	Area	% Area	Height	Units
1		37.458	14528378	48.78	148075	
2		42.317	15254857	51.22	131862	

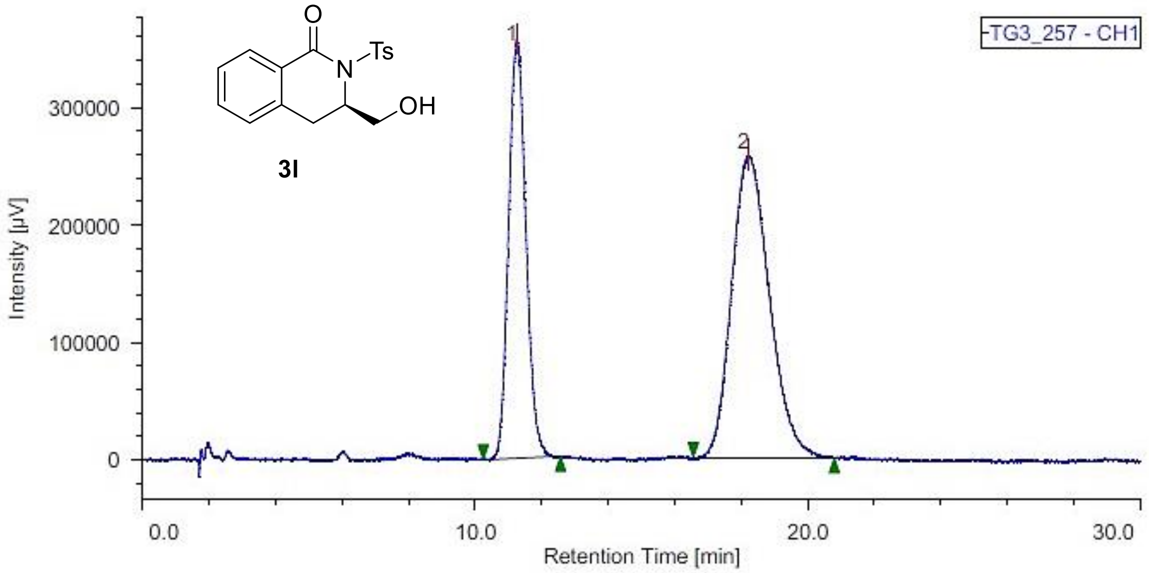

#	Peak Name	CH	tR [min]	Area [µV·sec]	Height [µV]	Area%	Height%	Quantity	NTP	Resolution	Symmetry Factor	Warning
1	Unknown	1	11.250	12863323	356955	38.843	58.073	N/A	2246	4.632	1.139	
2	Unknown	1	18.217	20253093	257707	61.157	41.927	N/A	1243	N/A	1.225	

Racemic

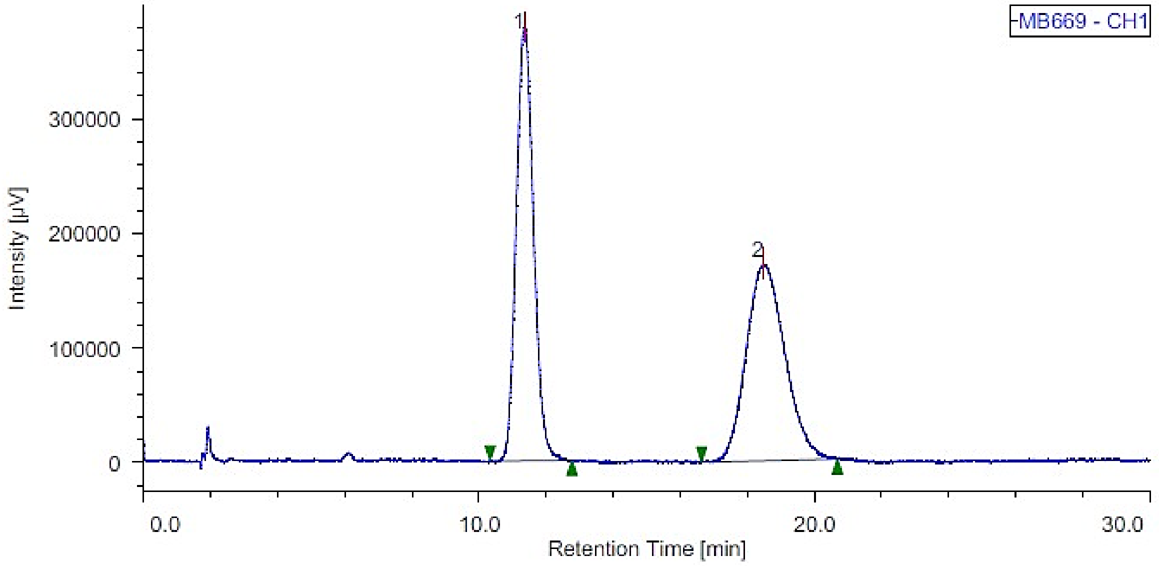

#	Peak Name	CH	tR [min]	Area [µV·sec]	Height [µV]	Area%	Height%	Quantity	NTP	Resolution	Symmetry Factor	Warning
1	Unknown	1	11.350	13580656	378024	49.685	68.661	N/A	2304	4.664	1.137	
2	Unknown	1	18.475	13752599	172541	50.315	31.339	N/A	1218	N/A	1.197	

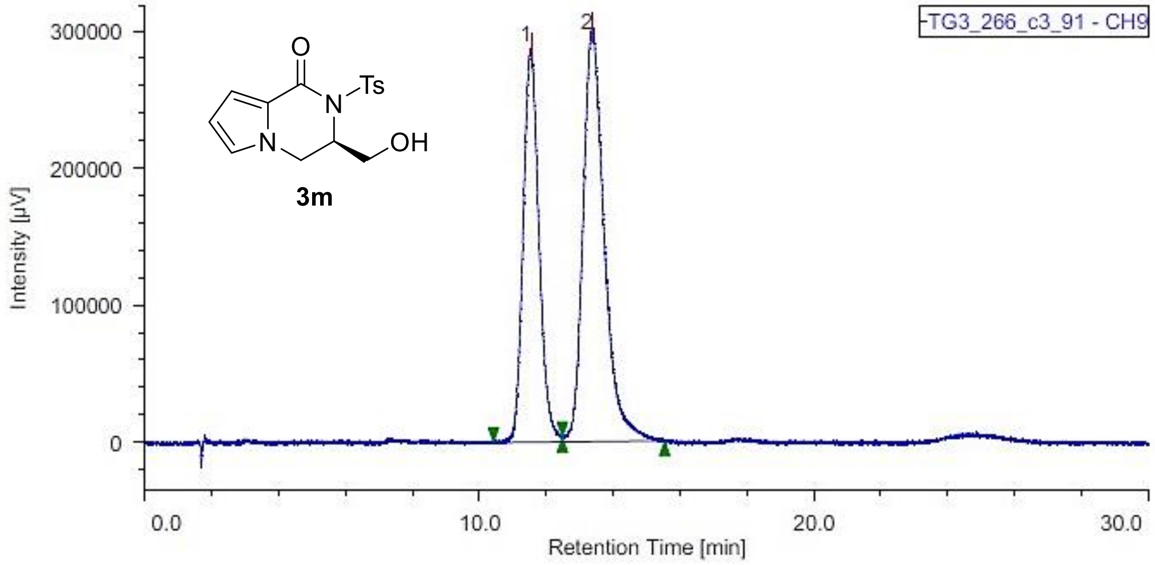

#	Peak Name	CH	tR [min]	Area [µV·sec]	Height [µV]	Area%	Height%	Quantity	NTP	Resolution	Symmetry Factor	Warning
2	Unknown	9	11.540	9427478	287636	41.339	48.781	N/A	2980	1.883	1.137	
1	Unknown	9	13.373	13377757	302007	58.661	51.219	N/A	2334	N/A	1.349	

Racemic

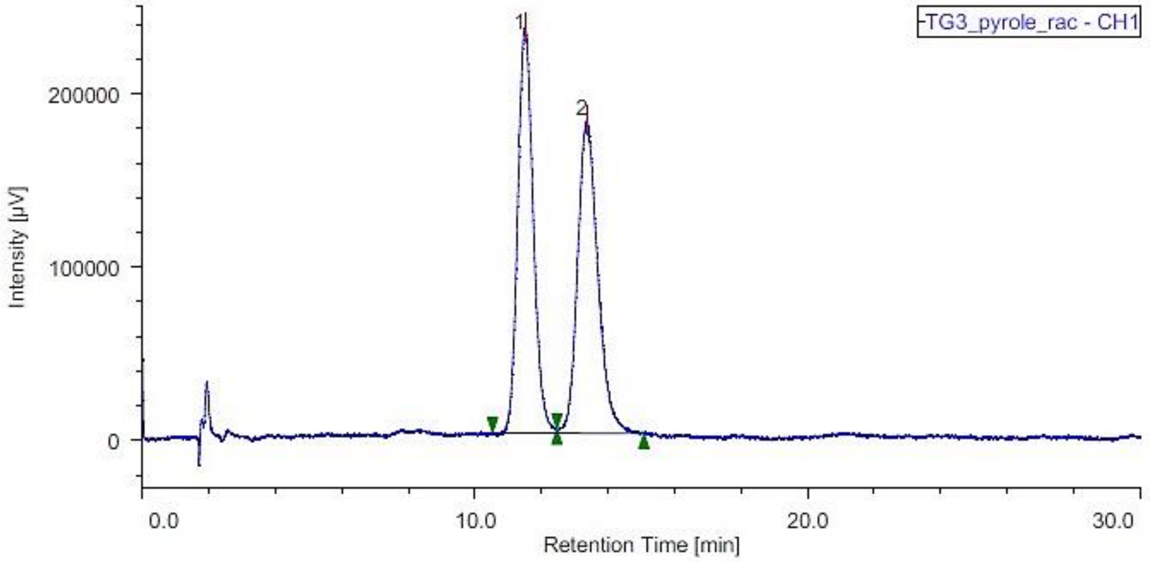

#	Peak Name	CH	tR [min]	Area [µV·sec]	Height [µV]	Area%	Height%	Quantity	NTP	Resolution	Symmetry Factor	Warning
1	Unknown	1	11.500	7559969	233591	49.680	56.419	N/A	2971	1.924	1.157	
2	Unknown	1	13.358	7657248	180436	50.320	43.581	N/A	2388	N/A	1.217	

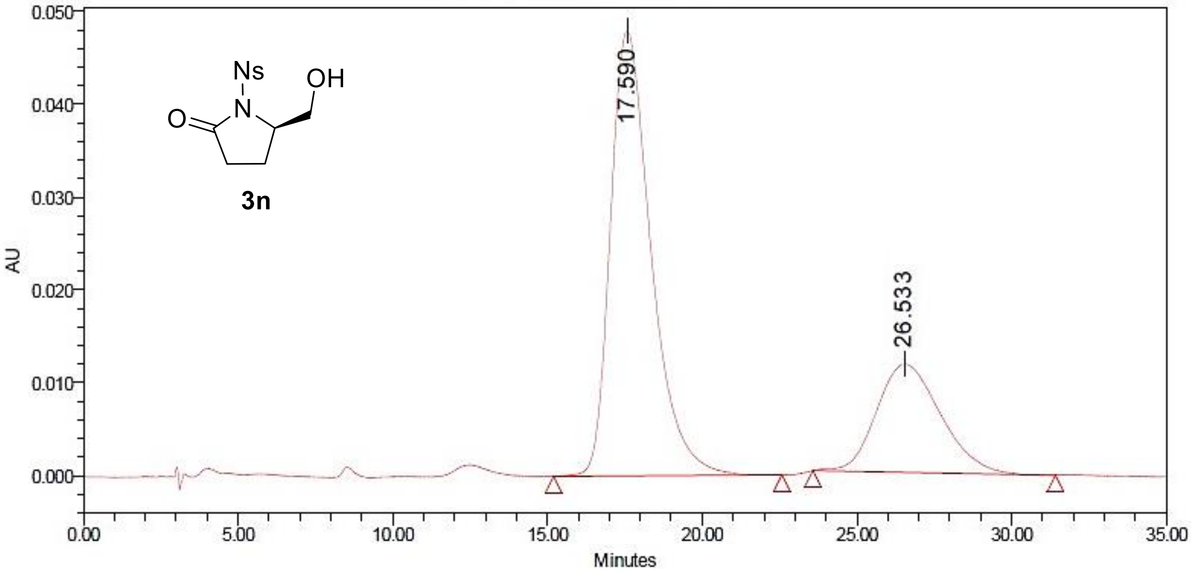

Peak Results

	Name	RT	Area	Height	Amount	Units	% Area
1		17.590	4383403	47968			72.25
2		26.533	1683539	11701			27.75

Racemic

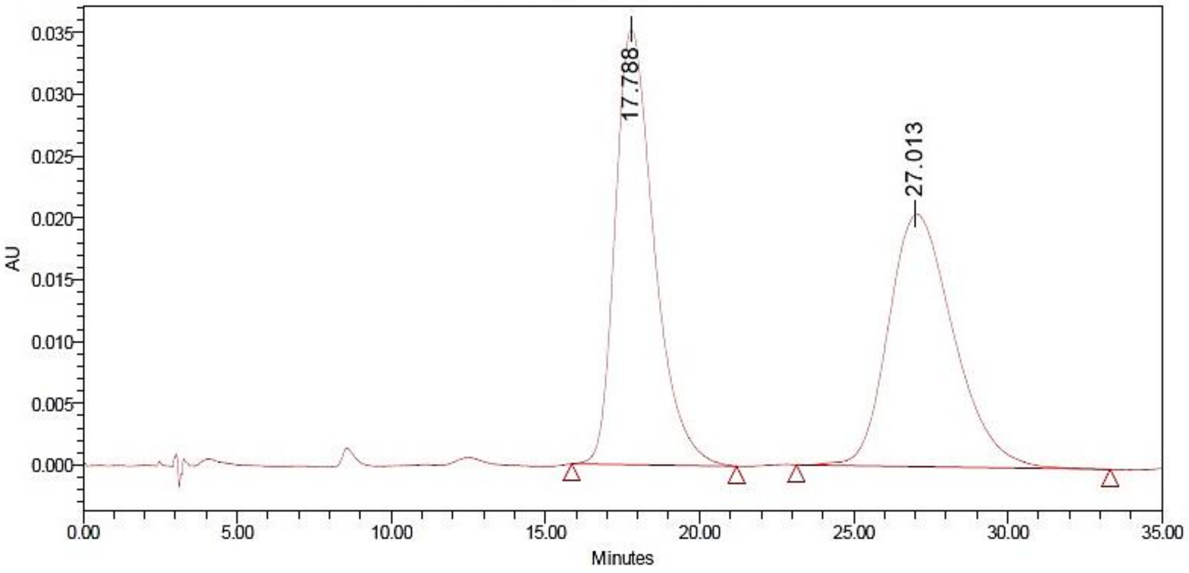

Peak Results

	Name	RT	Area	Height	Amount	Units	% Area
1		17.788	3095483	35265			50.74
2		27.013	3004850	20471			49.26

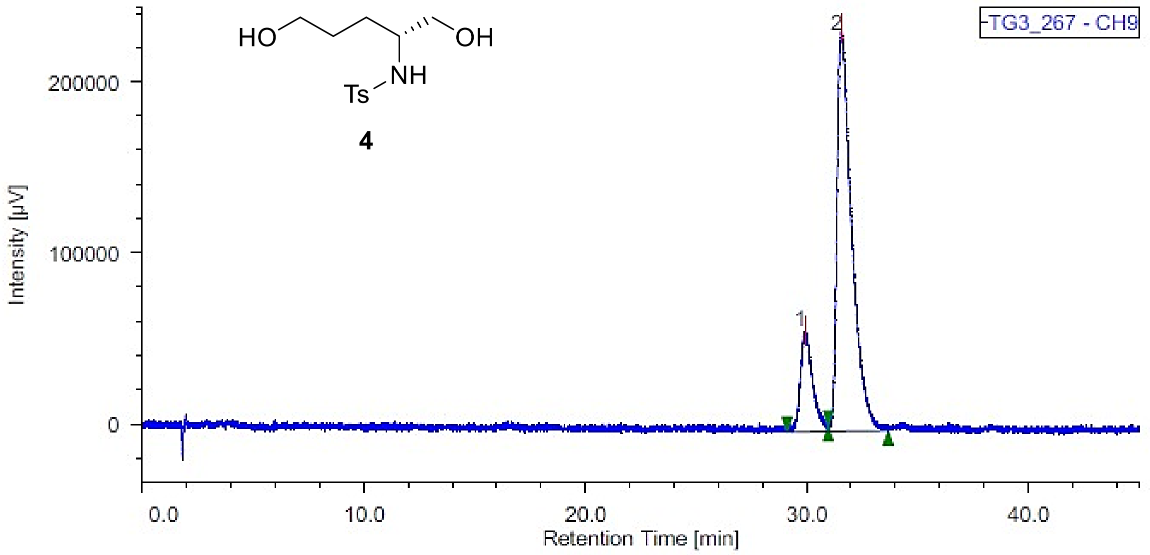

#	Peak Name	CH	tR [min]	Area [µV·sec]	Height [µV]	Area%	Height%	Quantity	NTP	Resolution	Symmetry Factor	Warning
2	Unknown	9	29.927	2281787	58835	17.368	20.012	N/A	15696	1.564	N/A	
1	Unknown	9	31.573	10855972	235165	82.632	79.988	N/A	11947	N/A	2.002	

Racemic

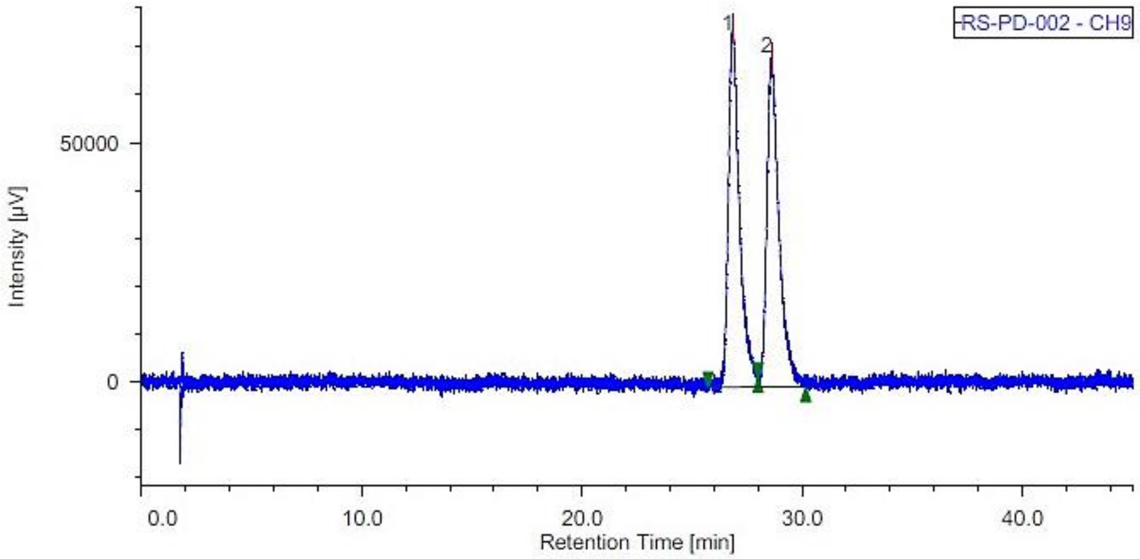

#	Peak Name	CH	tR [min]	Area [µV·sec]	Height [µV]	Area%	Height%	Quantity	NTP	Resolution	Symmetry Factor	Warning
1	Unknown	9	26.853	2664968	74966	50.021	52.174	N/A	15119	1.934	1.675	
2	Unknown	9	28.613	2662681	68717	49.979	47.826	N/A	14460	N/A	1.432	

11. ^{1}H, ^{13}C and NOESY NMR spectra

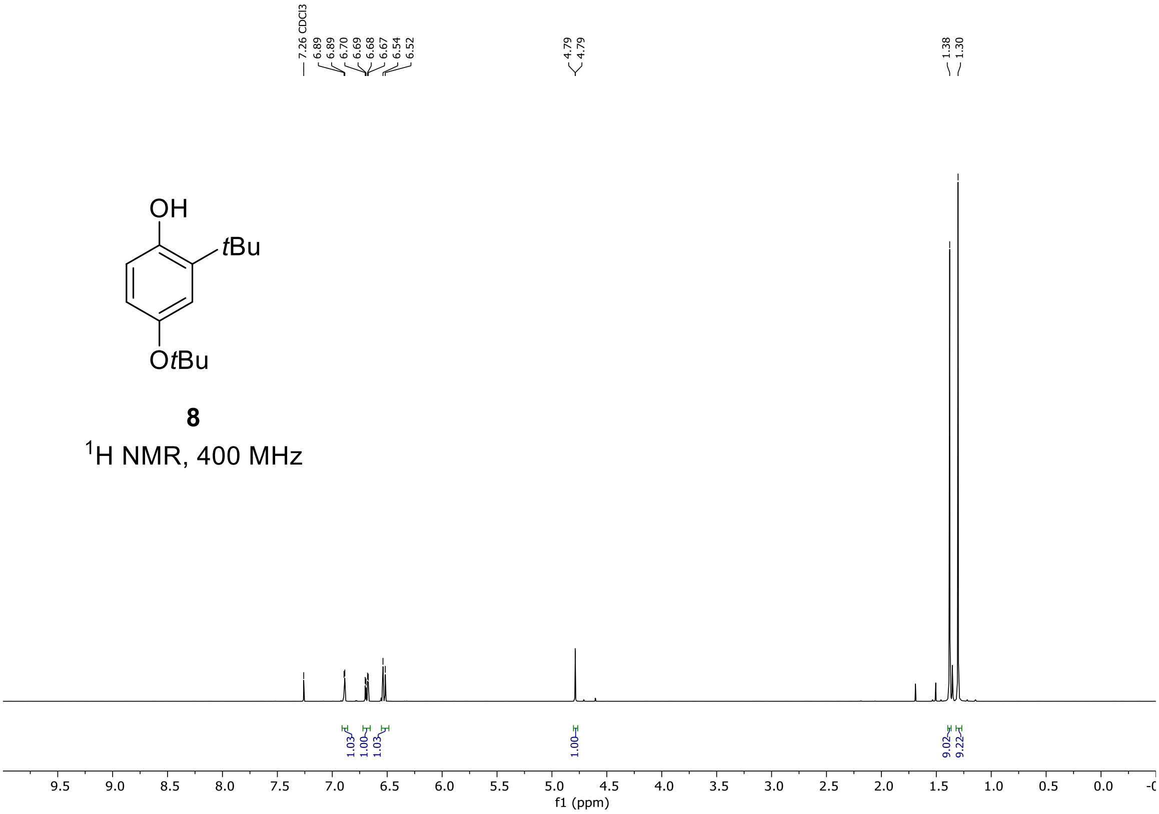

8

^{1}H NMR, 400 MHz

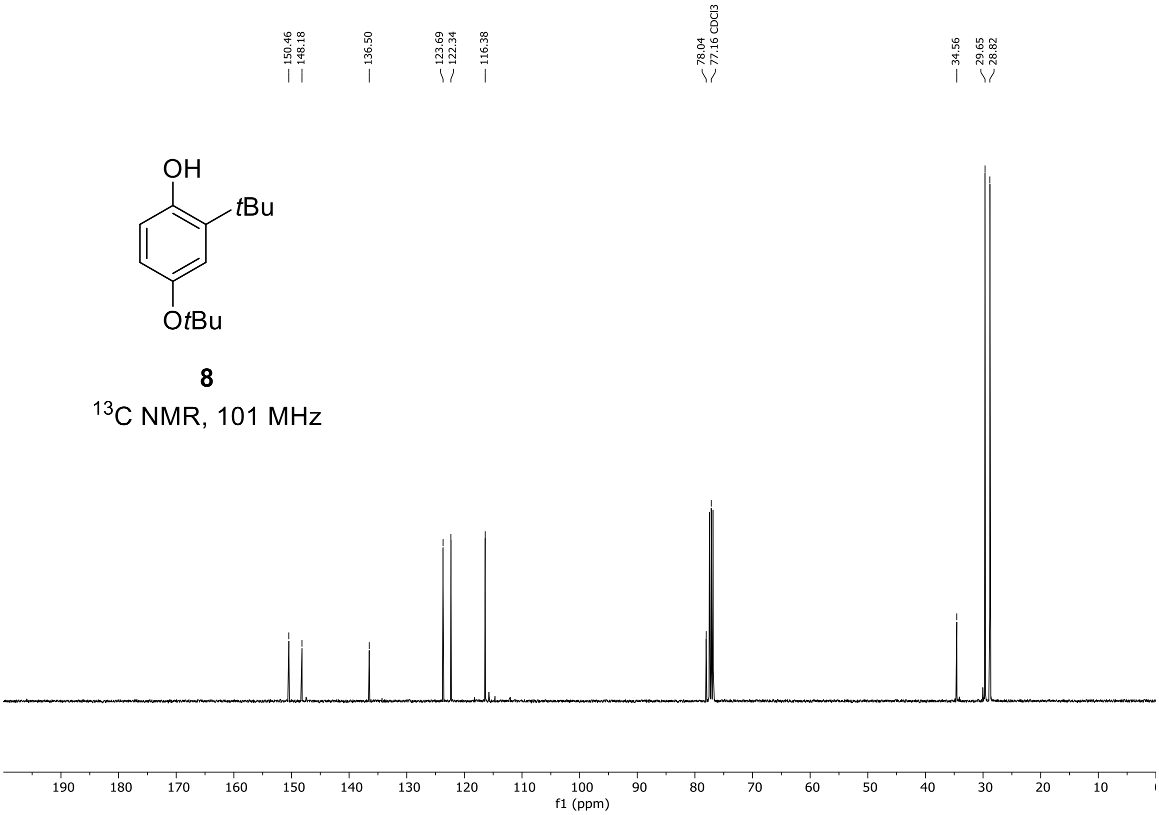

8

^{13}C NMR, 101 MHz

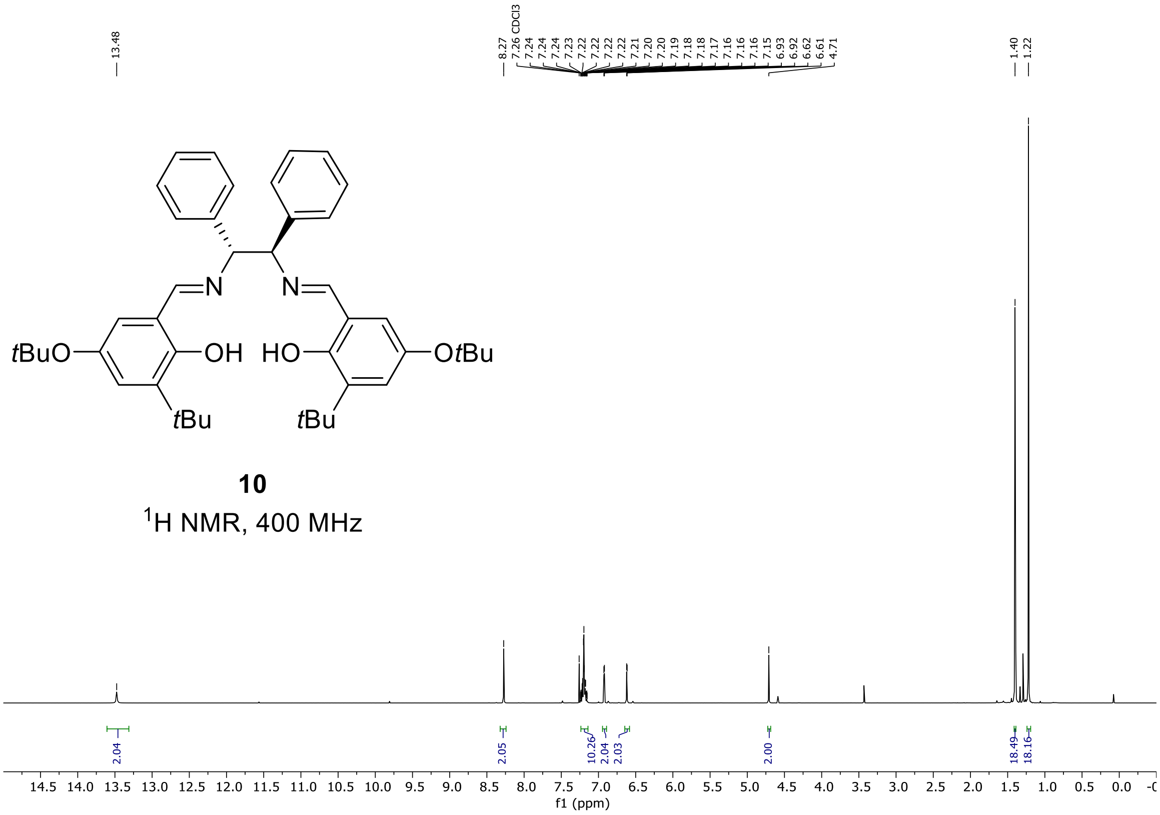
13.48
8.27
7.26 CDCl3
7.24
7.24
7.24
7.23
7.22
7.22
7.22
7.22
7.21
7.20
7.19
7.18
7.18
7.17
7.16
7.16
7.16
7.15
6.93
6.92
6.62
6.61
4.71
1.40
1.22
tBuO
OH
HO
OtBu
tBu
tBu
N
N
10
1H NMR, 400 MHz
2.04
2.05
10.26
2.04
2.03
2.00
18.49
18.16
14.5 14.0 13.5 13.0 12.5 12.0 11.5 11.0 10.5 10.0 9.5 9.0 8.5 8.0 7.5 7.0 6.5 6.0 5.5 5.0 4.5 4.0 3.5 3.0 2.5 2.0 1.5 1.0 0.5 0.0
f1 (ppm)

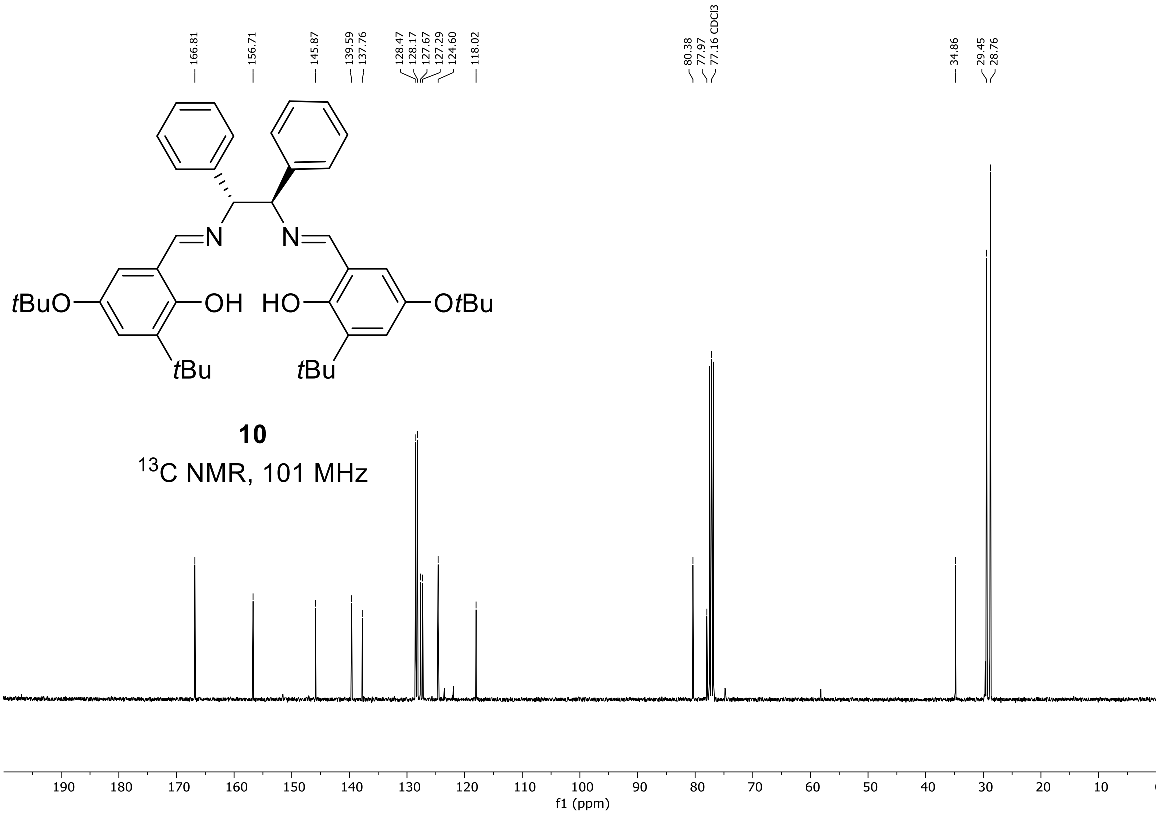
166.81
156.71
145.87
139.59
137.76
128.47
128.17
127.67
127.29
124.60
118.02
80.38
77.97
77.16 CDCl3
34.86
29.45
28.76
tBuO
OH
HO
OtBu
tBu
tBu
N
N
10
13C NMR, 101 MHz
190 180 170 160 150 140 130 120 110 100 90 80 70 60 50 40 30 20 10
f1 (ppm)

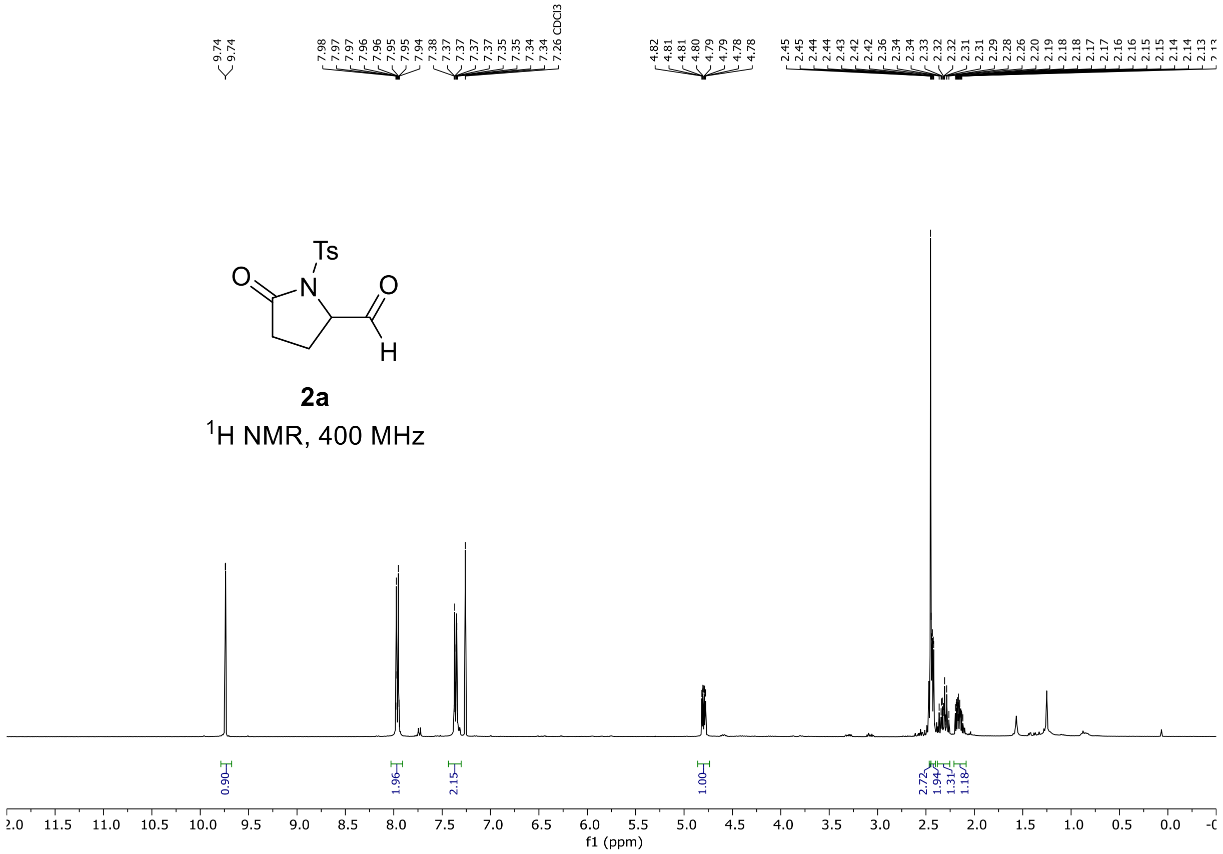
Ts
O
N
O
H
2a
^{1}H NMR, 400 MHz
f1 (ppm)

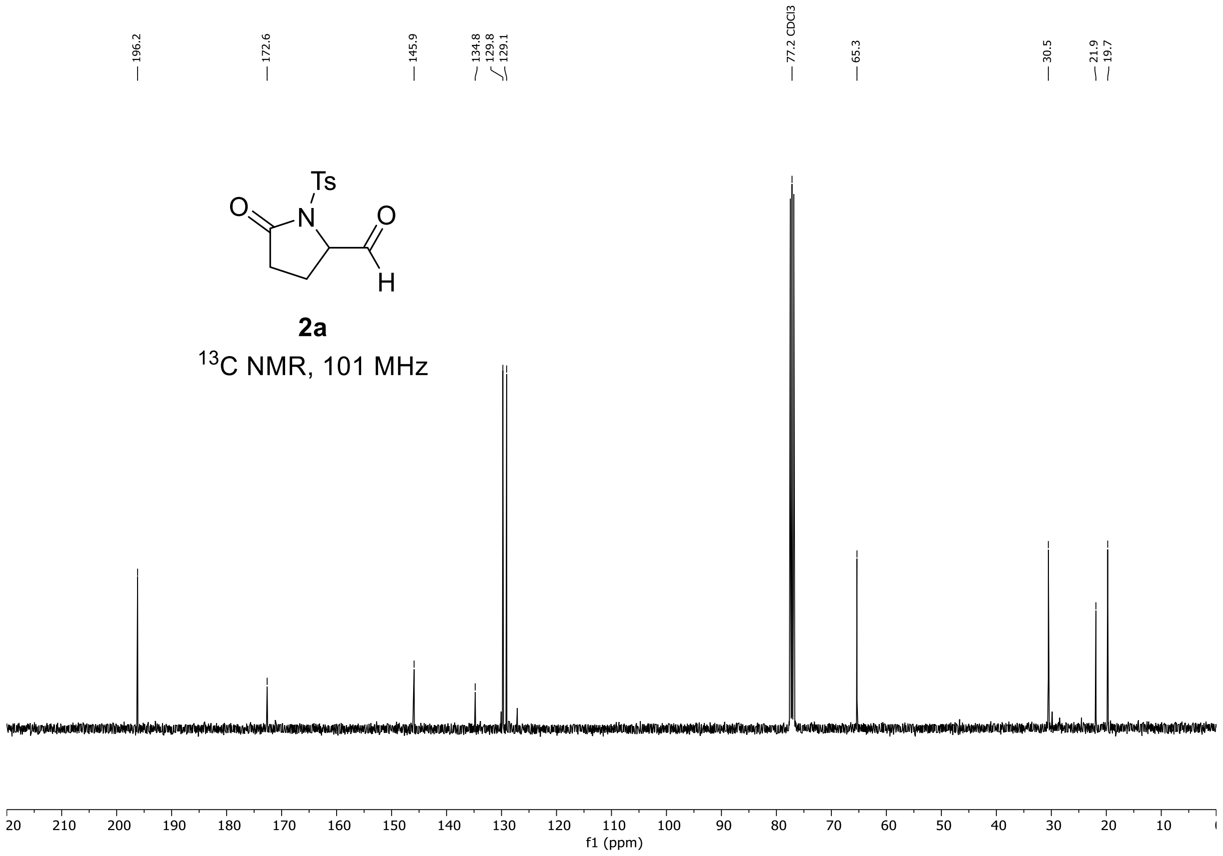
Ts
O
N
O
H
2a
^{13}C NMR, 101 MHz
f1 (ppm)

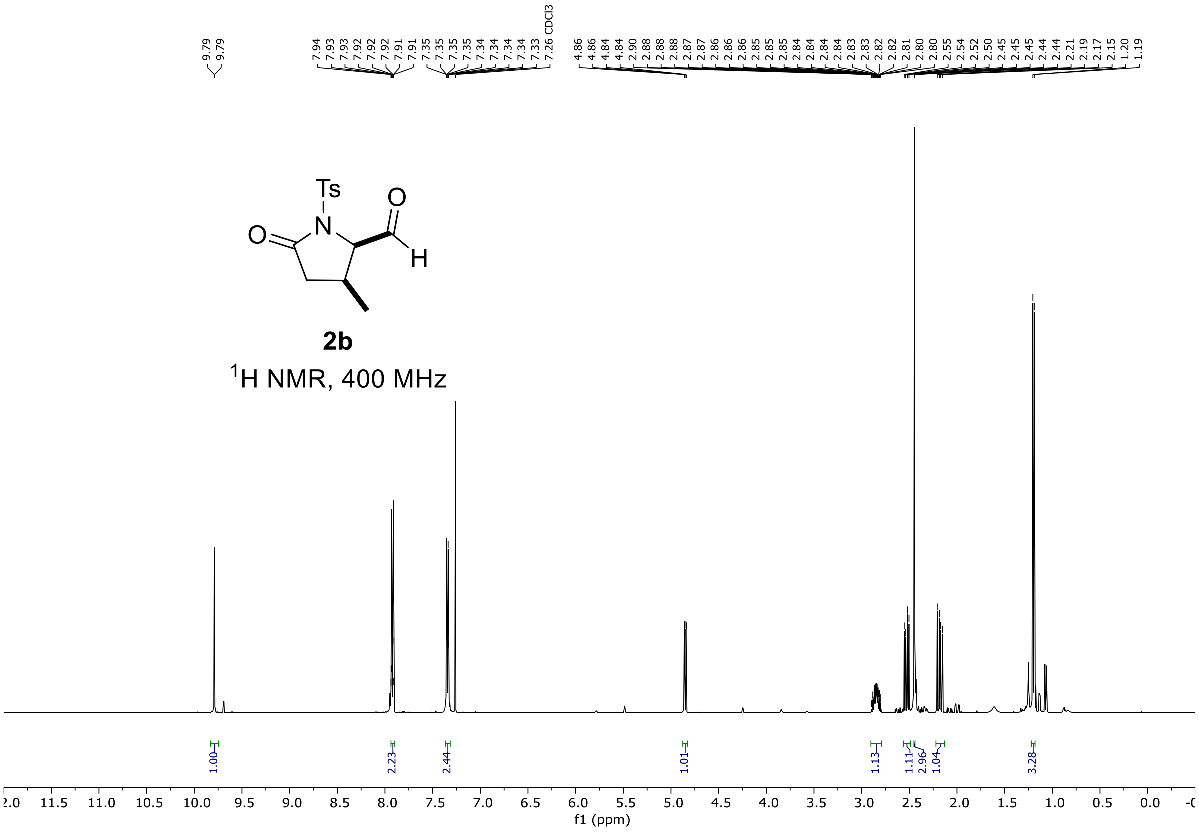

2b

^{1}H NMR, 400 MHz

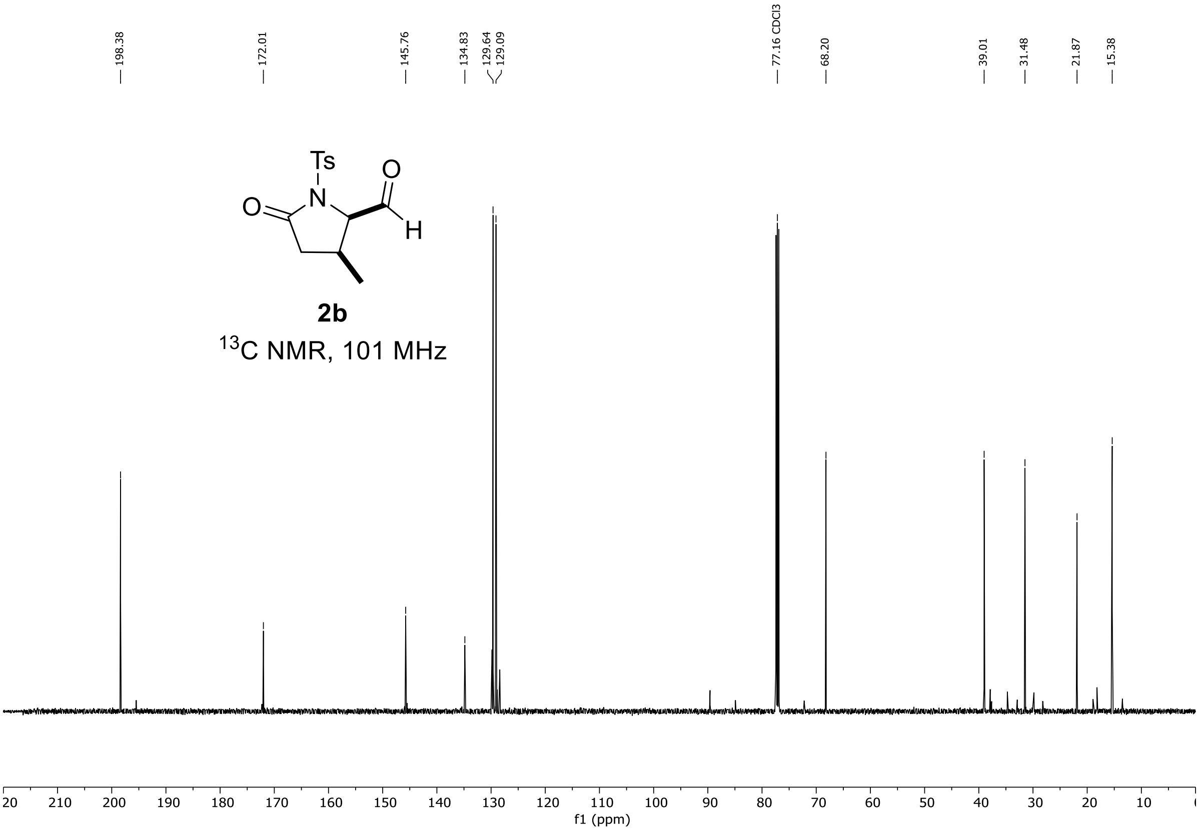

2b

^{13}C NMR, 101 MHz

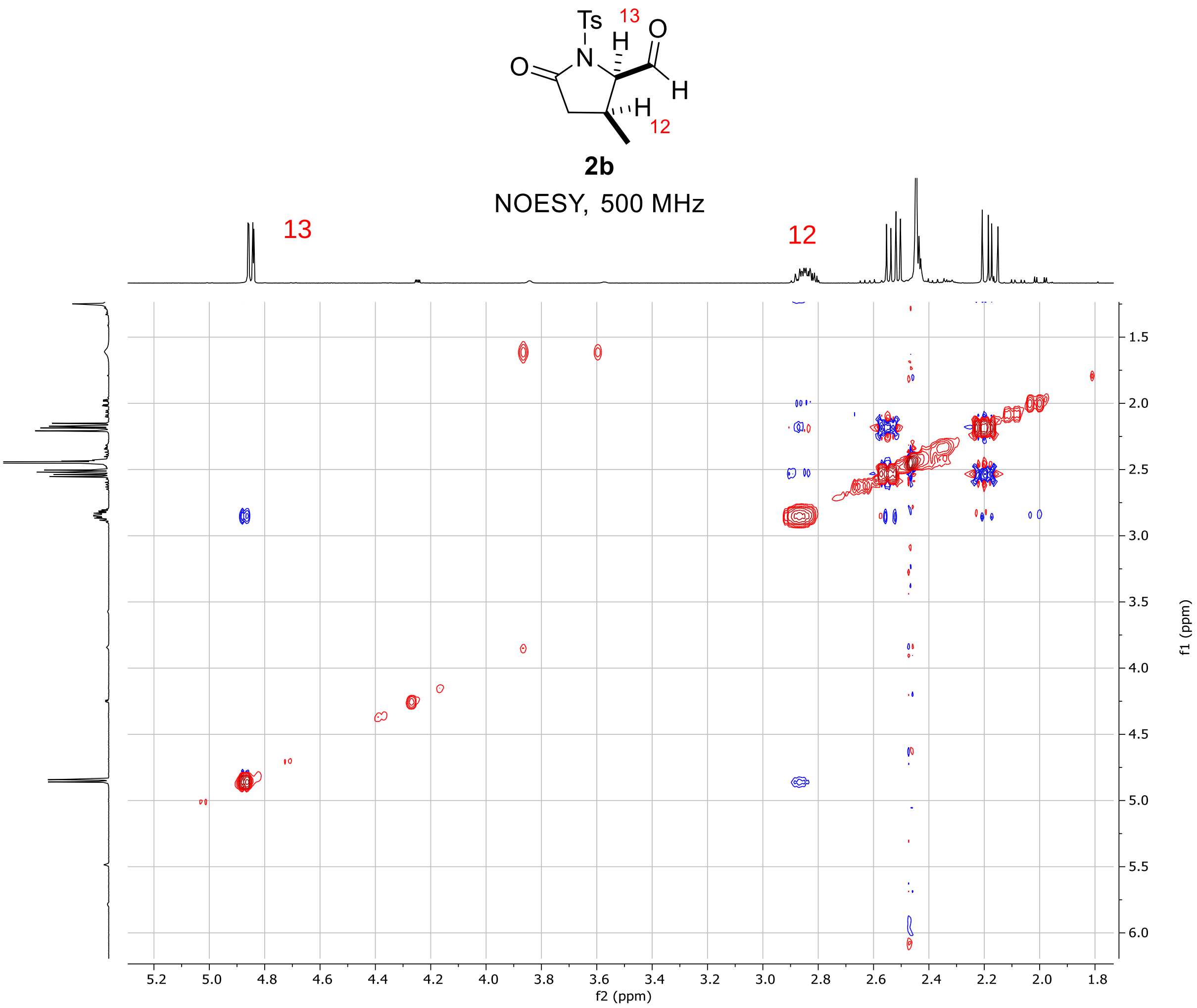
Ts
13
H
O
O
N
H
H
12
2b
NOESY, 500 MHz
13
12
f2 (ppm)
f1 (ppm)

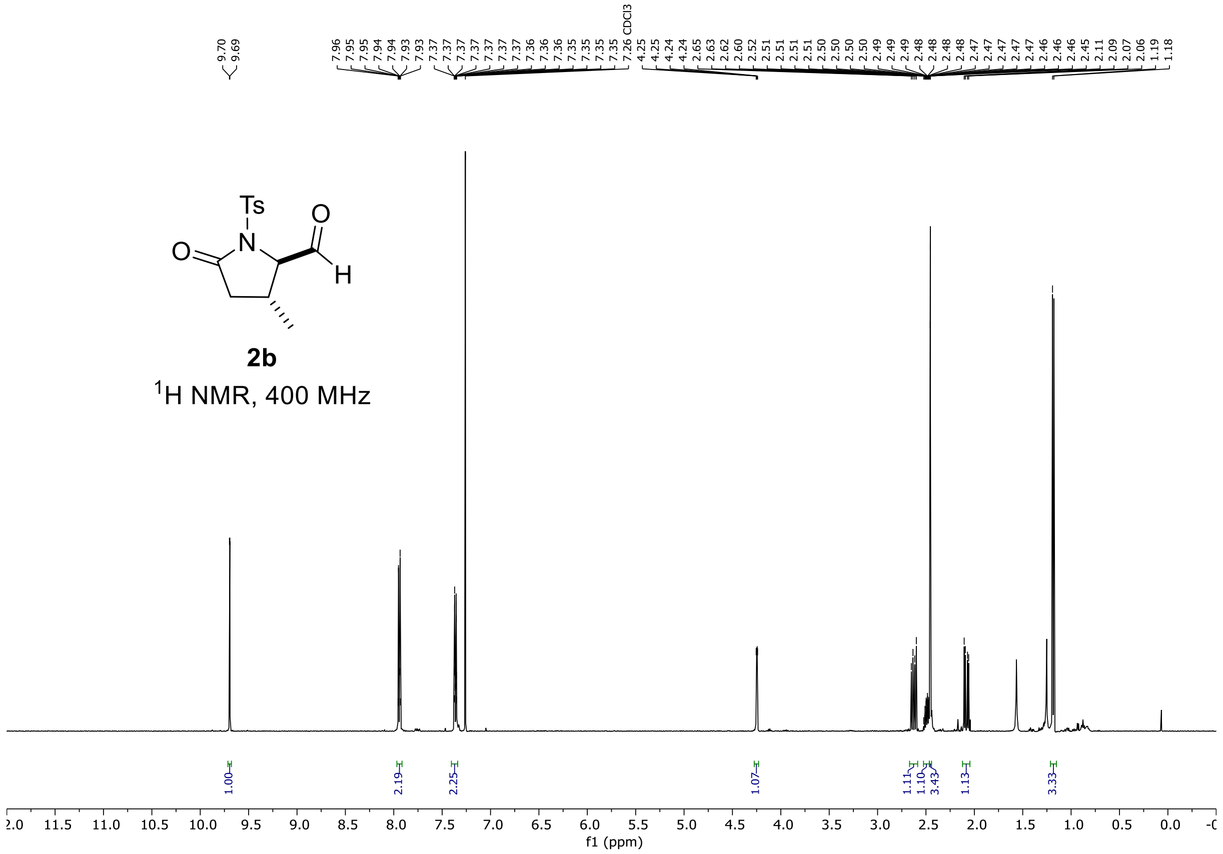

2b

^{1}H NMR, 400 MHz

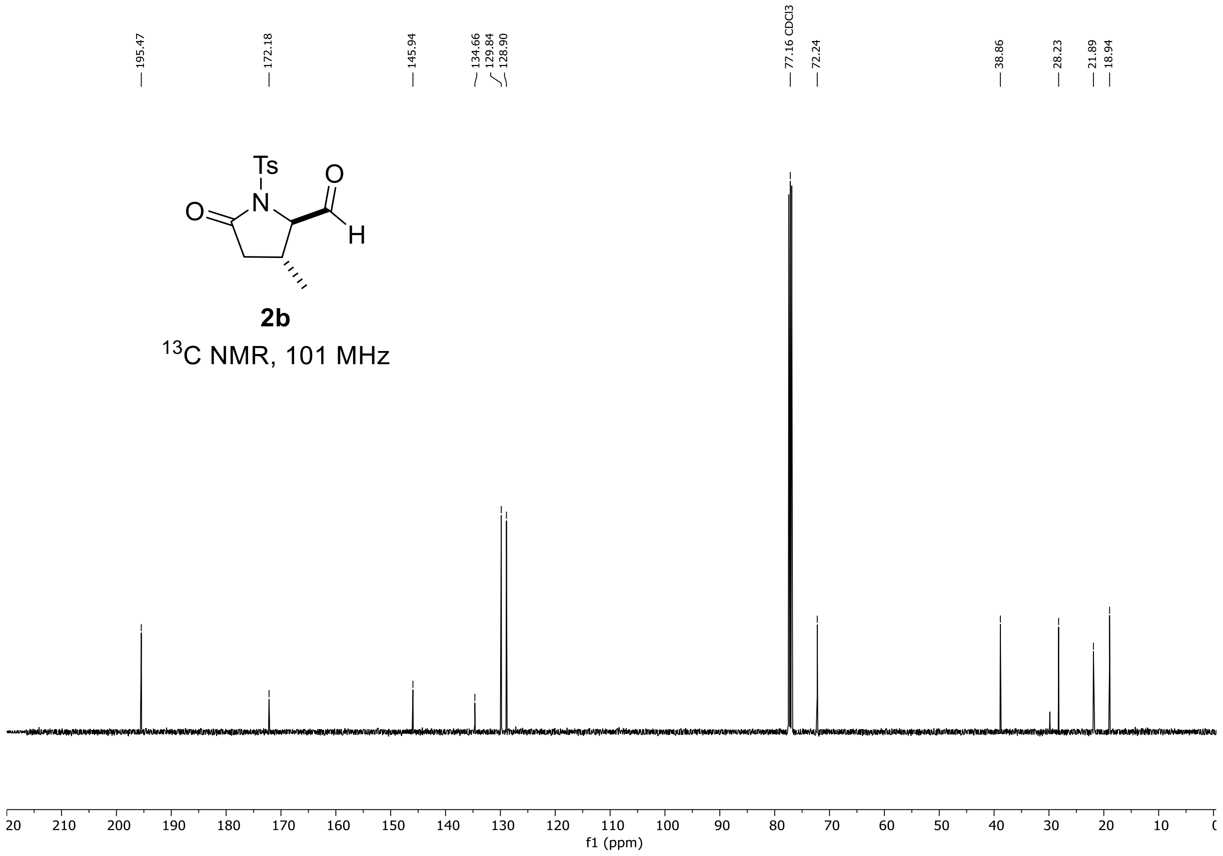

2b

^{13}C NMR, 101 MHz

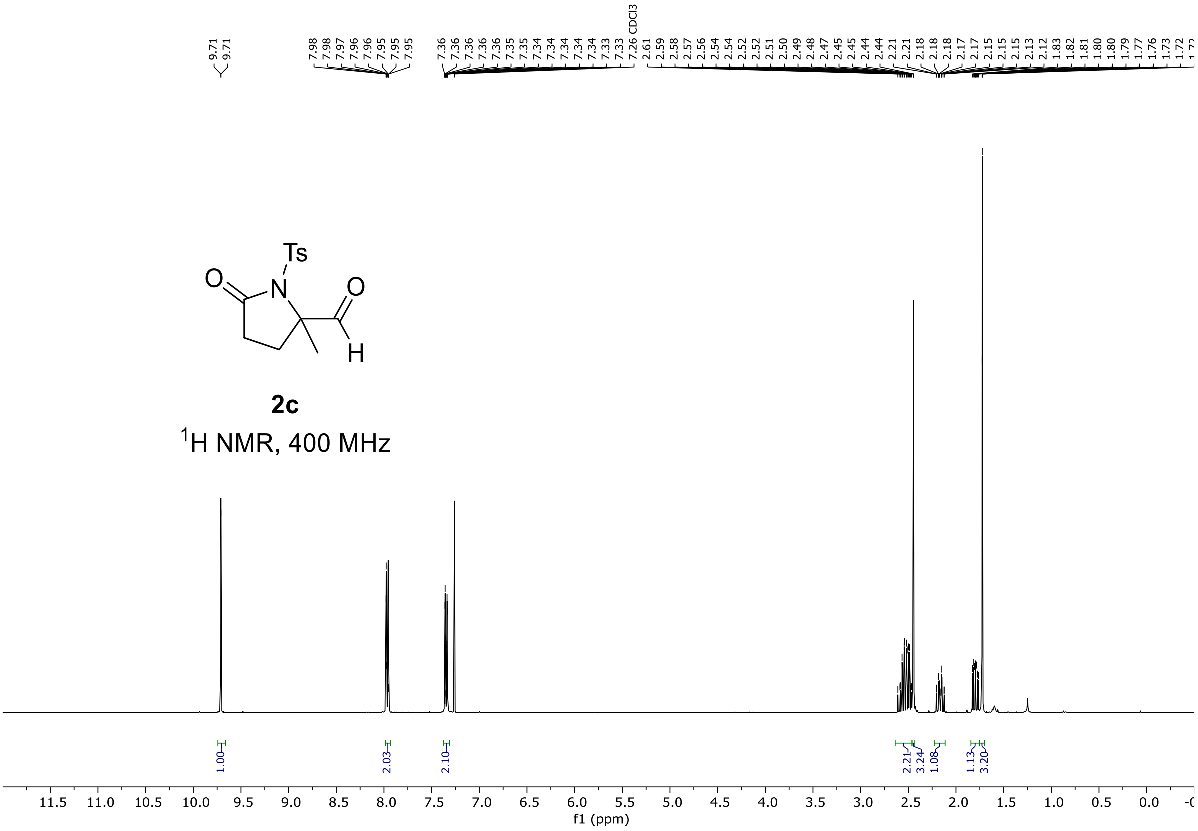
9.71
9.71
7.98
7.98
7.97
7.96
7.96
7.95
7.95
7.95
7.36
7.36
7.36
7.36
7.36
7.35
7.35
7.34
7.34
7.34
7.34
7.34
7.33
7.33
7.26 CDCl3
2.61
2.59
2.58
2.57
2.56
2.54
2.54
2.52
2.52
2.51
2.50
2.49
2.48
2.47
2.45
2.45
2.44
2.44
2.21
2.21
2.18
2.18
2.18
2.17
2.17
2.15
2.15
2.15
2.13
2.12
1.83
1.82
1.81
1.80
1.79
1.77
1.76
1.73
1.72
[illegible]
Ts
O
N
O
H
2c
^{1}H NMR, 400 MHz
1.00
2.03
2.10
2.21
3.24
1.08
1.13
3.20
f1 (ppm)

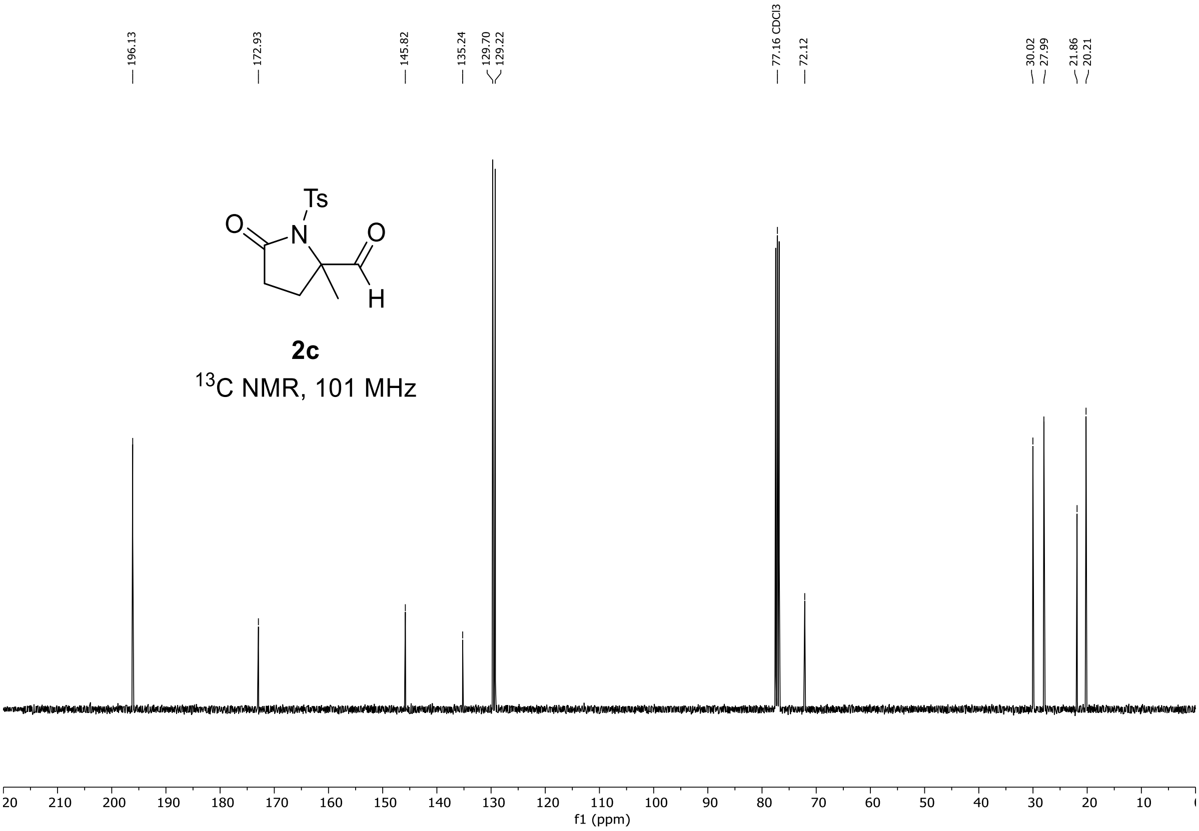
196.13
172.93
145.82
135.24
129.70
129.22
77.16 CDCl3
72.12
30.02
27.99
21.86
20.21
Ts
O
N
O
H
2c
^{13}C NMR, 101 MHz
f1 (ppm)

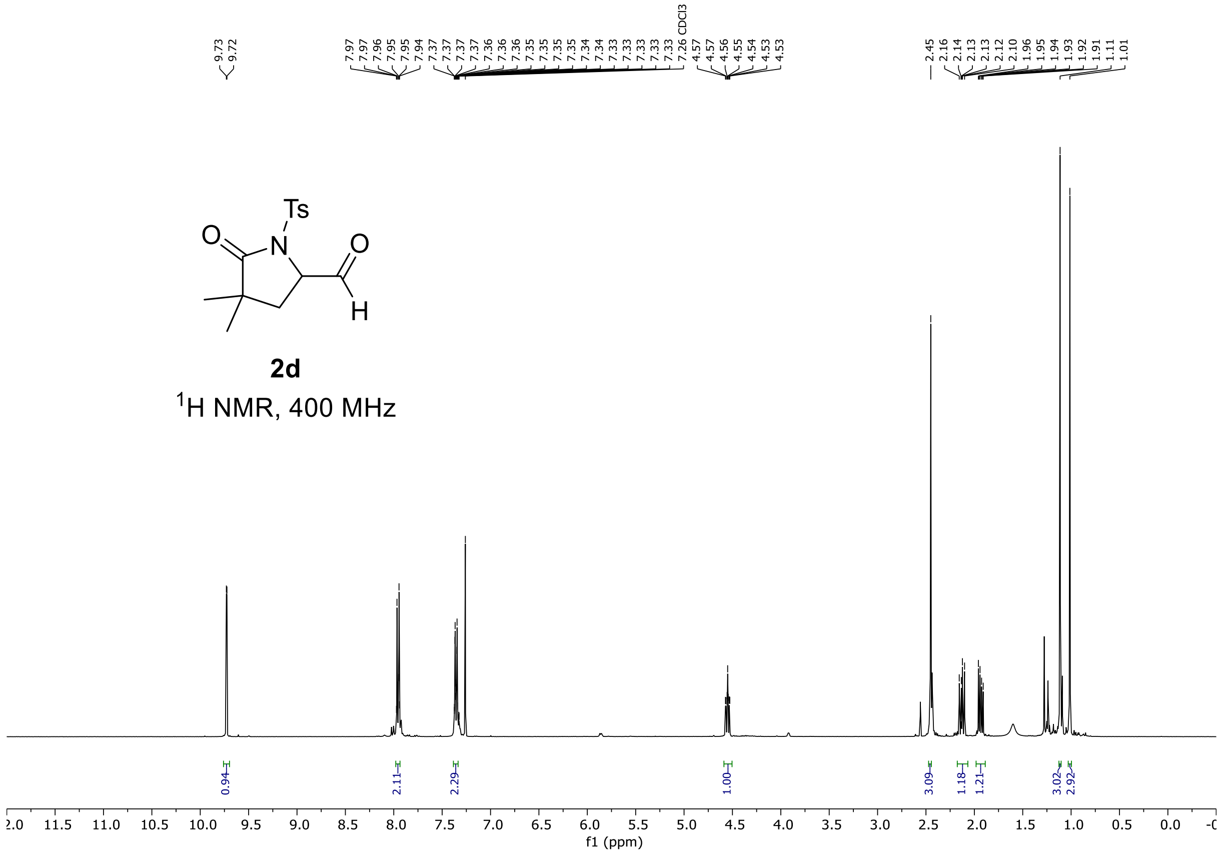
9.73
9.72
7.97
7.97
7.96
7.95
7.95
7.94
7.37
7.37
7.37
7.37
7.36
7.36
7.35
7.35
7.35
7.35
7.34
7.34
7.33
7.33
7.33
7.33
7.33
7.33
7.26 CDCl3
4.57
4.57
4.56
4.55
4.54
4.53
4.53
2.45
2.16
2.14
2.13
2.13
2.12
2.10
1.96
1.95
1.94
1.93
1.92
1.91
1.11
1.01
Ts
O
N
O
H
2d
1H NMR, 400 MHz
0.94
2.11
2.29
1.00
3.09
1.18
1.21
3.02
2.92
f1 (ppm)

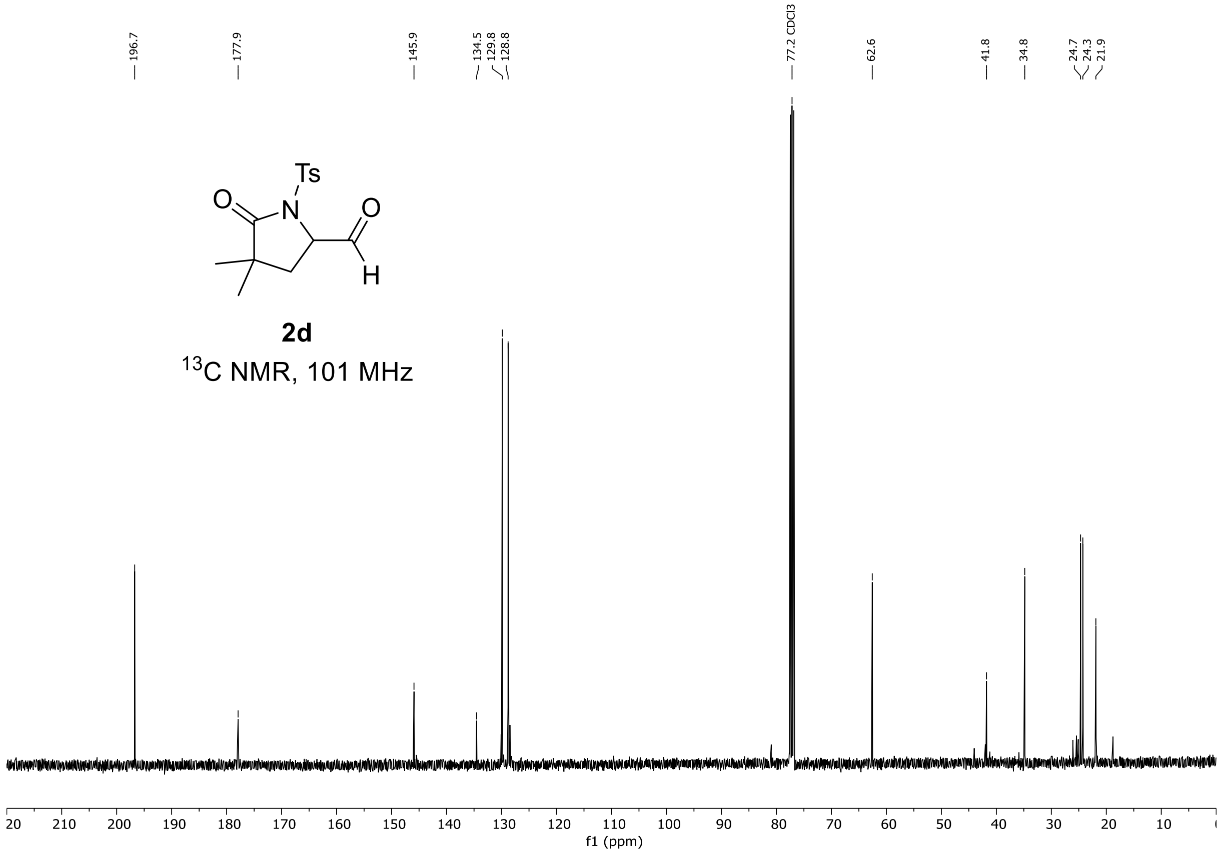
196.7
177.9
145.9
134.5
129.8
128.8
77.2 CDCl3
62.6
41.8
34.8
24.7
24.3
21.9
Ts
O
N
O
H
2d
13C NMR, 101 MHz
f1 (ppm)

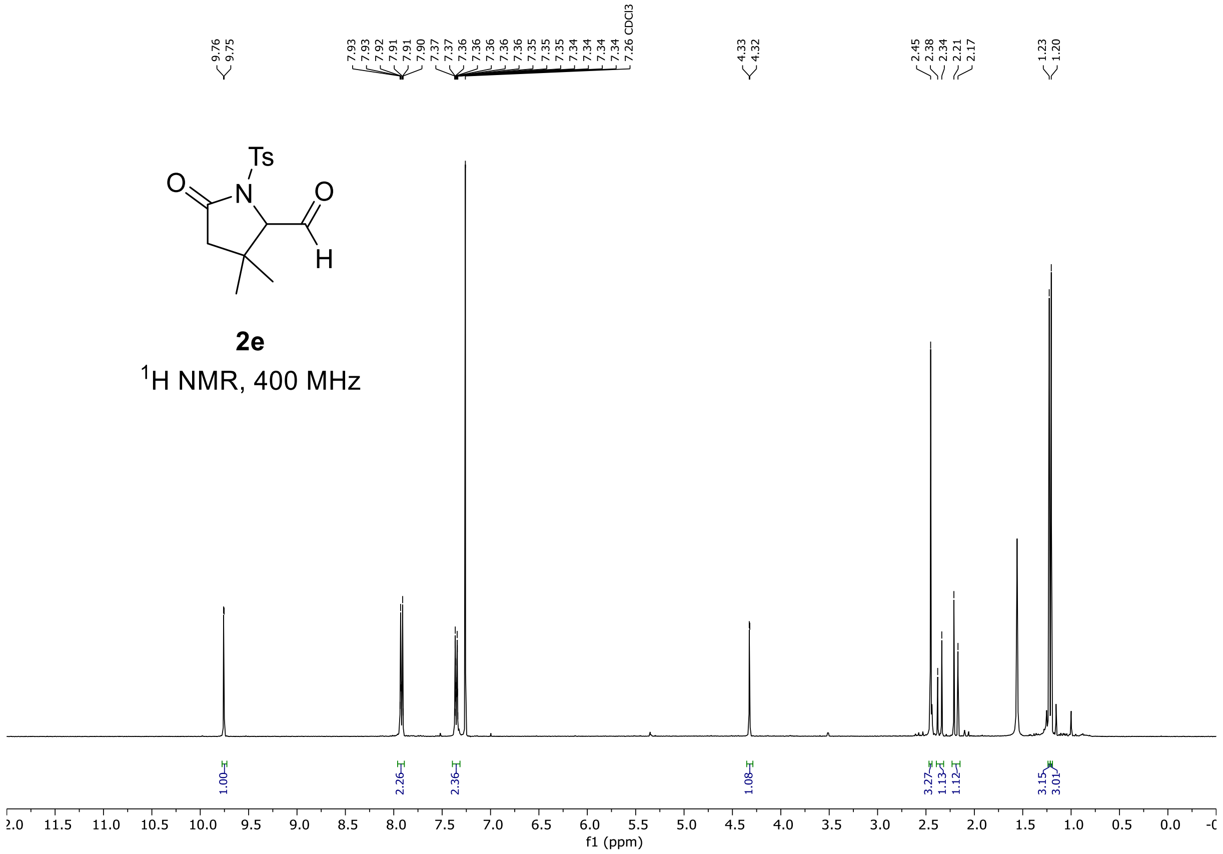
Ts
O
N
O
H
2e
^{1}H NMR, 400 MHz
f1 (ppm)

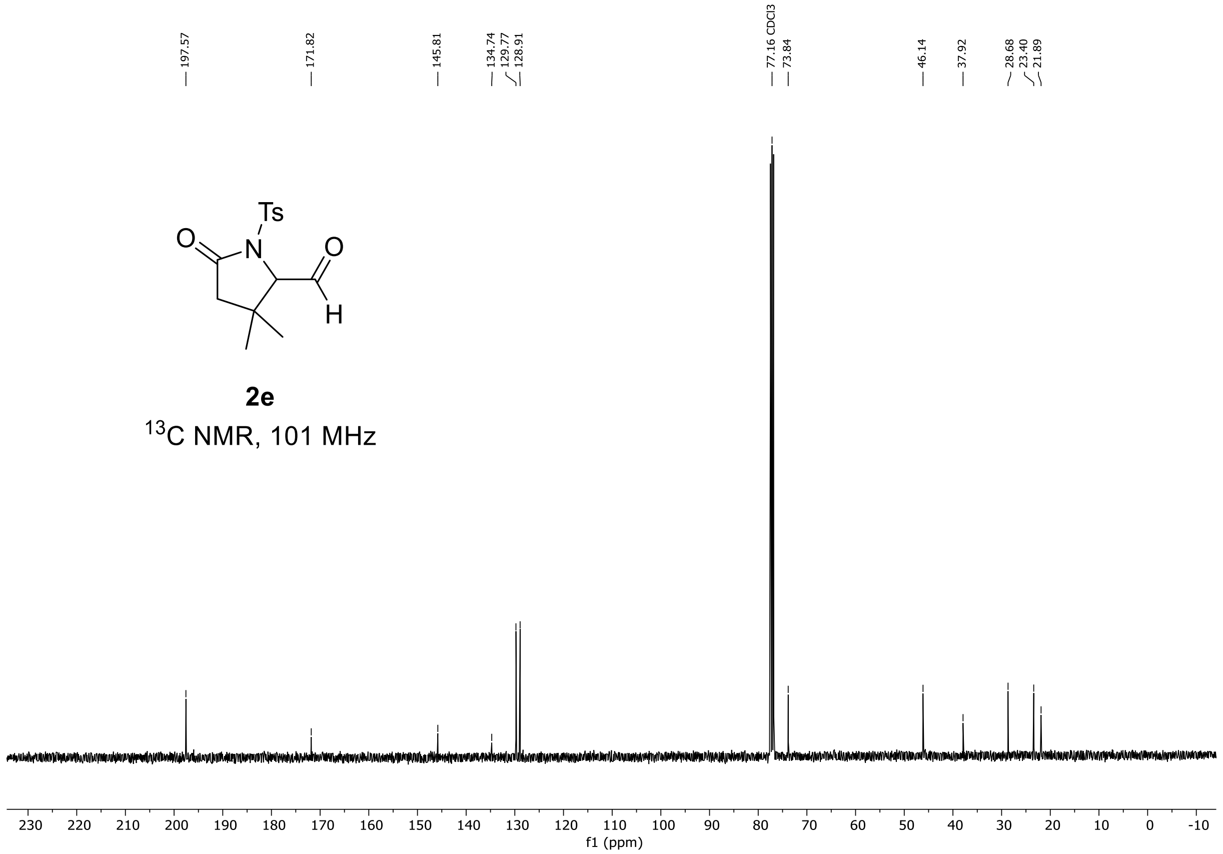
Ts
O
N
O
H
2e
^{13}C NMR, 101 MHz
f1 (ppm)

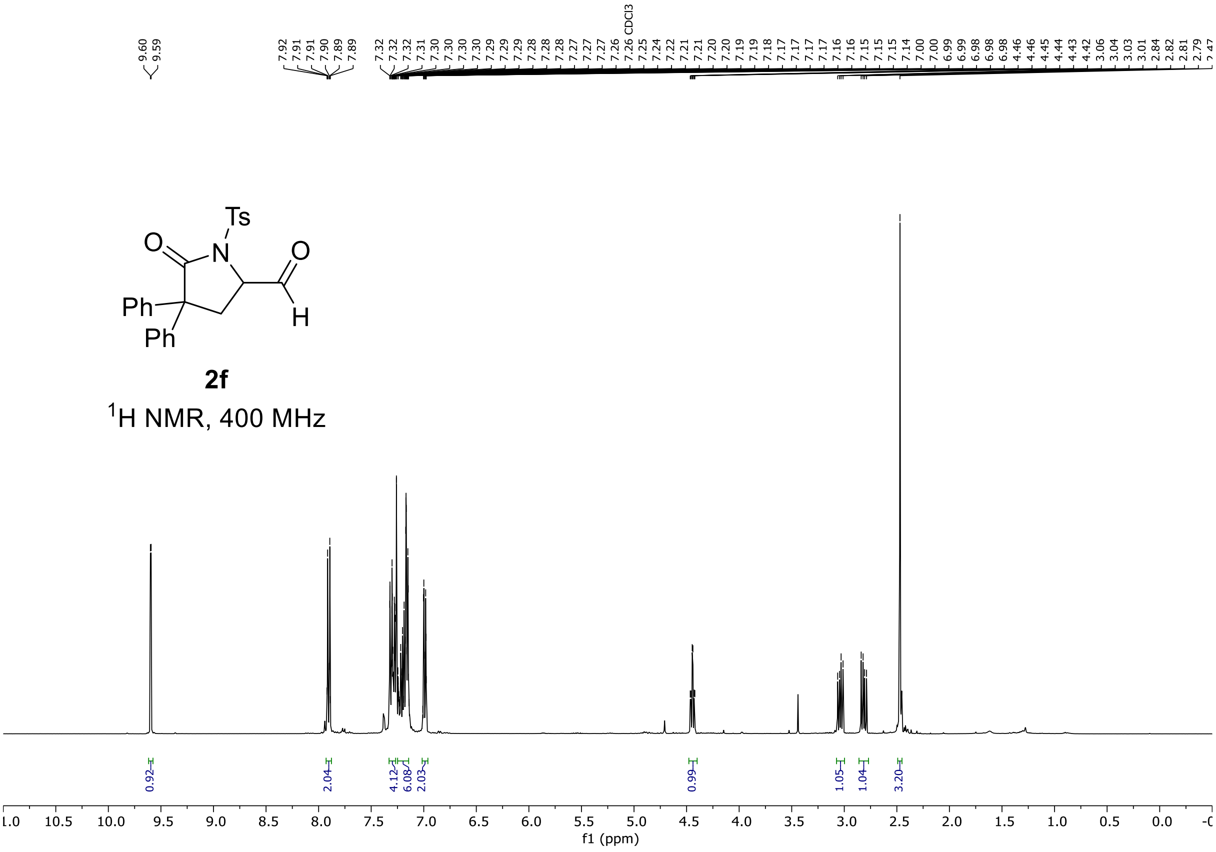

Ts
O
N
O
Ph
Ph
H
2f
^{1}H NMR, 400 MHz
f1 (ppm)

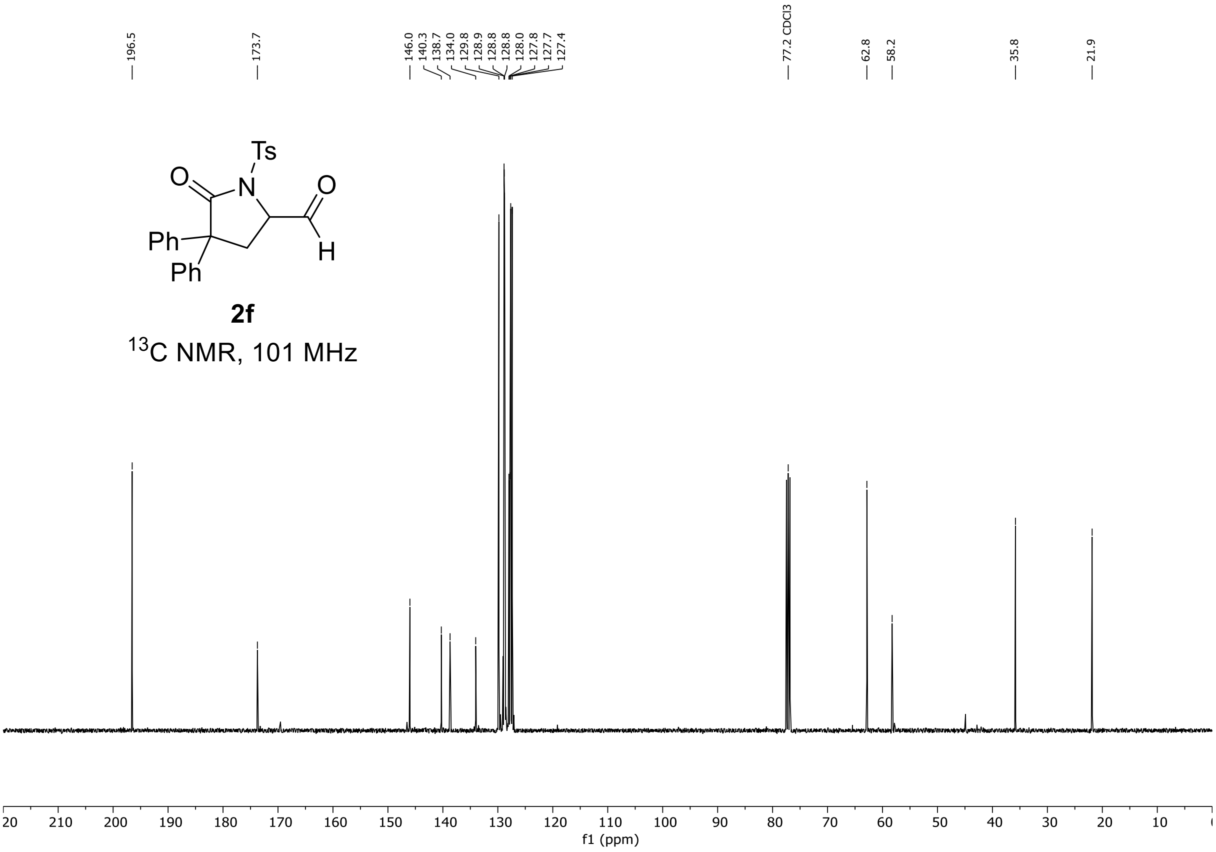

196.5
173.7
146.0
140.3
138.7
134.0
129.8
128.9
128.8
128.8
128.0
127.8
127.7
127.4
77.2 CDCl3
62.8
58.2
35.8
21.9
Ts
O
N
O
Ph
Ph
H
2f
^{13}C NMR, 101 MHz
f1 (ppm)

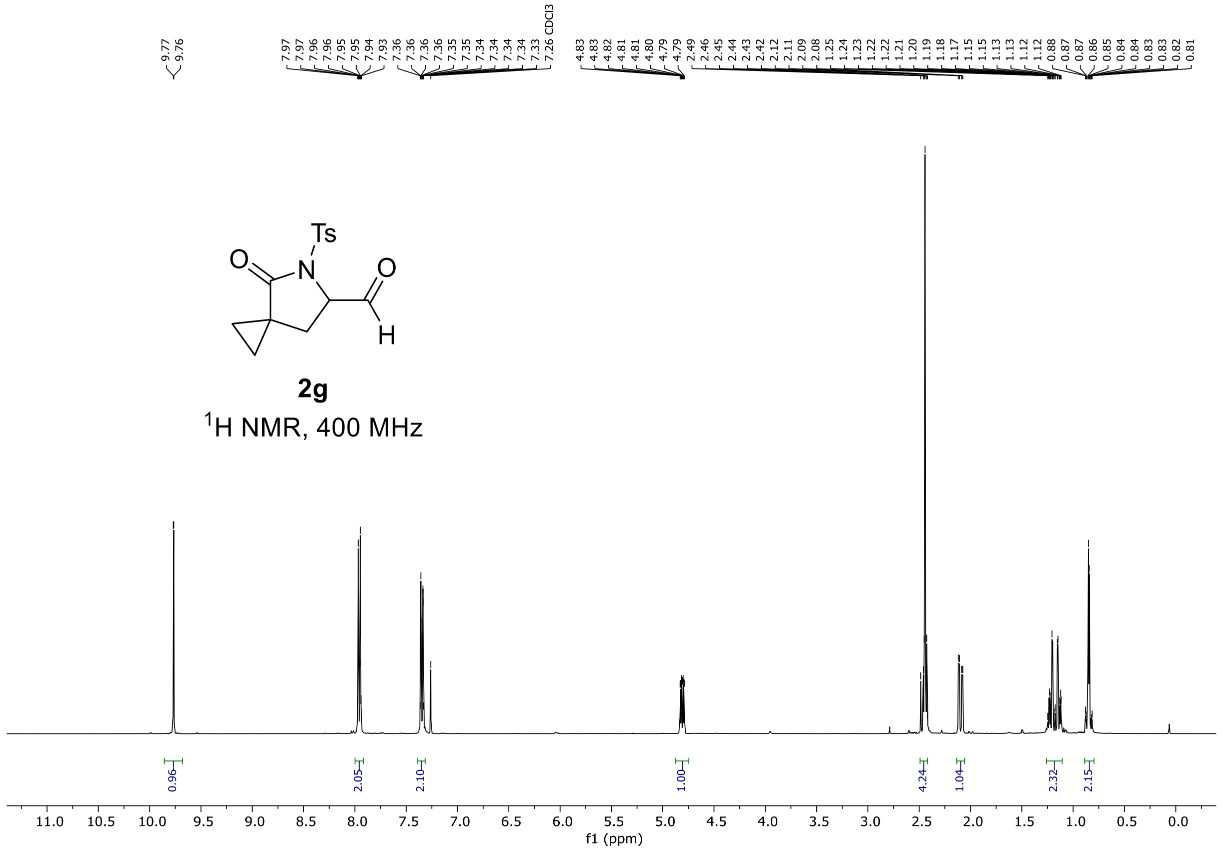

9.77
9.76
7.97
7.97
7.96
7.95
7.95
7.94
7.93
7.36
7.36
7.36
7.35
7.35
7.34
7.34
7.34
7.34
7.33
7.26 CDCl3
4.83
4.83
4.82
4.81
4.81
4.80
4.79
2.49
2.46
2.45
2.44
2.43
2.42
2.12
2.11
2.09
2.08
1.25
1.24
1.23
1.22
1.22
1.21
1.20
1.19
1.18
1.17
1.15
1.15
1.13
1.13
1.12
1.12
0.88
0.87
0.86
0.85
0.84
0.84
0.83
0.83
0.82
0.81
Ts
O
N
O
H
2g
1H NMR, 400 MHz
0.96
2.05
2.10
1.00
4.24
1.04
2.32
2.15
f1 (ppm)

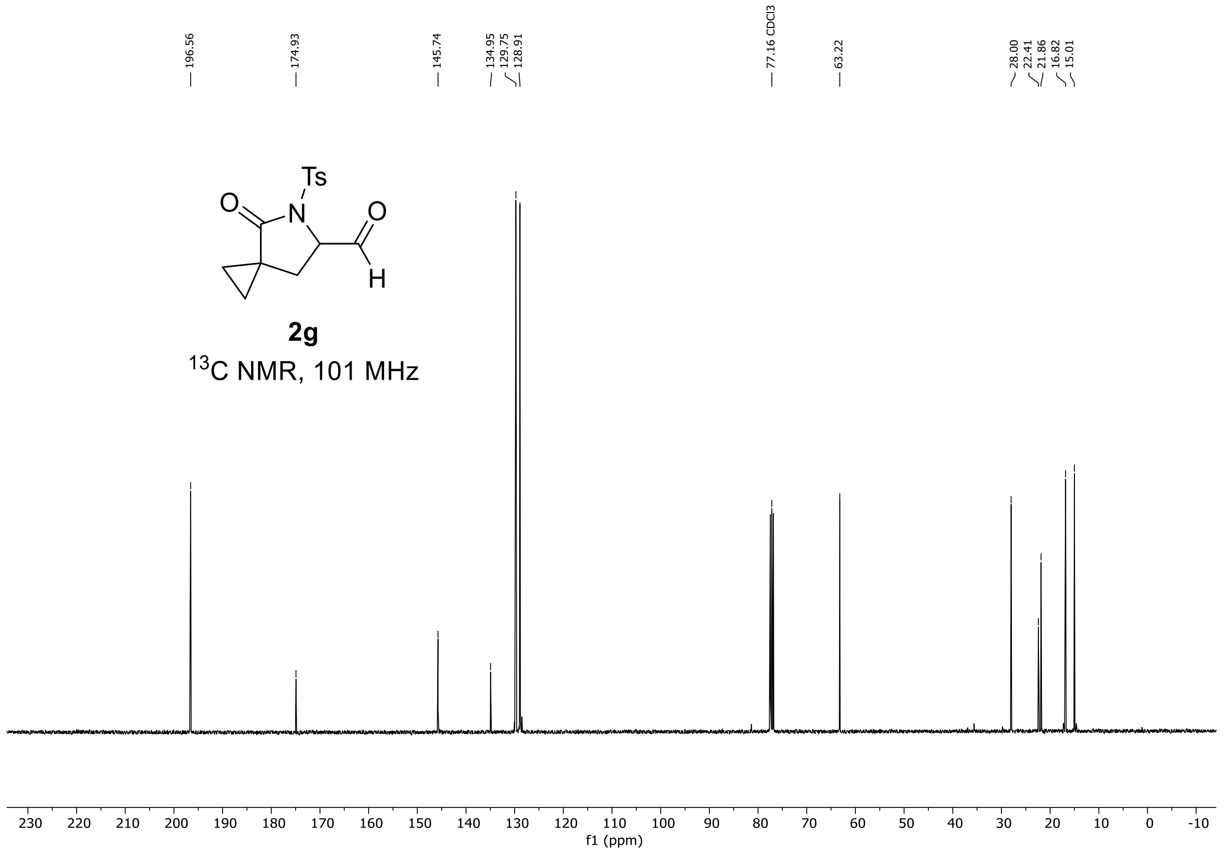

196.56
174.93
145.74
134.95
129.75
128.91
77.16 CDCl3
63.22
28.00
22.41
21.86
16.82
15.01
Ts
O
N
O
H
2g
13C NMR, 101 MHz
f1 (ppm)

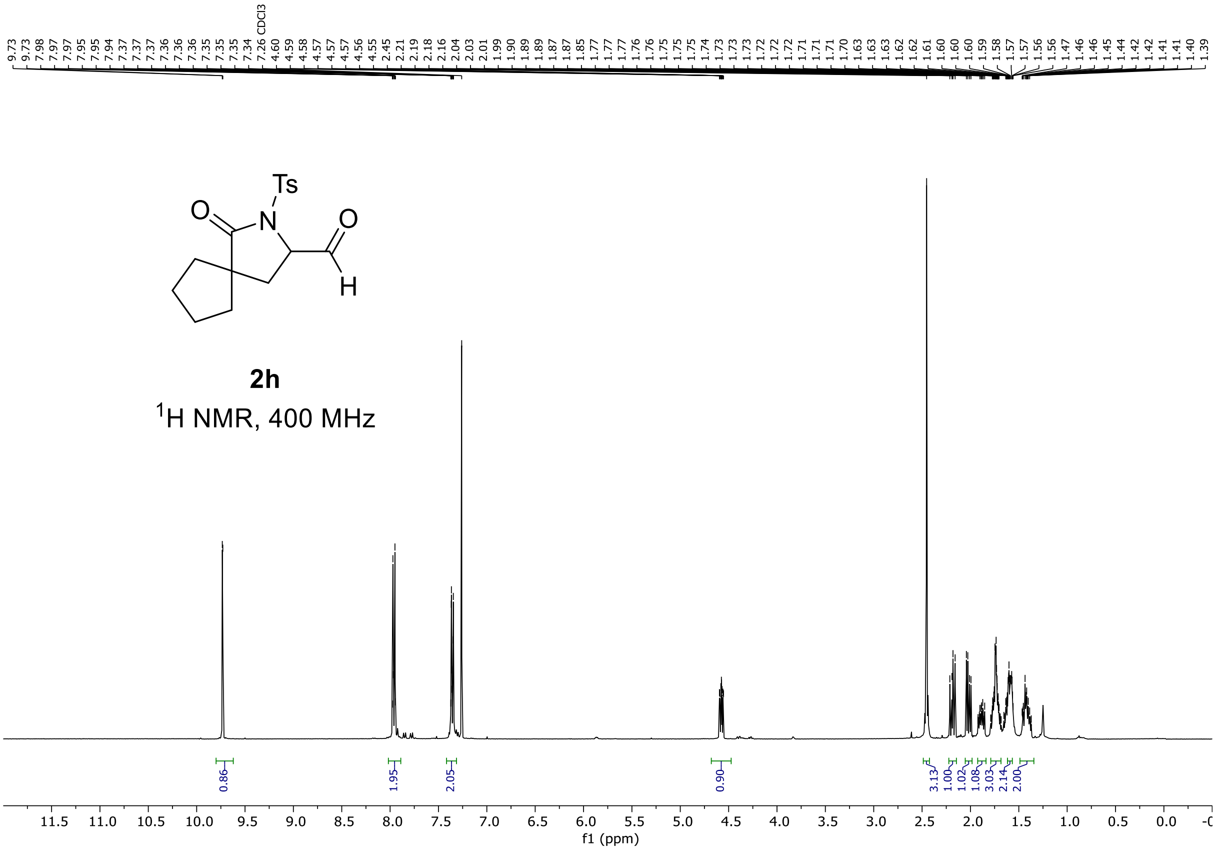
9.73 9.73 7.98 7.97 7.97 7.95 7.95 7.94 7.37 7.37 7.36 7.36 7.35 7.35 7.34 7.26 CDCl3 4.60 4.59 4.58 4.57 4.57 4.57 4.56 4.55 2.45 2.21 2.19 2.18 2.16 2.04 2.03 2.01 1.99 1.90 1.89 1.89 1.87 1.87 1.85 1.77 1.77 1.77 1.76 1.75 1.75 1.75 1.74 1.73 1.73 1.73 1.72 1.72 1.72 1.71 1.71 1.71 1.70 1.63 1.63 1.63 1.62 1.62 1.61 1.60 1.60 1.60 1.59 1.58 1.57 1.57 1.56 1.56 1.47 1.46 1.46 1.45 1.44 1.42 1.42 1.41 1.41 1.40 1.39
Ts
O
N
O
H
2h
1H NMR, 400 MHz
0.86
1.95
2.05
0.90
3.13
1.00
1.02
1.08
3.03
2.14
2.00
f1 (ppm)

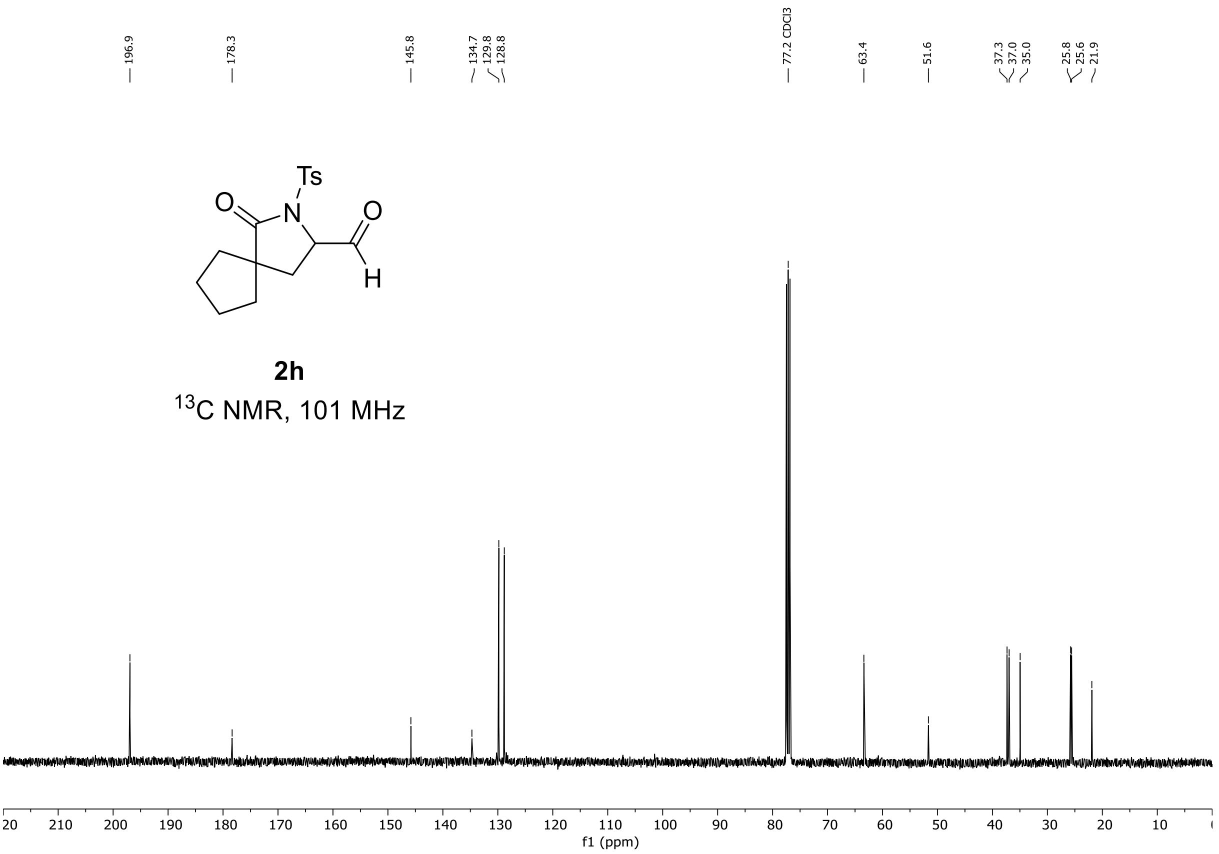
196.9
178.3
145.8
134.7
129.8
128.8
77.2 CDCl3
63.4
51.6
37.3
37.0
35.0
25.8
25.6
21.9
Ts
O
N
O
H
2h
13C NMR, 101 MHz
f1 (ppm)

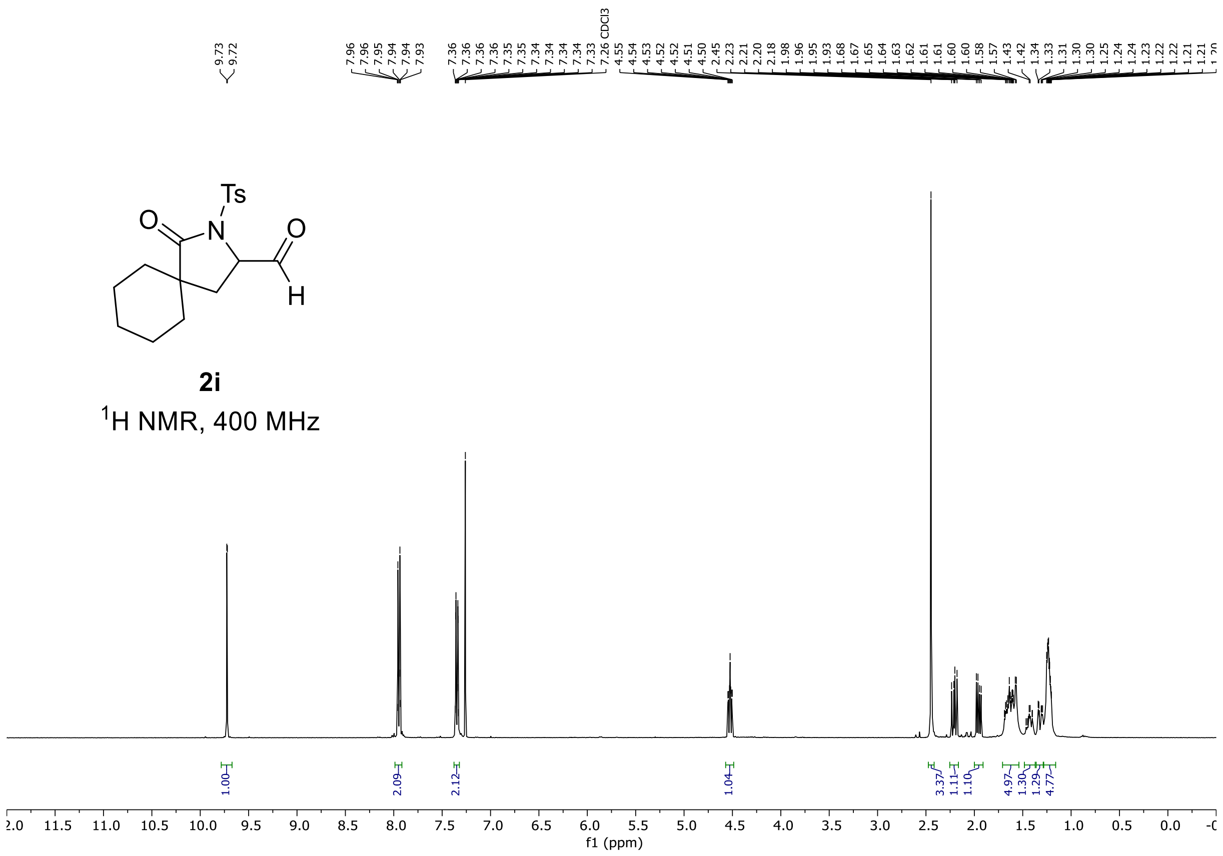

Ts
O
N
O
H
2i
¹H NMR, 400 MHz
f1 (ppm)

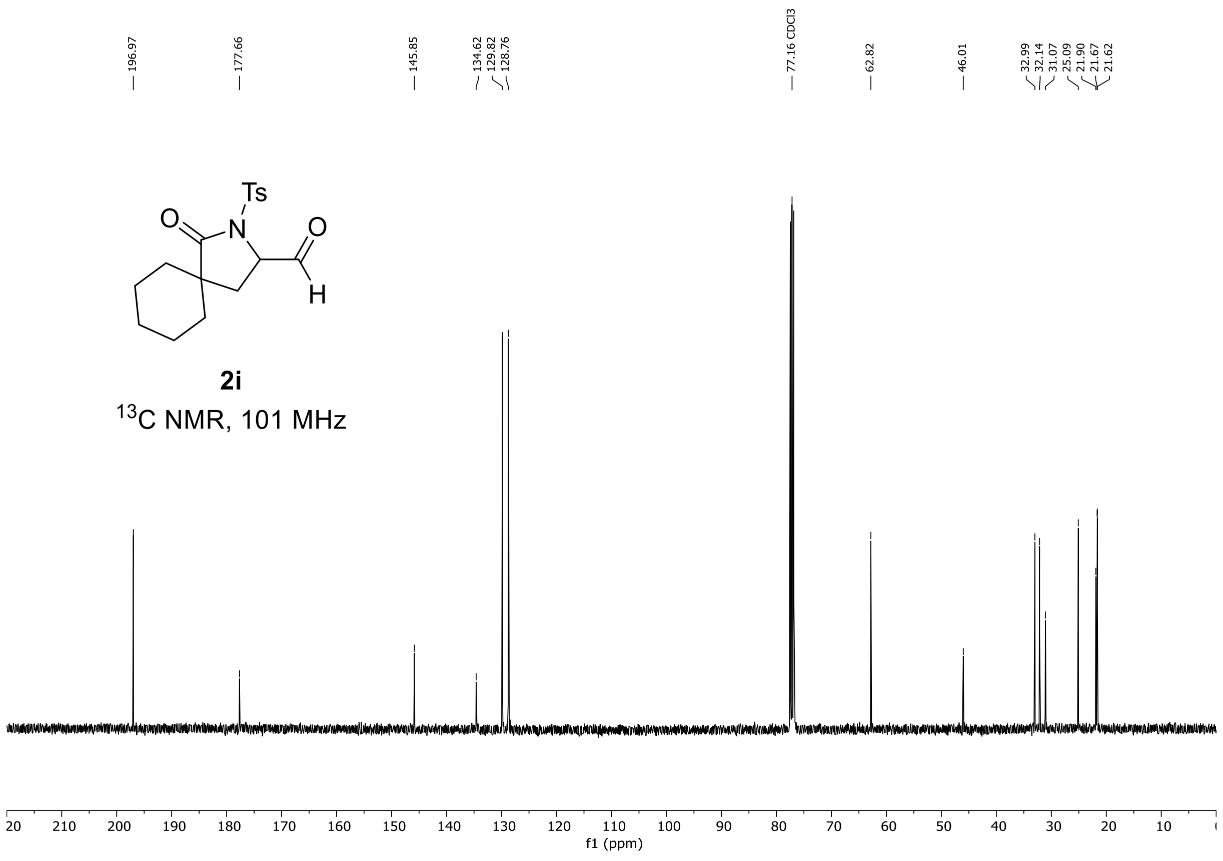

196.97
177.66
145.85
134.62
129.82
128.76
77.16 CDCl3
62.82
46.01
32.99
32.14
31.07
25.09
21.90
21.67
21.62
Ts
O
N
O
H
2i
¹³C NMR, 101 MHz
f1 (ppm)

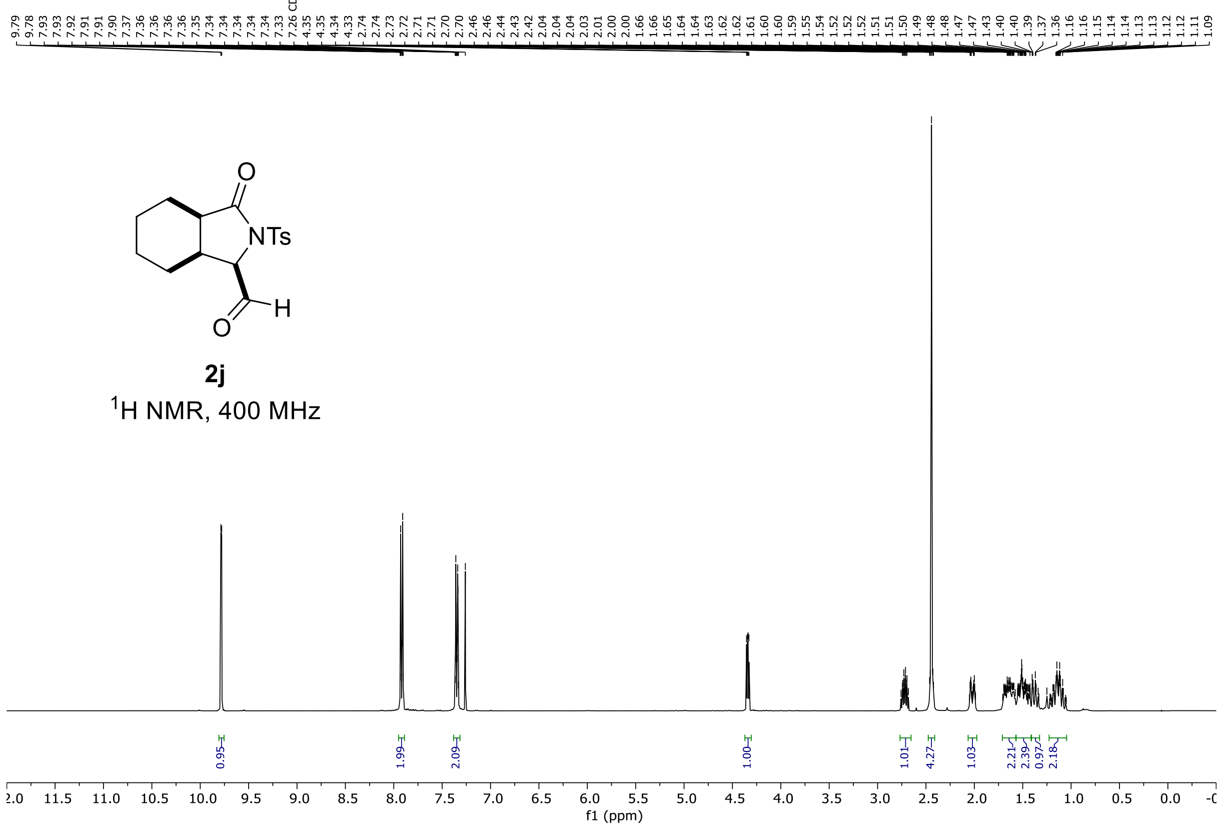

NTs
H
2j
^{1}H NMR, 400 MHz
f1 (ppm)

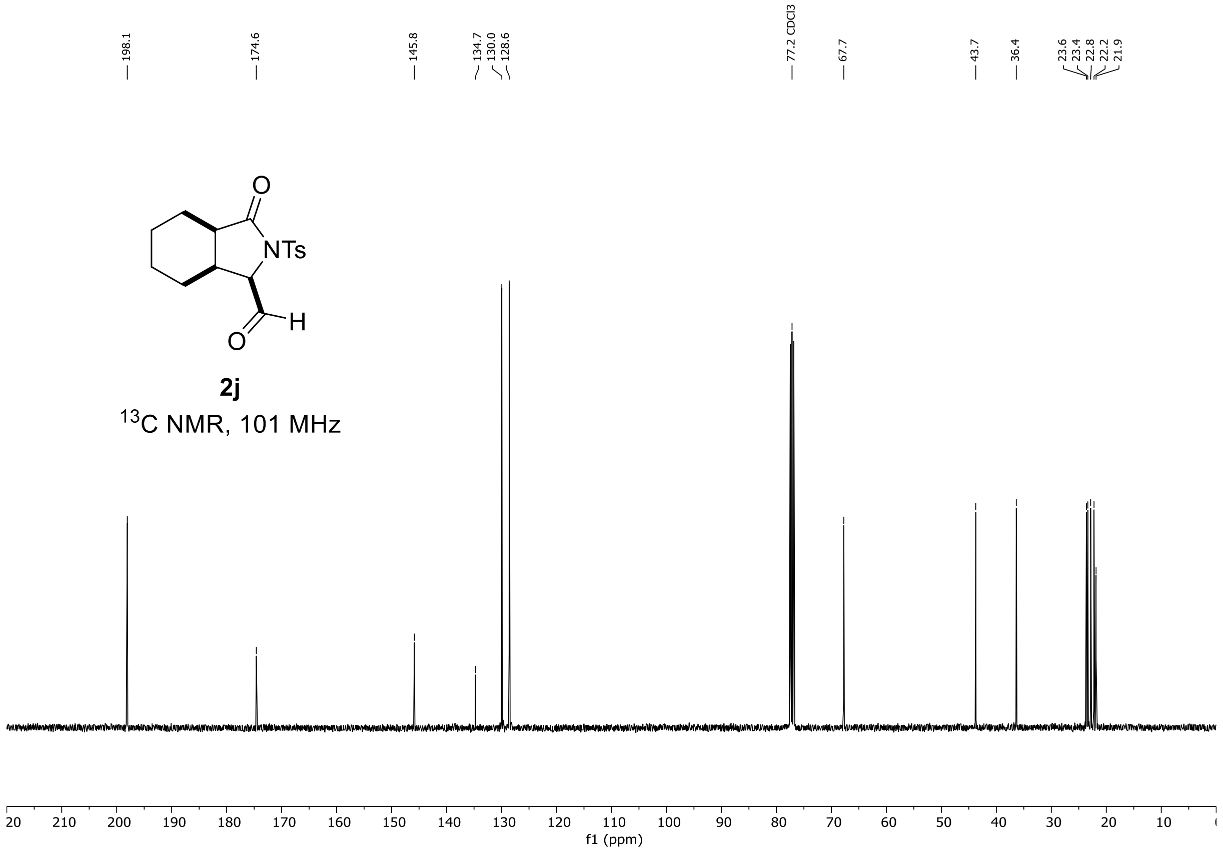

198.1
174.6
145.8
134.7
130.0
128.6
77.2 CDCl3
67.7
43.7
36.4
23.6
23.4
22.8
22.2
21.9
NTs
H
2j
^{13}C NMR, 101 MHz
f1 (ppm)

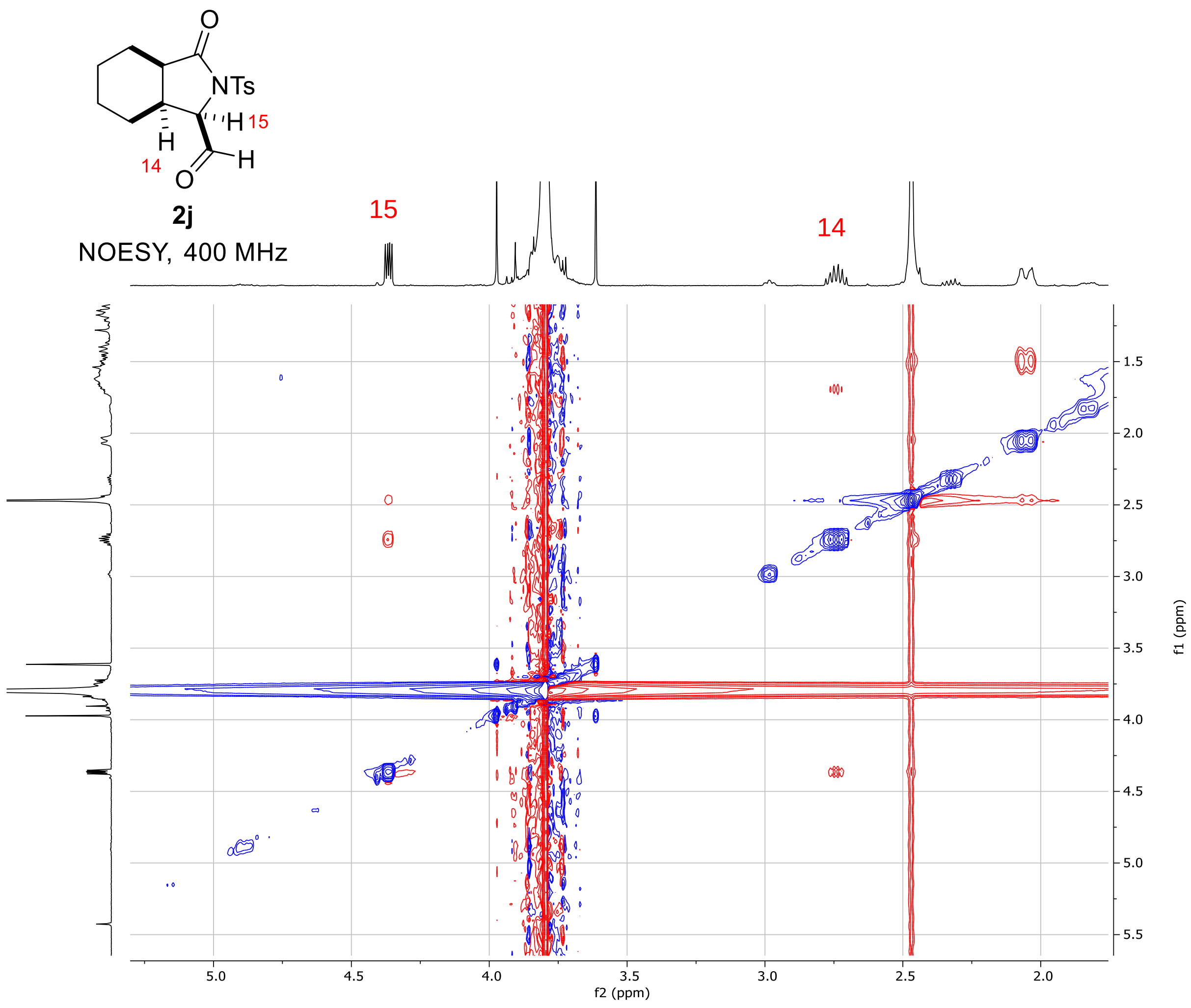

NTs
H 15
H
14
O
O
H
2j
NOESY, 400 MHz
15
14
f2 (ppm)
f1 (ppm)

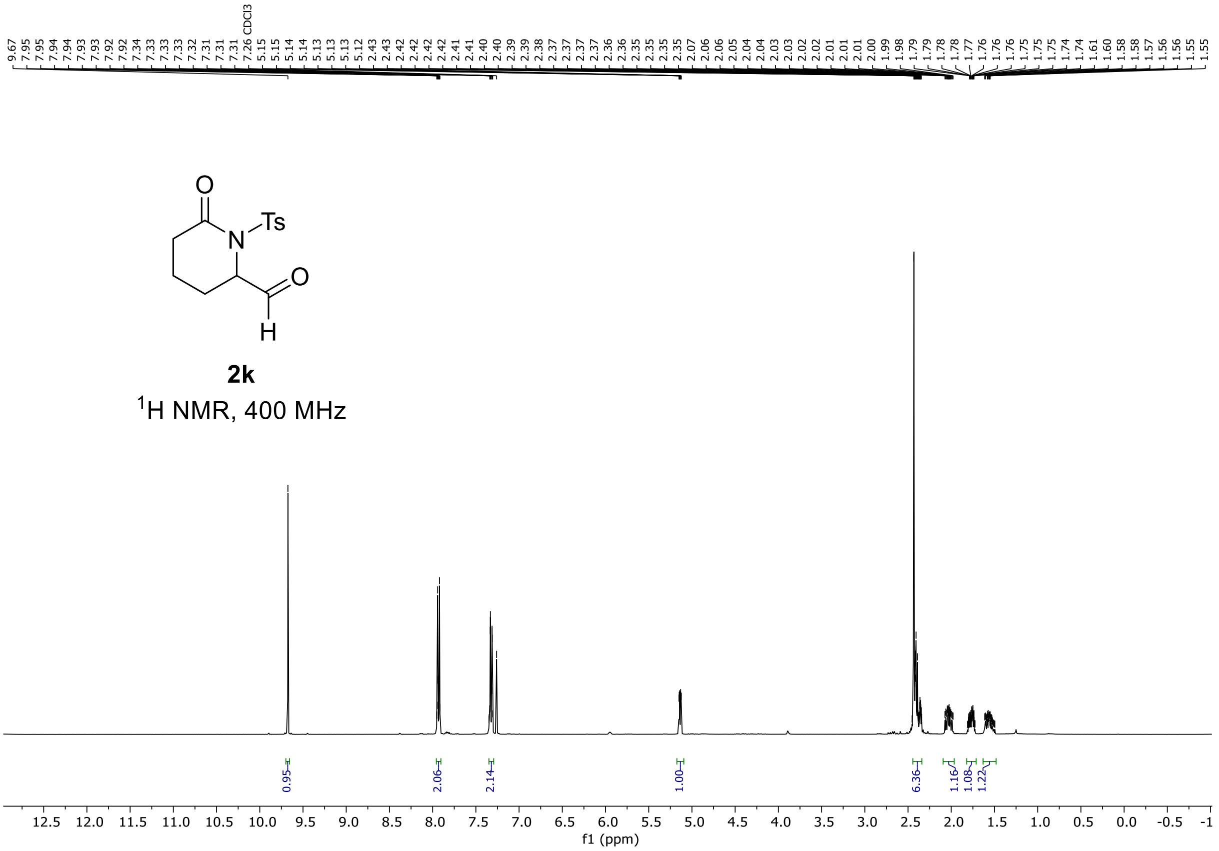

2k
^{1}H NMR, 400 MHz
f1 (ppm)

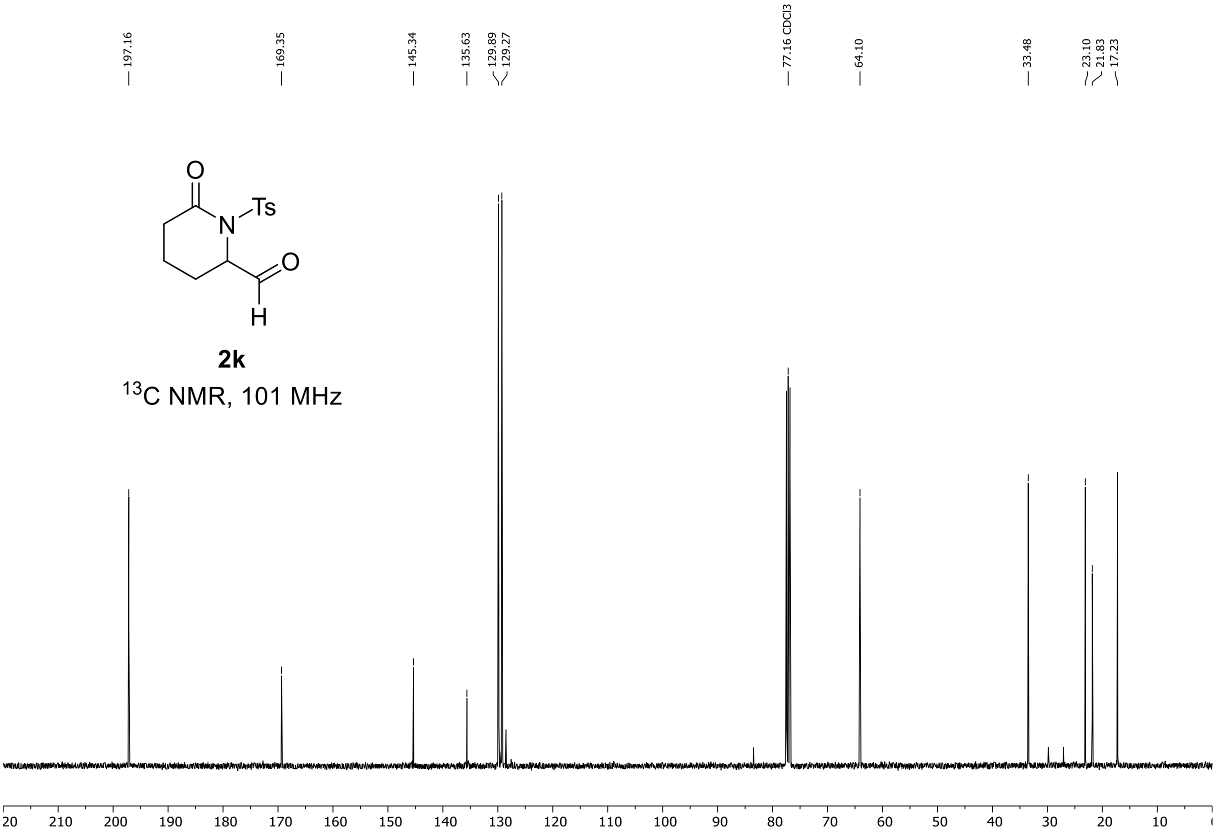

2k
^{13}C NMR, 101 MHz
197.16
169.35
145.34
135.63
129.89
129.27
77.16 CDCl3
64.10
33.48
23.10
21.83
17.23
f1 (ppm)

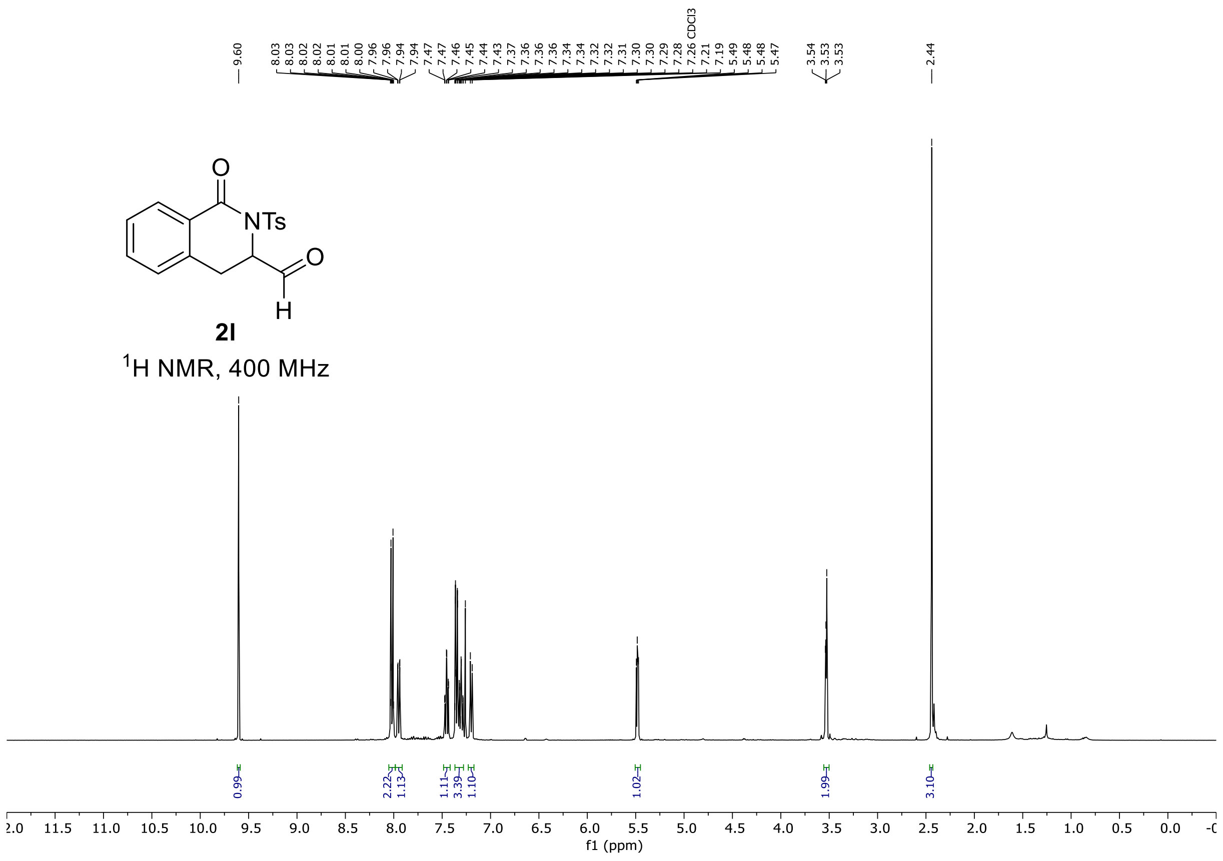

2l

^{1}H NMR, 400 MHz

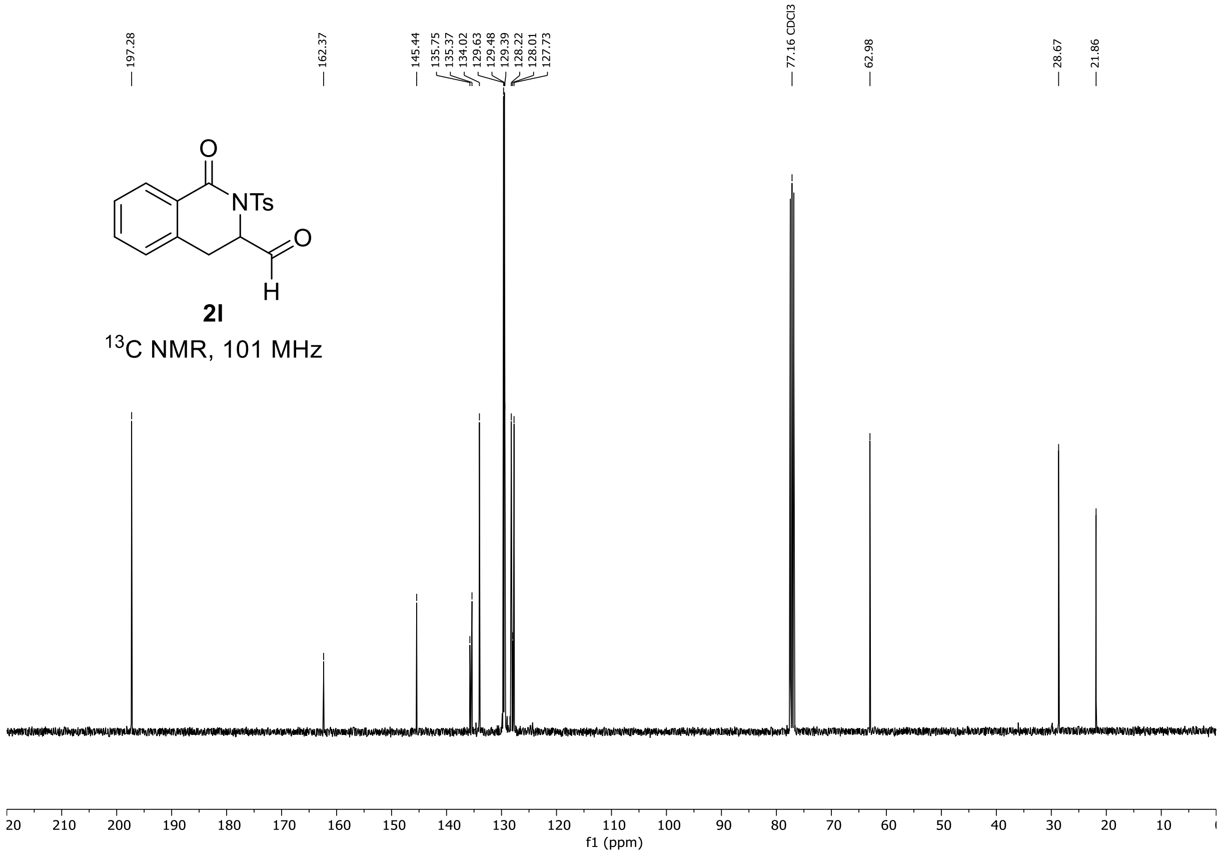

2l

^{13}C NMR, 101 MHz

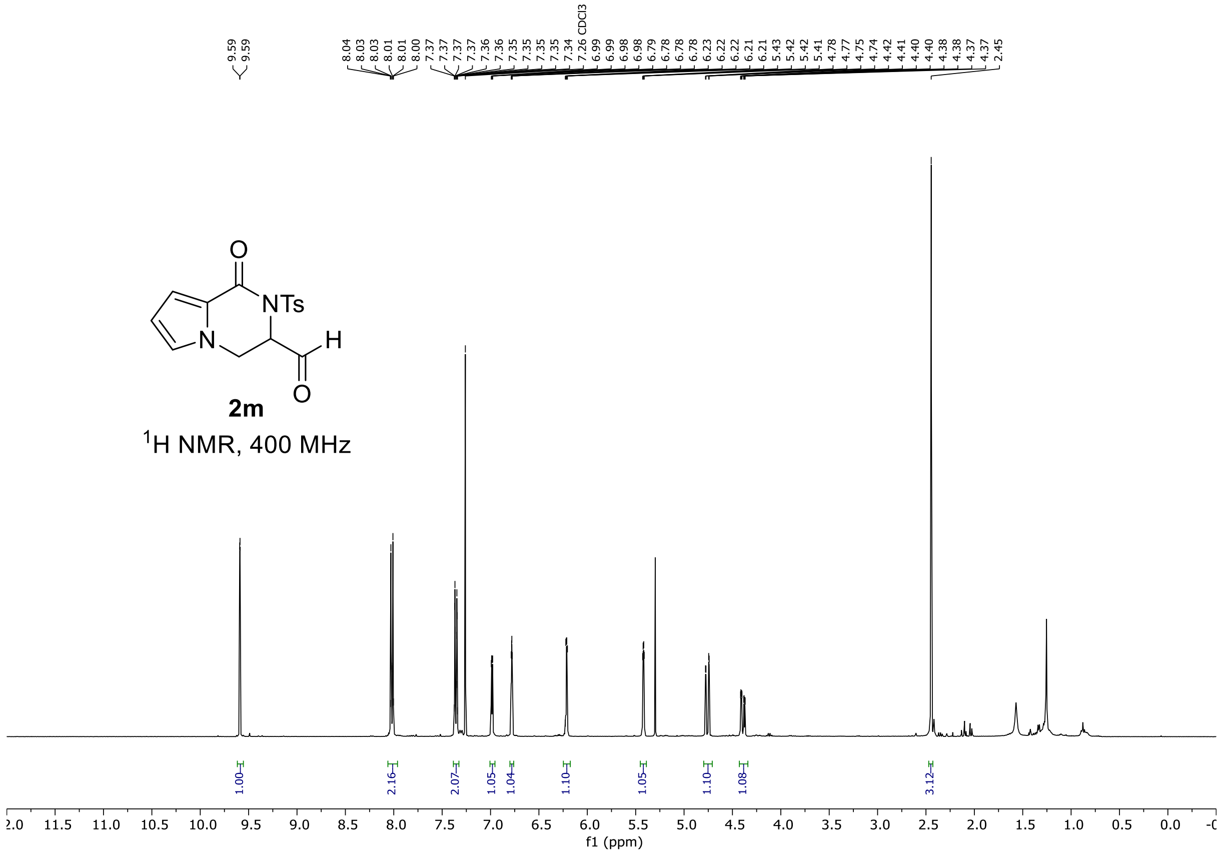

2m

^{1}H NMR, 400 MHz

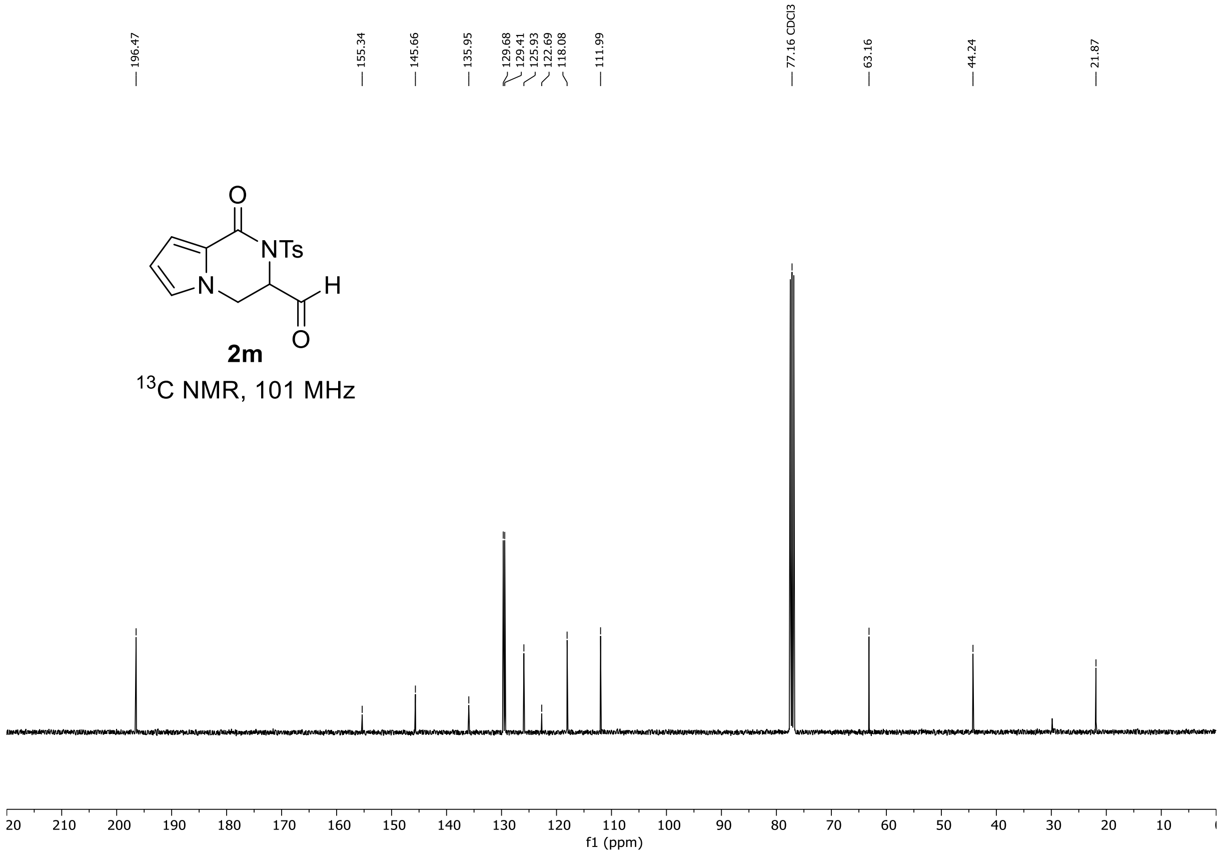

2m

^{13}C NMR, 101 MHz

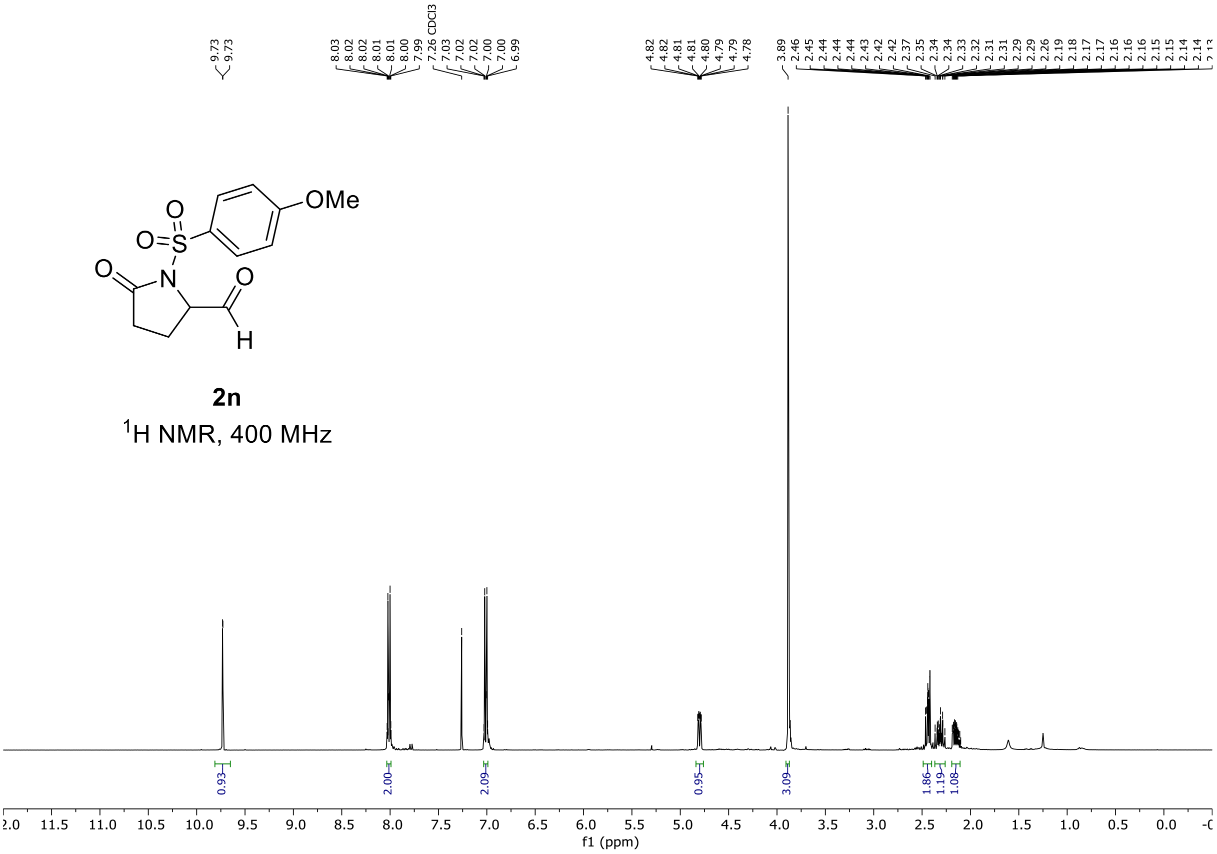

2n

^{1}H NMR, 400 MHz

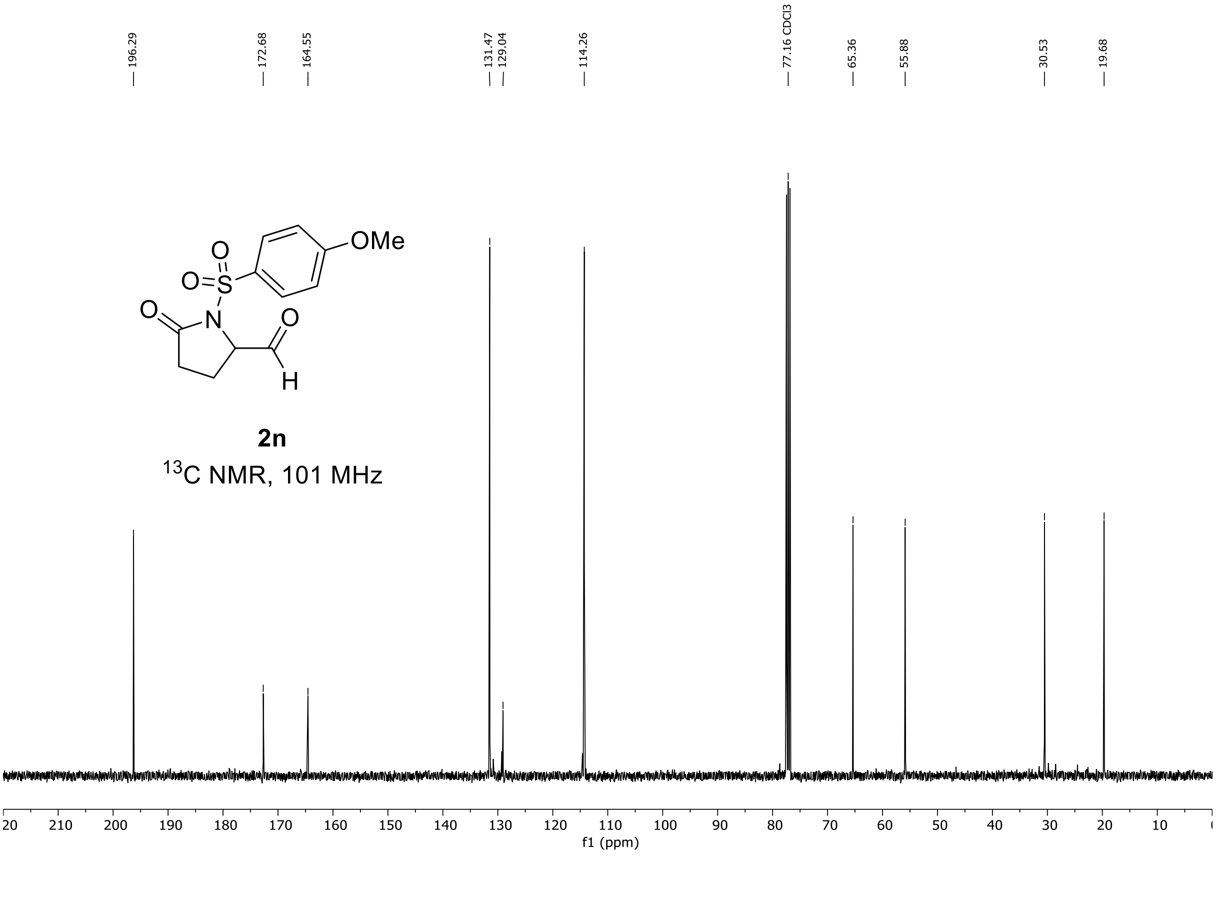

2n

^{13}C NMR, 101 MHz

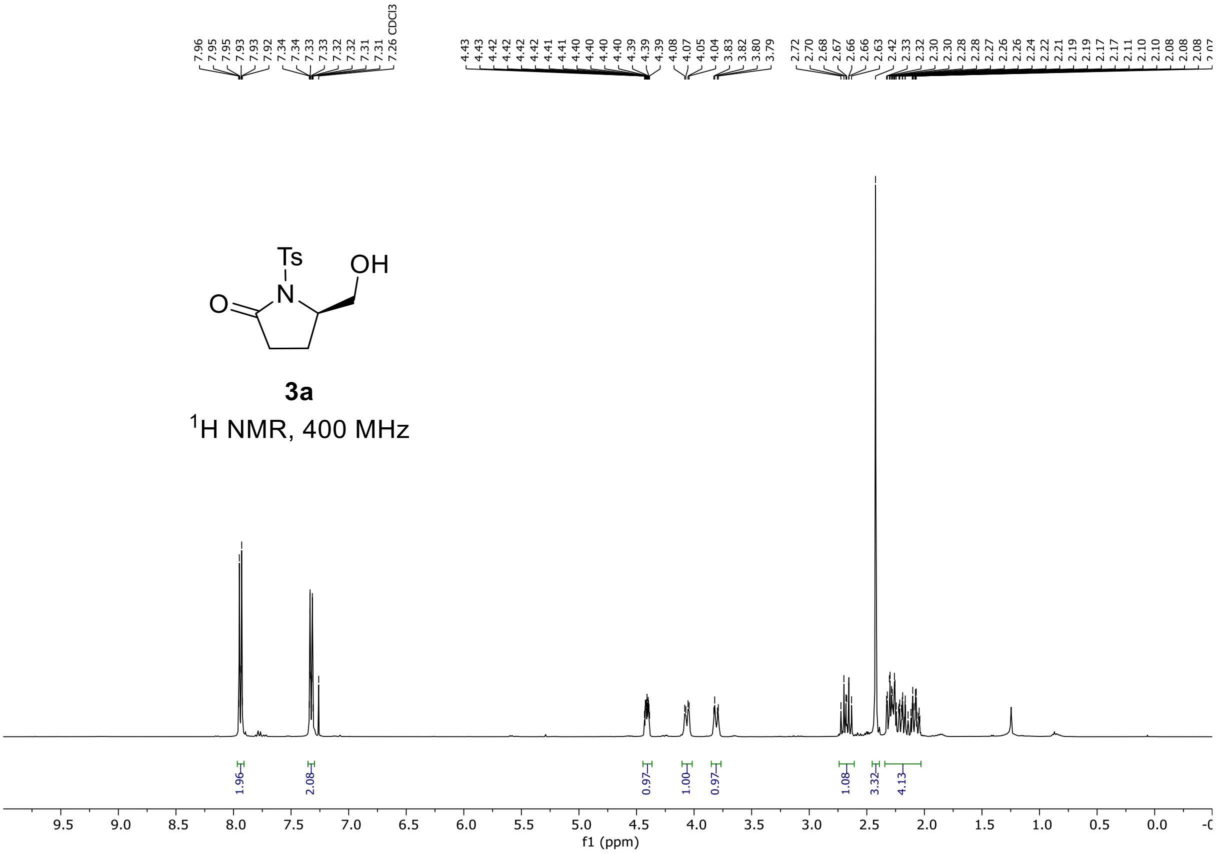

3a

^{1}H NMR, 400 MHz

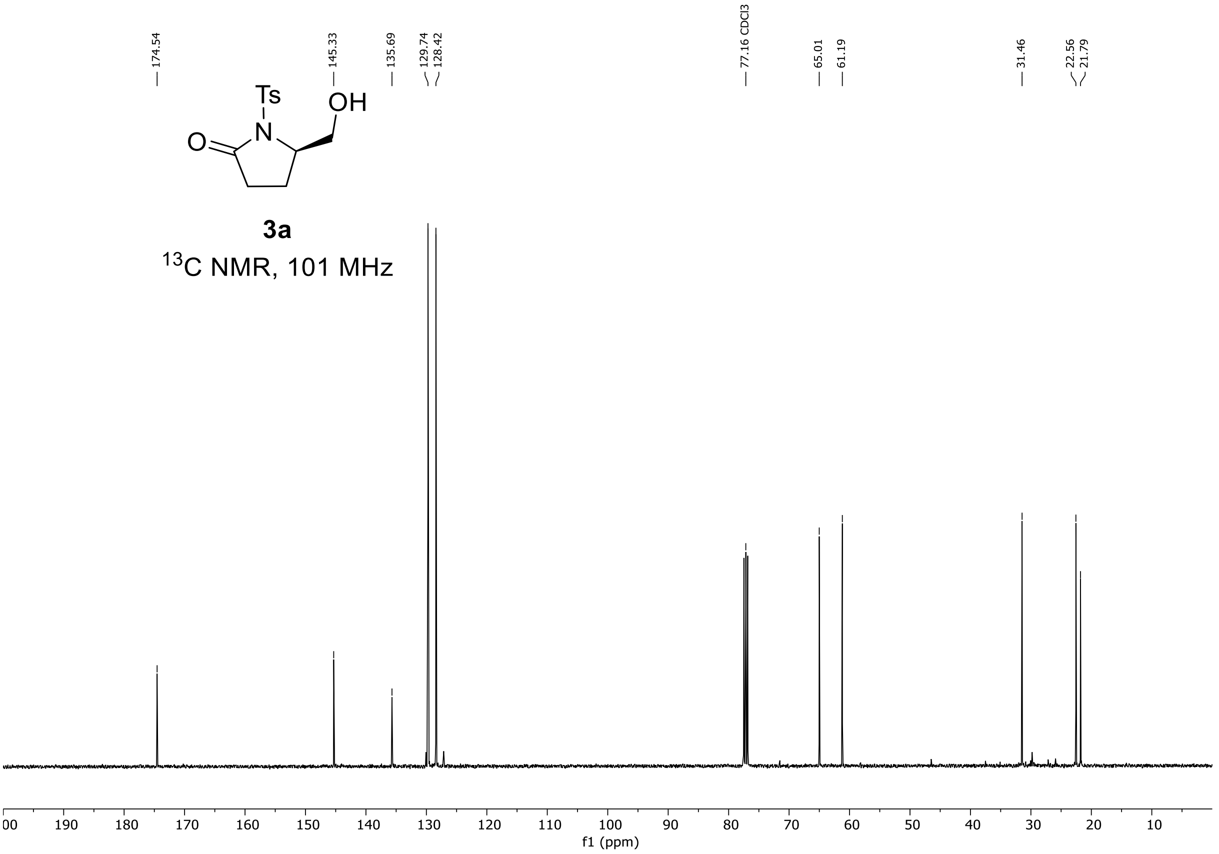

3a

^{13}C NMR, 101 MHz

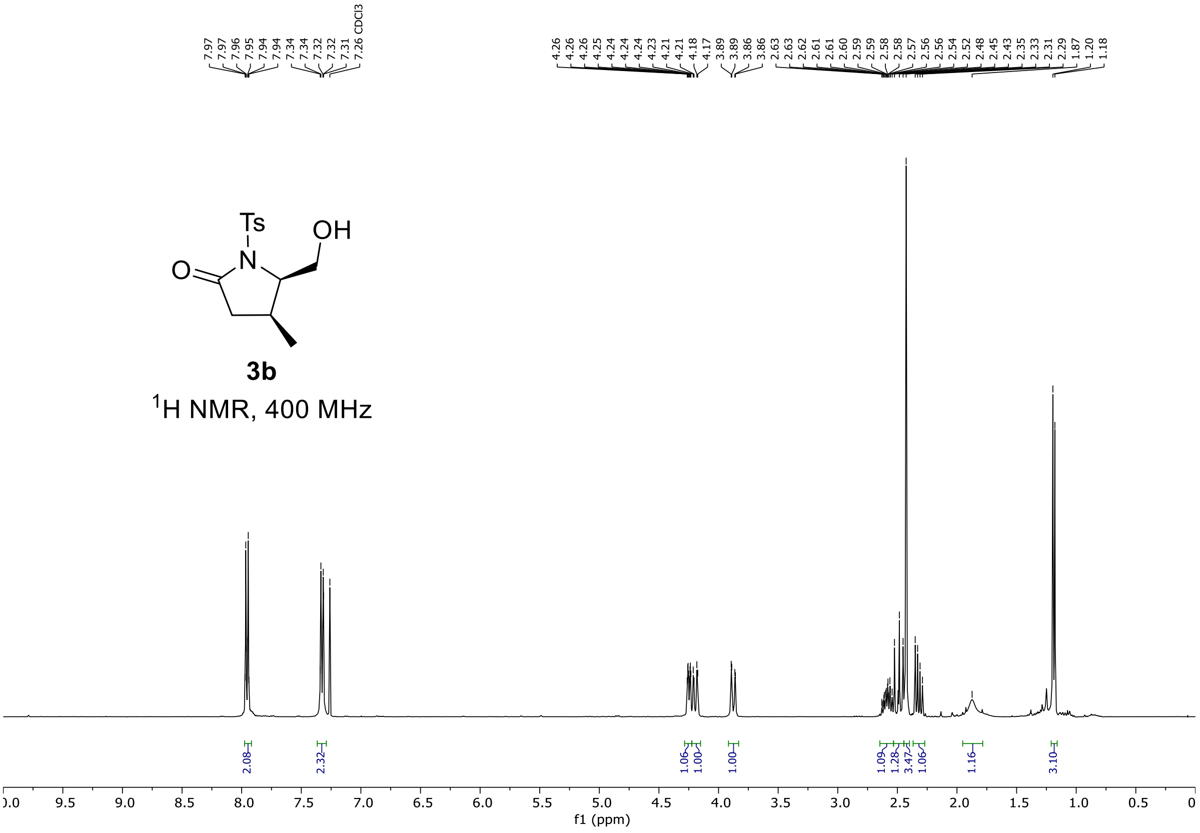
7.97
7.97
7.96
7.95
7.94
7.94
7.34
7.34
7.32
7.32
7.31
7.26 CDCl3
4.26
4.26
4.26
4.25
4.24
4.24
4.24
4.23
4.21
4.21
4.18
4.17
3.89
3.89
3.86
3.86
2.63
2.62
2.61
2.61
2.60
2.59
2.59
2.58
2.58
2.58
2.57
2.56
2.56
2.54
2.52
2.48
2.45
2.43
2.35
2.33
2.31
2.29
1.87
1.20
1.18
Ts
N
OH
O
3b
^{1}H NMR, 400 MHz
2.08
2.32
1.06
1.00
1.00
1.09
1.28
3.47
1.06
1.16
3.10
f1 (ppm)

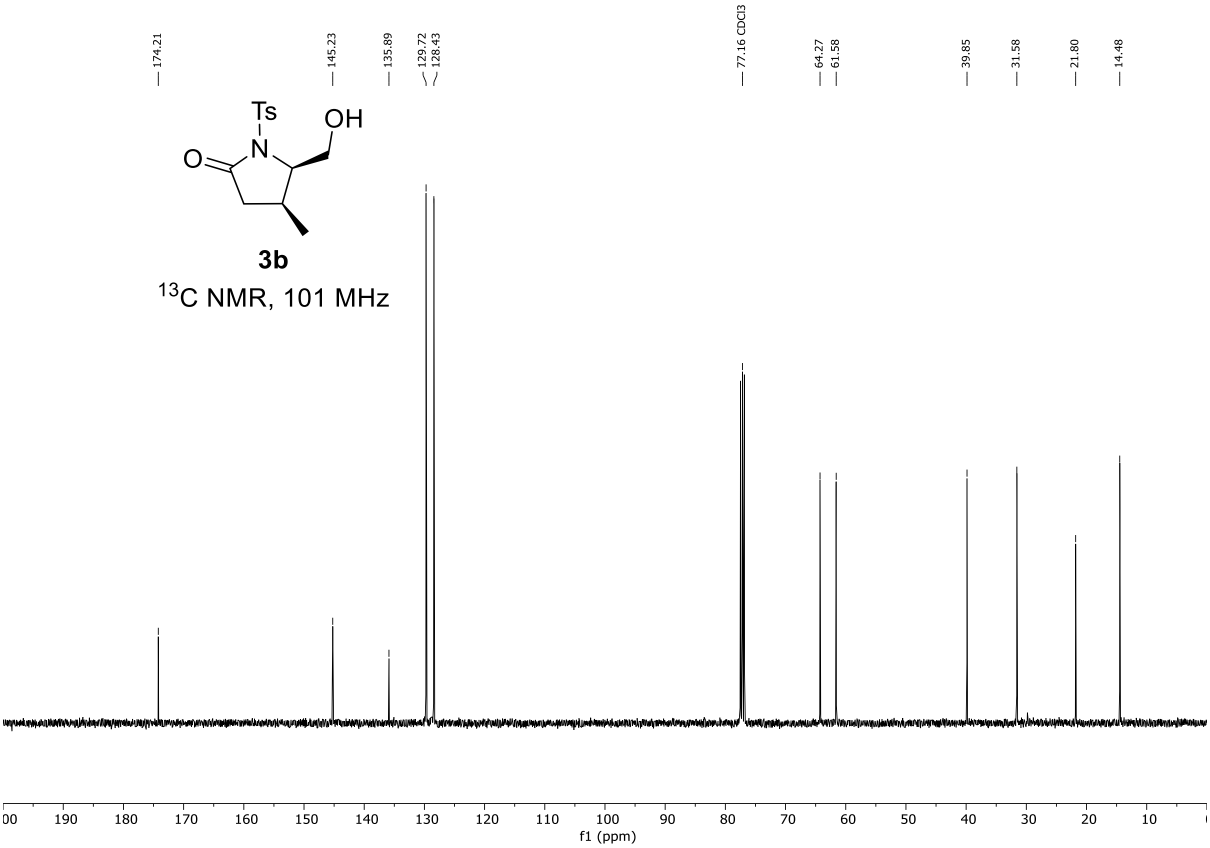
174.21
145.23
135.89
129.72
128.43
77.16 CDCl3
64.27
61.58
39.85
31.58
21.80
14.48
Ts
N
OH
O
3b
^{13}C NMR, 101 MHz
f1 (ppm)

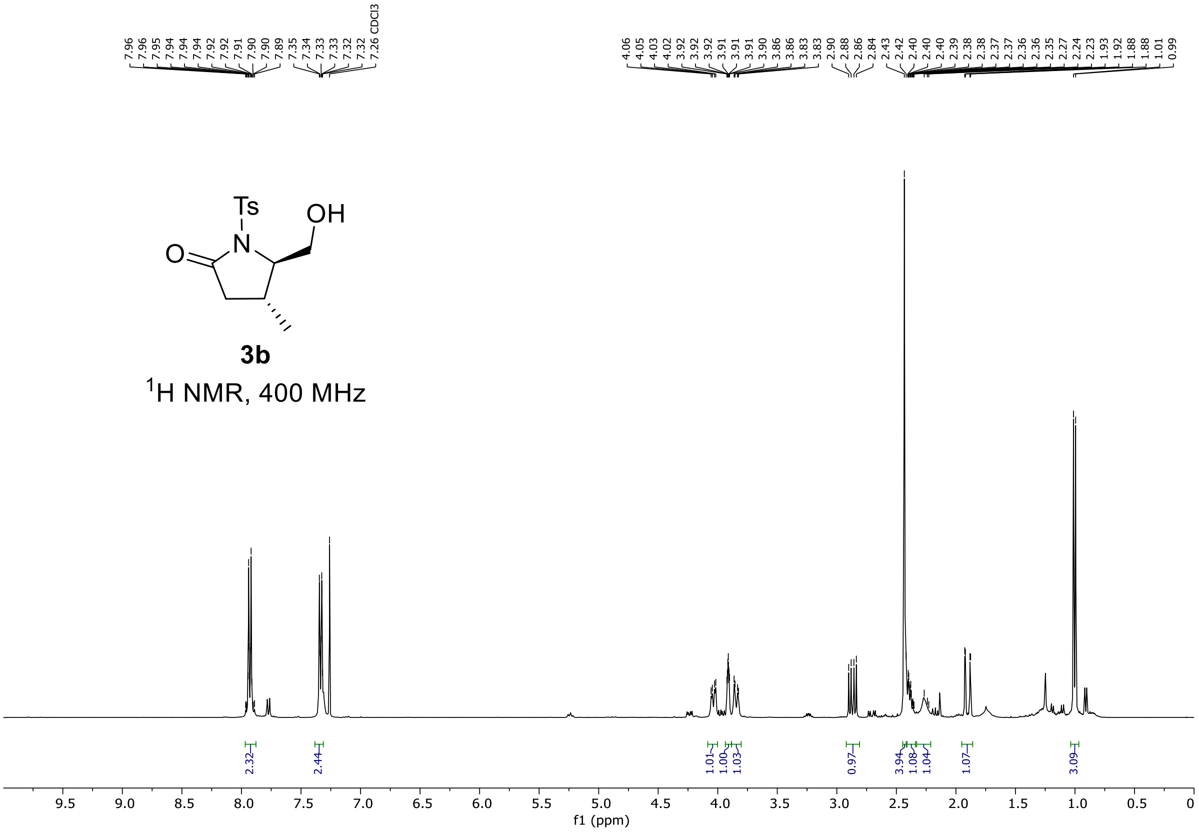
7.96 7.96 7.95 7.94 7.94 7.94 7.92 7.92 7.91 7.90 7.90 7.89 7.35 7.34 7.33 7.33 7.32 7.32 7.26 CDCl3
4.06 4.05 4.03 4.02 3.92 3.92 3.91 3.91 3.91 3.90 3.86 3.86 3.83 3.83 2.90 2.88 2.86 2.84 2.43 2.42 2.40 2.40 2.40 2.39 2.38 2.38 2.37 2.36 2.35 2.27 2.24 2.23 1.93 1.92 1.88 1.88 1.01 0.99
Ts
OH
N
O
3b
^{1}H NMR, 400 MHz
2.32
2.44
1.01
1.00
1.03
0.97
3.94
1.08
1.04
1.07
3.09
f1 (ppm)

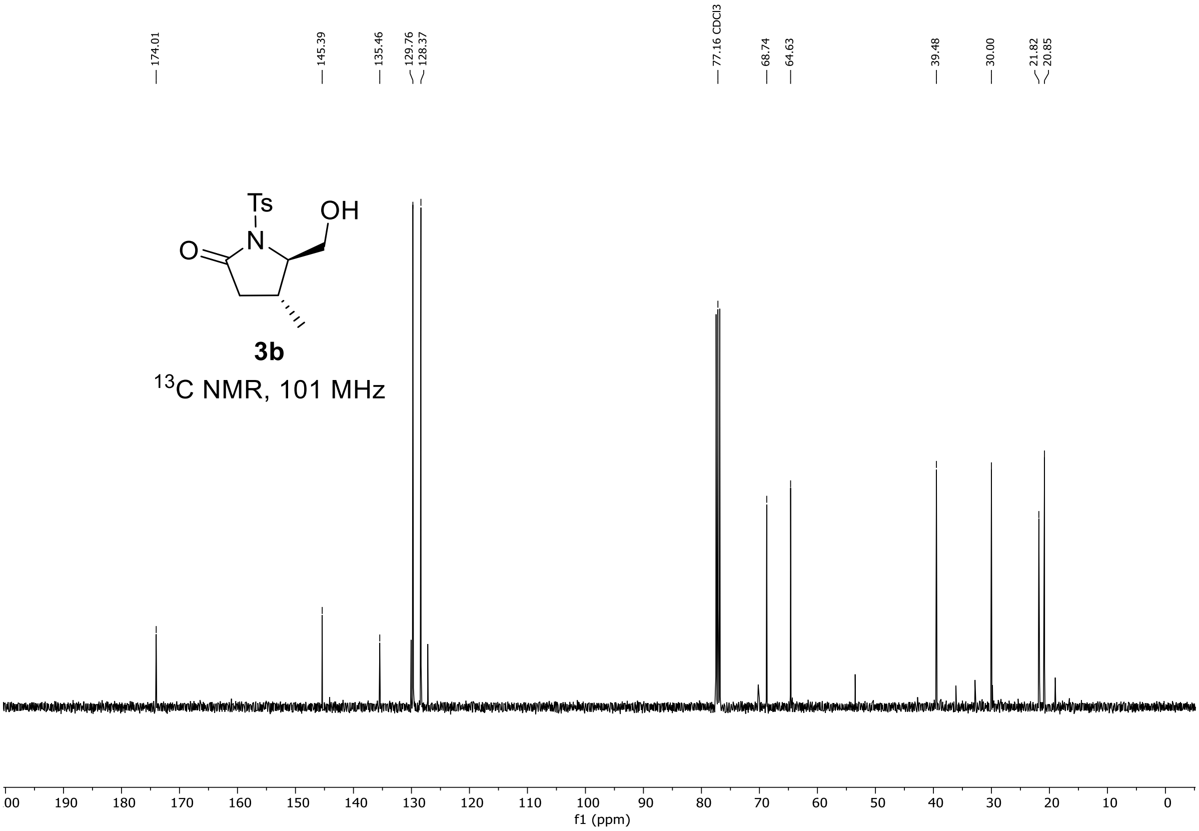
174.01
145.39
135.46
129.76
128.37
77.16 CDCl3
68.74
64.63
39.48
30.00
21.82
20.85
Ts
OH
N
O
3b
^{13}C NMR, 101 MHz
f1 (ppm)

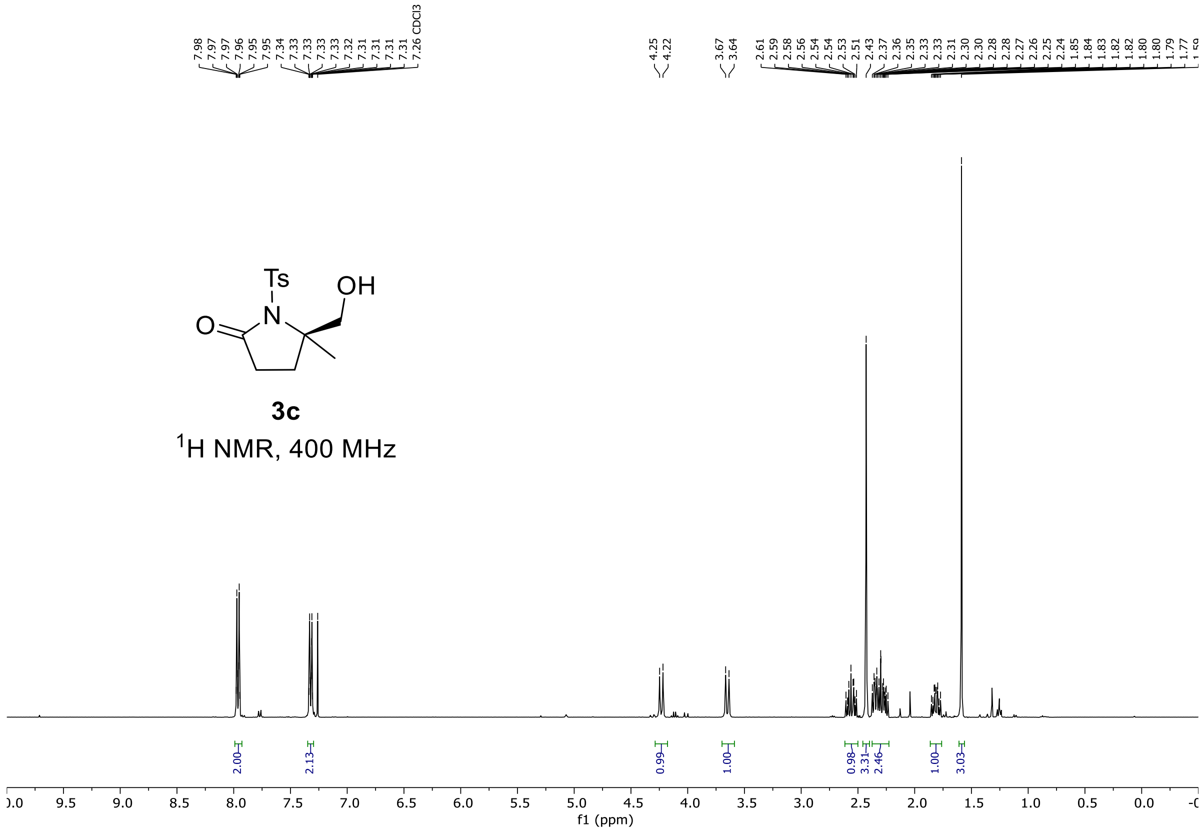

Ts
N
O
OH
3c
^{1}H NMR, 400 MHz
f1 (ppm)

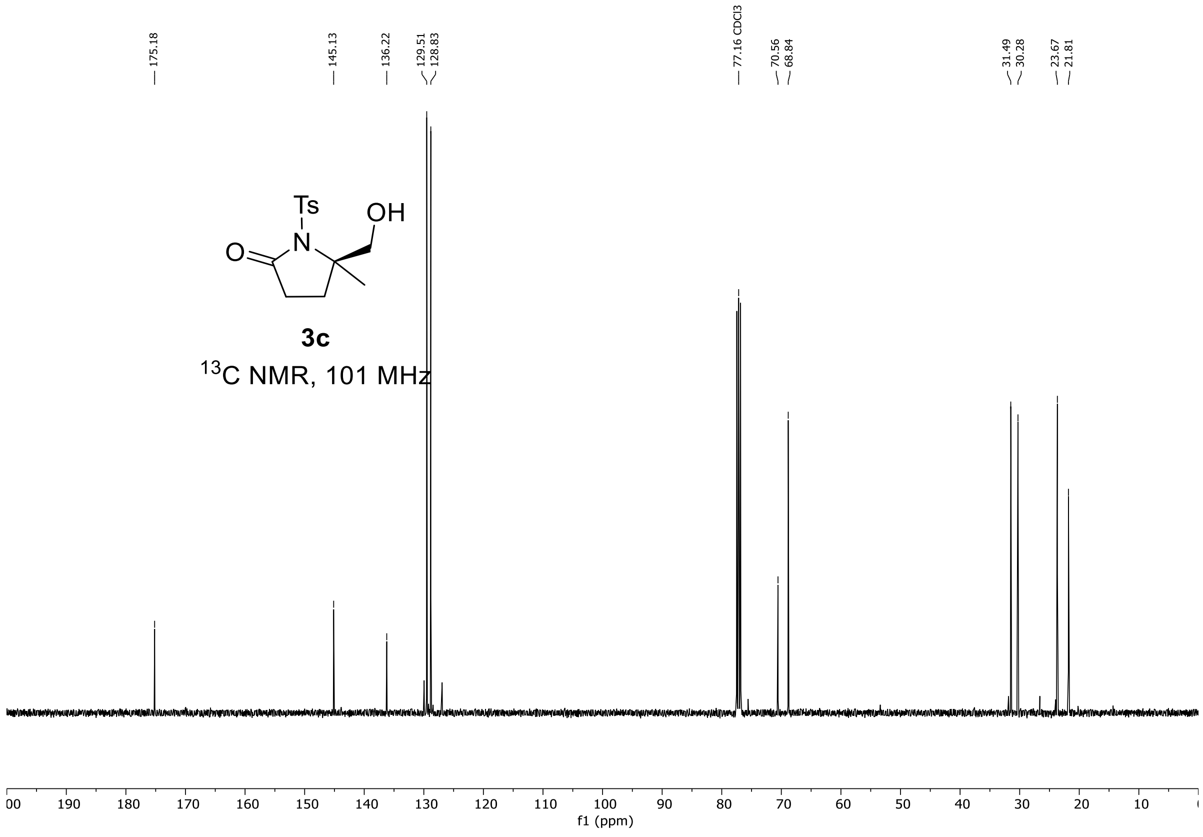

Ts
N
O
OH
3c
^{13}C NMR, 101 MHz
f1 (ppm)

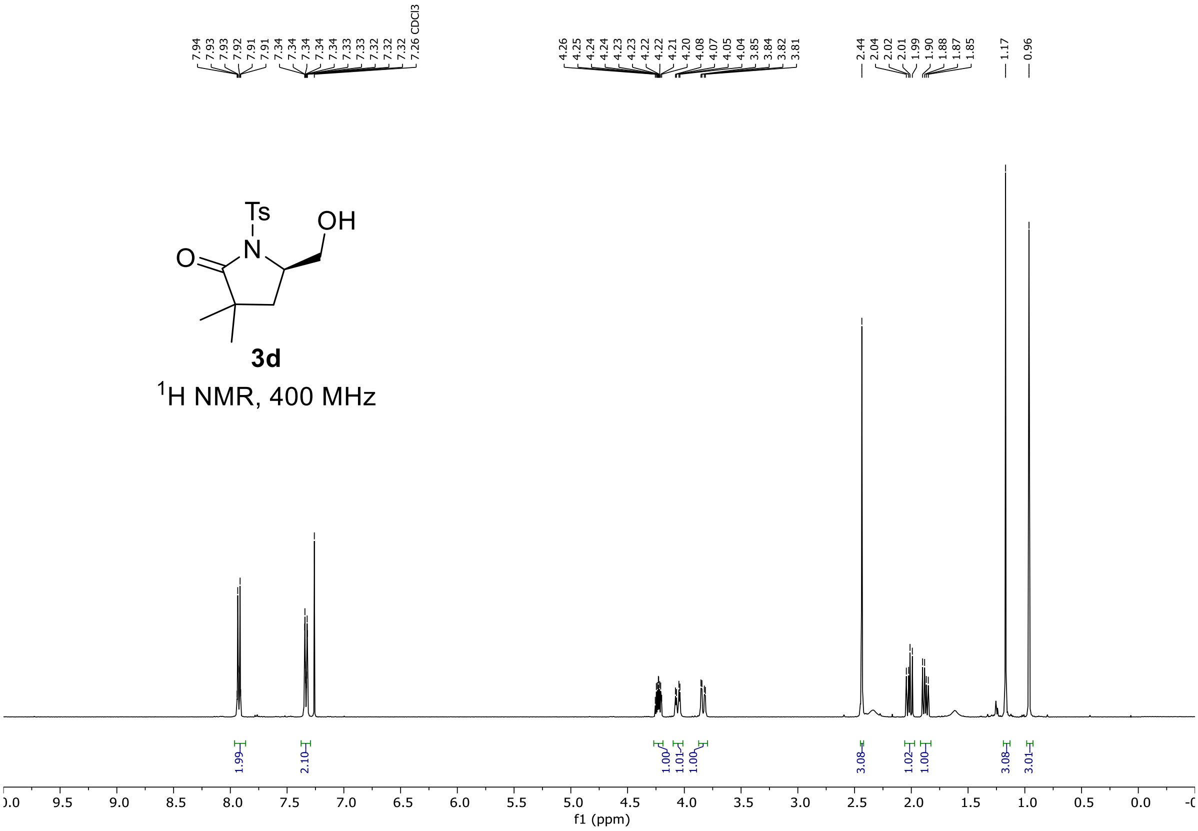

3d

^{1}H NMR, 400 MHz

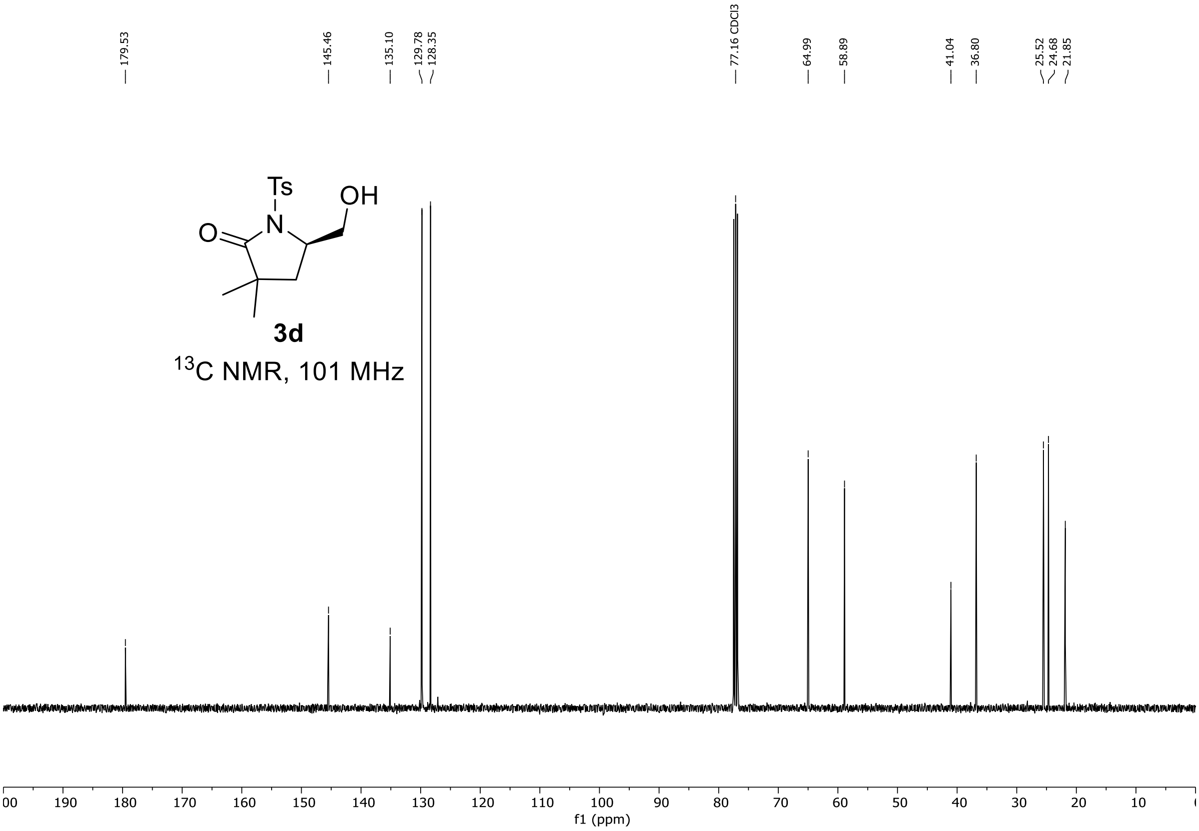

3d

^{13}C NMR, 101 MHz

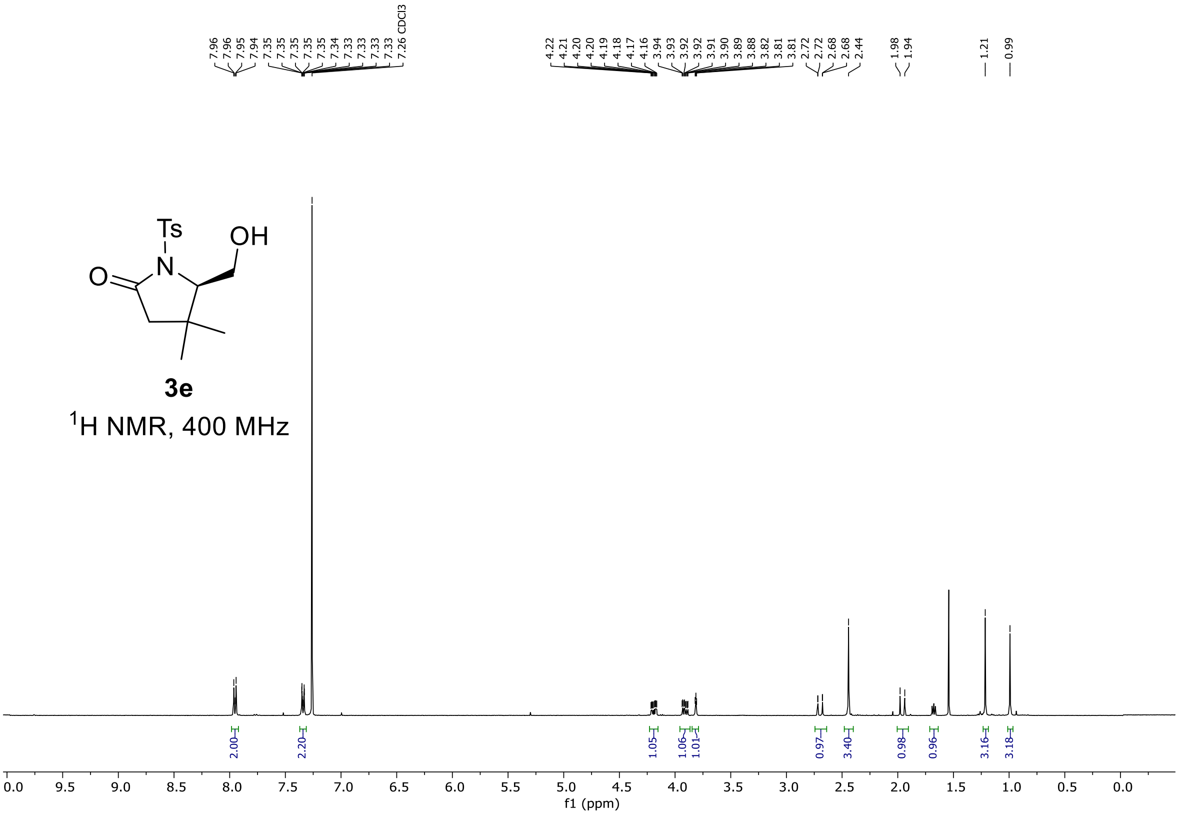
7.96
7.96
7.95
7.94
7.35
7.35
7.35
7.35
7.35
7.34
7.33
7.33
7.33
7.33
7.26 CDCl3
4.22
4.21
4.20
4.20
4.19
4.18
4.17
4.16
3.94
3.93
3.92
3.92
3.91
3.90
3.89
3.88
3.82
3.81
3.81
2.72
2.72
2.68
2.68
2.44
1.98
1.94
1.21
0.99
Ts
OH
O
N
3e
^{1}H NMR, 400 MHz
2.00
2.20
1.05
1.06
1.01
0.97
3.40
0.98
0.96
3.16
3.18
f1 (ppm)

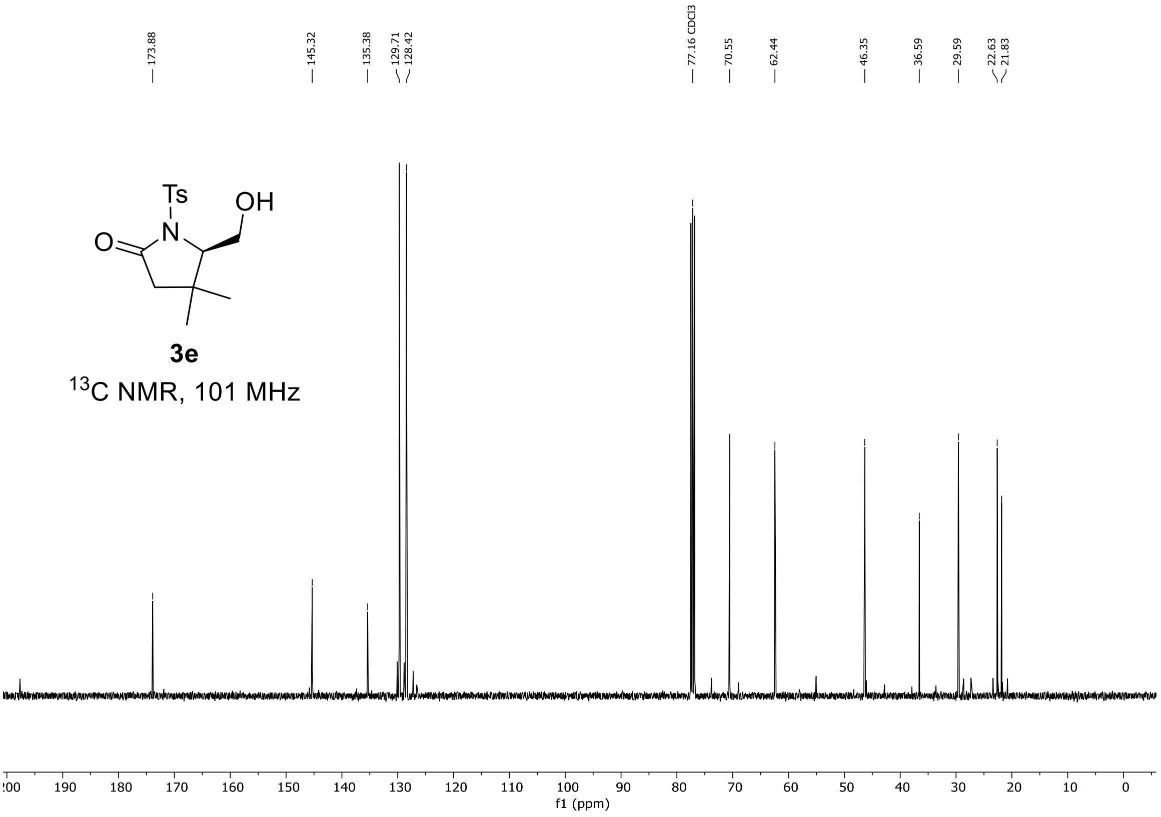
173.88
145.32
135.38
129.71
128.42
77.16 CDCl3
70.55
62.44
46.35
36.59
29.59
22.63
21.83
Ts
OH
O
N
3e
^{13}C NMR, 101 MHz
f1 (ppm)

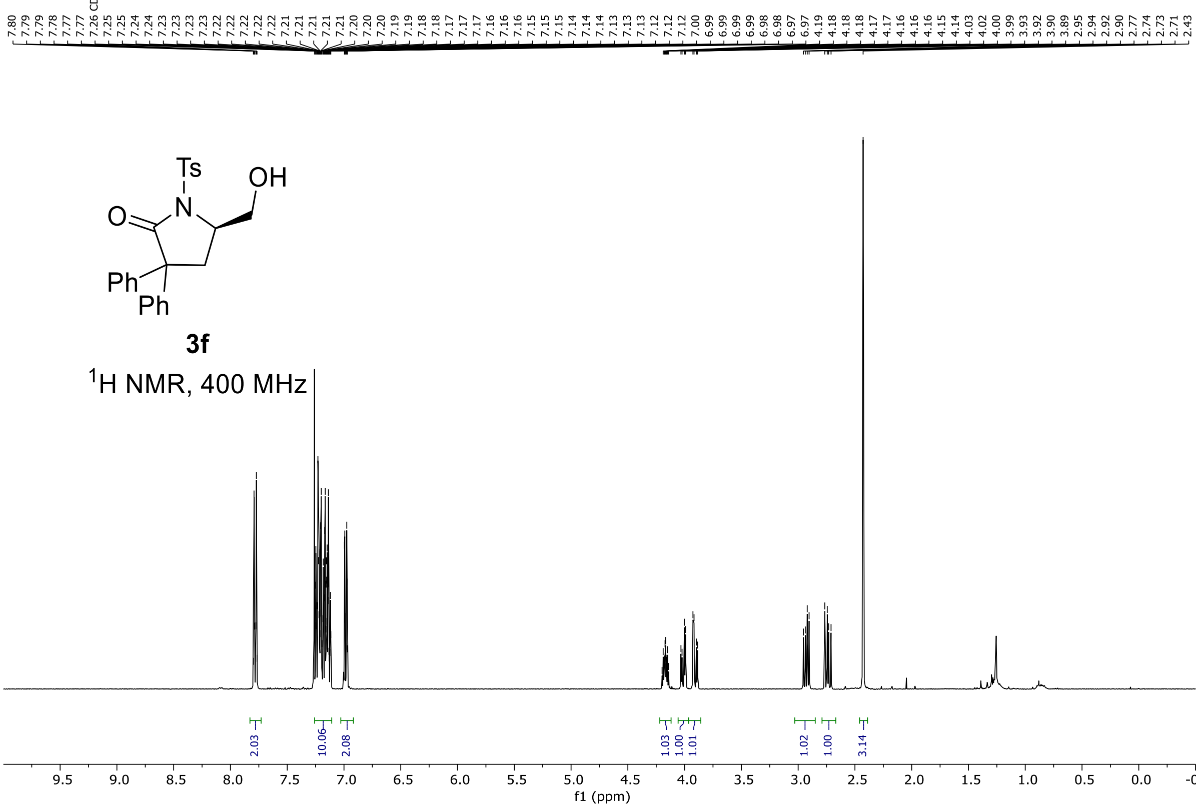

3f
1H NMR, 400 MHz
f1 (ppm)

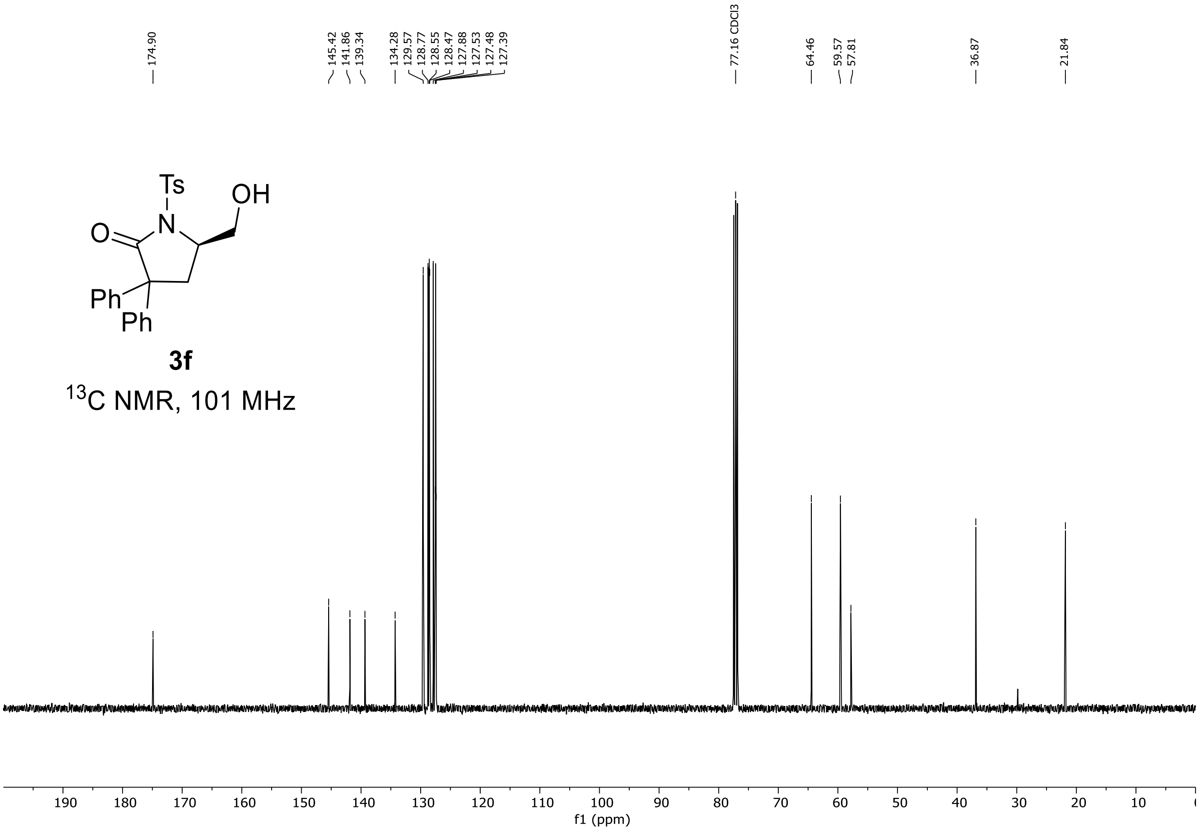

3f
13C NMR, 101 MHz
174.90
145.42
141.86
139.34
134.28
129.57
128.77
128.55
128.47
127.88
127.53
127.48
127.39
77.16 CDCl3
64.46
59.57
57.81
36.87
21.84
f1 (ppm)

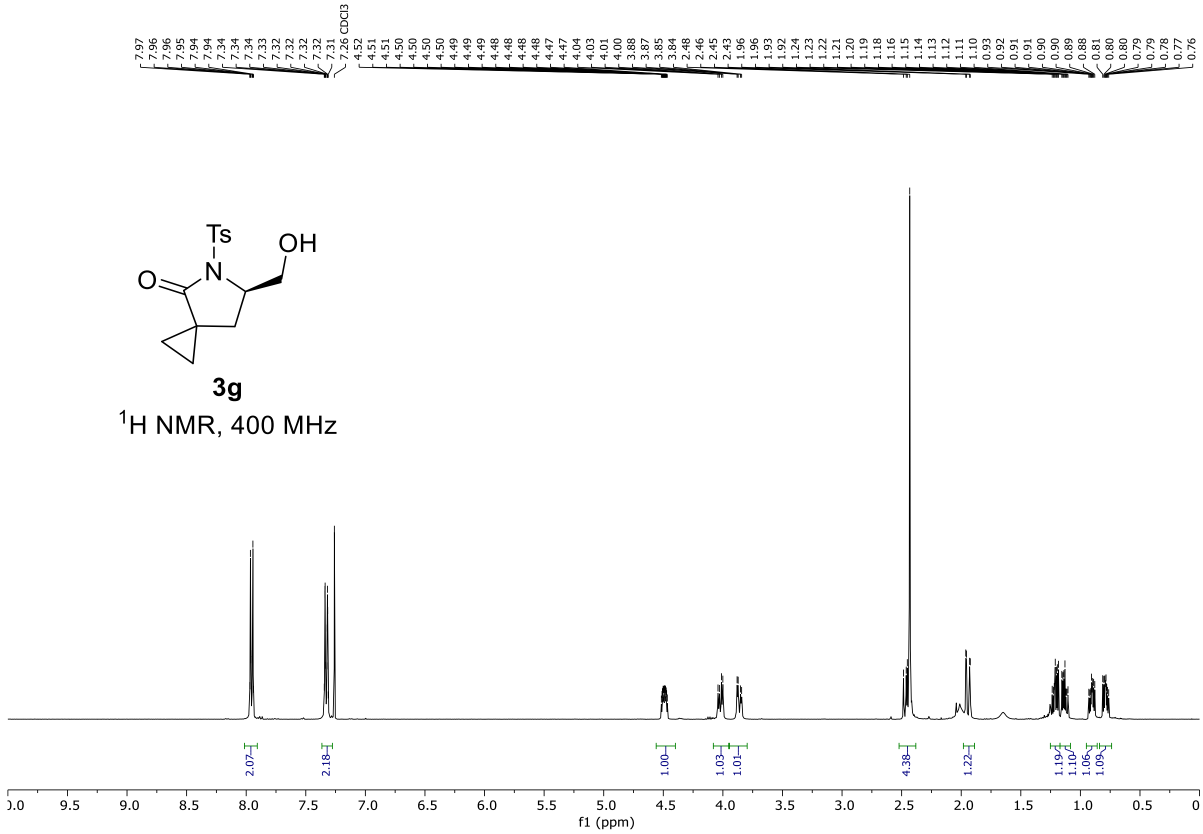

7.97 7.96 7.96 7.95 7.94 7.94 7.34 7.34 7.33 7.32 7.32 7.32 7.32 7.31 7.26 CDCl3
4.52 4.51 4.51 4.50 4.50 4.50 4.49 4.49 4.49 4.48 4.48 4.48 4.48 4.47 4.04 4.03 4.01 4.00 3.88 3.87 3.85 3.84 2.48 2.46 2.45 2.43 1.96 1.96 1.93 1.92 1.24 1.23 1.22 1.21 1.20 1.19 1.18 1.16 1.15 1.14 1.13 1.12 1.11 1.10 0.93 0.92 0.91 0.91 0.90 0.90 0.89 0.88 0.81 0.80 0.80 0.79 0.78 0.77 0.76
Ts
OH
O
N
3g
^{1}H NMR, 400 MHz
2.07
2.18
1.00
1.03
1.01
4.38
1.22
1.19
1.10
1.06
1.09
f1 (ppm)

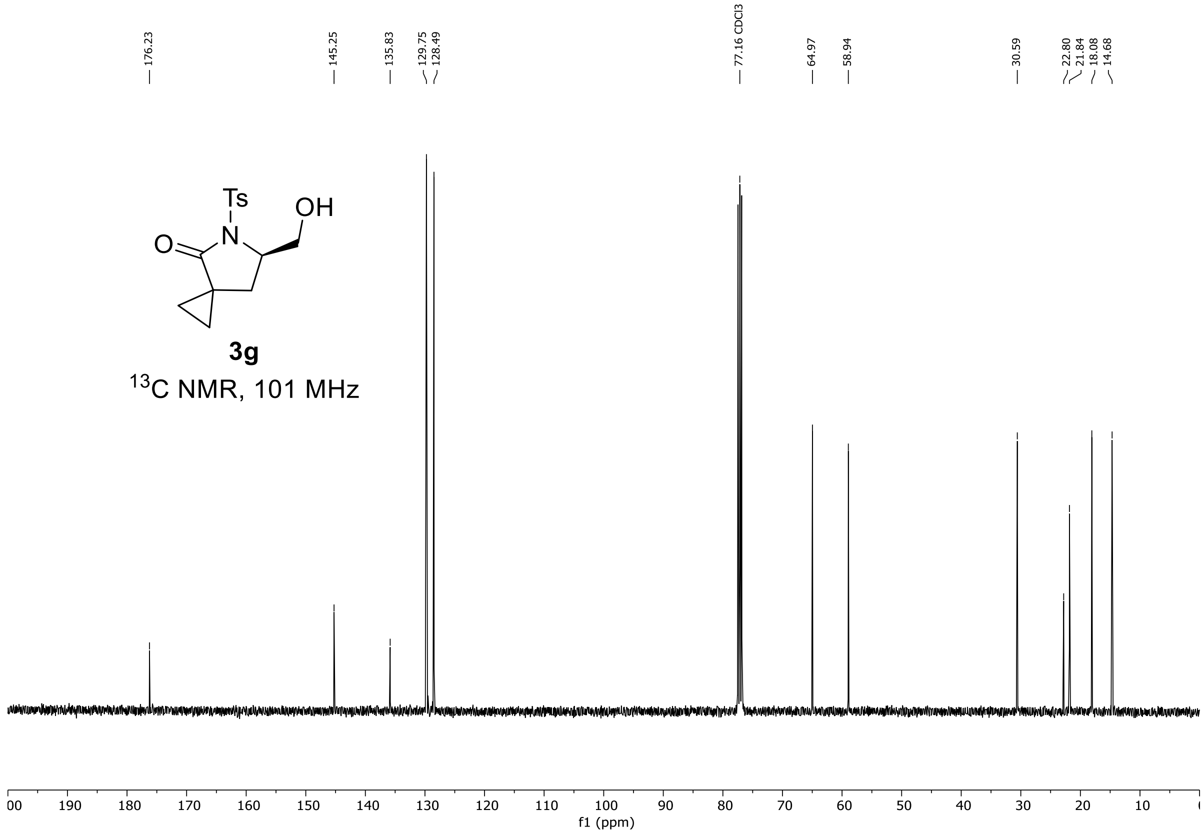

176.23
145.25
135.83
129.75
128.49
77.16 CDCl3
64.97
58.94
30.59
22.80
21.84
18.08
14.68
Ts
OH
O
N
3g
^{13}C NMR, 101 MHz
f1 (ppm)

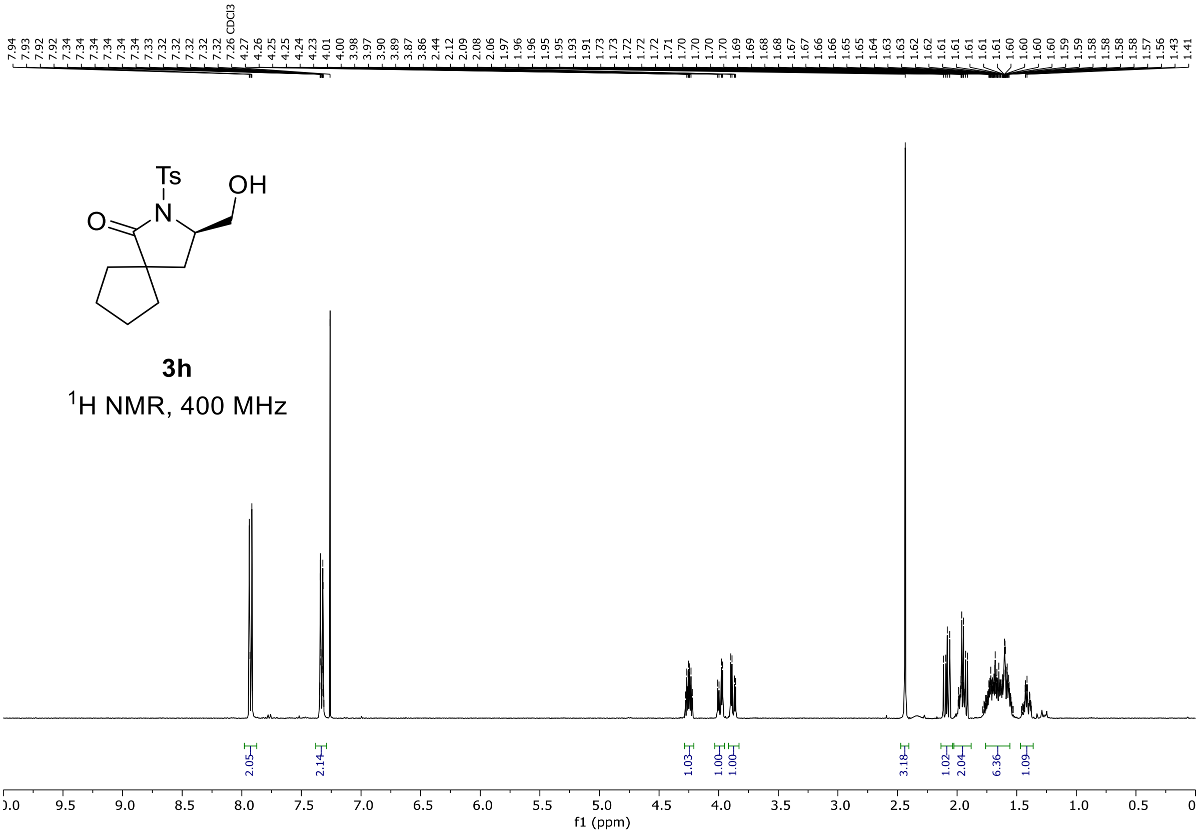

3h
^{1}H NMR, 400 MHz
f1 (ppm)

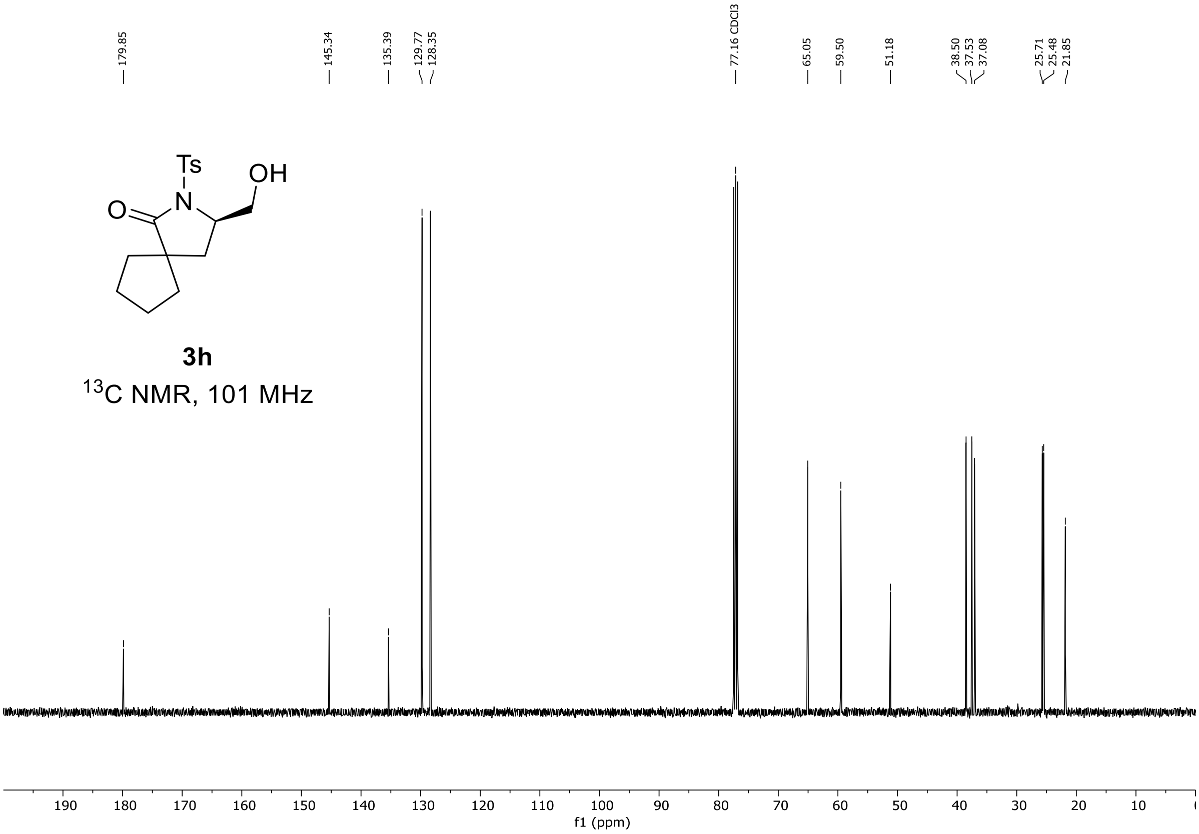

3h
^{13}C NMR, 101 MHz
179.85
145.34
135.39
129.77
128.35
77.16 CDCl3
65.05
59.50
51.18
38.50
37.53
37.08
25.71
25.48
21.85
f1 (ppm)

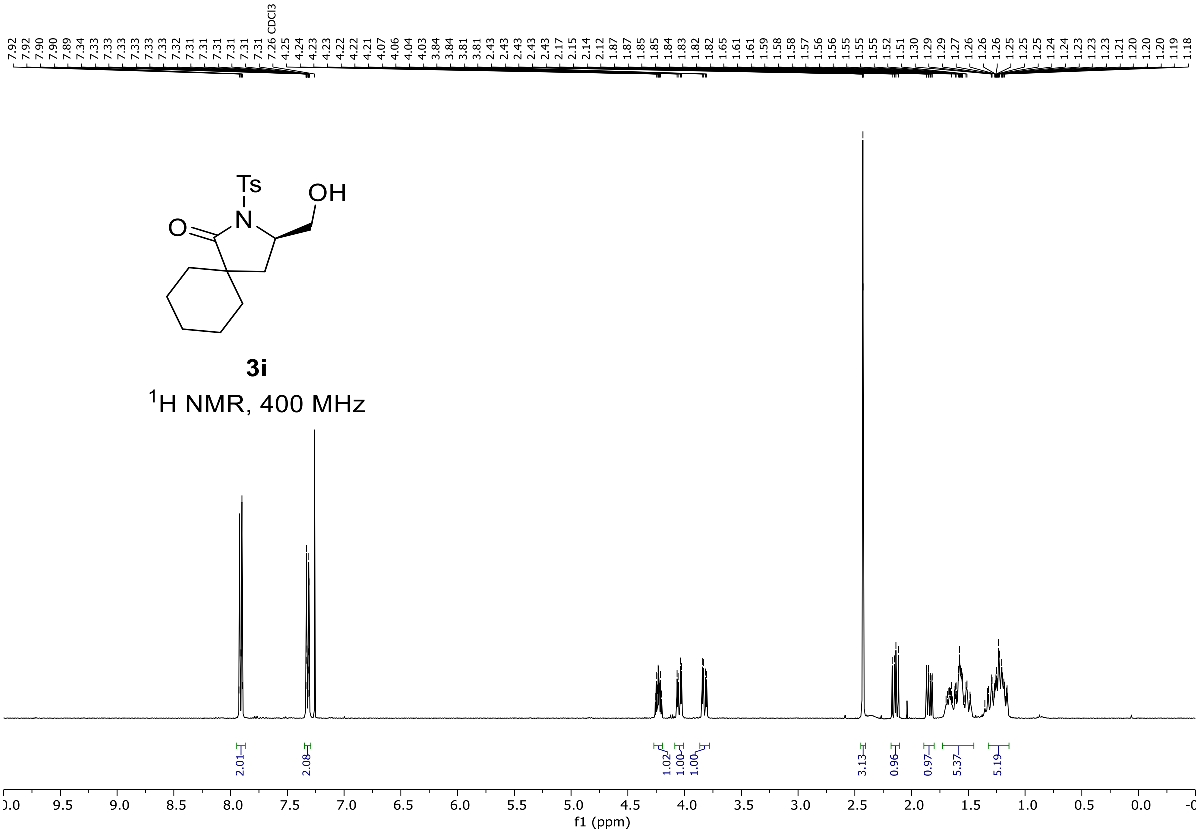
Ts
OH
O
N
3i
^{1}H NMR, 400 MHz
CDCl3
2.01
2.08
1.02
1.00
1.00
3.13
0.96
0.97
5.37
5.19
f1 (ppm)

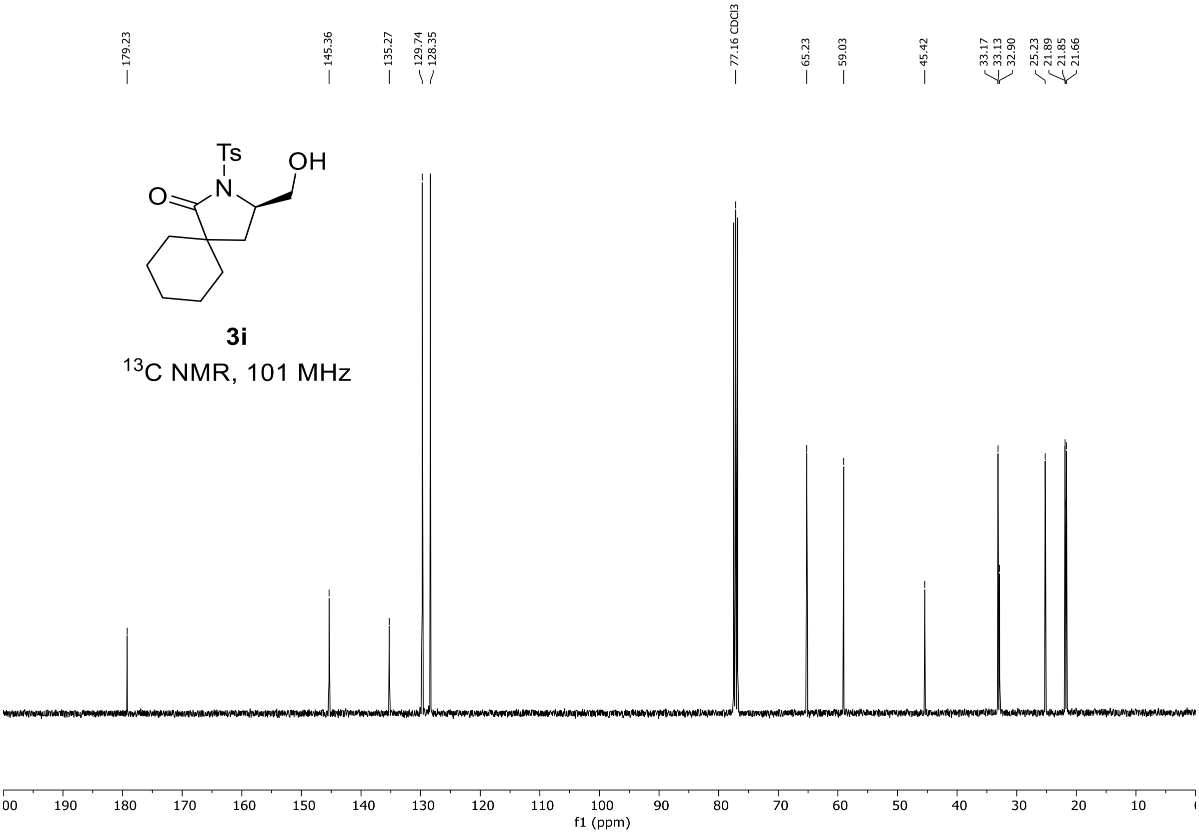
179.23
145.36
135.27
129.74
128.35
77.16 CDCl3
65.23
59.03
45.42
33.17
33.13
32.90
25.23
21.89
21.85
21.66
Ts
OH
O
N
3i
^{13}C NMR, 101 MHz
f1 (ppm)

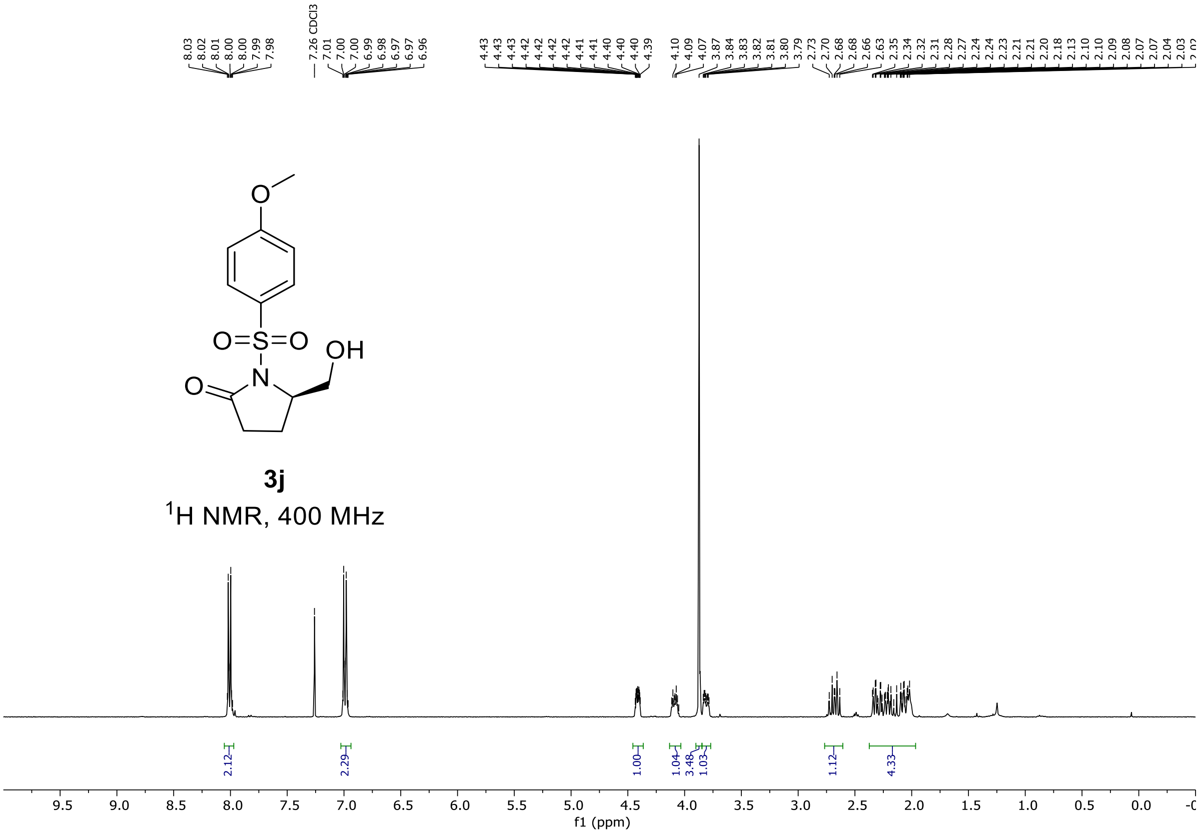

3j
^1H NMR, 400 MHz

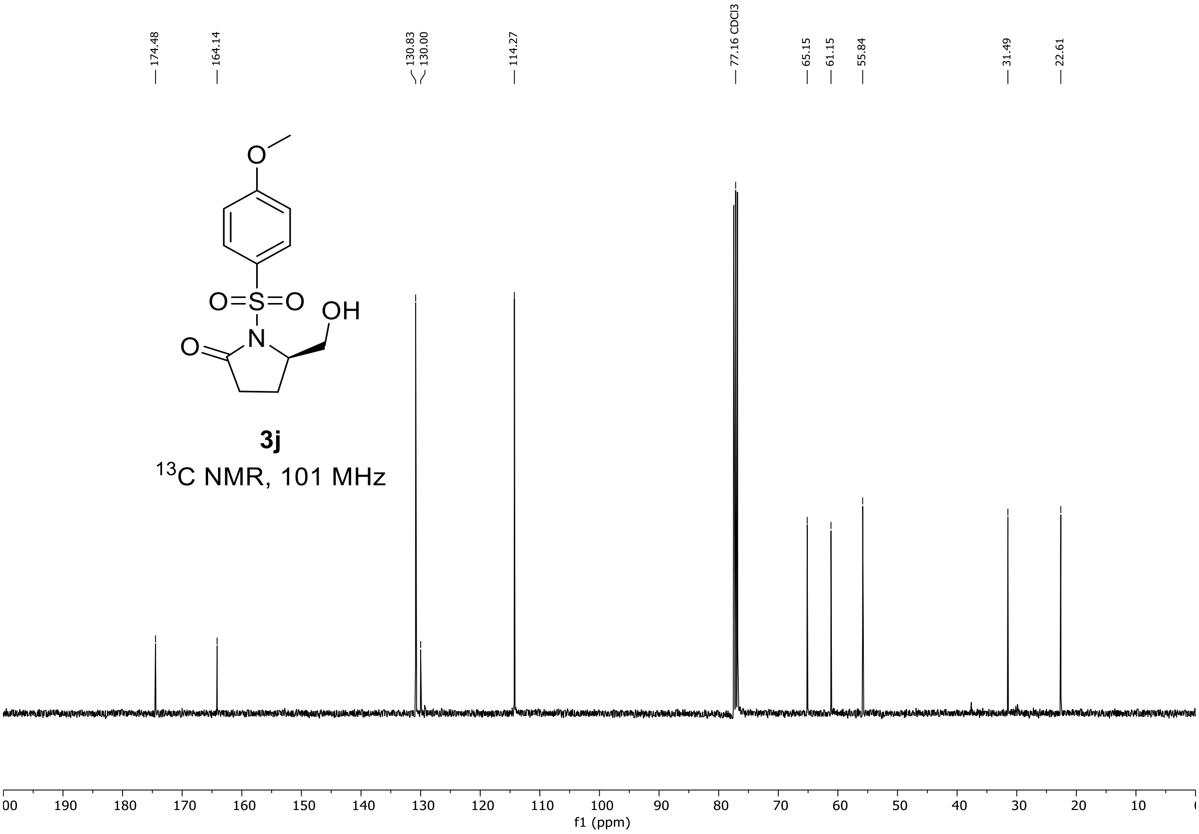

3j
^{13}C NMR, 101 MHz

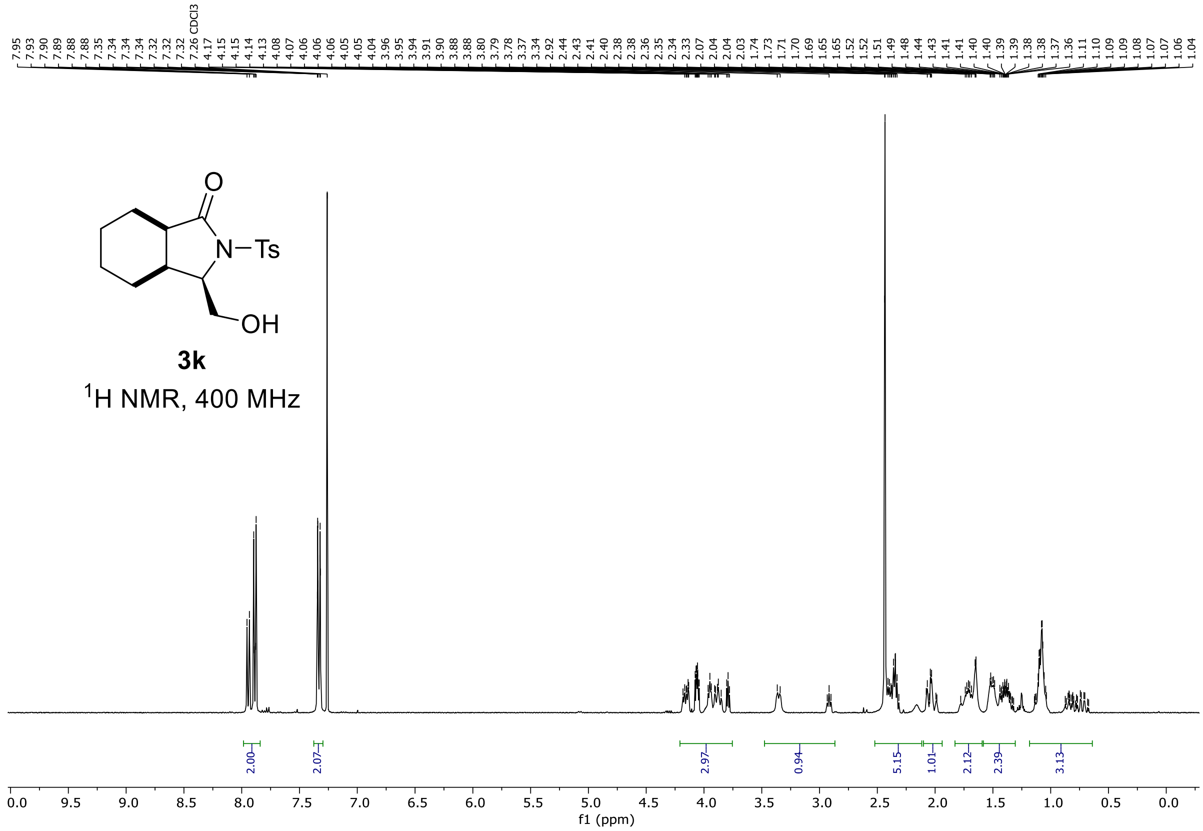
7.95 7.93 7.90 7.89 7.88 7.88 7.35 7.34 7.34 7.34 7.32 7.32 7.32 7.26 CDCl3 4.17 4.15 4.15 4.14 4.13 4.08 4.07 4.06 4.06 4.06 4.05 4.05 4.04 3.96 3.95 3.94 3.91 3.90 3.88 3.80 3.79 3.78 3.37 3.34 2.92 2.44 2.43 2.41 2.40 2.38 2.38 2.36 2.35 2.34 2.33 2.07 2.04 2.04 2.03 1.74 1.73 1.71 1.70 1.69 1.65 1.65 1.52 1.52 1.51 1.51 1.49 1.48 1.44 1.43 1.41 1.41 1.40 1.40 1.39 1.39 1.38 1.38 1.37 1.36 1.11 1.10 1.09 1.09 1.08 1.07 1.06 1.04
O
N−Ts
OH
3k
1H NMR, 400 MHz
2.00
2.07
2.97
0.94
5.15
1.01
2.12
2.39
3.13
0.0 9.5 9.0 8.5 8.0 7.5 7.0 6.5 6.0 5.5 5.0 4.5 4.0 3.5 3.0 2.5 2.0 1.5 1.0 0.5 0.0
f1 (ppm)

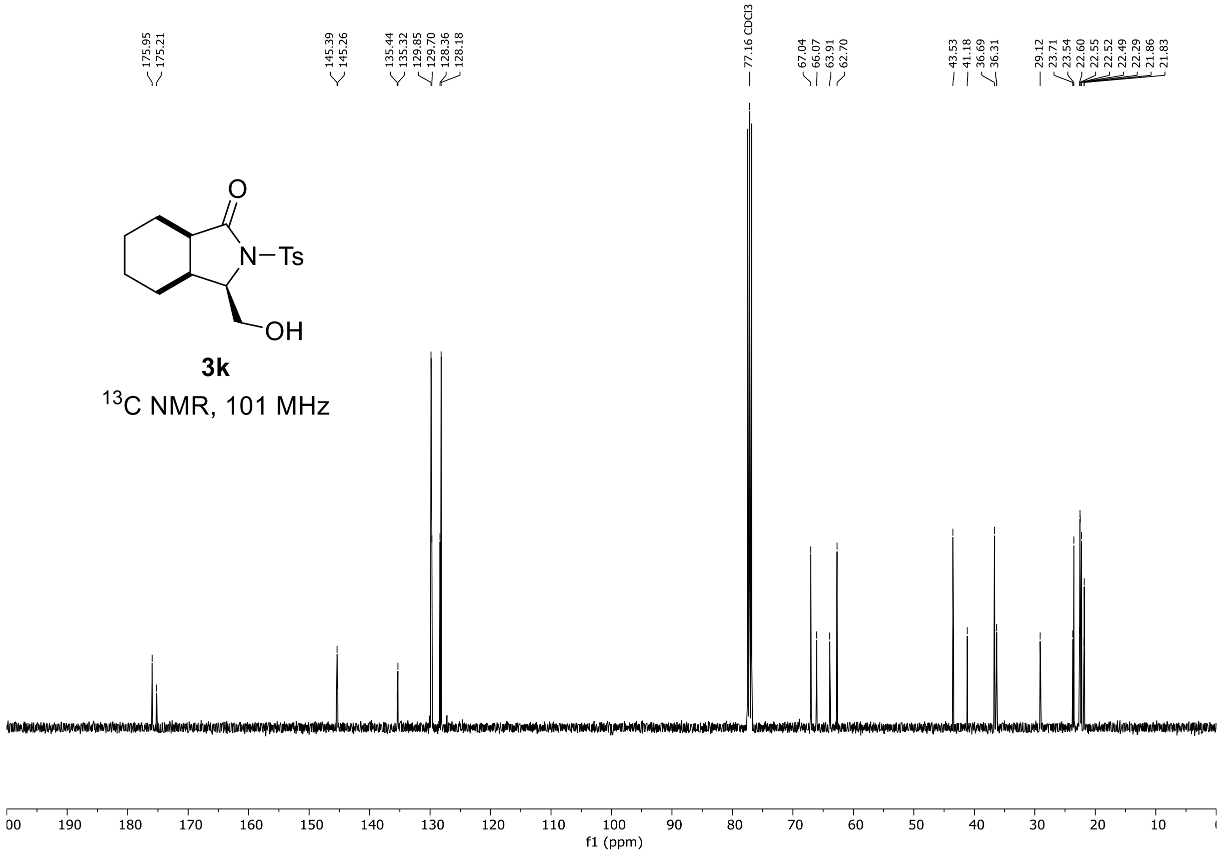
175.95 175.21
145.39 145.26
135.44 135.32 129.85 129.70 128.36 128.18
77.16 CDCl3
67.04 66.07 63.91 62.70
43.53 41.18 36.69 36.31
29.12 23.71 23.54 22.60 22.55 22.52 22.49 22.29 21.86 21.83
O
N−Ts
OH
3k
13C NMR, 101 MHz
00 190 180 170 160 150 140 130 120 110 100 90 80 70 60 50 40 30 20 10
f1 (ppm)

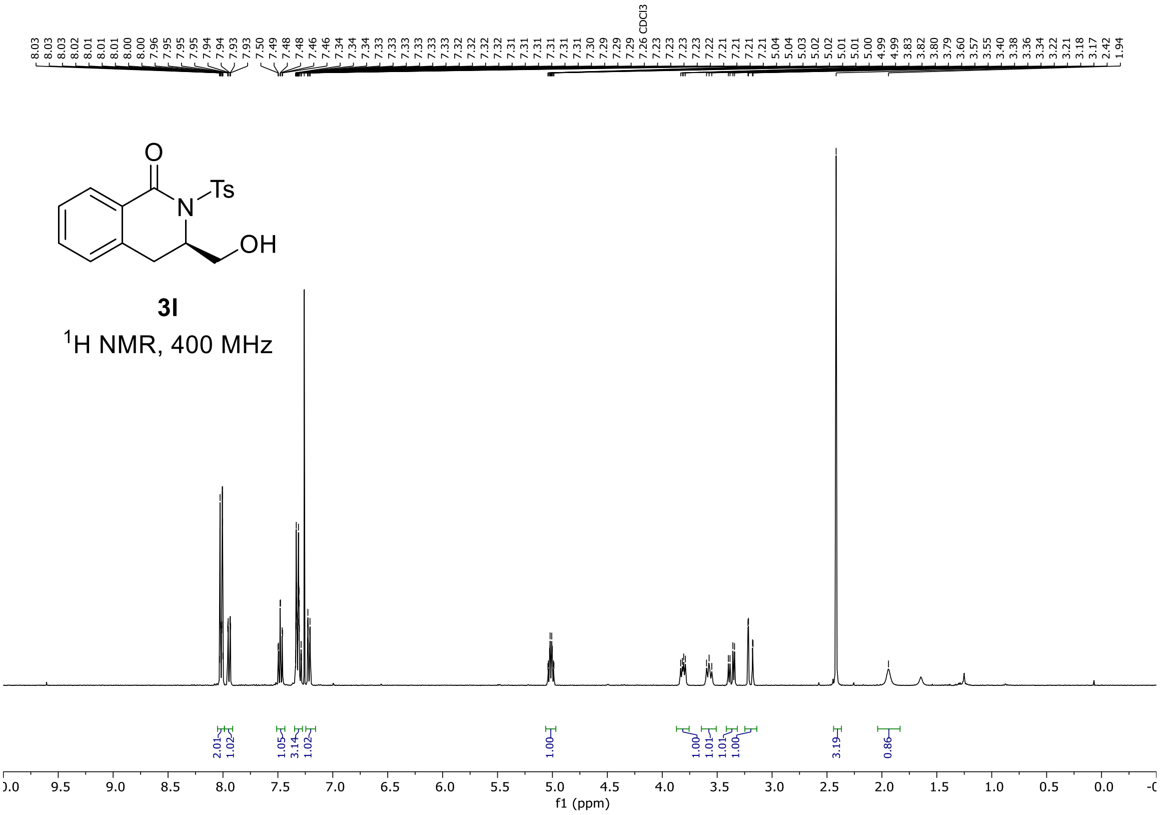
8.03 8.03 8.03 8.02 8.01 8.01 8.01 8.00 8.00 7.96 7.95 7.95 7.95 7.94 7.94 7.93 7.93 7.50 7.49 7.48 7.48 7.46 7.46 7.34 7.34 7.34 7.33 7.33 7.33 7.33 7.33 7.33 7.32 7.32 7.32 7.32 7.31 7.31 7.31 7.31 7.31 7.30 7.29 7.29 7.26 CDCl3 7.23 7.23 7.23 7.22 7.21 7.21 7.21 7.21 5.04 5.04 5.03 5.02 5.02 5.01 5.01 5.00 4.99 4.99 3.83 3.82 3.80 3.79 3.60 3.57 3.55 3.40 3.38 3.36 3.34 3.22 3.21 3.18 3.17 2.42 1.94
O
N
Ts
OH
3l
1H NMR, 400 MHz
2.01 1.02 1.05 3.14 1.02 1.00 1.00 1.01 1.01 1.00 3.19 0.86
f1 (ppm)

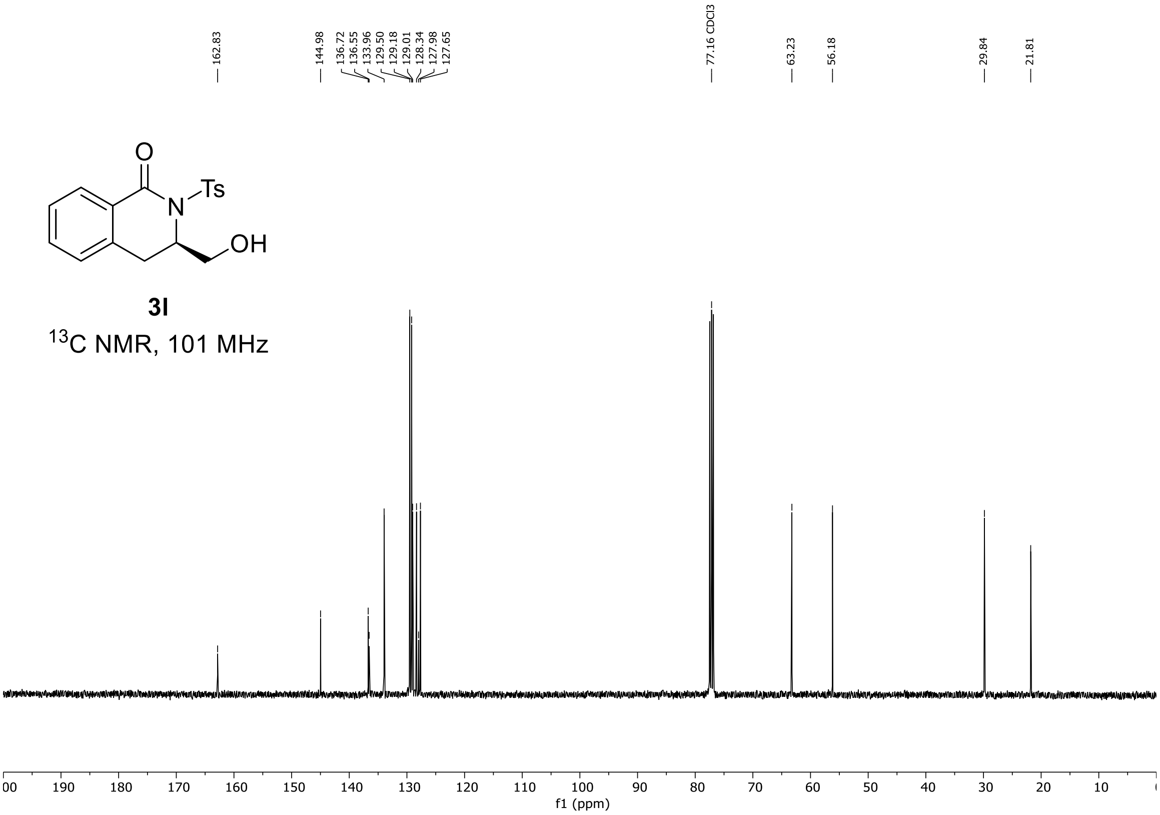
162.83 144.98 136.72 136.55 133.96 129.50 129.18 129.01 128.34 127.98 127.65 77.16 CDCl3 63.23 56.18 29.84 21.81
O
N
Ts
OH
3l
13C NMR, 101 MHz
f1 (ppm)

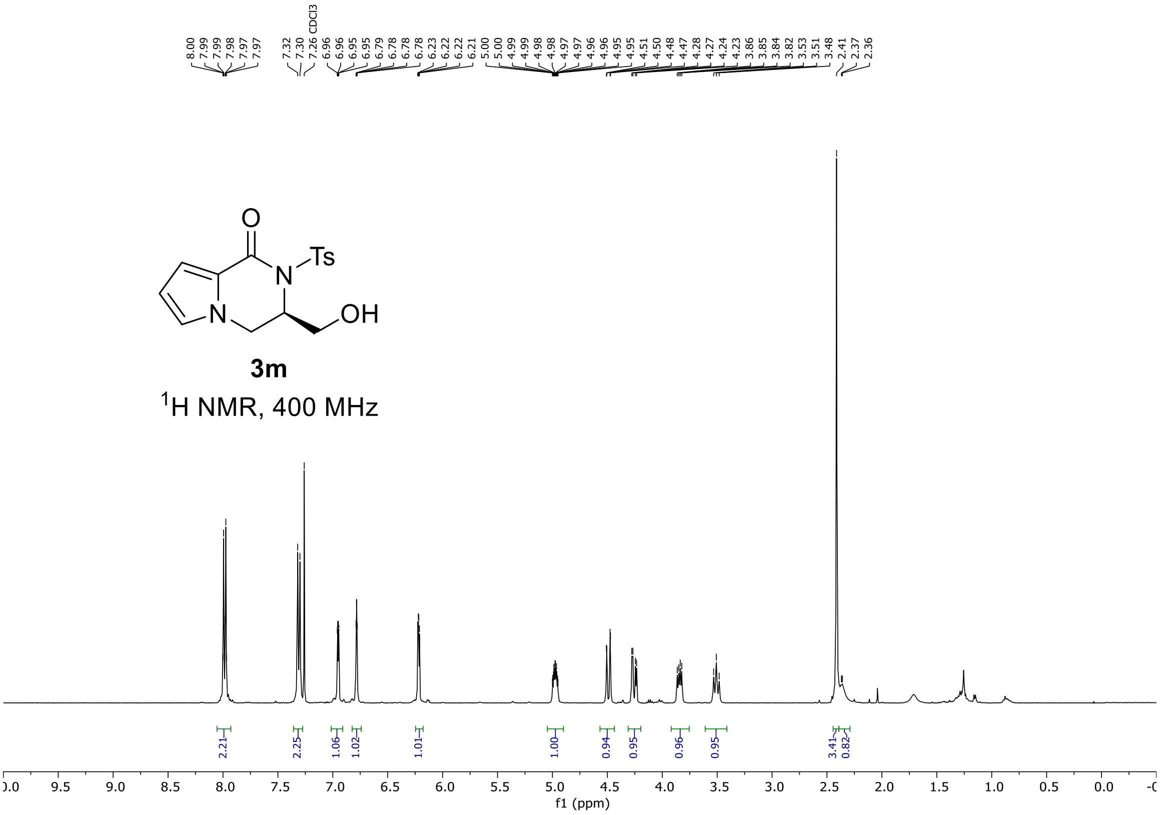
O
N
Ts
N
OH
3m
¹H NMR, 400 MHz
f1 (ppm)

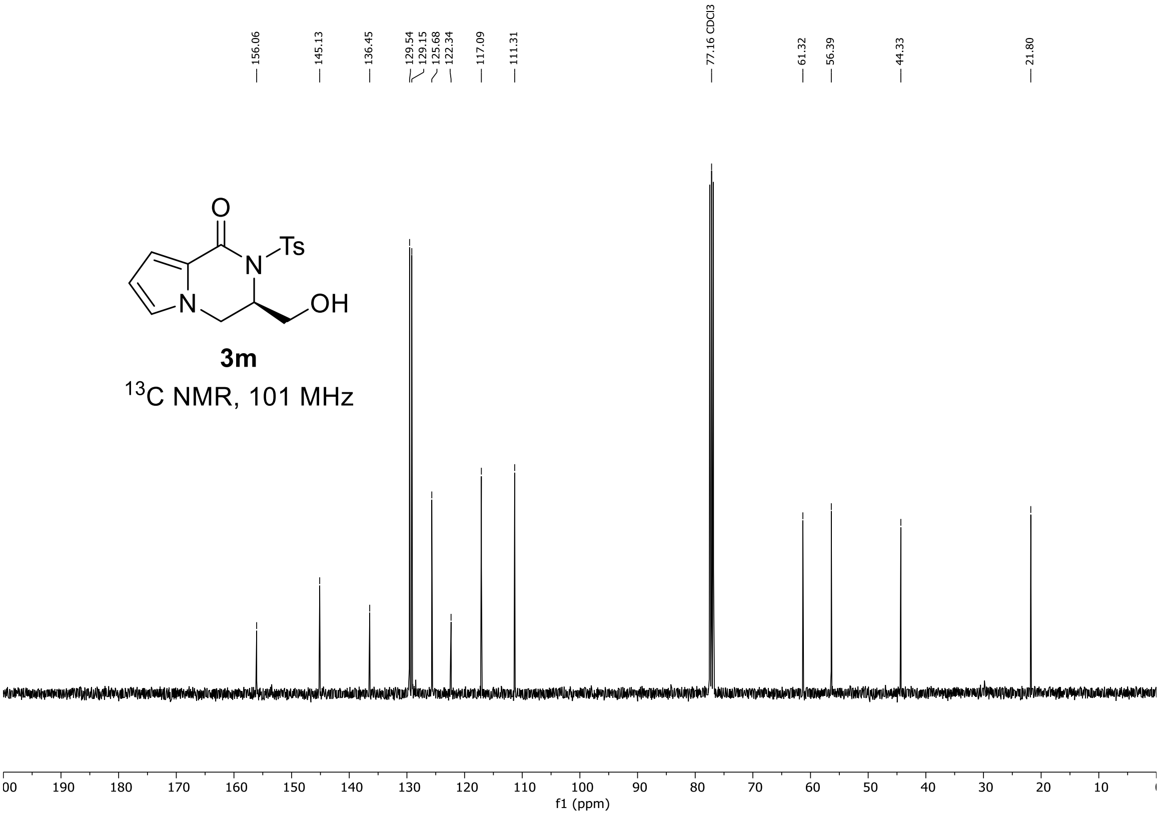
156.06
145.13
136.45
129.54
129.15
125.68
122.34
117.09
111.31
77.16 CDCl3
61.32
56.39
44.33
21.80
O
N
Ts
N
OH
3m
¹³C NMR, 101 MHz
f1 (ppm)

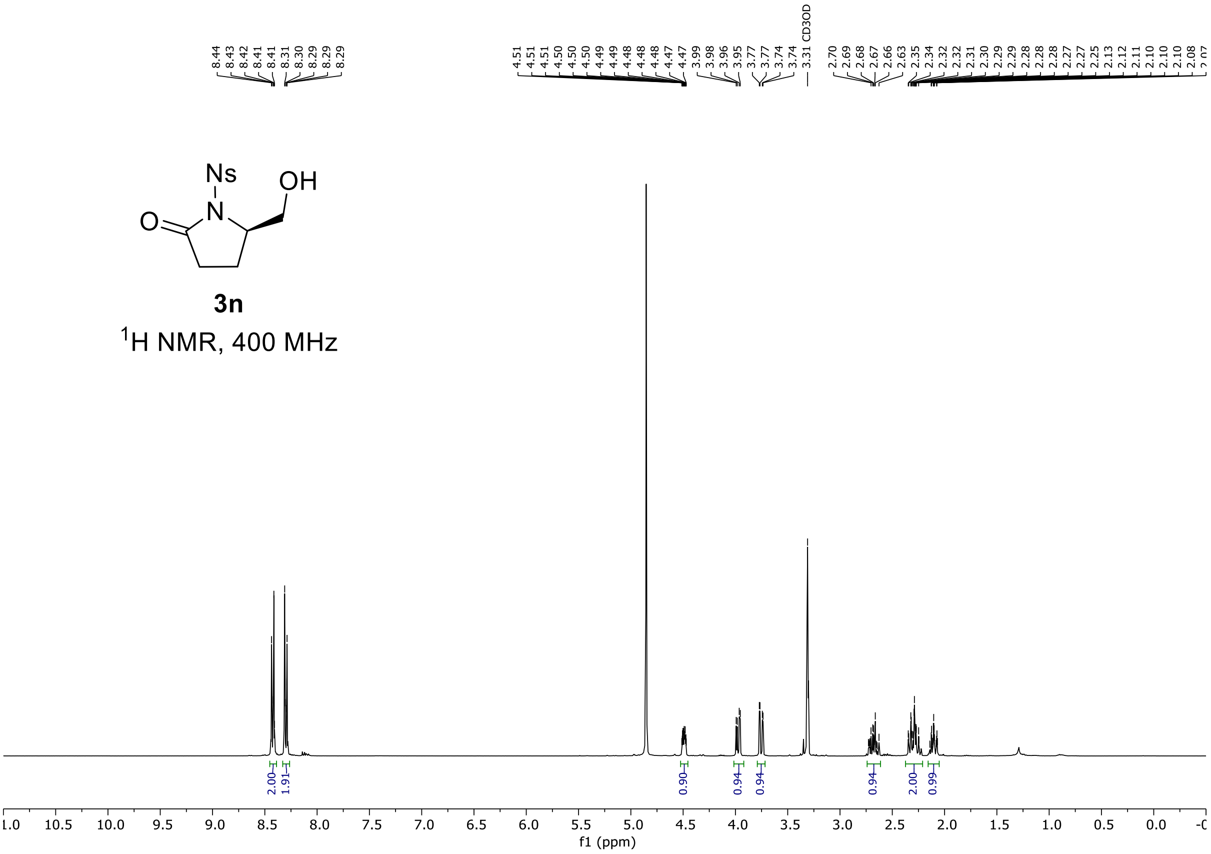
8.44
8.43
8.42
8.41
8.31
8.30
8.29
8.29
8.29
4.51
4.51
4.51
4.50
4.50
4.49
4.49
4.48
4.48
4.48
4.47
4.47
3.99
3.98
3.96
3.95
3.77
3.77
3.74
3.74
3.31 CD3OD
2.70
2.69
2.68
2.67
2.66
2.63
2.35
2.34
2.32
2.32
2.31
2.30
2.29
2.29
2.28
2.28
2.28
2.27
2.27
2.25
2.13
2.12
2.11
2.10
2.10
2.08
Ns
OH
O
N
3n
¹H NMR, 400 MHz
2.00
1.91
0.90
0.94
0.94
0.94
2.00
0.99
f1 (ppm)

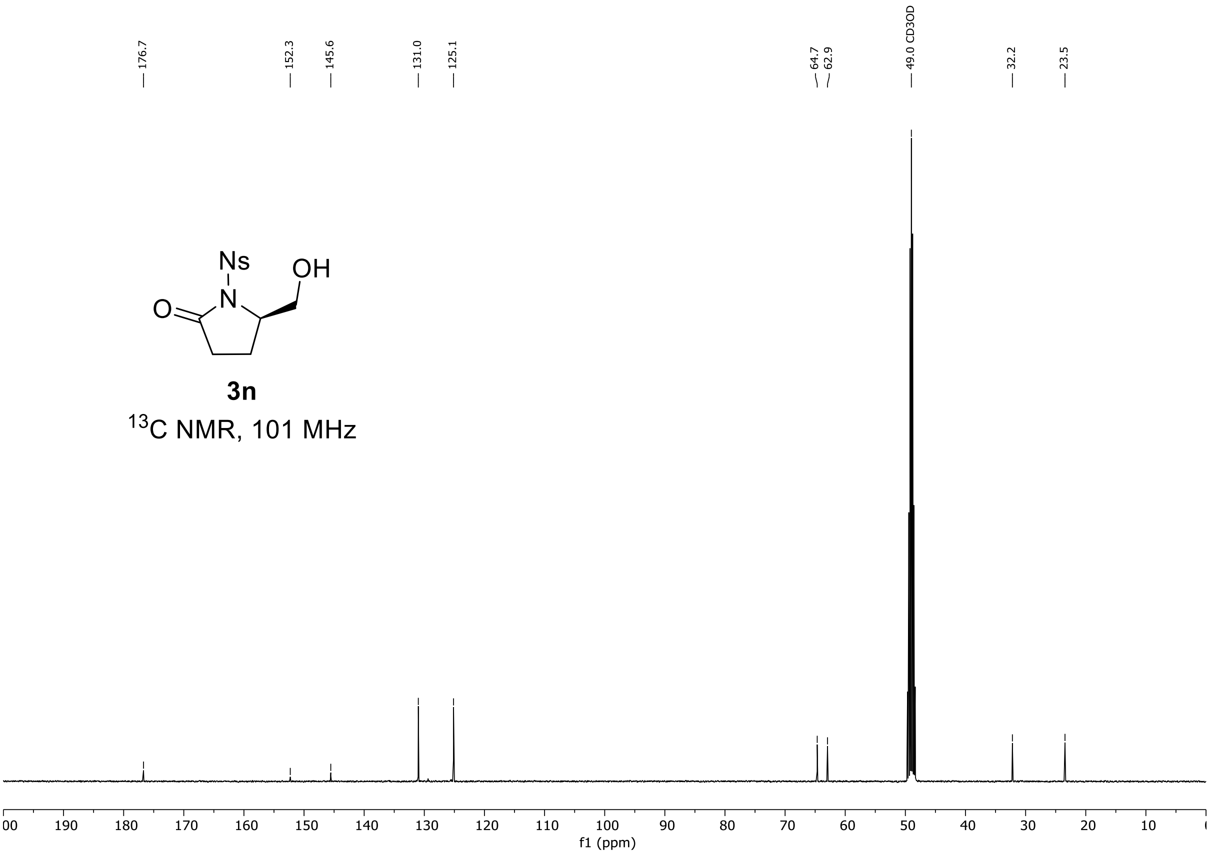
176.7
152.3
145.6
131.0
125.1
64.7
62.9
49.0 CD3OD
32.2
23.5
Ns
OH
O
N
3n
¹³C NMR, 101 MHz
f1 (ppm)

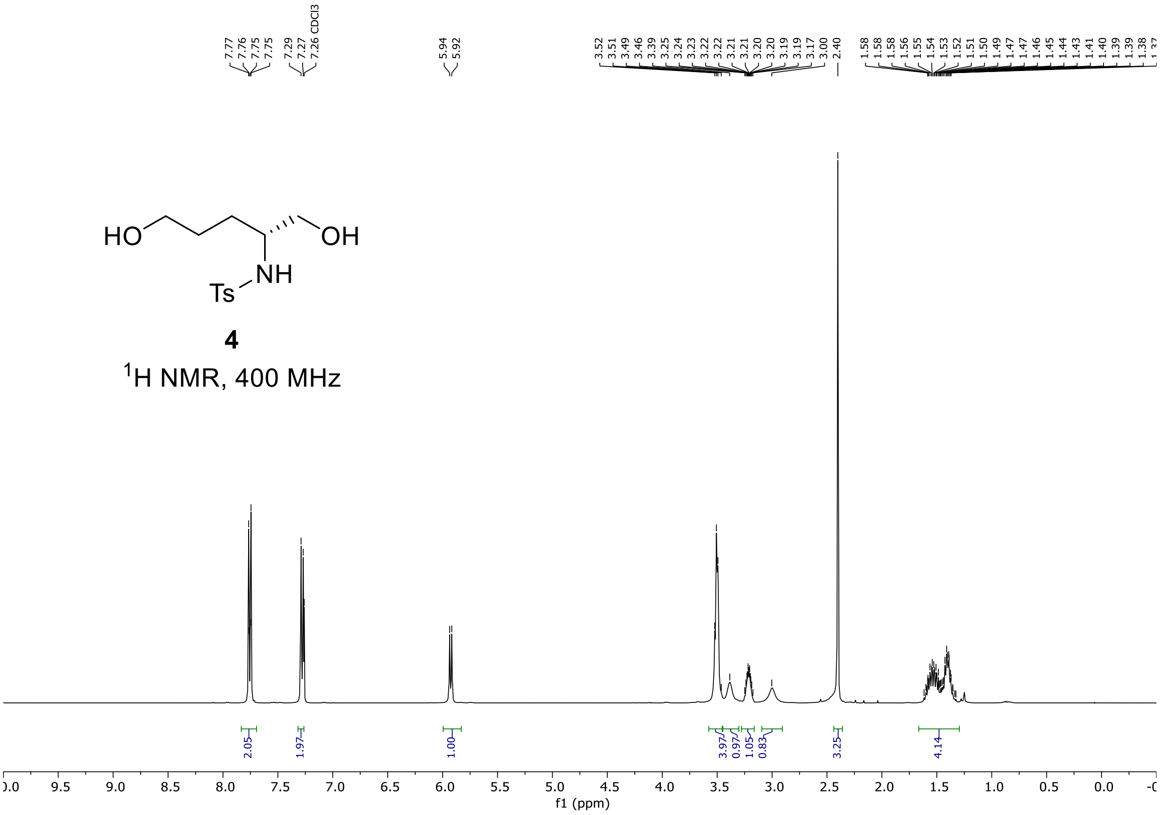
7.77
7.76
7.75
7.75
7.29
7.27
7.26 CDCl3
5.94
5.92
3.52
3.51
3.49
3.46
3.39
3.25
3.24
3.23
3.22
3.22
3.21
3.21
3.20
3.20
3.19
3.19
3.17
3.00
2.40
HO
OH
NH
Ts
4
1H NMR, 400 MHz
2.05
1.97
1.00
3.97
0.97
1.05
0.83
3.25
4.14
f1 (ppm)

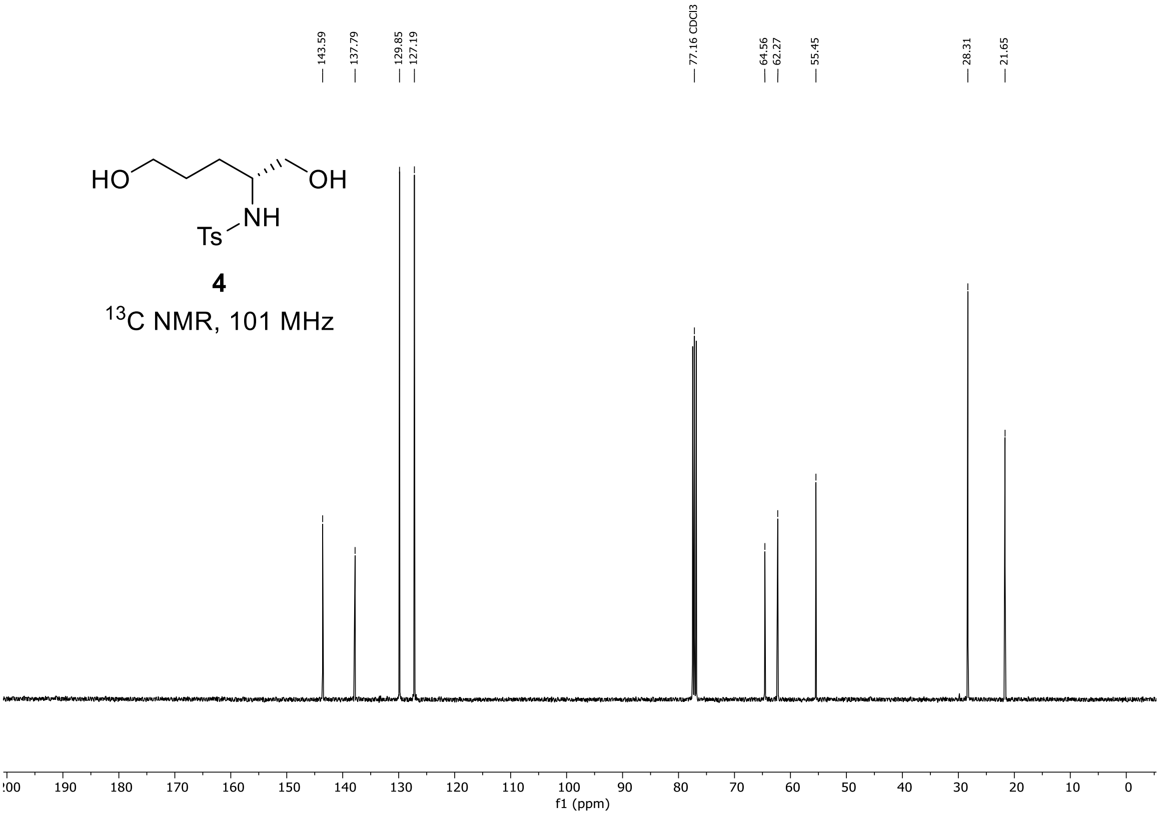
143.59
137.79
129.85
127.19
77.16 CDCl3
64.56
62.27
55.45
28.31
21.65
HO
OH
NH
Ts
4
13C NMR, 101 MHz
f1 (ppm)

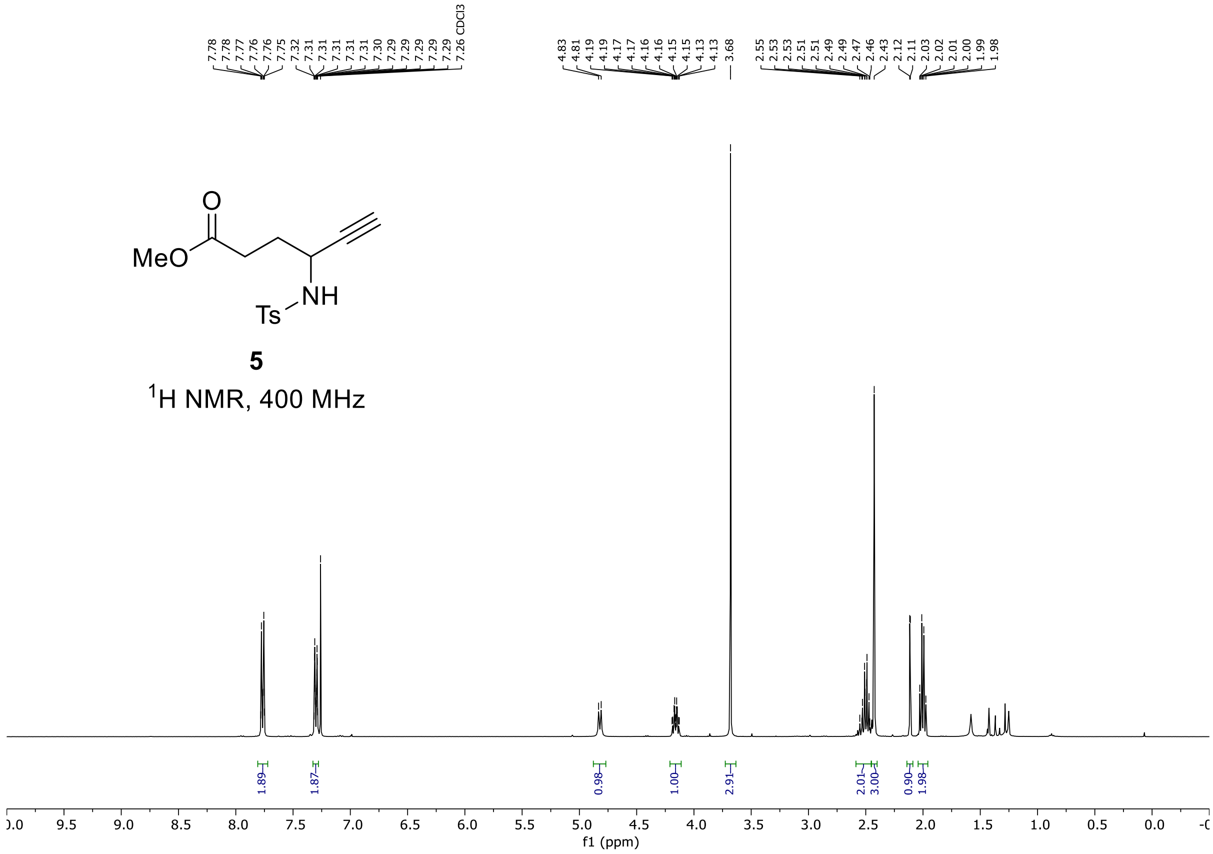

7.78 7.78 7.77 7.76 7.76 7.75 7.32 7.31 7.31 7.31 7.31 7.30 7.29 7.29 7.29 7.29 7.29 7.26 CDCl3
4.83 4.81 4.19 4.19 4.17 4.17 4.16 4.15 4.15 4.13 4.13
3.68
2.55 2.53 2.53 2.51 2.51 2.49 2.49 2.47 2.46 2.43 2.12 2.11 2.03 2.02 2.01 2.00 1.99 1.98
MeO
O
NH
Ts
5
1H NMR, 400 MHz
1.89 1.87 0.98 1.00 2.91 2.01 3.00 0.90 1.98
f1 (ppm)

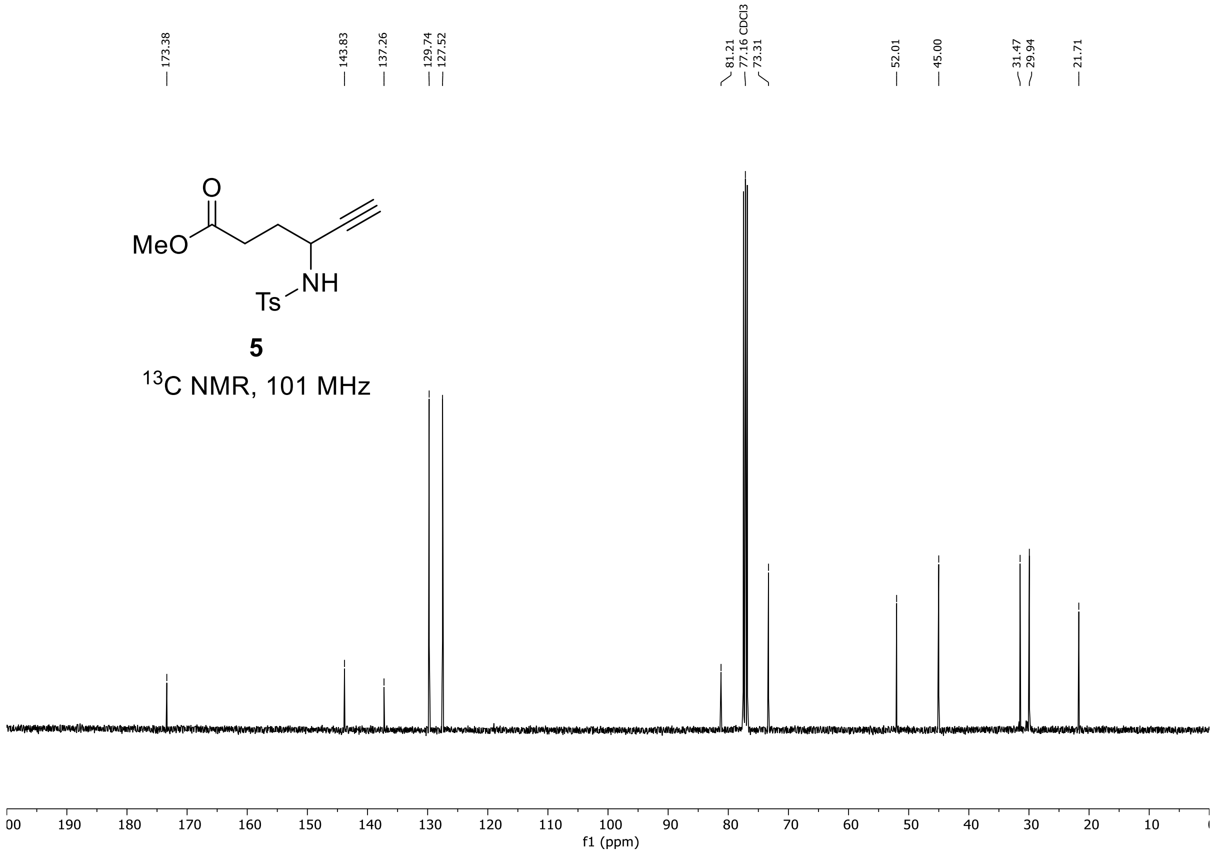

173.38
143.83
137.26
129.74
127.52
81.21
77.16 CDCl3
73.31
52.01
45.00
31.47
29.94
21.71
MeO
O
NH
Ts
5
13C NMR, 101 MHz
f1 (ppm)

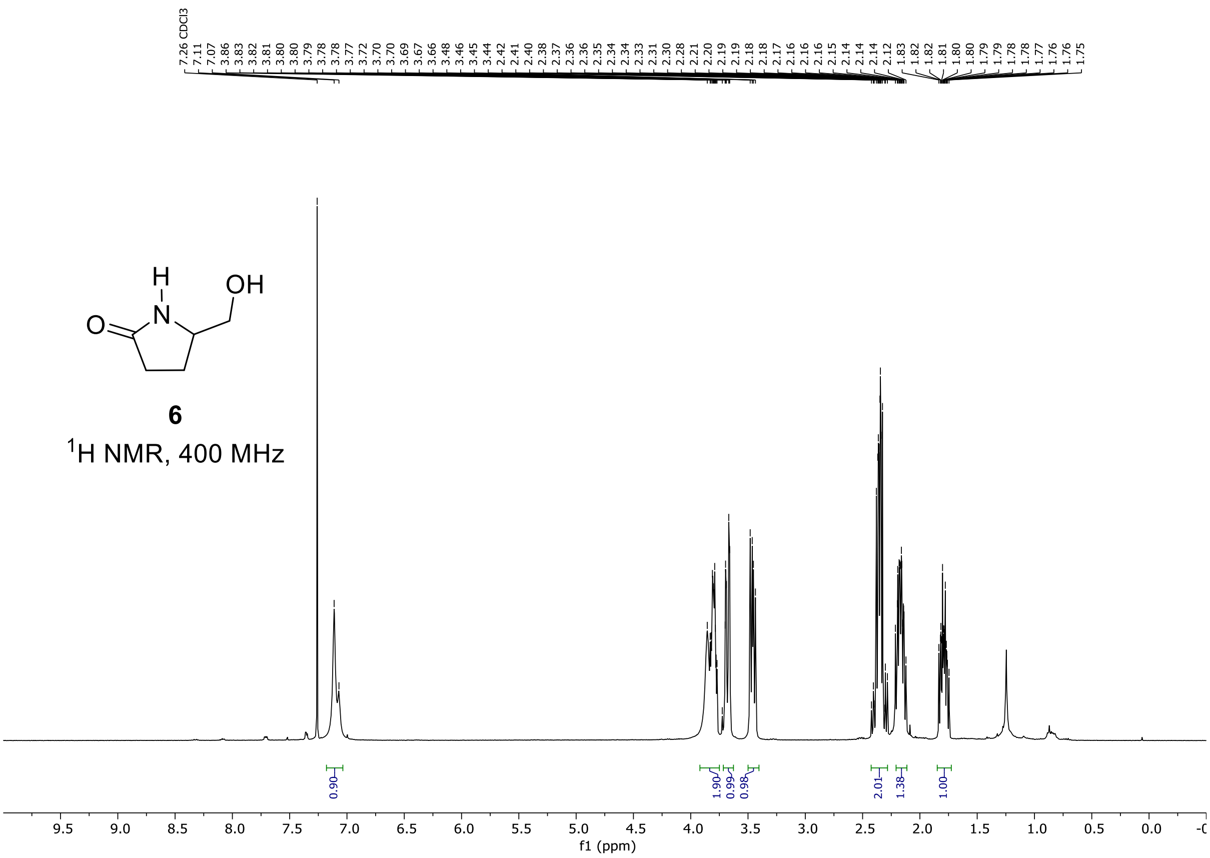
7.26 CDCl3
7.11
7.07
3.86
3.83
3.82
3.81
3.80
3.80
3.79
3.78
3.78
3.77
3.72
3.70
3.70
3.69
3.67
3.66
3.48
3.46
3.45
3.44
2.42
2.41
2.40
2.38
2.37
2.36
2.36
2.35
2.34
2.34
2.33
2.31
2.30
2.28
2.21
2.20
2.19
2.19
2.18
2.18
2.17
2.16
2.16
2.15
2.14
2.14
2.14
2.12
1.83
1.82
1.82
1.81
1.80
1.80
1.79
1.79
1.78
1.77
1.76
1.76
1.75
H
OH
N
O
6
1H NMR, 400 MHz
0.90
1.90
0.99
0.98
2.01
1.38
1.00
f1 (ppm)

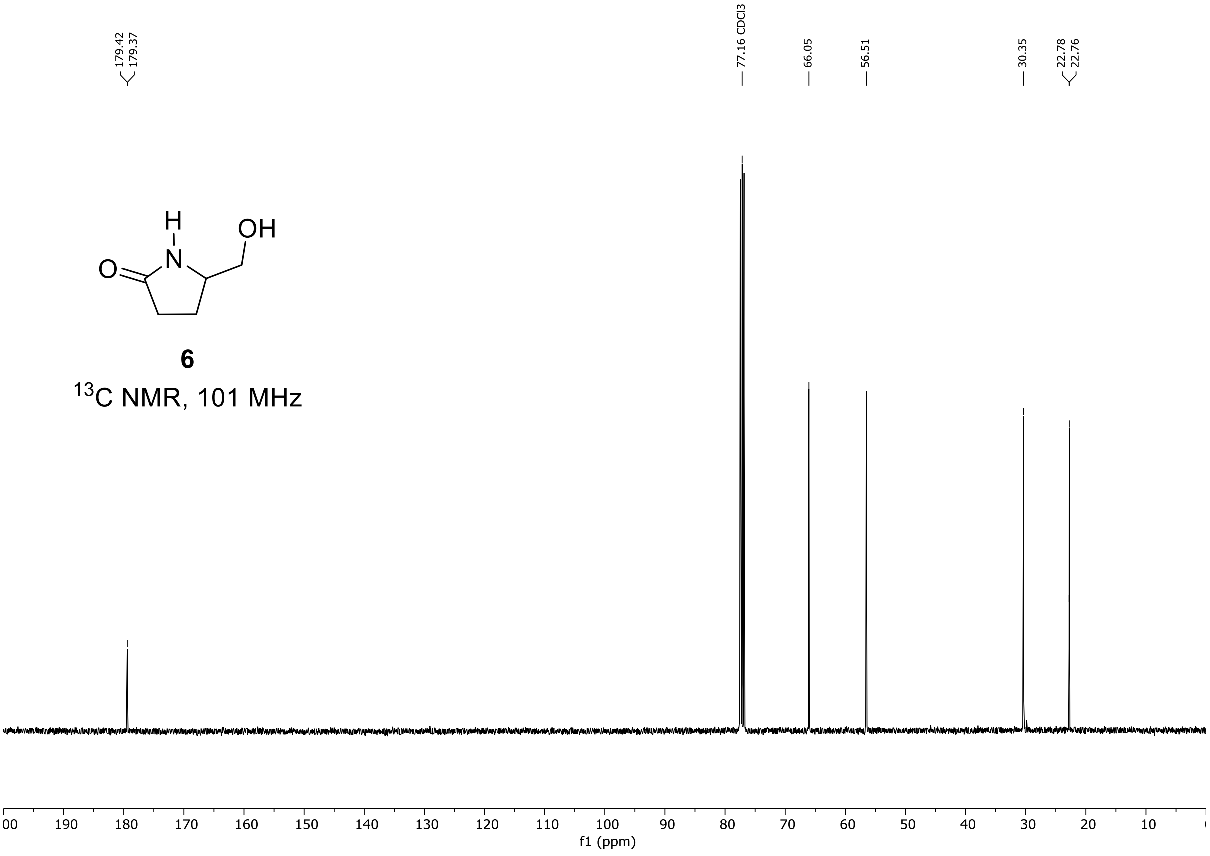
179.42
179.37
77.16 CDCl3
66.05
56.51
30.35
22.78
22.76
H
OH
N
O
6
13C NMR, 101 MHz
f1 (ppm)

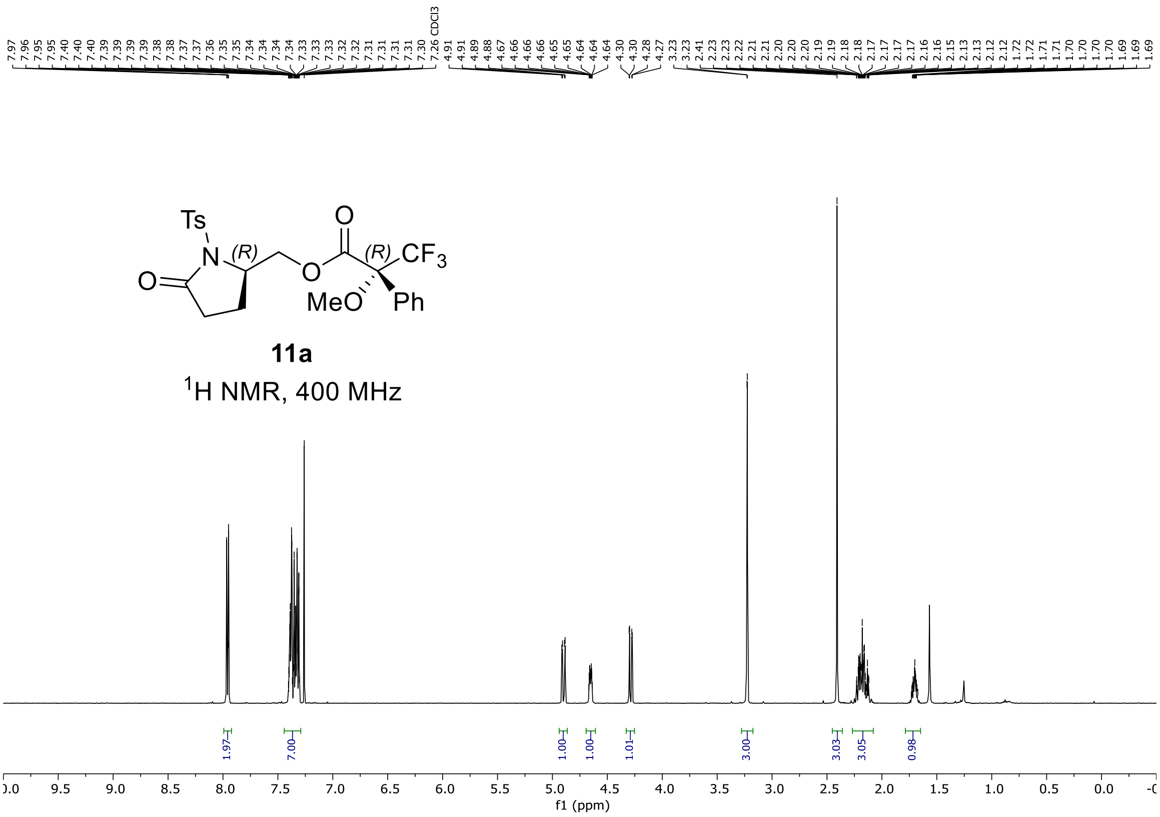
7.97 7.96 7.95 7.95 7.40 7.40 7.39 7.39 7.39 7.39 7.38 7.38 7.37 7.37 7.36 7.35 7.35 7.34 7.34 7.34 7.34 7.33 7.33 7.33 7.32 7.32 7.31 7.31 7.31 7.31 7.30 7.26 CDCl3 4.91 4.91 4.89 4.88 4.67 4.66 4.66 4.66 4.65 4.65 4.64 4.64 4.64 4.30 4.30 4.28 4.27 3.23 3.23 2.41 2.23 2.23 2.22 2.21 2.21 2.20 2.20 2.20 2.19 2.19 2.18 2.18 2.17 2.17 2.17 2.17 2.16 2.16 2.15 2.13 2.13 2.12 2.12 1.72 1.72 1.71 1.71 1.70 1.70 1.70 1.69 1.69 1.69
Ts
N
(R)
O
O
O
(R)
CF3
MeO
Ph
11a
1H NMR, 400 MHz
1.97
7.00
1.00
1.00
1.01
3.00
3.03
3.05
0.98
f1 (ppm)

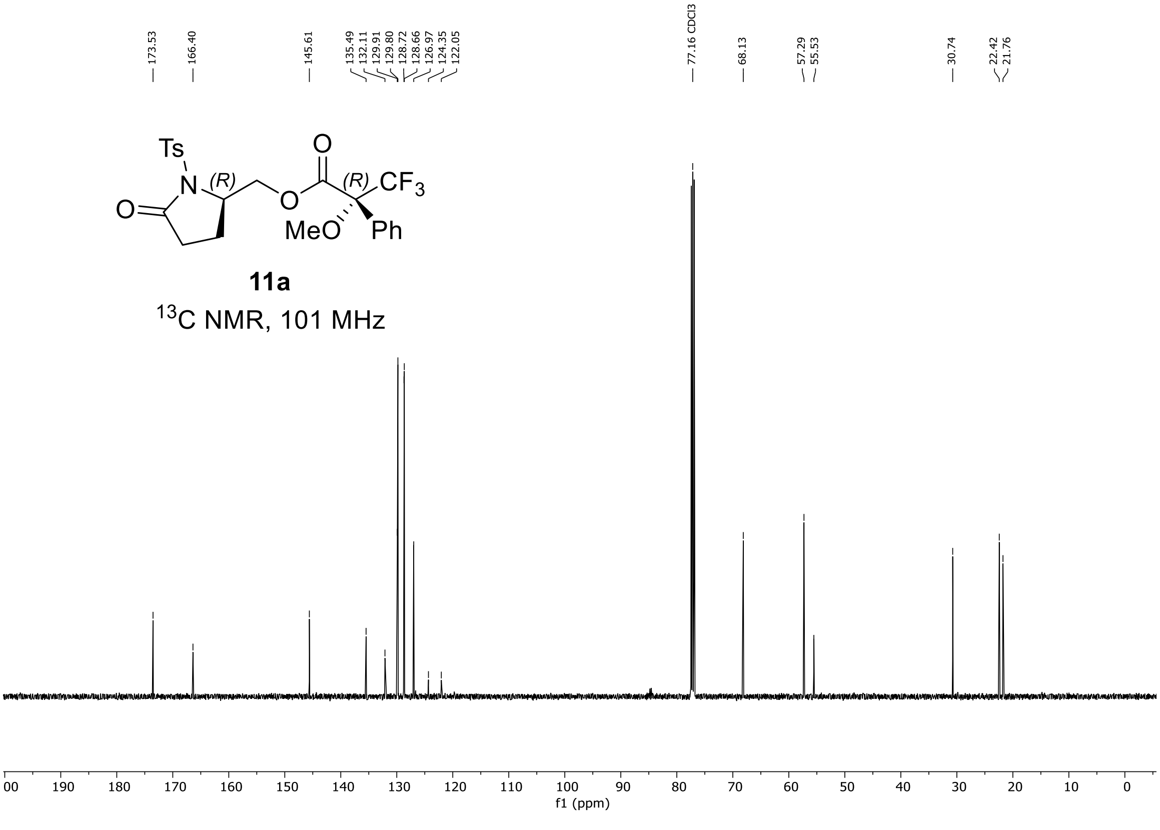
173.53
166.40
145.61
135.49 132.11 129.91 129.80 128.72 128.66 126.97 124.35 122.05
77.16 CDCl3
68.13
57.29 55.53
30.74
22.42 21.76
Ts
N
(R)
O
O
O
(R)
CF3
MeO
Ph
11a
13C NMR, 101 MHz
f1 (ppm)

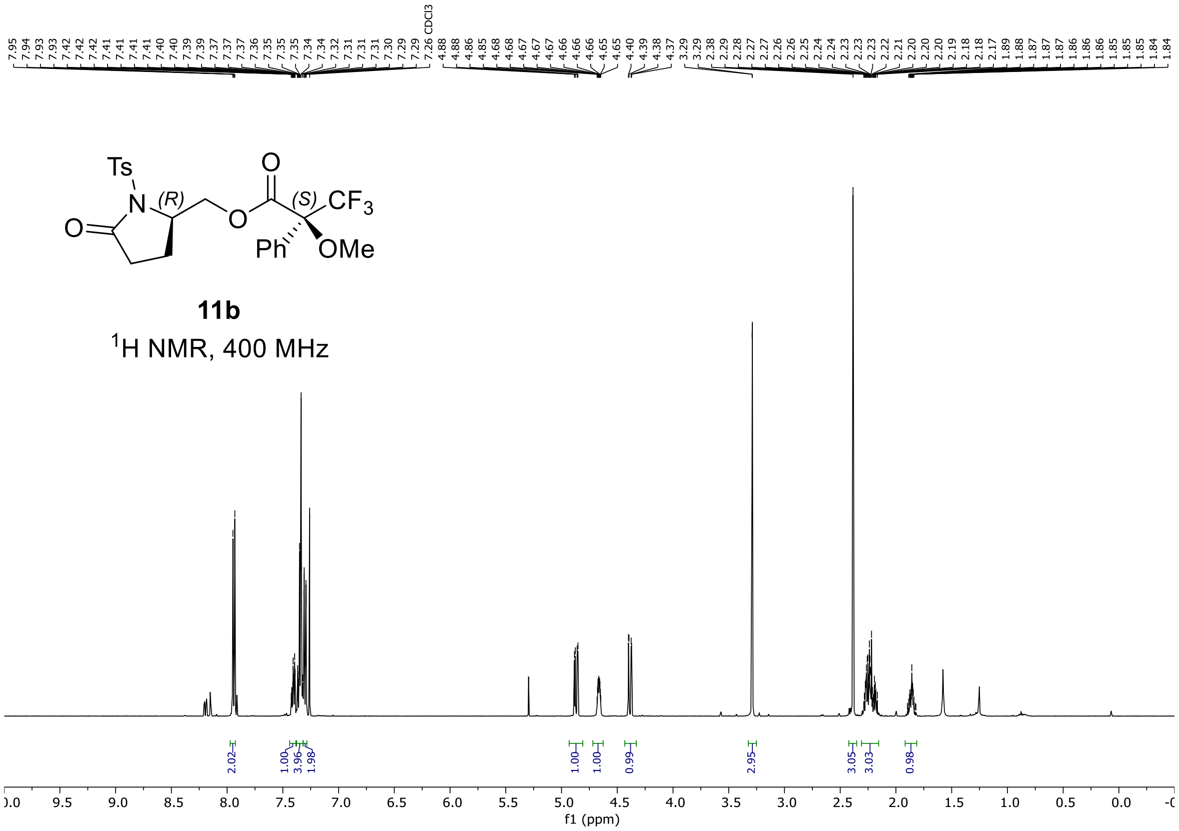
7.95 7.94 7.93 7.42 7.42 7.41 7.41 7.41 7.40 7.39 7.39 7.37 7.37 7.36 7.35 7.35 7.35 7.34 7.34 7.32 7.31 7.31 7.31 7.30 7.29 7.29 7.26 CDCl3 4.88 4.88 4.86 4.85 4.68 4.67 4.67 4.67 4.66 4.66 4.66 4.65 4.65 4.40 4.39 4.38 4.37 3.29 2.38 2.29 2.28 2.27 2.27 2.26 2.26 2.25 2.24 2.24 2.23 2.23 2.23 2.22 2.21 2.20 2.20 2.19 2.18 2.18 2.17 1.89 1.88 1.87 1.87 1.87 1.86 1.86 1.86 1.85 1.85 1.85 1.84 1.84
Ts
N
(R)
O
O
O
(S)
CF3
Ph
OMe
11b
1H NMR, 400 MHz
2.02 1.00 3.96 1.98 1.00 1.00 0.99 2.95 3.05 3.03 0.98
f1 (ppm)

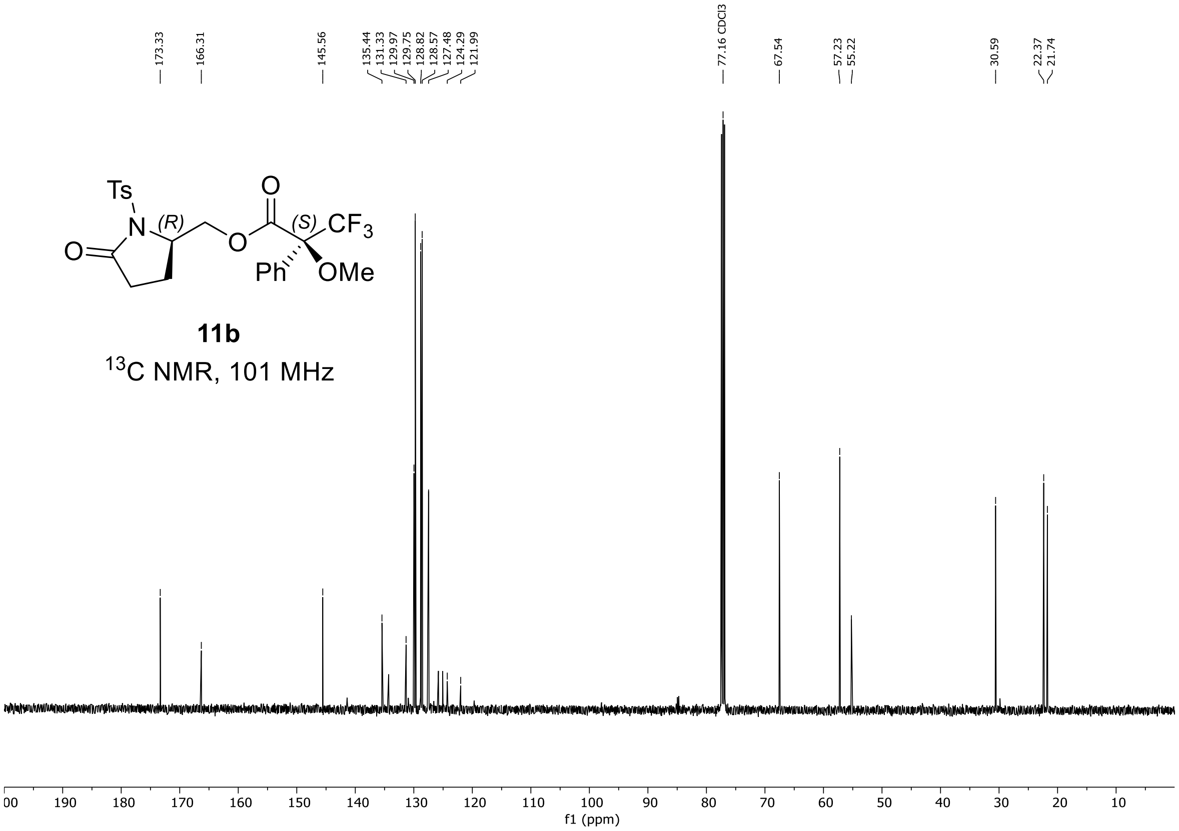
173.33 166.31 145.56 135.44 131.33 129.97 129.75 128.82 128.57 127.48 124.29 121.99 77.16 CDCl3 67.54 57.23 55.22 30.59 22.37 21.74
Ts
N
(R)
O
O
O
(S)
CF3
Ph
OMe
11b
13C NMR, 101 MHz
f1 (ppm)
